# Identifying Selected Regions from Heterozygosity and Divergence Using a Light-Coverage Genomic Dataset from Two Human Populations

**DOI:** 10.1371/journal.pone.0001712

**Published:** 2008-03-05

**Authors:** Taras K. Oleksyk, Kai Zhao, Francisco M. De La Vega, Dennis A. Gilbert, Stephen J. O'Brien, Michael W. Smith

**Affiliations:** 1 Laboratory of Genomic Diversity, National Cancer Institute at Frederick, Frederick, Maryland, United States of America; 2 Basic Research Program, SAIC-Frederick, Inc., National Cancer Institute at Frederick, Frederick, Maryland, United States of America; 3 Applied Biosystems, Foster City, California, United States of America; Indiana University, United States of America

## Abstract

When a selective sweep occurs in the chromosomal region around a target gene in two populations that have recently separated, it produces three dramatic genomic consequences: 1) decreased multi-locus heterozygosity in the region; 2) elevated or diminished genetic divergence (F_ST_) of multiple polymorphic variants adjacent to the selected locus between the divergent populations, due to the alternative fixation of alleles; and 3) a consequent regional increase in the variance of F_ST_ (S^2^F_ST_) for the same clustered variants, due to the increased alternative fixation of alleles in the loci surrounding the selection target. In the first part of our study, to search for potential targets of directional selection, we developed and validated a resampling-based computational approach; we then scanned an array of 31 different-sized moving windows of SNP variants (5–65 SNPs) across the human genome in a set of European and African American population samples with 183,997 SNP loci after correcting for the recombination rate variation. The analysis revealed 180 regions of recent selection with very strong evidence in either population or both. In the second part of our study, we compared the newly discovered putative regions to those sites previously postulated in the literature, using methods based on inspecting patterns of linkage disequilibrium, population divergence and other methodologies. The newly found regions were cross-validated with those found in nine other studies that have searched for selection signals. Our study was replicated especially well in those regions confirmed by three or more studies. These validated regions were independently verified, using a combination of different methods and different databases in other studies, and should include fewer false positives. The main strength of our analysis method compared to others is that it does not require dense genotyping and therefore can be used with data from population-based genome SNP scans from smaller studies of humans or other species.

## Introduction

Patterns of allelic variation in the genome are shaped by successes and failures of genes influenced by evolutionary forces acting throughout population history. When a genetic variant becomes adaptive, populations experience changes in allele frequencies that reflect the strength and recurrence of the selective pressure(s). By identifying these residual footprints of genomic evolutionary processes in the form of “signatures of selection,” we hope to gain valuable insight into the evolutionary past of a species. A principal selection signature involves the local reduction in variation within the selected gene, as well as in adjacent SNP variants, around the selected chromosomal region known as “selective seep” [1]. Further, when two isolated populations are examined, one of which underwent strong selection in the past but the other did not, the frequencies of the selected SNP and adjacent alleles will often be more different between the populations than expected under the assumption of neutral genetic drift [2]–[4]. In addition, selection affects chromosomal segments, not simply individual SNPs, thus creating complex patterns of allele frequencies in the regions immediately surrounding the targeted site. In this study, we explore patterns of reduced heterozygosity and elevated between-population allele differentiation to identify strong selection signatures in the human genome. The method can also be applied to other diploid species when light-coverage SNP allele frequency genome scans of similar magnitude become available.

To address these aspects as well as to explore a new approach, we designed and tested a strategy for revealing footprints of recent selection first by simulation and then based upon a sample of 183,993 SNP markers genotyped in 45 European Americans and 45 African Americans (www.allsnp.com). This dataset was chosen since it is independent and has never been used to estimate selection signatures. It also represents a modest database size that is smaller than the sizes of current whole-genome genotyping human population studies based on existing genotyping technologies [5]. We used minimum information that would likely be available from such databases and searched for selective signatures by analysing three parameters observed for each SNP: 1) heterozygosity in European Americans (H_EA_); 2) heterozygosity in African Americans (H_AA_); and 3) F_ST_ between the corresponding individual SNPs in the two populations (F_ST_). Centered on each available SNP, 31 arrays (or windows) including 5–65 SNPs were sampled along all human chromosomes except Y to evaluate each window for: 1) average SNP heterozygosity in European Americans (H̀_EA_); 2) average SNP heterozygosity in African Americans (H̀_AA_); and 3) variance of F_ST_ among the adjacent SNPs (S^2^F_ST_). We used S^2^F_ST_ instead of the F_ST_ mean estimator from each group of adjacent SNPs, since a measure of F_ST_ mean across an array of loci would be more sensitive to those alleles that reached fixation in the form of the opposite allele, and less sensitive for those fixed in the same direction while variance captures this alternation.

Several previous analyses of selective signatures have appeared which have been based either on decreased heterozygosity (H), population differentiation (F_ST_), extended linkage disequilibrium, and even the premise that certain modern hereditary disease alleles were adaptive sometime in the past [3], [4], [6]–[15]. Discovered selection candidate regions included genes involved in development, immune defenses, reproduction, nutrition, behavior and other functions [16]. Although several of these regions have been discovered with multiple approaches (e.g., *CCR5*, *FY, LCT, G6PD, FOXP2* and others; Table S1, Notes S1), other provocative regions have not, raising issues around the context of different algorithms and approaches, the strength and mode of selection, the timing of imputed selective events, the influence of study design, and the validity of unreplicated regions. To test the validity of our approach, the results of our scan was applied to previously nominated regions to explore how well this method validated previous discoveries.

Finally, we incorporated nine other genome-wide or chromosome-wide attempts to find signatures of selection that included whole-genome searches for signatures of selection either by searching for the high values of local genomic divergence alone [4] or in combination with the allelic frequency spectrum [17], [18], looking for gene neighborhoods exhibiting extended linkage disequilibrium alone [9], [19], [20] or in combination with local genomic divergence [21], or by examining an aberrant frequency spectrum [22], [23]. We assessed the ten studies, including our own, and evaluated our findings in a multiple study comparison.

## Results and Discussion

### A resampling-based selection scan

**Figure 1:**
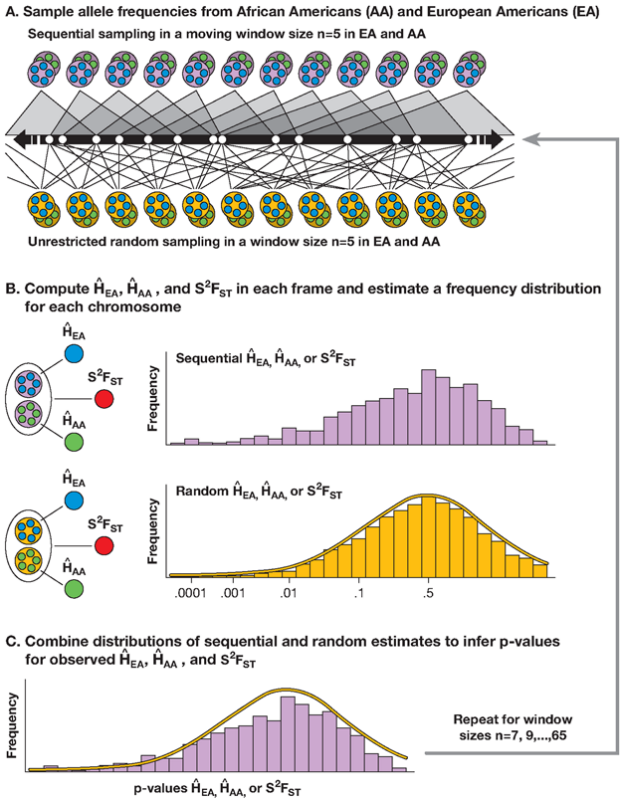
A flow chart for analyzing regions for local heterozygosity in African Americans (H̀_AA_) and European Americans (H̀_EA_), along with the variance of F_ST_ (S^2^F_ST_) to derive the lowest mean value fractional rank (λ) for each SNP. (A) Sampling and resampling process: (Top) We selected five adjacent SNPs at the beginning of a single chromosome and computed H̀_EA_, H̀_AA_ and S^2^F_ST _for the group, then moved the window sequentially to the right, one SNP at a time (observed values). (Bottom) To establish the baseline, we randomly resampled 10 million groups of five random SNPs on the same chromosome with replacements and computed H̀_EA_, H̀_AA_ and S^2^F_ST _(random values). (B) Determining observed and random distributions of H̀_EA,_ H̀_AA_ and S^2^F_ST_: (Top) We built a frequency distribution for each chromosome using observed values; (Bottom) we built a frequency distribution and assigned fractional rank values to the distribution of random values. (C) Superimposing the distributions to derive fractional rank values: the two distributions were combined and each observed value assigned a fractional rank from the closest larger random value. The same computation was done for all 31 SNP window group sizes (N = 5, 7, 9 … 65) 100 times, and mean values were calculated. For each SNP, the lowest mean value (λ) for H̀_EA_ was chosen from the 31 windows of size 5 to 65, and plotted across the length of each chromosome in cM. Likewise, the same derivation was applied to H̀_AA_ and S^2^F_ST_.

**Figure 2:**
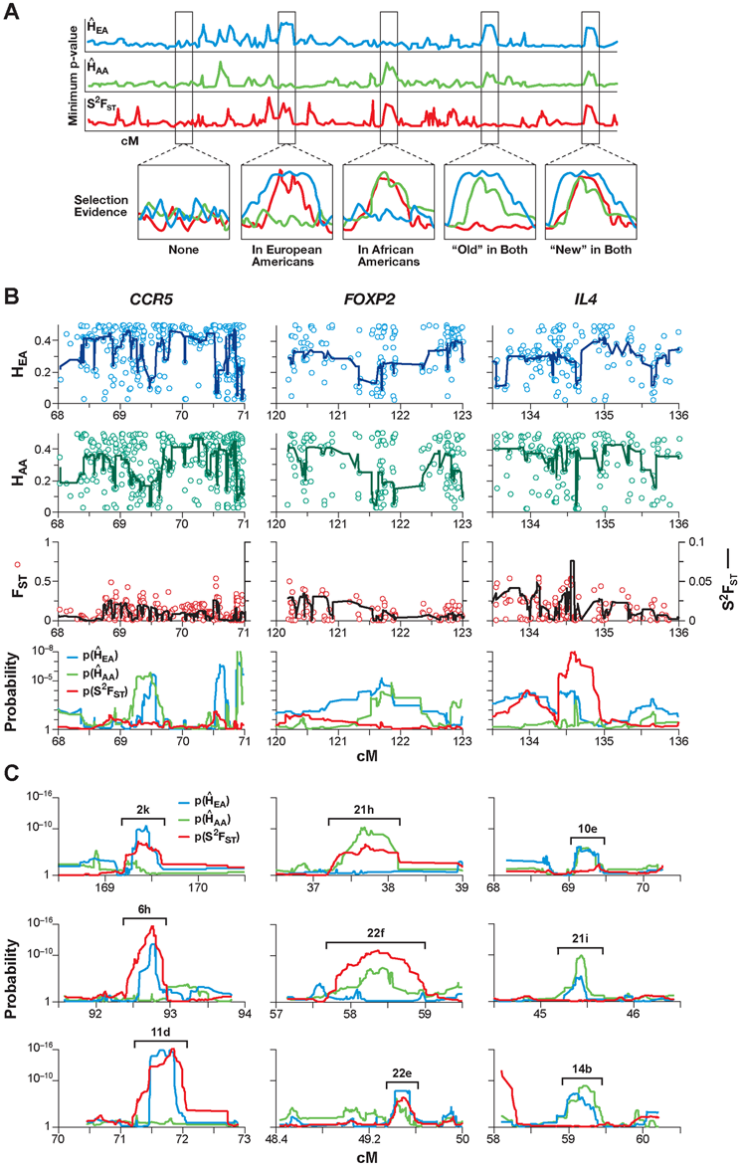
(A) Evidence and types of selection: (Top) Schematic of the lowest mean value fractional rank value (λ) distributions for local heterozygosity in a sample of 45 European Americans λ(H̀_EA_), 45 African Americans λ(H̀_AA_), and variance of population divergence λ(S^2^F_ST_) plotted across a chromosome. (Bottom) Four kinds of putative selection sites where one of the two populations has either lower values of heterozygosity as well as a rise in S^2^F_ST_ values at the same location, or a decreased local heterozygosity in both populations (see Table 1). (B) Three examples of known selection regions. *CCR5* and *FOXP2* genes have low values of heterozygosity in both Europeans and Africans, implying putative selection in the ancestral population (“old”; see Figure 2A, bottom). The region around *IL-4* (which includes the *IL-13* gene) shows a putative selection signature, as indicated by a decrease in H̀_EA_ and H̀_AA_ and increased S^2^F_ST_. Values of H_EA_ (blue), H_AA_ (green), and F_ST _(red) are plotted individually with most significant medians (H̀_EA _and H̀_AA_), and variance of F_ST_ (S^2^F_ST_) across 31 sliding windows of size 5 to 65 loci. (Bottom) λ values derived from H̀_EA_, H̀_AA_, and S^2^F_ST_ based on the 5 to 65 loci sliding windows around *CCR5*, *FOXP2* and *IL4*. (C) Similar plots of nine examples from 180 putative selection sites discovered in the current study (all plotted in Figure S3).

The resampling-based approach compared values of population-based statistics such as heterozygosity and population divergence in a number of adjacent SNPs, to the distribution of values obtained by the resampling of the same number of SNPs from random locations along each chromosome. The strategy for selection detection involved the following six steps (see also Figure 1 and Materials and Methods): 1) regional levels of heterozygosity (H̀) and population divergence (S^2^F_ST_) were estimated by choosing adjacent SNPs at the beginning of a single chromosome and computing H̀_EA_, H̀_AA_ and S^2^F_ST _for each SNP in the two populations. 2) The SNP window was moved one SNP to the right and the same parameters were computed, and then again for the next group of five SNPs, until the results for 10,864 groups (for chromosome 1, see Table S2) were determined. 3) The same computation but using windows of 7, 9, 11 … 65 SNPs (i.e., a total of 31 SNP window sizes from 5 to 65 SNPs in length), was assessed across each chromosome. A baseline distribution to which these estimates are compared, was developed by choosing five (then 7, 9, 11 ... 65) random SNPs sampled 100,000 times across each chromosome. We analyzed odd numbers to center the sampling window on a SNP. The upper window size was dictated by limits in computational capabilities. 4) A distribution of empirical values was obtained and fractionally ranked relative to the randomly sampled expectations (Figure 1). This process was then repeated 100 times to reach a total 10 million values. By combining these values, a chromosome-wide distribution of the mean fractional ranks (0<r<1) of given values (H̀_EA_, H̀_AA_, or S^2^F_ST_) for each of the 31 SNP array replicates (of increasing SNP window size) was assessed (Figure 1C). 5) For each SNP, the lowest fractional rank mean value (λ) was chosen among the 31 windows reflecting the size of the region with the largest deviation from expectations (see Materials and Methods). The distribution of λ values across each chromosome provides a quantitative indication of the departure from the random expectation and is plotted across the length of each chromosome versus cM position of each SNP (Figure 2A).

### Identifying a selection target in a simulated dataset

**Figure 3:**
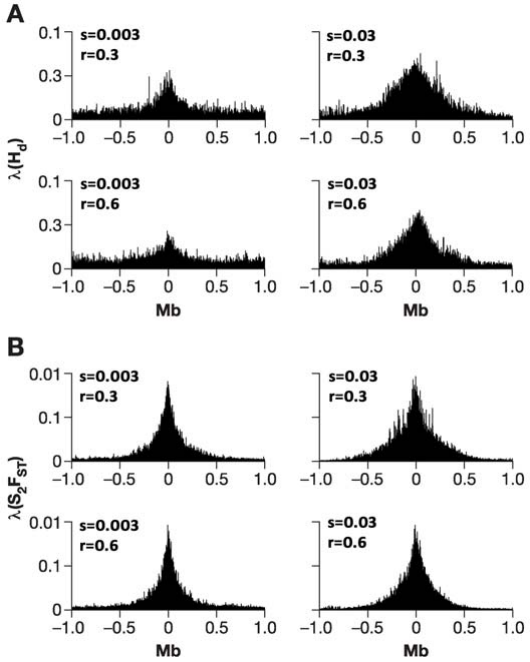
SelSim simulation results summarizing the effect of selection on the estimates of (A) λ(H̀_D_) and (B) λ(S^2^F_ST_) among 100 replicate of 2.61 Mb simulated chromosomes of chromosomes for each of the four parameter combination with varying selection coefficient and recombination rate. These simulations were performed using SelSim [24], assuming equal sampling (48 chromosomes) and a complete separation between the two populations. Selection parameter (s) ranged from neutral to 0.3% to 3% (left to right) and recombination intensity per site (r) from 0.3 to 0.6 (from the top down), assuming 10,000 effective population size in each of the two simulated populations (See Materials and Methods).

We addressed the validity of assumptions in our proposed computational approach by using coalescent simulations with a single selected site positioned on a simulated chromosome in SelSim [24]. If one population experienced selection at a mutant locus that arose in the ancestral population, a partial sweep represents the kind of selection we are interested in detecting. The assumptions we made were that neutral variation was present in the ancestral population before the selected mutation, and that the loci in the selected population carries a portion of the ancestral variation. We imitated genome-wide average mutation rate, the same population size as in our present study (24 individuals in each population), and a marker density to be close to that in our experimental dataset: one marker for each 10 kb. In turn, we varied selection strength, and intensity of recombination (see Materials and Methods). Values of λ were then calculated for the control and the selected population. In Figure 3, we summarize maximum λ values calculated for 100 replicate “chromosomes” in four different parameter combinations, alternating high (s = 0.03) and low (s = 0.003) selection coefficient versus high (r = 0.6) and low (r = 0.3) recombination rate. The choice of selection coefficients in the SelSim simulations was based on previously reported estimates. For example, selection coefficient for the lactase-persistence allele was predicted to be between 0.014 and 0.15 in CEPH, and between 0.09 and 0.19 in the Scandinavian population [25]. Furthermore, the selection coefficient has been set between 0.02 and 0.05 for *G6PD* deficiency which gives advantage to survival in the malarial regions [26].

Analysis of the simulation results show that a selective sweep results in a reduction of variation linked to the target of selection, and the extent of such reduction is dependent on the selection coefficient (s) (Figure 3). Coincidentally, strong recombination acts to break up long-range haplotypes which results in narrower signal. Overall, variance of F_ST_ across loci (S^2^F_ST_) yields more noteworthy λ values than those based on heterozygosity, and the location of the identified selected site was only 0.001 Mb for λ(S^2^F_ST_) and 0.005 Mb away for λ(H̀_D_) away from the target site on average. The overall less notable values of λ resulted from many fewer sites simulated on a single chromosome (261) rather than in our genome-wide scan (183,993). Reassuringly, a chromosome simulated under the neutral conditions (s = 0) does not demonstrate a characteristic pattern in heterozygosity or S^2^F_ST_ that indicates a selective sweep (not shown) but matches noise present beyond the selection sites (Figure 3).

### Evaluation of candidate regions

When a selective sweep occurs in the chromosomal region around a target gene in two populations that have recently separated, it produces signatures in more than one of the three estimates evaluated. Therefore, the interpretation of the derived data was based upon certain theoretical predictions (Table 1) validated by simulations (above). First, consider an “old” selection event that occurred prior to differentiation of modern European and African populations. Under this scenario, H̀_EA_ and H̀_AA_ are diminished but S^2^F_ST_ is diminished somewhat or remains at baseline expectations (Table 1, Figure 2A). For a “recent” selection event in one, but not the other population, the selected region displays lower H̀ values for that population and higher S^2^F_ST_ as different alleles approach fixation in the two populations (Table 1, Figure 2A). Thus, S^2^F_ST_ uniquely captures alterations of high and low F_ST_ values expected in the area of the selective sweep. In Figure 2B, three chromosomal regions (containing *CCR5*, *FOXP2* and *IL4*) illustrate these effects. An “old” selection event (lower levels of heterozygosity in two populations) likely occurred in the region that included *CCR5* (and several other chemokine receptors, notably *CCR1*) and *FOXP2*, while the *IL4* region has both decreased heterozygosity and increased S^2^F_ST_ (Figure 2B). The region of increased homozygosity is broad in African Americans (implying a recent selection event) but narrow in Europeans (perhaps indicating an older selective event, reduced in size by more generations of recombination). To further pilot our approach, a screen for natural selection imprints on the genomic neighborhood of 18 genes previously reported as objects of historic section validated *IL4* (Figure 2C), plus seven more (*IL13*, *ALDH2*, *SIGLEL1*, *SIGLEL9*, *FOXP2*, *CCR5*, and *AGT*) as demonstrating “recent” or “old” selection patterns (Table S1, Notes S1, and Supplemental References S1). The 13 genes where selection was reported only in the human lineage (Table S1) displayed significantly lower λ of H̀_EA _than a 10-times larger sample of randomly selected genes from the NCBI list (GLM, d.f. = 1, F = 8.76, p = 0.004). We did not see this difference for λ of either H̀_AA_ or S^2^F_ST_ (p = 0.97 and 0.12 respectively).

**Table 1:** Expected effects of different types of selection sweeps on regional levels of heterozygosity (H̀) and F_ST_ variance (S^2^F_ST_) in the chromosome neighborhood of a selected locus in two populations (African Americans [AA] and European Americans [EA]).

Positive Selection Type	H̀_AA_	H̀_EA_	S^2^F_ST_
Ancestral (old)	decrease	decrease	same or decrease†
Recent (European)	same	decrease	increase
Recent (African)	decrease	same	increase
Recent (both populations)	decrease	decrease	increase

† Expectation for S^2^F_ST_ from ancestral selection is dependent on its direction and magnitude, so this criterion was not evaluated in our analysis.

Ancestral selection is assumed to occur before the two populations separated, while recent selection is assumed in one or both isolated populations.

### Scanning the genome for selected regions

**Figure 4:**
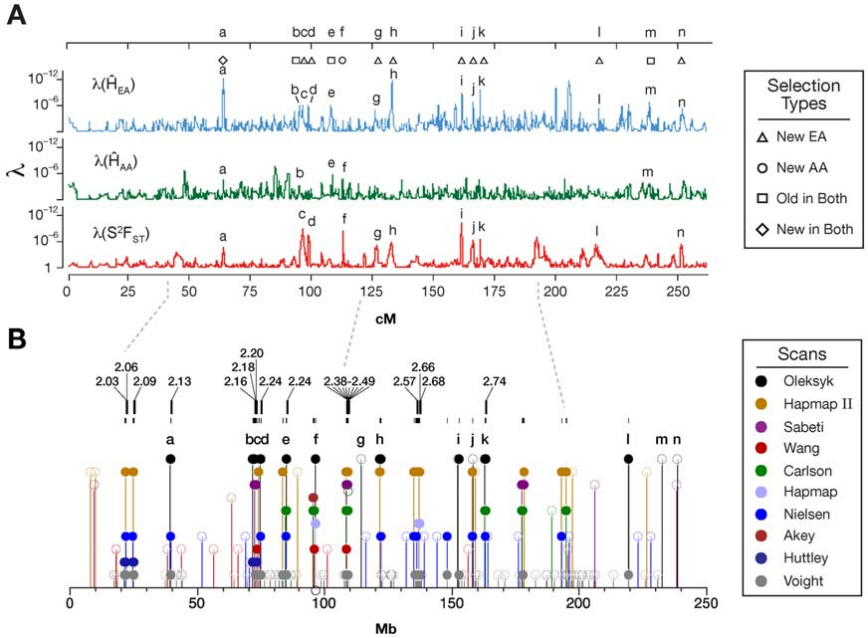
Scans for selected regions on chromosome 2 from this work and others [4], [9], [18], [20], [22], [23]. (A) Our scan for putative selection regions. A putative selected site is identified as the locus of a peak in two of three tests (λ(H̀_EA_), λ(H̀_AA_), or λ(S^2^F_ST_)) overlapped (as in Figure 2A) with ancestral selection (H̀_EA_ and H̀_AA_, squares), along with recent selection in Europeans (H̀_EA_ and S^2^F_ST_, triangles) and Africans (H̀_AA_ and S^2^F_ST_, circles; see Figures 5 and 6 for a full genome scan). Locations, genes, and evidence strength (λ) for the putative selection peaks are in Table S3. (B) Locations of regions implicated as selected by our study compared to nine other recent genome scans for selection [4], [9], [17], [18], [20]–[23]. Solid figures indicate that selected regions detected in at least two of these studies are overlapping (cross-validated). Open symbols indicate that the peaks do not overlap. Our full genome scan (Figures 5 and 6) cross-validated 356 regions whose locations are listed in Table S4. Dotted lines indicate cross-verified for which evidence of a signal is apparent in regions from other studies, but did not pass our criteria for inclusion (See Materials and Methods and Figures 7 and 8).

**Figure 5:**
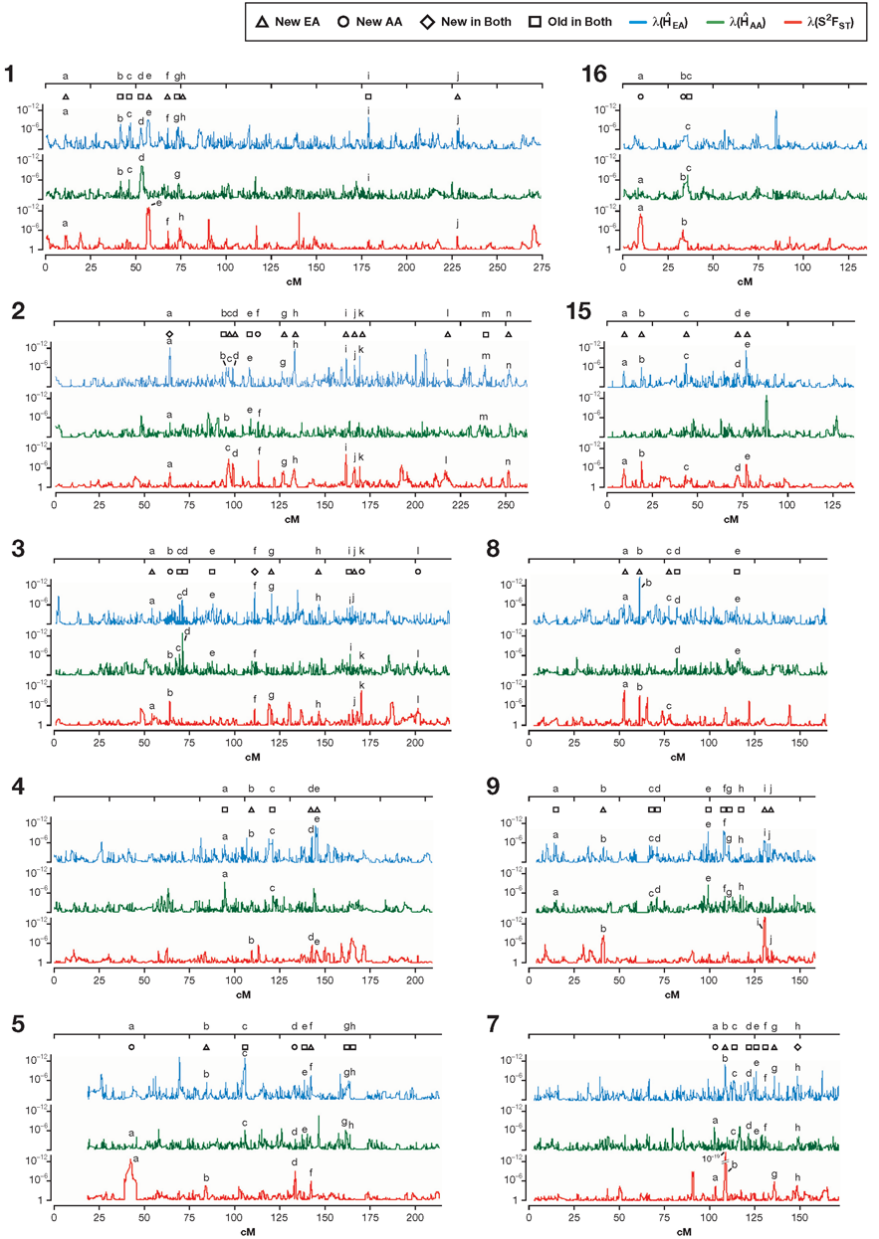
Scans for putative selection regions in the autosomes and X chromosome. Squares indicate the regions of the ancestral (old) selection, triangles indicate recent selection in Europeans, circles in Africans, and diamonds designate the new selection in both of these populations. A putative selected site is identified where two of the peaks overlap (as in Figure 1A). Both peaks are identified with a consecutive letter. Locations, genes, and evidence strength (λ) for the putative selection peaks are listed in Table S3. For clarity, the figure shows half of the genome: chromosomes 1–5, 7–9, 15 and 16.

**Figure 6:**
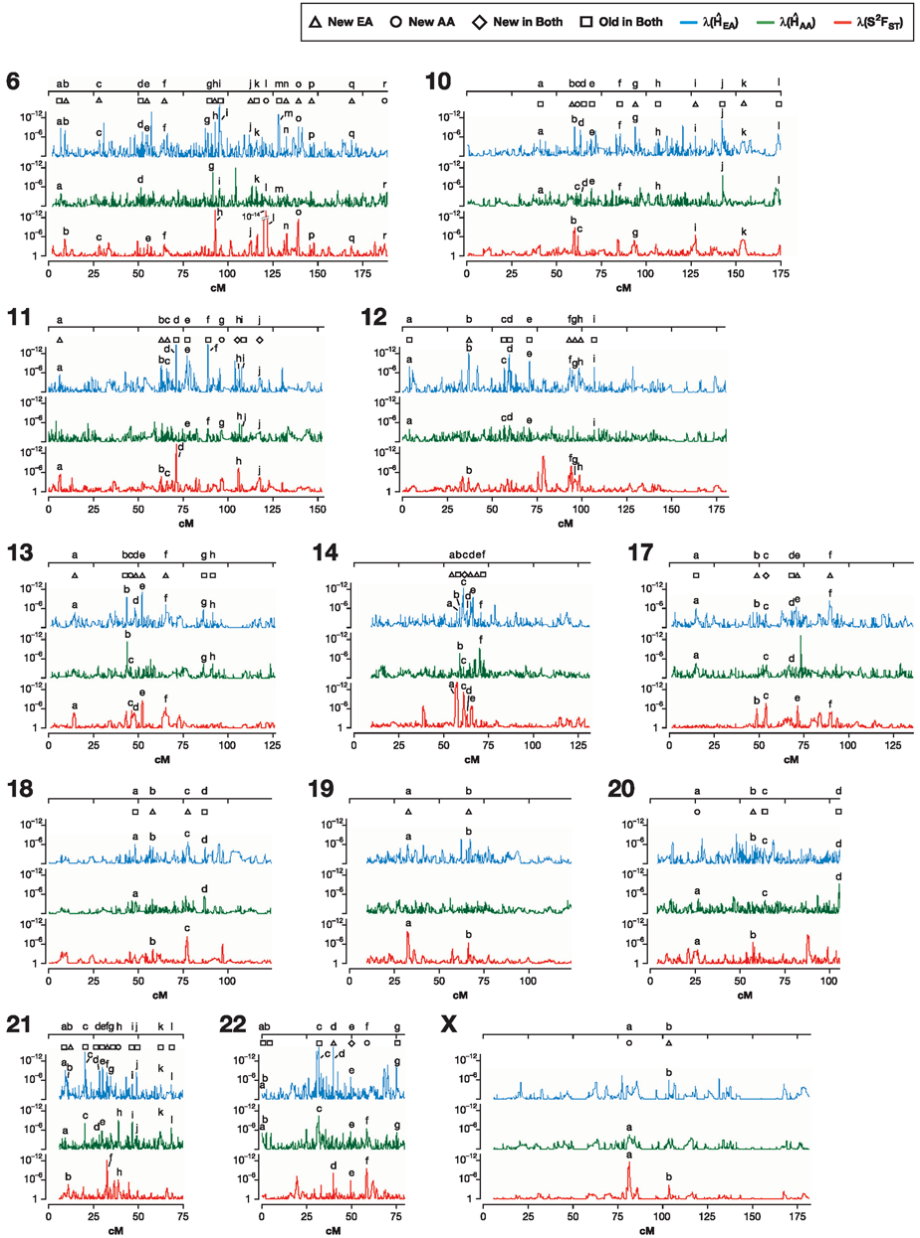
Scans for putative selection regions in the autosomes and X chromosome. Squares indicate the regions of the ancestral (old) selection, triangles indicate recent selection in Europeans, circles in Africans, and diamonds designate the new selection in both of these populations. A putative selected site is identified where two of the peaks overlap (as in Figure 1A). Both peaks are identified with a consecutive letter. Locations, genes, and evidence strength (λ) for the putative selection peaks are listed in Table S3. For clarity, the figure shows half of the genome: chromosomes 6, 10–14, 17–22 and chromosome X.

Regions with selection signatures were discovered from a scan across the genome for both ancestral and recent signatures of selection (Table 1). A challenge was to identify regions within the extreme 5% of observations that satisfy defined criteria. For each λ distribution, the upper 95% quantiles were: λ(H̀_EA_) = 3.8×10^−5^, λ(H̀_AA_) = 6.4×10^−4^, and λ(S^2^F_ST_) = 4.6×10^−4^. Overlapping locations with λ values lower than these cutoffs were classified as candidate regions for positive selection (Figure 2A). Suspected sites were inspected to pinpoint locations of genes and to estimate sizes of gene neighborhoods that show selection signatures. Analogous to Figure 4A, Figures 5 and 6 present plots for all 22 human autosomes and chromosome X, implicating 180 regions. Overall, 18 regions in African Americans and 77 in European Americans and 8 regions in both populations had strong evidence of recent selection, while ancestral selection was seen at 77 genomic regions, as illustrated schematically in Figure 1D (bottom) and shown in Figures 4A, 5 and 6. Each of these regions is also individually represented in Figure S1 (see Notes S2).

A complete list of the 180 selected regions, their locations, Kb length, range of extent, λ for H̀_AA_ and H̀_EA_ and S^2^F_ST_, and genes located within these regions are listed in Table S3. Some regions of the genome are devoid of any evidence of historic selection (0–50 cM of chromosomes 2 and 10), while others have multiple sites of selection (25–75 cM of chromosome 1; 55–90 cM chromosome 3). We expected old selection sites to be relatively smaller than the new ones, due to the long-term effects of recombination. Indeed, older sites were 77 Kb on average (95% C.I. = 4–831 Kb), almost 60% smaller than the recent sites (147 Kb, 95% C.I. = 6–1,349 Kb) (GLM, d.f. = 1, F = 5.59, = 2.364, p = 0.019). A comparison based on the cM distance yielded similar results (p = 0.001). Figure 4A shows a full scan for chromosome 2 where sites of selection in European Americans only, selection in African Americans only, “old” selection in both populations, and “new” selection in both populations (analogous to Figure 2A) are indicated by different symbols. Similarly, a complete scan of all the autosomes and chromosome X in Figure 5 displays locations of the overlapping peaks (designated by letters) indicating the putative selection sites (designated by symbols).

The human genome also has large variations in diversity on a micro and macro chromosomal scale [18], and recombination “hot spots” are an important source shaping linked genetic variation [18]. However, recent work has shown that most of the effect of recombination on diversity is on a relatively small scale (2–4 Kb, around the size of recombination hotspots) [17], [27]. Still, we accounted for linkage disequilibrium (LD) by using recombination-based distances (cM) interpolated from localized curvilinear regressions using deCODE genetic distances [28]. Since our study examined the distribution of allelic frequencies on a larger scale (markers were spaced about every 10 Kb in genes) and the regions found are much larger (average size = 107 Kb, 95% C.I. = 5–977, from a lognormal distribution), recombination cold spots are unlikely to explain the regions we have discovered. In our own simulation study, higher recombination rates result in a narrower footprint of selection (see: Identifying a selection target in a simulated dataset).

Indeed, simulation studies have been undertaken to test specificity and sensitivity of empirical approaches to detect signatures of recent selection from genome-wide polymorphism data similar to that in our study [29]. Analysis of these models indicated the possibility of discovery of some candidate genes, especially those subject to high selection pressures. At the same time, it is likely that empirical approaches could miss large numbers of loci, especially those displaying small to moderate selection effects [29], [30]. Additionally, strong confounding effects of demographic history complicate deduction of selection sites, especially when a small number of loci are studied [30]. However, these effects affect all loci in the genome indiscriminately, while natural selection is locus-specific [3], [31]. Consequently, while sampling large genomic SNP databases, empirical distributions can be constructed and genes subjected to the local forces, such as selection, as opposed to the genome-wide forces, like demography, which can be identified by the outlier approach [30], including the one used in our study and others (e.g. Voight et al. [19], and Wang et al. [20]). Finally, ascertainment bias is omnipresent in large-scale polymorphism studies because SNPs are routinely discovered on a limited number of chromosomes before being genotyped in a population study suitable for a selection scan. Ongoing efforts aim to avoid or minimize ascertainment bias, so hopefully, future datasets constructed for local human populations or other species will be less biased. Analyses of those datasets with modest ascertainment bias can dependably infer selected regions, resulting in an enhanced set of genes targeted by positive selection [30]. In addition, results from these scans, including ours, can be further validated. While many studies considered one statistic at a time, some of the recent studies [17], [21] considered looking at combinations of different approaches. While it is not obvious how to rank the input of each statistic, combinatorial approaches may be instrumental in decreasing error rates. This is why in our study, we first looked at different statistics according to our own expectation model (Table 1) and then compared coordinates of our putative regions to the coordinates of the regions found in other selection scans, hoping that they would provide us with further independent validation of these findings.

The identified regions and their interpretation are subject to important limitations. First, the power of our analysis is limited by the number of samples used (45 for each population) and the number of loci examined. Increased sample sizes would have resulted in better estimates, especially for the variance of population differentiation (S^2^F_ST_). Second, African American samples came from populations that are known to be admixed [32], [33]. Using a discrete, non-mixed African population might have implicated more and different regions or missed others. Third, the choice of the SNPs was not random, but rather comprises a source of commercially available TaqMan® assays for variable SNPs targeted for disease gene discovery [34] (see Materials and Methods). However, we expected that favored alleles would generally be located within extended and shared haplotypes [13], [27] that encompass larger regions, partially compensating for the gene-centric limitations of the dataset. Finally, our dataset is smaller than several other human ones. However, a strength of our study is its analysis of an independent dataset, especially since the recent literature largely focuses on either HapMap [2], [17]–[19], [21], [23] or Perlegen [20], [22] data in a series of overlapping studies [2].

### A synthesis of scans for selection across the genome

**Figure 7:**
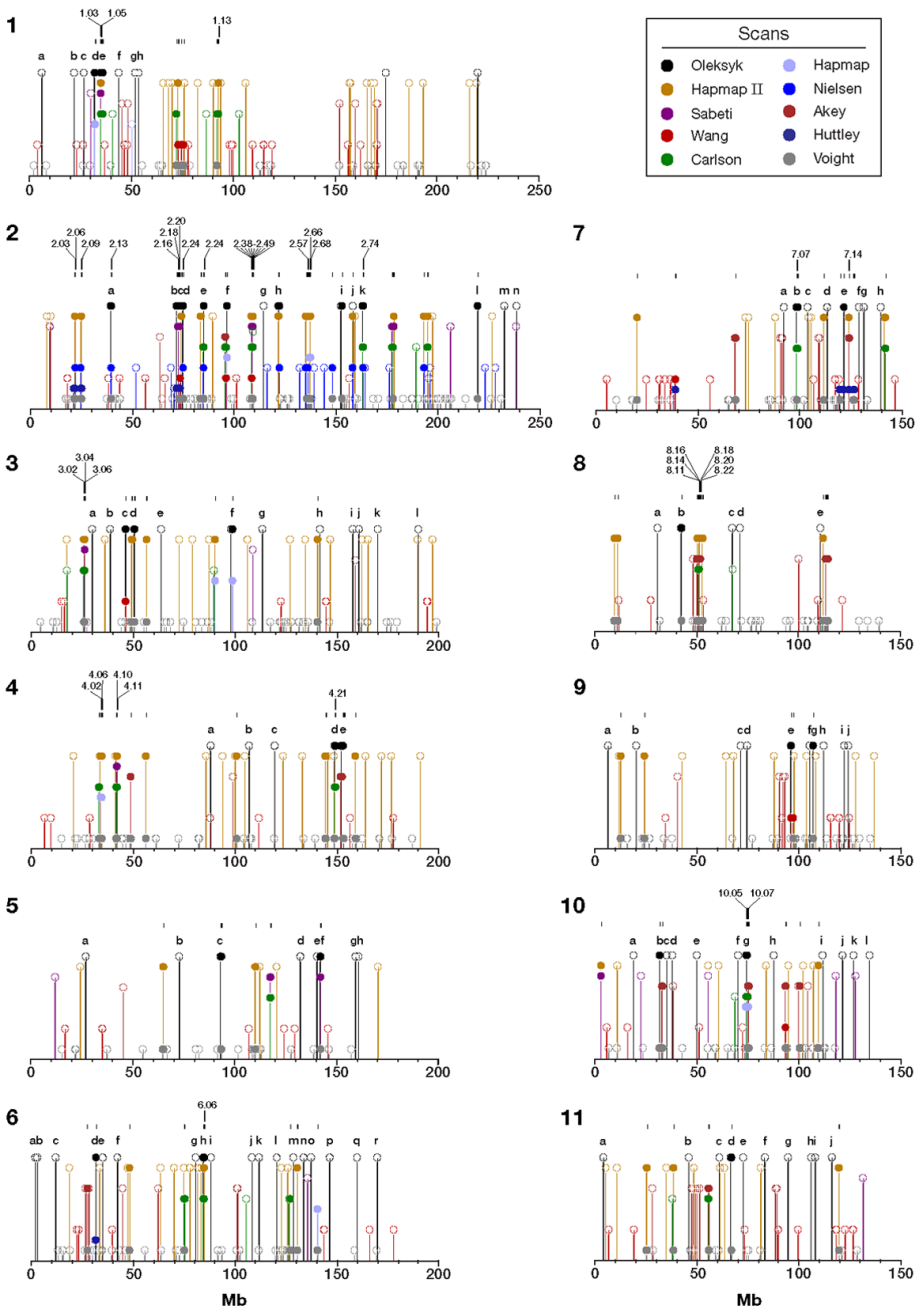
Locations of regions implicated as selected by our study compared to six other recent genome scans for selection [4], [9], [17]–[23]. Solid figures indicate that selected regions detected in at least two of these studies are overlapping. Open symbols indicate that the peaks do not overlap. Locations of regions included in the regions cross-validated by more than one study are listed in Table S4. Sequential numbers and locations of all the regions in this comparison are listed in Table S5. For clarity, the figure shows half of the genome: chromosomes 1–11.

**Figure 8:**
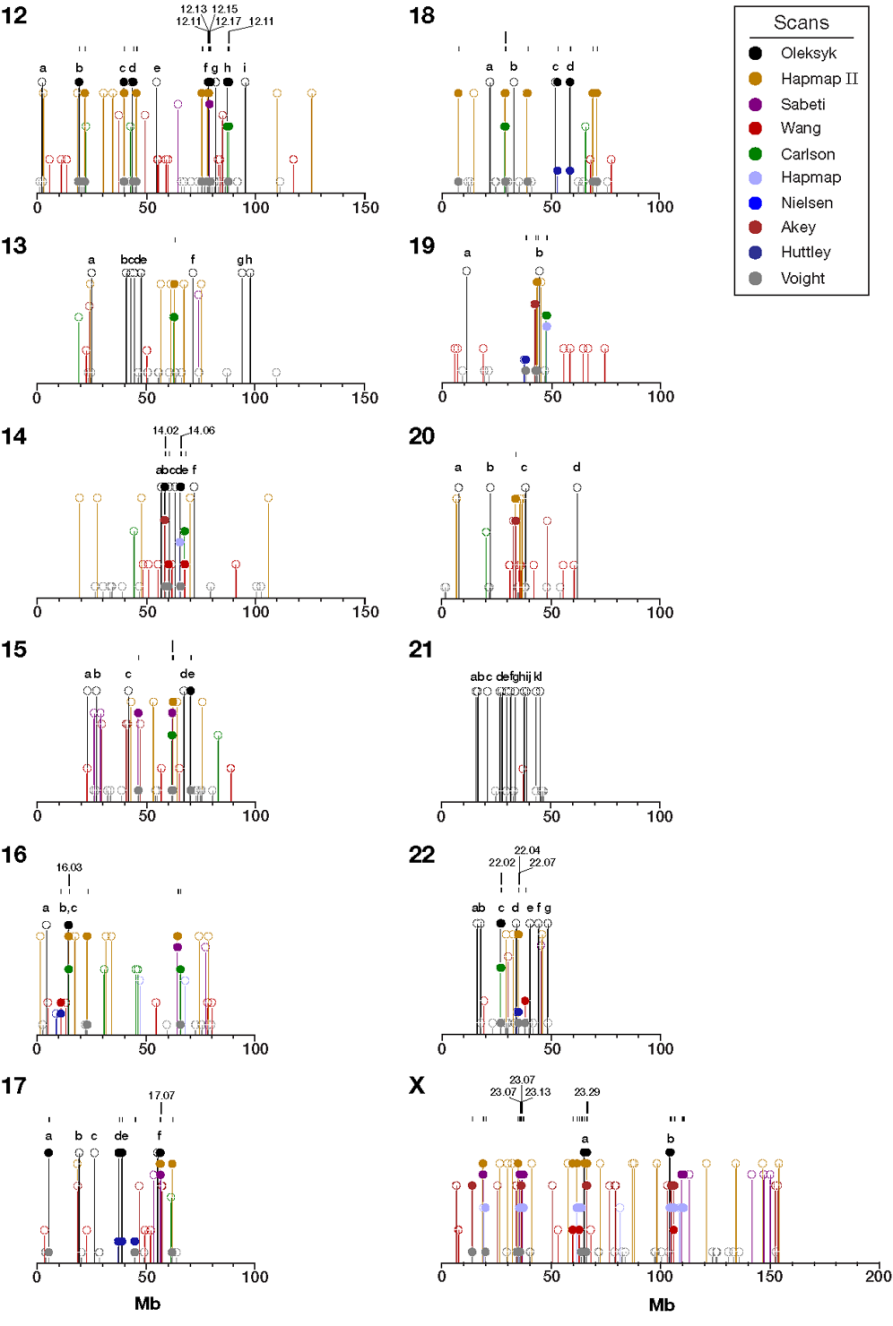
Locations of regions implicated as selected by our study compared to six other recent genome scans for selection [4], [9], [17]–[23]. Solid figures indicate that selected regions detected in at least two of these studies are overlapping. Open symbols indicate that the peaks do not overlap. Locations of regions included in the regions cross-validated by more than one study are listed in Table S4. Sequential numbers and locations of all the regions in this comparison are listed in Table S5. For clarity, the figure shows half of the genome: chromosomes 12–23, and chromosome X.

Including this one, ten studies have scanned the human genome for regions of selective sweeps to date, with varying levels of success by utilizing extended linkage disequilibrium, elevated divergence and reduced homozygosity [4], [9], [17]–[23]. Taken together, those efforts have identified 1,599 regions and sites of putative selection (Tables S3 and S4). We have compared the overlap of these studies to see how frequently the implicated sites were replicated by different approaches, populations, or both (Figures 4B, 7 and 8). To illustrate our comparative findings, consider first chromosome 2 for which there are data from eight studies, including ours (Figure 4B).

On chromosome 2, 81 regions are replicated by two or more studies. Analysis by Voight et al. [19] found 35 of these selected regions, and the analysis by Nielsen et al. [23] and the second generation HapMap [17] each found 12. Our analysis found 10, followed by Carlson et al. [22] with seven; the remaining five studies [4], [9], [18], [20], [21] each validated just one to three sites (Table S5). However, some of these studies used the same, or very similar approaches [17], [19] and/or similar data (different versions of the HapMap) [17], [18]. As a result, we would expect that many of the sites would be cross-validated by these studies, but these comparisons are simply not independent. One way to look past the correlation between non-independent studies is to count those regions cross-validated by three or more studies. Among these, 23 (26%) selected regions are validated by three or more methods on chromosome 2. As an independent approach with a different set of data, our study cross-validated very well: 6 out of 10 studies (60%) were still on the list, while other studies with more than 5 sites originally replicated by other studies had smaller fractions validated in this fashion: to 42% [17], [23], and 34% [19]. Carlson et al. [22] had a similar success (four out of seven, or 57%).

Interestingly, five out of seven sites with the strongest signals of positive selection reported by Nielsen et al. [23] on chromosome 2 were independently validated by our scan. Since Nielsen et al. [23] only evaluated one chromosome, we would expect successful cross-validation of many of the 180 sites we found throughout the rest of the genome, but would require similar (ongoing) comprehensive scans. Considering the genome as a whole and some limitations of these studies, extrapolation of our success on chromosome 2 genome-wide would suggest that many hundreds of sites will show multiple lines of evidence of selective sweeps. Note that we report exclusive coordinates for the validated regions. For example, in Figure 2, our overlapping regions 2.16 and 2.18 included two regions from this study (2b and 2c) that are encompassed by a combined region formed by an overlap of sites reported in Huttley et al. [9] and Sabeti et al. [21] (Table S4), while the same 3Mb-long extensive site reported in Huttley at al. [9] is replicated by five different studies, and results is listed in eight exclusive validated overlapping regions (2.15–2.22)(Table S4). Also on chromosome 2, two other cross-verified regions that were not seen in our scan but by the overlapping results of two other studies (peaks 2.1 [43.89–48.25 cM] and 2.6 [121.08–121.81 cM]) show peaks in one of our three tests (connected by a dotted line on Figure 2B); however, their values lie below our detection thresholds. There is also an indication of a peak on chromosome 2 that corresponds to a cluster of regions at 108.35–109.12 Mb (121.08–121.81 cM). Similar low level peaks are sometimes evident at additional regions validated by other studies, but were not significant in our scan (not shown).

Across the whole genome, a total of 356 regions were validated by at least two of the ten studies (Figures 7 and 8, Table S3, and Supplemental References S1). The study by Voight et al. [19] has the most validated regions (180 of 356). These, however, include 122 sites overlapped by 73 sites from another study that used the same algorithm [17]. Voight et al. [19] also has a relatively high number of locations reported (750), compared to all the others (10–180). Regions in our analysis are reported by other studies 47 times, while other studies are individually cross-validated in from 14 to 34 regions (Figures 7 and 8, Table S4). These cross-validated, or “golden” regions are particularly interesting since they were independently verified using a combination of different methods and different databases, and are probably less likely to include false positives.

Our analysis targeted sites surrounded by a local decrease in genetic variation and alternations of allele frequencies between the two populations examined, or so-called “selective sweeps.” These patterns are caused primarily by positive selection, so our scan missed other types of selection. Drift, bottlenecks, and recombination could also have created effects similar to those produced by selective sweeps. For example, low heterozygosity in Europeans could be accounted for by the bottleneck of the out-of-Africa dispersal event [35]. We adjusted our λ cutoffs genome-wide for each of λ(H̀_AA_) and λ(H̀_EA_) and λ(S^2^F_ST_) when identifying peaks with more stringent criteria for European American heterozygosity (see Materials and Methods). In addition, regions were identified as selected when at least two estimates showed unusually low λ, partially accounting for the demographic events in specific populations.

### The future of scanning genomes for selection

In addition and perhaps most importantly, the present study captures the signals of LD but does not require a family-based HapMap strategy and its large investment cost to capture signals of LD, and does not require the estimation of the ancestral allele state. A smaller scan can be applied to search for selection by comparing populations adapted to different local environments or disease, while using current chip-based genotyping technologies where the costs are several orders of magnitude lower. In addition, genome studies that focus on species other than our own will only be available at high density some time in the future. Certainly, genomes of some model organisms like *Drosophila* will be studied very closely, and densely genotyped on a scale comparable to humans [36]. However, this will not be the case for many other diploid animals or plants, including those attracting significant scientific interest because of their conservation status or usefulness. Currently, genotyping technologies are capable of processing 100,000–1,000,000 SNP assays in custom scans, and obtaining frequency estimates on a scale that yields datasets similar to the one we examined [37]. Therefore, our analysis method will be applicable to the studies of selection in the populations of mammalian species nominated by NHGRI for whole genome sequence at 7x coverage, and where SNP collections are assembled (e.g., chimp, dog, elephant, armadillo, cat, horse, cow, rabbit, etc.). Generally, dense genotyping will not be available on the scale currently only available in humans for most species anytime soon.

We are still developing an understanding of the shifting adaptive landscapes [38] and forces that act on populations and alter individual genomes. There are irregular patterns of variation, other than natural selection, that can arise under the influence of the evolutionary forces. Harboring an unusual pattern of variation compared to the rest of the genome (or chromosome) is not a guarantee that the locus is selected, and vice versa. However, it is likely that genomic regions containing selected loci have unusual patterns of genetic variation. Using different methodologies and different datasets may help us to better understand these phenomena. Many of the effects of selection are likely more subtle than those we identified. Thus, the criteria and indices used for the purpose of this investigation are by no means exclusive or necessarily optimal. For example, while heterozygosity is a universal indicator of genetic variation, central allele frequency changes (∼0.35–0.65) yield little signal. Our focus on those sites approaching fixation likely found older and more complete selection events.

One of the main challenges in the genetics field is the identification of functional relevance genome-wide. Determining genomic regions with signatures of recent selection has application to the discovery of new disease genes and other human phenotypes. Incorporating allele frequency and population differentiation approaches with haplotype-based methods should prove to be a powerful tool in genome-wide identification of selected regions. Evaluation of various approaches and datasets in identifying selected regions is particularly important, because it provides an independent verification of regions found by a combination of different methodologies and databases. Understanding of imprints of historic selection/adaptation episodes written in genomes of humans and other species offers modest promise in interpreting modern and ancestral gene origins and modifications.

## Materials and Methods

### SNP genotypes

A total of 183,997 SNPs were identified from the TaqMan® Validated SNP Genotyping Assays (formerly known as the TaqMan® Assays-on-Demand SNP Genotyping products) [34]. An initial collection of over 4 million SNPs was narrowed down by selecting high-quality candidate SNPs, aiming for a gene-centric picket fence of 10 kb spacing. Assays were then designed, manufactured, and validated on up to 90 individual DNA samples from African Americans and European Americans (45 individual unrelated DNA samples per group)[34], [39]. The resulting TaqMan® Validated SNP Genotyping Assays have a minor allele frequency ≥5% in at least one population.

Of these, 156,287 SNPs satisfied criteria for sample size and variability (n>14 people in both populations, and heterozygosity [H]>0 in at least one population). The distribution of heterozygosity was skewed towards the larger values in both populations as expected because SNPs were intentionally selected for genotyping with a minor allele frequency (MAF) >0.05 or other strong evidence of their polymorphic status. Most loci were highly polymorphic; 68.53% of loci in African Americans and 63.79% of loci in European Americans had H>45%, while only in 6.83% of loci in African Americans and 8.55% of all loci the levels of heterozygosity were below 5%. At the same time, overall average heterozygosity is higher in European Americans (0.34) than in African Americans (0.33). Chromosome X shows the highest levels of F_ST_ (0.201) and is almost twice as differentiated as the autosomes (0.125). There was a significant difference in the distribution of H_AA_, H_EA_, or F_ST_ among the chromosomes overall, as well as among the autosomes in an analysis of variance (p<0.0001, Proc GLM, SAS 9.1). While some of this significance can be attributed to the known ascertainment bias, the difference remains significant when accounting for the density of the SNPs assayed on different chromosomes (p<0.0001).

### Genome scan for selection, analytic methods

A database of allelic frequencies, number of samples genotyped, and the physical positions for each of 183,997 SNPs from European (n = 45) and African Americans (n = 45) developed by Applied Biosystems was evaluated. All individuals were evaluated on a single 96-well plate. SNPs chosen for validation in this dataset were likely to be the more common ones, since criteria of known frequencies or multiple lines of evidence were used to choose them for the assay development [34], [39]. Only those SNP allele frequency estimates with sample sizes over 15 or more individuals in each population were analyzed. Recombination-based distances (cM) were interpolated from a linear regression centered on the closest known marker and three neighboring markers flanking it on either side (cM distances were from an NCBI update [Build 35] of the combined linkage-physical map of the human genome [28]). In a few local regions (no more than one or two with errant data points per chromosome), the local regression was unable to reliably predict the position of the central marker. The errant data was dropped from analysis so that the predictive regressions were based on the remaining markers.

The expected heterozygosity for each locus in both European and African Americans was calculated as follows [40]:
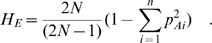



We computed the unbiased estimate of F_ST_ as described by Weir and Cockerham [41] and in a manner similar to Akey et al. [42]. For *i* populations (where *i = *1, 2, …, s), frequency of the allele A in the subpopulation *i* is denoted as *p*Ai and sample size in each population as n_i_. Given this, F_ST_ can be calculated in the following equation:
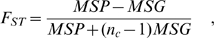
where MSG is the observed mean square error for loci within populations, MSP is the observed mean square errors between the populations, and *n_c_* is the average sample size across samples that also incorporate the variance in sample sizes over the populations [41]:
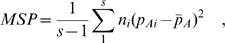


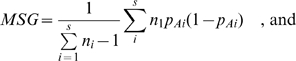


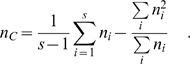



To calculate the distributions of genetic frequencies within and between the two populations, we sampled loci sequentially along each chromosome with the variable size frames in increasing size order, using only the odd numbers (Figure 1A top). The smallest frame included n = 5 data points, the next was n = 7, and the largest frame size included 65 points, resulting in 31 odd-numbered sizes of sliding frames. For each sliding frame of size N, moving median of expected heterozygosity across the sampling frame for each of the two human populations in this study (H̀_AA_ for African Americans and H̀_EA_ for European Americans), as well as a moving variance S^2^F_ST_ for the same region, was calculated using PROC EXPAND (SAS 9.1):
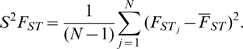



Distributions of H̀_AA_, H̀_EA_, and S^2^F_ST_, were estimated by resampling each chromosome for each of the 31 odd-number sizes from 5 to 65 (Figure 1A, bottom). The unrestricted random sampling (URS) option in PROC SURVEYSELECT (SAS 9.1) was used in the resampling process.

### Coalescent simulations

To address the conjecture of using S^2^F_ST_ as a tool to detect selection and the power of our computational approach, we used simulations with a single selected site positioned among 261 markers along a simulated 2.61 Mb-long chromosome. The model utilized marker density close to that in our experimental dataset: one marker for each 10 kb. We also assumed an average mutation rate of *μ* = 10^−8^ per site. The models varied in selection, strength, and intensity of recombination. These simulations were performed using SelSim [24], assuming equal sampling (48 chromosomes) and a complete separation between the two populations. Values of λ were calculated for the control or ancestral, and the selected or derived population. We calculated maximum λ values for 100 replicate “chromosomes” by the same resampling-based algorithm in four different parameter combinations, alternating high and low selection coefficients versus high and low recombination rates. Selection parameter (s) ranged from neutral to 0.3% to 3% and recombination intensity per site (r) from 0.3 to 0.6, assuming an 10,000 effective population size for each population (maximum allowed by the program).

The simulation scheme emulates one previously utilized to model population substructure of dog breeds where Pollinger et al. [43] showed significant heterozygosity and F_ST_ effects after a selective sweep. The SelSim model assumes the initial mutation to be rare [24], and may overestimate the selection signal if selection started on a mutation that reached significant frequency [43].Our study used SelSim for “proof of principle” purposes to demonstrate the patterns of our measurements: a decrease in heterozygosity, and particularly the increase in the S^2^F_ST_ measure we described. The exact strength and the extent of the selection signatures under different conditions are beyond the scope of the SelSim program and this report. In the simulation presently employed, 48 chromosomes carried the selected mutation (derived) and 48 chromosomes did not (ancestral), approximating the numbers of individuals we examined in each population. If one of the populations was recently separated from the other and selection acted on a mutation that arose in an ancestral population, the partial selective sweep is an approximation of the true process with the assumption that most of the neutral variation appeared before the introduction of the selected mutation. Therefore, the set of haplotypes that carry the selected mutation contains a subsample of neutral variation in the ancestral population, and can be contrasted with the set carrying the ancestral allele.

### Scanning the genome for selected regions

Each chromosome was sampled 100,000 times and fractional ranks, ranging from 0 to 1, determined for the sliding windows (described above; Figure 1B, C). The procedure was repeated 100 times, and fractional ranks for each SNP sliding window were averaged. Extreme values of fractional rank were predicted using curvilinear regression (with r^2^ consistently >0.99) to consistently extend estimates of λ below about 1×10^−7 ^attainable by fitting the observed data in the most extreme 2.5% SNP frames into their resampled distributions.

These results were summarized by determining the lowest mean value of the fractional ranks of the sliding windows centered on that SNP (λ) for each H̀_AA_, H̀_EA_, and S^2^F_ST_. The values of λ(H̀_AA_), λ(H̀_EA_), and λ(S^2^F_ST_) are evaluated relative to each other and are plotted against the cM position for each chromosome (illustrated in Figure 1D top). Diagrams of expected putative regions of recent selection in African Americans, European Americans, and in both populations, as well as of the ancestral selection, are illustrated for λ in Figure 1D (bottom).

## Supporting Information

Table S1 Lowest mean fractional rank values (λ) of H_AA_, H_EA_, and S^2^F_ST_ for regions implicated previously as selection targets. Only those genes indicating recent positive selection in humans or humans and primates are shown. Significant values are underlined.
(0.10 MB DOC)

Notes S1 Table S1 Notes
(0.03 MB DOC)

Table S2 Percentages of the SNPs and portion of chromosomes sampled
(0.05 MB DOC)

Table S3 Locations of putative selected sites, range of their extent, and λ for H̀_AA_ and H̀_AA_ and S^2^F_ST_, as well as the genes located within these regions.
(0.36 MB DOC)

Table S4 Locations of the cross-validated regions.
(0.95 MB DOC)

Table S5 Locations of all the discovered regions and sites used in this scan and other studies
(3.00 MB DOC)

Figure S1 Individual graphs of 180 putative selection regions. Peak numbers correspond to Table S3 where the lowest mean rank value (λ), locations, and genes included in the putative selection regions are also shown. The vertical scale corresponds to the negative logarithm of the λ(H_AA_) (green line), λ(H_EA_) (blue line), and λ(S^2^F_ST_) (red line). A putative selected site is identified where two of the peaks overlap (as in Figure 2A, bottom). The horizontal scale indicates location in cM. The locations of SNPs are represented by the black hash marks on the top of the graph. The extent of genes is represented by the horizontal blue line. Chromosome numbers (Chrom), location in cM, the most significant window size (out of 30 possible, windowsize), type of selection (SelcType), name of the closest gene (Gene), and the distance to it from the central location in cM (Dist) are all listed in the heading above each graph. The circle in the middle indicates the central location of the putative region. The range of the selected region is indicated by the two black vertical lines. The names of the genes included in the selected regions are listed from left to right in Table S3.
(1.29 MB PDF)

Notes S2 Figure S1 Notes
(0.02 MB DOC)

Supplemental References S1 (0.03 MB DOC)
