# Supplementary material for: Identifying Selected Regions from Heterozygosity and Divergence Using a Light-Coverage Genomic Dataset from Two Human Populations
Source: PLoS One. 2008 Mar 5;3(3):e1712. doi: 10.1371/journal.pone.0001712 (PMC2248624; doi:10.1371/journal.pone.0001712)
Supplement: Table S1 — Lowest mean fractional rank values (λ) of HAA, HEA, and S2FST for regions implicated previously as selection targets. Only those genes indicating recent positive selection in humans or humans and primates are shown. Significant values are underlined. (0.10 MB DOC) [file pone.0001712.s001.doc]

Table S1. Lowest mean fractional rank values (λ)^a^ of Ĥ_AA_, Ĥ_EA_, and S^2^F_ST_ for regions implicated previously as selection targets. Only those genes indicating recent positive selection in humans or humans and primates are shown. Significant values are underlined.

| **Gene Name** | **Chromo some** | **Position**  **(bp)** | **Position**  **(cM)** | **λ(**Ĥ**_EA_)^a^** | **λ(**Ĥ**_AA_)** | **λ(S^2^F_ST_)** | **Evidence^b^** | **Organism** | **Function** | **Reference** |
| --- | --- | --- | --- | --- | --- | --- | --- | --- | --- | --- |
|
| ***IL13*** | 5 | 132,023,232 ± 1,468 | 134.584 ± 0.001 | 4.4 x 10^-5^ | .42 | 1.4 x 10^-9^ | ● | Humans | host-pathogen interaction | [1] |
| ***IL4*** | 5 | 132,041,770 ± 4,498 | 134.596 ± 0.003 | 4.4 x 10^-5^ | .47 | 2.0 x 10^-9^ | ● | Humans | host-pathogen interaction | [1] |
| ***ALDH2*** | 12 | 110,688,785 ± 21,719 | 127.278 ± 0.027 | .01 | .25 | .003 | ● | Humans | diet | [2] |
| ***SIGLEC9*** | 19 | 56,322,678 ± 2,701 | 86.391 ± 0.012 | .005 | .61 | .05 | ● | Humans | host-pathogen interaction | [3] |
| ***FOXP2*** | 7 | 113,786,667 ± 137,440 | 121.672 ± 0.073 | 9.8 x 10^-7^ | 4.9 x 10^-5^ | .14 | ● | Humans | behavior | [4] |
| ***CCR5^c^*** | 3 | 46,389,666 ± 3,029 | 69.531 ± 0.002 | 4.4 x 10^-7^ | 4.7 x 10^-6^ | .15 | ● | Humans | host-pathogen interaction | [5] |
| ***SIGLECL1*** | 19 | 56,691,640 ± 5,215 | 87.816 ± 0.022 | .005 | .01 | .17 | ● | Humans | host-pathogen interaction | [6] |
| ***AGT*** | 1 | 227,150,811 ± 5,791 | 234.200 ± 0.008 | .009 | .04 | .24 | ● | Humans | miscellaneous | [7] |
| ***G6PD^d^*** | 23 | 153,331,242 ± 7,932 | 180.418 ± 0.017 | 3.4 x 10^-4^ | .59 | .06 | ○ | Humans | host-pathogen interaction | [8] |
| ***LCT*** | 2 | 136,403,815 ± 24,668 | 149.030 ± 0.022 | .003 | .13 | .10 | ○ | Humans | diet | [9] |
| ***ASPM*** | 1 | 193,816,168 ± 31,057 | 194.349 ± 0.013 | 5.0 x 10^-5^ | .57 | .25 | ○ | Humans | brain anatomy | [10] |
| ***CD59*** | 11 | 33,697,866 ± 16,734 | 49.265 ± 0.020 | .37 | .02 | .34 | ○ | Humans/Catharines | host-pathogen interaction | [11] |
| ***BRCA1*** | 17 | 38,490,389 ± 40,545 | 71.511 ± 0.030 | .10 | .16 | 4.7 x 10^-7^ | ○ | Humans/Primates | miscellaneous | [12] |
| ***COX8A*** | 11 | 63,499,623 ± 968 | 68.908 ± 0.001 | .09 | .13 | .03 | ○ | Humans/Primates | miscellaneous | [11] |
| ***LYZ*** | 12 | 68,031,356 ± 2,925 | 84.364 ± 0.003 | .56 | .005 | .06 | ○ | Humans/Primates | diet | [13] |
| ***FY*** | 1 | 155,988,473 ± 890 | 154.127 ± 0.002 | .29 | .06 | .16 | - | Humans | host-pathogen interaction | [14,15] |
| ***TNFSF5*** | 23 | 135,461,979 ± 6,090 | 139.324 ± 0.007 | .81 | .33 | .55 | - | Humans | host-pathogen interaction | [16] |
| ***COX4I1*** | 16 | 84,394,403 ± 3,706 | 120.542 ± 0.013 | .33 | .72 | .06 | - | Humans/Primates | miscellaneous | [17] |

^a^ λ values indicate the lowest mean fractional rank value that was chosen among the replicates reflecting a size of the region with the largest deviation from expectations within each gene. ^b^ Solid circle (●) indicates that at least two of the three λ values, and an open circle (○) indicates that at least one of the three λ values in the region surrounding the selected gene was located in the tail of the distribution for that chromosome (<.05). ^c^ Recent directional selection was originally postulated [5] following by a claim for balancing selection [18], but these claims have been recently disputed [19]. ^d^ Evidence for selection was expected in Africans, not Europeans as observed here [8].
