# Supplementary material for: Identifying Selected Regions from Heterozygosity and Divergence Using a Light-Coverage Genomic Dataset from Two Human Populations
Source: PLoS One. 2008 Mar 5;3(3):e1712. doi: 10.1371/journal.pone.0001712 (PMC2248624; doi:10.1371/journal.pone.0001712)
Supplement: Notes S1 — Table S1 Notes (0.03 MB DOC) [file pone.0001712.s002.doc]

**Table S1 Notes**

To better understand the pattern of signatures that natural selection imprints on the genomic neighborhood of its targets, we examined the distributions of λvalues around the genes where effects of positive selection were substantiated in the literature. When a “selective sweep” occurs in one or both populations after their separation, it should result in an increased variance of genetic divergence (S^2^F_ST_) with a simultaneous localized reduction of genetic variation (Ĥ_EA_ or Ĥ_AA_). We were able to observe one or both of these phenomena in most of the chromosomal regions known to harbor signatures of positive selection in human populations or between humans and primates. When considering those 18 genes where signatures of selection have been identified previously, we expected only one or two to deviate significantly from the average S^2^F_ST_ and heterozygosity levels by chance alone. However, *CCR5*, *FOXP2* (both in Figure 1), *SIGLECL1,* and *AGT* showed reduced heterozygosity in both European American and African American samples (old selection) while *IL4* (Figure 1B), *IL13, SIGLEC9* and *ALDH2* had evidence of reduced heterozygosity in European Americans, and elevated S^2^F_ST_ in the chromosomal regions around their locations (new EA selection). In addition, *G6PD,* *LCT* and *ASPM* had reduced heterozygosity in European Americans, and *CD59* and *LYZ* had decreased heterozygosity in African Americans. Finally, *BRCA1* and *COX8A* had strong evidence of high differentiation between the two populations (elevated S^2^F_ST_ values across these regions). The remaining genes (*FY*, *TNFSF5*, and *COX4I1*) showed no evidence of altered patterns of genetic variation that could have been missed either due to a lack of power, inability of this technique to detect balancing or purifying selection, admixture in African Americans, or because they are located in the regions poorly sampled with the variable SNPs.

Overall, given prior expectations, we were able to clearly validate seven genes with our scan criteria and see evidence of selection at seven more, while missing only three. Combined with our ability to see 10 of 14 cross-validated regions on chromosome 2 (the best characterized one, Fig. 2B), these analyses provide evidence in support of the majority of the 180 regions we have putatively identified as selected.
