# Supplementary material for: Identifying Selected Regions from Heterozygosity and Divergence Using a Light-Coverage Genomic Dataset from Two Human Populations
Source: PLoS One. 2008 Mar 5;3(3):e1712. doi: 10.1371/journal.pone.0001712 (PMC2248624; doi:10.1371/journal.pone.0001712)
Supplement: Table S2 — Percentages of the SNPs and portion of chromosomes sampled (0.05 MB DOC) [file pone.0001712.s003.doc]

Table S2. Percentages of the SNPs and portion of chromosomes sampled^a^

| Chromosome | Length  (cM) | Total # of SNPs | # SNPs excluded | % SNPs included | Extent of the excluded portion of the chromosome (cM) | Portion of Chromosome Covered (%)^b^ |
| --- | --- | --- | --- | --- | --- | --- |
| 1 | 272.30 | 10864 | 391 | 96.4 | 23.13 | 91.5 |
| 2 | 260.03 | 11056 | 354 | 96.8 | 18.14 | 93.0 |
| 3 | 221.78 | 10953 | 212 | 98.1 | 16.51 | 92.6 |
| 4 | 204.51 | 7346 | 0 | 100.0 | 0 | 100.0 |
| 5 | 212.41 | 7332 | 303 | 95.9 | 17.50 | 91.8 |
| 6 | 190.34 | 13414 | 0 | 100.0 | 0 | 100.0 |
| 7 | 191.12 | 8841 | 0 | 100.0 | 0 | 100.0 |
| 8 | 164.01 | 6443 | 0 | 100.0 | 0 | 100.0 |
| 9 | 156.61 | 6401 | 0 | 100.0 | 0 | 100.0 |
| 10 | 180.80 | 7247 | 0 | 100.0 | 0 | 100.0 |
| 11 | 153.36 | 7958 | 0 | 100.0 | 0 | 100.0 |
| 12 | 179.41 | 7665 | 0 | 100.0 | 0 | 100.0 |
| 13 | 129.65 | 4134 | 0 | 100.0 | 0 | 100.0 |
| 14 | 130.45 | 5009 | 0 | 100.0 | 0 | 100.0 |
| 15 | 134.28 | 4421 | 26 | 99.4 | 1.40 | 99.0 |
| 16 | 133.83 | 3765 | 0 | 100.0 | 0 | 100.0 |
| 17 | 137.31 | 4926 | 0 | 100.0 | 0 | 100.0 |
| 18 | 122.88 | 3376 | 0 | 100.0 | 0 | 100.0 |
| 19 | 120.74 | 4059 | 0 | 100.0 | 0 | 100.0 |
| 20 | 103.28 | 6579 | 0 | 100.0 | 0 | 100.0 |
| 21 | 71.80 | 5039 | 0 | 100.0 | 0 | 100.0 |
| 22 | 79.57 | 5907 | 0 | 100.0 | 0 | 100.0 |
| Average for autosomes | 161.38 | 6942.5 | 58.5 | 99.4 | 3.5 | 98.5 |
| X-chrom. | 179.62 | 3753.0 | 1145.0 | 69.5 | 91.5 | 49.1 |

^a^ The number of SNPs included was based upon the presence of summary statistics for at least 20 of the 31 sliding windows (Fig. 1A top).

^b^ Positions in the genome included in 20 out of 31 sliding windows.
