# Supplementary material for: Identifying Selected Regions from Heterozygosity and Divergence Using a Light-Coverage Genomic Dataset from Two Human Populations
Source: PLoS One. 2008 Mar 5;3(3):e1712. doi: 10.1371/journal.pone.0001712 (PMC2248624; doi:10.1371/journal.pone.0001712)
Supplement: Table S3 — Locations of putative selected sites, range of their extent, and λ for H̀AA and H̀AA and S2FST, as well as the genes located within these regions. (0.36 MB DOC) [file pone.0001712.s004.doc]

Table S3. Locations of putative selected sites, range of their extent, and λ^a^ for Ĥ_AA_ and Ĥ_EA_ and S^2^F_ST_, as well as the genes located within these regions

Chromosome 1

| **Peak Number** | **Peak Name** | **Selection type^b^** | **Location (bp)^c^** | **Range (bp)^d^** | **Location (cM)** | **Range (cM)** | **λ(Ĥ_EA_)** | **λ(Ĥ_AA_)** | **λ(S^2^F_ST_)** | **Genes Included^e^** |
| --- | --- | --- | --- | --- | --- | --- | --- | --- | --- | --- |
| 1 | **a** | new EA | 6,266,087 | 3,612 | 10.74 | 0.008 | 2.71x10^-5^ | 7.19x10^-1^ | 7.05x10^-5^ |  |
| 2 | **b** | old | 22,338,339 | 442,643 | 41.32 | 0.533 | 2.31x10^-8^ | 1.38x10^-4^ | 5.43x10^-1^ | *CDC42, WNT4, LOC343384, KIAA0478* |
| 3 | **c** | old | 26,913,547 | 13,615 | 46.00 | 0.012 | 2.69x10^-5^ | 9.81x10^-7^ | 3.71x10^-1^ | *FLJ12455* |
| 4 | **d** | old | 32,550,083 | 718,391 | 52.93 | 1.080 | 3.35x10^-7^ | 3.11x10^-10^ | 4.63x10^-1^ | *FLJ10315, KPNA6, DKFZp451J, MGC1203, FLJ10547, LOC441881, RP4^-6^22L5, EIF3S2, LOC339483, MGC10820, LCK, HDAC1, MLP, LOC400749, STK22C, FLJ10276, LOC343338, ZBTB8, ARCH, RBBP4, SYNCOILIN, KIAA1522* |
| 5 | **e** | new EA | 35,865,719 | 1,498,055 | 57.02 | 1.383 | 1.42x10^-9^ | 6.55x10^-1^ | 6.44x10^-14^ | *ZNF258, MGC14276, ZMYM1, SFPQ, ZNF262, PKD1-like, NCDN, AP2E, PSMB2, FLJ38984, CLSPN, EIF2C4, EIF2C1, EIF2C3, LOC388620, TEKT2, ADPRHL2, COL8A2, TRAPPC3, FLJ10350, THRAP3, FLJ22938, FLJ10647, MGC4796, LSM10, NOR1, MRPS15, CSF3R* |
| 6 | **f** | new EA | 44,069,897 | 85,311 | 67.79 | 0.071 | 2.84x10^-7^ | 6.29x10^-2^ | 2.63x10^-6^ | *SIAT6, ARTN, IPO13, DPH2L2, ATP6V0B* |
| 7 | **g** | old | 52,472,004 | 687,810 | 73.53 | 0.716 | 1.37x10^-7^ | 3.06x10^-5^ | 9.28x10^-4^ | *RAB3B, TLP19, MGC20419, MGC23908, LOC401951, ZFYVE9, KIAA1836, ORC1L, FLJ14936, ZCCHC11, GPX7, MGC52498* |
| 8 | **h** | new EA | 53,923,518 | 288,686 | 75.09 | 0.371 | 2.62x10^-6^ | 6.52x10^-1^ | 1.06x10^-4^ | *GLIS1, FLJ10407, DJ167A19.* |
| 9 | **i** | old | 175,150,026 | 40,553 | 179.41 | 0.025 | 2.01x10^-9^ | 6.32x10^-4^ | 2.49x10^-3^ |  |
| 10 | **j** | new EA | 220,496,717 | 427,574 | 228.37 | 0.330 | 1.59x10^-6^ | 6.53x10^-1^ | 2.35x10^-4^ | *TP53BP2, LOC440719, LOC440720, LOC440721, LOC441920, FBXO28, LOC388746, DEGS* |

^a^ λ values indicate the lowest mean fractional rank value that was chosen among the replicates, reflecting a size of the region with the largest deviation from expectations within each gene.

^b^ Selection types are either old, having predated the shared ancestral population of African and Europeans, or new in either Africans (new AA) or Europeans (new EA) or both (new in both).

^c^ Chromosome positions are from the UCSC human genome build 17, corresponding to NCBI build 35.

^d^ Range refers to the extent of the largest sampling frame with the lowest λ-s for this region. It indicates an area that encompasses the location of the selected region.

^e^ Genes included are from NCBI.

**Table S3 (cont.)** Chromosome 2

| **Peak Number** | **Peak Name** | **Selection type** | **Location (bp)** | **Range (bp)** | **Location (cM)** | **Range (cM)** | **λ(Ĥ_EA_)** | **λ(Ĥ_AA_)** | **λ(S^2^F_ST_)** | **Genes Included** |
| --- | --- | --- | --- | --- | --- | --- | --- | --- | --- | --- |
| 11 | **a** | new in both | 39,341,697 | 421,386 | 63.73 | 0.485 | 5.84x10^-13^ | 2.34x10^-5^ | 7.19x10^-5^ | *SOS1, LOC440857, CDKL4, MAP4K3* |
| 12 | **b** | old | 71,951,007 | 765,616 | 95.06 | 1.175 | 2.37x10^-5^ | 5.66x10^-4^ | 8.10x10^-1^ | *ZFML, DYSF, CYP26B1, SEC15L2* |
| 13 | **c** | new EA | 72,023,156 | 909,913 | 96.36 | 1.403 | 9.57X10^-7^ | 1.39x10^-1^ | 5.35x10^-9^ | *ZFML, DYSF, CYP26B1, SEC15L2* |
| 14 | **d** | new EA | 74,684,965 | 464,785 | 98.80 | 0.443 | 2.80x10^-6^ | 9.31x10^-2^ | 1.25x10^-7^ | *MOBK1B, MTHFD2, DCTN1, SLC4A5, LOC388963, LOC440869, FLJ12953, RTKN, HMGA1L4, WBP1, GCS1, MRPL53, FLJ14397, FLJ12788, LOC400962, LOC151534, NSPC1, TLX2, DQX1, AUP1, PRSS25, LOXL3, DOK1, LOC130951, SEMA4F* |
| 15 | **e** | old | 84,900,804 | 41,091 | 108.10 | 0.016 | 3.39x10^-6^ | 3.83x10^-4^ | 4.11x10^-2^ |  |
| 16 | **f** | new AA | 96,363,972 | 58,622 | 112.86 | 0.011 | 4.20x10^-1^ | 1.50x10^-4^ | 9.95x10^-5^ | *FLJ20507, CIAO1, U5^-20^0KD* |
| 17 | **g** | new EA | 114,364,004 | 120,585 | 126.21 | 0.095 | 1.97x10^-5^ | 9.86x10^-3^ | 1.48x10^-4^ | *ACTR3* |
| 18 | **h** | new EA | 121,770,798 | 493,154 | 133.29 | 0.747 | 1.65x10^-12^ | 9.96x10^-1^ | 1.90x10^-5^ | *TFCP2L1, CLASP1* |
| 19 | **i** | new EA | 152,484,853 | 464,192 | 161.94 | 0.386 | 1.11x10^-9^ | 7.31x10^-2^ | 5.21x10^-9^ | *NEB, ARL5, CACNB4* |
| 20 | **j** | new EA | 158,397,794 | 587,091 | 166.56 | 0.479 | 2.60x10^-7^ | 6.58x10^-1^ | 1.60x10^-6^ | *PSCDBP, ACVR1C, ACVR1, UPP2* |
| 21 | **k** | new EA | 163,109,385 | 507,084 | 169.45 | 0.246 | 2.52x10^-10^ | 3.17x10^-1^ | 1.48x10^-6^ | *FAP, IFIH1, GCA, KCNH7* |
| 22 | **l** | new EA | 219,312,501 | 6,230 | 218.30 | 0.014 | 5.55x10^-6^ | 6.20x10^-3^ | 2.80x10^-4^ | *RNF25* |
| 23 | **m** | old | 232,679,638 | 444,574 | 238.96 | 0.736 | 6.56x10^-7^ | 3.96x10^-4^ | 2.34x10^-1^ | *PDE6D, COPS7B, LOC391491, NPPC, MGC42174* |
| 24 | **n** | new EA | 238,452,816 | 47,462 | 252.53 | 0.123 | 4.65x10^-6^ | 2.09x10^-2^ | 1.10x10^-4^ | *LRRFIP1* |

**Table S3 (cont.)** Chromosome 3

| **Peak Number** | **Peak Name** | **Selection Type** | **Location (bp)** | **Range (bp)** | **Location (cM)** | **Range (cM)** | **λ(Ĥ_EA_)** | **λ(Ĥ_AA_)** | **λ(S^2^F_ST_)** | **Genes Included** |
| --- | --- | --- | --- | --- | --- | --- | --- | --- | --- | --- |
| 25 | **a** | new EA | 30,007,474 | - | 54.26 | - | 9.26x10^-6^ | 6.51x10^-2^ | 2.96x10^-4^ |  |
| 26 | **b** | new AA | 38,797,394 | 33,999 | 63.91 | 0.024 | 2.06x10^-2^ | 3.00x10^-4^ | 7.95x10^-5^ | *SCN10A* |
| 27 | **c** | old | 46,381,816 | 190,060 | 69.51 | 0.097 | 4.36x10^-7^ | 4.75x10^-6^ | 1.78x10^-1^ | *CCR1, CCR3, CCR5, LTF* |
| 28 | **d** | old | 51,001,110 | 861,930 | 70.93 | 0.081 | 3.88x10^-8^ | 1.39x10^-13^ | 9.03x10^-1^ | *LOC51161, HEMK1, CISH, MAPKAPK3, DOCK3, ARMET, HUMAGCGB, VprBP* |
| 29 | **e** | old | 63,882,277 | 148,374 | 87.56 | 0.259 | 2.67x10^-5^ | 4.07x10^-5^ | 7.88x10^-1^ | *LOC132200, NIF3L1BP1, SCA7* |
| 30 | **f** | new in both | 98,443,103 | 769,991 | 111.15 | 0.107 | 9.45x10^-11^ | 8.51x10^-5^ | 8.08x10^-6^ | *EPHA6, DKFZp434C* |
| 31 | **g** | new EA | 113,684,926 | 292,793 | 120.61 | 0.149 | 2.93x10^-10^ | 2.12x10^-1^ | 2.53x10^-5^ | *MOX2, BTLA, OR7E100P, APG3L, SLC35A5, URB* |
| 32 | **h** | new EA | 141,977,937 | 405,405 | 146.81 | 0.446 | 5.26x10^-7^ | 4.37x10^-1^ | 3.14x10^-4^ | *TRIM42, FLJ10618* |
| 33 | **i** | old | 157,912,316 | 14,614 | 164.18 | 0.008 | 3.79x10^-6^ | 1.29x10^-4^ | 9.40x10^-1^ | *TIPARP, LOC401095* |
| 34 | **j** | new EA | 160,940,773 | 87,204 | 165.75 | 0.014 | 1.39x10^-6^ | 5.10x10^-1^ | 1.30x10^-4^ |  |
| 35 | **k** | new AA | 170,111,911 | 12,183 | 170.12 | 0.011 | 8.20x10^-1^ | 5.27x10^-4^ | 3.76x10^-7^ |  |
| 36 | **l** | new AA | 190,062,424 | 31,900 | 201.46 | 0.075 | 2.44x10^-2^ | 4.30x10^-5^ | 6.40x10^-5^ |  |

**Table S3 (cont.)** Chromosome 4

| **Peak Number** | **Peak Name** | **Selection type** | **Location (bp)** | **Range (bp)** | **Location (cM)** | **Range (cM)** | **λ(Ĥ_EA_)** | **λ(Ĥ_AA_)** | **λ(S^2^F_ST_)** | **Genes Included** |
| --- | --- | --- | --- | --- | --- | --- | --- | --- | --- | --- |
| 37 | **a** | old | 88,056,666 | 108,751 | 94.89 | 0.108 | 7.37x10^-6^ | 3.54x10^-10^ | 4.46x10^-1^ | *PTPN13, SOAT* |
| 38 | **b** | new EA | 106,905,681 | 64,527 | 109.90 | 0.054 | 2.10x10^-5^ | 1.33x10^-3^ | 1.11x10^-4^ | *FLJ20184, LOC401147* |
| 39 | **c** | old | 119,530,185 | 159,483 | 121.65 | 0.075 | 9.94x10^-7^ | 9.97x10^-5^ | 2.01x10^-1^ | *NDST3, PRSS12* |
| 40 | **d** | new EA | 149,081,812 | 492,660 | 143.38 | 0.411 | 7.81x10^-9^ | 6.31x10^-1^ | 4.98x10^-6^ | *FLJ10846, LOC90826, ARHGAP10* |
| 41 | **e** | new EA | 152,469,847 | 968,597 | 145.99 | 0.851 | 1.48x10^-9^ | 4.44x10^-1^ | 4.02x10^-4^ | *LRBA, LOC441047, RPS3A, U73B, RNU73, DKFZp434D, ESSPL, LOC391705, PET112L* |

**Table S3 (cont.)** Chromosome 5

| **Peak Number** | **Peak Name** | **Selection type** | **Location (bp)** | **Range (bp)** | **Location (cM)** | **Range (cM)** | **λ(Ĥ_EA_)** | **λ(Ĥ_AA_)** | **λ(S^2^F_ST_)** | **Genes Included** |
| --- | --- | --- | --- | --- | --- | --- | --- | --- | --- | --- |
| 42 | **a** | new AA | 26,918,229 | - | 46.32 | - | 1.01x10^-2^ | 1.29x10^-4^ | 9.75x10^-5^ |  |
| 43 | **b** | new EA | 72,833,645 | 5,039 | 85.31 | 0.008 | 4.65x10^-6^ | 4.96x10^-1^ | 2.73x10^-4^ |  |
| 44 | **c** | old | 93,356,700 | 838,383 | 106.73 | 0.609 | 1.44x10^-13^ | 1.26x10^-6^ | 6.12x10^-1^ | *LOC441095, NR2F1, DKFZP564D, FLJ25680, KIAA0825, LOC401202* |
| 45 | **d** | new AA | 132,267,080 | - | 134.63 | - | 3.99x10^-4^ | 4.88x10^-4^ | 1.94x10^-8^ |  |
| 46 | **e** | old | 140,441,100 | 37,413 | 141.27 | 0.041 | 3.17x10^-5^ | 1.68x10^-5^ | 5.26x10^-1^ | *PCDHB2* |
| 47 | **f** | new EA | 142,105,412 | 258,902 | 143.52 | 0.411 | 4.18x10^-8^ | 2.31x10^-2^ | 5.50x10^-6^ | *FGF1, ARHGAP26* |
| 48 | **g** | old | 159,387,491 | 12,795 | 163.69 | 0.016 | 2.54x10^-5^ | 5.83x10^-4^ | 7.96x10^-1^ |  |
| 49 | **h** | old | 160,826,806 | 47,451 | 165.35 | 0.040 | 2.73x10^-6^ | 1.89x10^-5^ | 5.63x10^-1^ |  |

**Table S3 (cont.)** Chromosome 6

| **Peak Number** | **Peak Name** | **Selection type** | **Location (bp)** | **Range (bp)** | **Location (cM)** | **Range (cM)** | **λ(Ĥ_EA_)** | **λ(Ĥ_AA_)** | **λ(S^2^F_ST_)** | **Genes Included** |
| --- | --- | --- | --- | --- | --- | --- | --- | --- | --- | --- |
| 50 | **a** | old | 2,133,400 | 42,352 | 6.14 | 0.102 | 8.14x10^-10^ | 3.20x10^-4^ | 5.74x10^-2^ |  |
| 51 | **b** | new EA | 3,118,113 | 195,121 | 8.74 | 0.471 | 3.23x10^-9^ | 1.77x10^-1^ | 6.08x10^-6^ | *RIPK1, BPHL, TUBB, LOC442152, MGC8685, LOC389362, C6orf85* |
| 52 | **c** | new EA | 12,044,786 | 34,771 | 27.83 | 0.070 | 4.24x10^-6^ | 5.88x10^-1^ | 2.24x10^-4^ |  |
| 53 | **d** | old | 31,770,571 | 5,721 | 51.79 | 0.002 | 1.00x10^-7^ | 5.38x10^-4^ | 8.68x10^-1^ |  |
| 54 | **e** | new EA | 35,154,398 | 20,046 | 54.76 | 0.016 | 1.00x10^-7^ | 1.52x10^-1^ | 4.01x10^-4^ |  |
| 55 | **f** | new EA | 42,428,704 | 160,713 | 64.11 | 0.289 | 5.33x10^-6^ | 5.89x10^-2^ | 9.45x10^-5^ |  |
| 56 | **g** | old | 80,731,877 | 620 | 90.69 | 0.000 | 1.82x10^-7^ | 6.08x10^-4^ | 5.65x10^-2^ |  |
| 57 | **h** | new EA | 84,690,538 | 428,051 | 92.77 | 0.247 | 9.38x10^-12^ | 4.16x10^-1^ | 6.82x10^-15^ | *LOC401268, C6orf159, NCB5OR, C6orf117, C6orf84* |
| 58 | **i** | old | 88,531,354 | 513,947 | 95.04 | 0.437 | 3.23x10^-17^ | 1.87x10^-6^ | 2.26x10^-2^ | *SLC35A1, RARSL, ORC3L, C6orf166* |
| 59 | **j** | new EA | 108,590,184 | 442,549 | 111.91 | 0.796 | 1.00x10^-7^ | 1.10x10^-1^ | 1.80x10^-4^ | *SEC63, OSTM1, NR2E1, SNX3, LACE1* |
| 60 | **k** | old | 111,870,809 | 38,596 | 115.72 | 0.039 | 3.01x10^-5^ | 4.19x10^-6^ | 4.78x10^-4^ |  |
| 61 | **l** | new AA | 120,758,654 | 413,952 | 120.21 | 0.403 | 1.28x10^-1^ | 6.19x10^-4^ | 9.75x10^-14^ |  |
| 62 | **m** | old | 128,723,867 | 103,038 | 127.93 | 0.069 | 5.32x10^-13^ | 3.89x10^-4^ | 1.02x10^-1^ |  |
| 63 | **n** | new EA | 133,755,005 | 9,415 | 132.68 | 0.012 | 2.88x10^-7^ | 5.56x10^-1^ | 1.29x10^-7^ |  |
| 64 | **o** | new EA | 137,377,208 | 29,296 | 139.16 | 0.055 | 2.25x10^-10^ | 9.30x10^-1^ | 1.98x10^-11^ | *IL20RA* |
| 65 | **p** | new EA | 146,562,209 | 18,844 | 147.96 | 0.015 | 3.01x10^-5^ | 8.96x10^-2^ | 5.07x10^-5^ |  |
| 66 | **q** | new EA | 159,940,054 | 12,712 | 169.12 | 0.018 | 3.89x10^-6^ | 5.71x10^-2^ | 3.19x10^-4^ |  |
| 67 | **r** | new AA | 169,934,860 | 128,960 | 187.32 | 0.250 | 1.31x10^-1^ | 3.60x10^-4^ | 4.42x10^-4^ | *MGC43690, PHF10, TCTE3, C6orf70* |

**Table S3 (cont.)** Chromosome 7

| **Peak Number** | **Peak Name** | **Selection type** | **Location (bp)** | **Range (bp)** | **Location (cM)** | **Range (cM)** | **λ(Ĥ_EA_)** | **λ(Ĥ_AA_)** | **λ(S^2^F_ST_)** | **Genes Included** |
| --- | --- | --- | --- | --- | --- | --- | --- | --- | --- | --- |
| 68 | **a** | new AA | 92,327,527 | 126,852 | 103.38 | 0.077 | 3.37x10^-3^ | 2.80x10^-4^ | 3.52x10^-4^ | *SAMD9* |
| 69 | **b** | new EA | 98,706,812 | 833,287 | 109.38 | 0.751 | 6.40x10^-10^ | 1.06x10^-1^ | 2.71x10^-19^ | *SMURF1, LOC401391, ARPC1A, ARPC1B, PDAP1, G10, PTCD1, CPSF4, ATP5J2, LOC285989, ZNF394, ZFP95, DKFZp727G, VIK, ZNF498, CYP3A5, CYP3A5P1, CYP3A7, CYP3A5P2, CYP3A4, CYP3A43* |
| 70 | **c** | old | 103,854,954 | - | 113.32 | - | 2.24x10^-6^ | 5.89x10^-4^ | 3.25x10^-1^ |  |
| 71 | **d** | old | 113,866,430 | 679,260 | 121.71 | 0.349 | 9.83x10^-7^ | 4.91x10^-5^ | 2.23x10^-1^ | *FOXP2, HIC* |
| 72 | **e** | old | 121,731,797 | 19,044 | 125.99 | 0.010 | 2.12x10^-8^ | 4.61x10^-4^ | 7.88x10^-1^ |  |
| 73 | **f** | old | 129,106,198 | 27,171 | 131.33 | 0.027 | 3.53x10^-5^ | 3.56x10^-5^ | 6.44x10^-1^ |  |
| 74 | **g** | new EA | 131,603,757 | 128,749 | 136.33 | 0.271 | 1.58x10^-8^ | 6.36x10^-2^ | 8.85x10^-7^ | *DKFZp434G* |
| 75 | **h** | new in both | 140,324,112 | 980,988 | 149.11 | 1.000 | 1.97x10^-8^ | 1.88x10^-4^ | 2.71x10^-4^ | *ADCK2, NDUFB2, BRAF, MRPS33, LOC441284, LOC401410, FLJ10842* |

**Table S3 (cont.)** Chromosome 8

| **Peak Number** | **Peak Name** | **Selection type** | **Location (bp)** | **Range (bp)** | **Location (cM)** | **Range (cM)** | **λ(Ĥ_EA_)** | **λ(Ĥ_AA_)** | **λ(S^2^F_ST_)** | **Genes Included** |
| --- | --- | --- | --- | --- | --- | --- | --- | --- | --- | --- |
| 76 | **a** | new EA | 30,863,685 | 270,129 | 52.35 | 0.372 | 2.30x10^-5^ | 6.06x10^-1^ | 1.12x10^-11^ | *D8S2298E, PPP2CB, TEX15, LOC441344* |
| 77 | **b** | new EA | 42,629,374 | 890,446 | 60.72 | 0.208 | 1.89x10^-15^ | 7.31x10^-1^ | 1.55x10^-9^ | *PLAT, LOC389650, IKBKB, POLB, DKK4, VDAC3, SLC20A2, LOC114926, CHRNB3, CHRNA6, THAP1, DKFZP564A, HOOK3, FNTA, FLJ23356* |
| 78 | **c** | new EA | 67,451,263 | 137,962 | 77.05 | 0.080 | 1.72x10^-6^ | 6.51x10^-2^ | 4.09x10^-4^ | *RRS1, ADHFE1* |
| 79 | **d** | old | 71,263,569 | 67,293 | 81.49 | 0.118 | 2.99x10^-5^ | 5.35x10^-6^ | 9.12x10^-1^ |  |
| 80 | **e** | old | 110,663,841 | 24,598 | 114.86 | 0.012 | 3.15x10^-6^ | 3.80x10^-4^ | 6.01x10^-2^ | *FLJ20366* |

**Table S3 (cont.)** Chromosome 9

| **Peak Number** | **Peak Name** | **Selection type** | **Location (bp)** | **Range (bp)** | **Location (cM)** | **Range (cM)** | **λ(Ĥ_EA_)** | **λ(Ĥ_AA_)** | **λ(S^2^F_ST_)** | **Genes Included** |
| --- | --- | --- | --- | --- | --- | --- | --- | --- | --- | --- |
| 81 | **a** | old | 6,424,611 | 7,040 | 14.55 | 0.012 | 4.60x10^-6^ | 1.55x10^-4^ | 6.87x10^-1^ |  |
| 82 | **b** | new EA | 20,309,127 | 181,348 | 40.92 | 0.284 | 1.45x10^-5^ | 5.67x10^-1^ | 2.51x10^-9^ | *MLLT3* |
| 83 | **c** | old | 71,582,148 | 5,183 | 67.51 | 0.003 | 9.17x10^-6^ | 5.65x10^-4^ | 5.91x10^-1^ |  |
| 84 | **d** | old | 74,759,766 | 209,510 | 70.70 | 0.245 | 1.27x10^-5^ | 3.49x10^-4^ | 3.54x10^-1^ | *TRPM6, C9orf40, C9orf41* |
| 85 | **e** | old | 96,257,070 | 153,759 | 99.12 | 0.086 | 2.94x10^-10^ | 1.15x10^-8^ | 4.24x10^-2^ | *SLC35D2, ZNF367, HABP4, CDC14B* |
| 86 | **f** | old | 105,366,775 | 121,836 | 108.14 | 0.145 | 2.05x10^-10^ | 4.84x10^-4^ | 1.79x10^-1^ | *CSDUFD1, MGC45564, FCMD* |
| 87 | **g** | old | 107,157,694 | 42,799 | 110.42 | 0.056 | 1.03x10^-5^ | 4.15x10^-4^ | 2.47x10^-2^ | *RAD23B* |
| 88 | **h** | old | 112,299,713 | 3,961 | 117.20 | 0.006 | 2.83x10^-5^ | 3.71x10^-5^ | 9.83x10^-1^ |  |
| 89 | **i** | new EA | 122,771,883 | 891,072 | 130.17 | 0.953 | 1.78x10^-7^ | 1.71x10^-1^ | 4.59x10^-14^ | *OR1J2, OR1J4, OR1N1, OR1N2, OR1L8, OR1H1P, OR1Q1, OR1B1, LOC158130, OR1L6, OR5C1, LOC392392, PDCL, MNAB, ZNF482, ZBTB26, RABGAP1, GPR21, C9orf45, STRBP, CRB2* |
| 90 | **j** | new EA | 124,363,223 | 17,636 | 132.04 | 0.022 | 1.04x10^-5^ | 4.36x10^-1^ | 1.82x10^-5^ | *NR6A1* |

**Table S3 (cont.)** **Chromosome 10**

| **Peak Number** | **Peak Name** | **Selection type** | **Location (bp)** | **Range (bp)** | **Location (cM)** | **Range (cM)** | **λ(Ĥ_EA_)** | **λ(Ĥ_AA_)** | **λ(S^2^F_ST_)** | **Genes Included** |
| --- | --- | --- | --- | --- | --- | --- | --- | --- | --- | --- |
| 91 | **a** | old | 18,923,671 | - | 43.34 | - | 2.68x10^-5^ | 2.81x10^-4^ | 3.74x10^-1^ |  |
| 92 | **b** | new EA | 31,788,090 | 109,133 | 59.90 | 0.233 | 5.93x10^-10^ | 2.09x10^-1^ | 4.59x10^-9^ | *TCF8* |
| 93 | **c** | new AA | 35,221,302 | - | 61.83 | - | 3.85x10^-2^ | 6.23x10^-4^ | 3.41x10^-4^ |  |
| 94 | **d** | old | 38,079,953 | 386,852 | 63.08 | 0.198 | 2.02x10^-7^ | 1.26x10^-4^ | 9.92x10^-1^ | *LOC219752, LOC399740, ZNF248, LOC387654, BA775A3.1, BA393J16.* |
| 95 | **e** | old | 50,129,172 | 306,287 | 69.17 | 0.174 | 4.37x10^-6^ | 3.42x10^-6^ | 1.51x10^-1^ | *C10orf72, C10orf73, C10orf128, C10orf71, PRRXL1* |
| 96 | **f** | old | 70,267,226 | 25,978 | 85.56 | 0.028 | 5.46x10^-7^ | 6.11x10^-4^ | 8.50x10^-1^ |  |
| 97 | **g** | new EA | 74,500,295 | 328,448 | 93.47 | 0.163 | 6.74x10^-10^ | 7.01x10^-1^ | 4.73x10^-5^ | *OIT3, PLA2G12B, LOC148854, P4HA1, NUDT13, HSGT1* |
| 98 | **h** | old | 87,781,123 | 15,731 | 106.76 | 0.012 | 1.07x10^-5^ | 4.78x10^-4^ | 7.37x10^-1^ |  |
| 99 | **i** | new EA | 111,859,027 | 41,377 | 127.51 | 0.032 | 8.84x10^-7^ | 2.17x10^-1^ | 3.16x10^-6^ |  |
| 100 | **j** | old | 121,749,892 | 376,789 | 142.81 | 0.674 | 2.88x10^-9^ | 3.00x10^-10^ | 8.92x10^-1^ | *INPP5F, C10orf119, SEC23IP, LOC441576* |
| 101 | **k** | new EA | 127,126,142 | 427,695 | 154.69 | 1.169 | 2.38x10^-5^ | 5.84x10^-1^ | 1.44x10^-5^ | *C10orf122* |
| 102 | **l** | old | 134,885,792 | 159,227 | 173.78 | 0.401 | 9.68x10^-8^ | 1.15x10^-5^ | 8.71x10^-2^ | *GPR123, KNDC1, UTF1, VENTX2, ADAM8* |

**Table S3 (cont.) Chro**mosome 11

| **Peak Number** | **Peak Name** | **Selection type** | **Location (bp)** | **Range (bp)** | **Location (cM)** | **Range (cM)** | **λ(Ĥ_EA_)** | **λ(Ĥ_AA_)** | **λ(S^2^F_ST_)** | **Genes Included** |
| --- | --- | --- | --- | --- | --- | --- | --- | --- | --- | --- |
| 103 | **a** | new EA | 4,044,297 | 51,348 | 6.41 | 0.090 | 2.58x10^-5^ | 6.96x10^-2^ | 1.41x10^-5^ |  |
| 104 | **b** | new EA | 46,129,281 | 396,124 | 63.25 | 0.127 | 3.85x10^-9^ | 3.77x10^-2^ | 2.83x10^-5^ | *BHC80, LOC401679, CREB3L1, DGKZ* |
| 105 | **c** | new EA | 61,010,827 | 138,188 | 66.56 | 0.139 | 3.16x10^-8^ | 2.26x10^-2^ | 3.12x10^-4^ | *FLJ12529, FLJ20487, FLJ32771, LOC390205, SYT7* |
| 106 | **d** | new EA | 66,707,556 | 547,228 | 71.87 | 0.433 | 5.00x10^-15^ | 2.91x10^-1^ | 7.72x10^-16^ | *PC, LOC254439, SYT12, RHOD, FBXL11, ADRBK1, LOC338692, SSH3, POLD4, CLC, RAD9A, PPP1CA, FLJ00332, KIAA1394, RPS6KB2, PTPRCAP, CORO1B, GPR152, CABP4* |
| 107 | **e** | old | 72,835,460 | 417,771 | 79.22 | 0.511 | 2.43x10^-9^ | 5.35x10^-4^ | 2.27x10^-2^ | *OR8R1P, P2RY6, ARHGEF17, TNFRSF19L, KIAA0280, PLEKHB1* |
| 108 | **f** | old | 83,526,029 | 317,141 | 89.45 | 0.171 | 7.00x10^-16^ | 5.84x10^-5^ | 1.27x10^-2^ |  |
| 109 | **g** | new AA | 94,949,561 | 413,247 | 97.39 | 0.567 | 6.29x10^-1^ | 3.22x10^-4^ | 5.85x10^-5^ | *MGC33371* |
| 110 | **h** | new in both | 106,143,678 | 146,127 | 106.94 | 0.095 | 1.00x10^-7^ | 3.51x10^-4^ | 4.30x10^-6^ |  |
| 111 | **i** | old | 108,132,821 | 136,116 | 108.28 | 0.087 | 1.22x10^-8^ | 5.80x10^-6^ | 3.02x10^-2^ | *HCP29* |
| 112 | **j** | new in both | 116,378,438 | 283,935 | 118.36 | 0.444 | 2.20x10^-5^ | 6.15x10^-4^ | 2.70x10^-5^ | *KIAA0999, LOC196266, PAFAH1B2* |

**Table S3 (cont.)** Chromosome 12

| **Peak Number** | **Peak Name** | **Selection type** | **Location (bp)** | **Range (bp)** | **Location (cM)** | **Range (cM)** | **λ(Ĥ_EA_)** | **λ(Ĥ_AA_)** | **λ(S^2^F_ST_)** | **Genes Included** |
| --- | --- | --- | --- | --- | --- | --- | --- | --- | --- | --- |
| 113 | **a** | old | 2,276,705 | 38,689 | 4.21 | 0.153 | 5.76x10^-9^ | 4.09x10^-4^ | 2.47x10^-1^ |  |
| 114 | **b** | new EA | 19,371,656 | 253,811 | 37.24 | 0.260 | 3.12x10^-13^ | 5.15x10^-3^ | 7.13x10^-5^ | *PEPP2, AEBP2* |
| 115 | **c** | old | 39,700,633 | 68,128 | 57.27 | 0.020 | 4.80x10^-7^ | 3.52x10^-5^ | 5.10x10^-1^ |  |
| 116 | **d** | old | 43,979,331 | 964,810 | 60.06 | 0.731 | 1.81x10^-11^ | 8.50x10^-6^ | 7.21x10^-1^ | *NELL2, LOC440097, FKSG42, LOC51054, TMEM16F, LOC400027, ARID2* |
| 117 | **e** | old | 54,654,766 | 141,748 | 71.26 | 0.124 | 2.52x10^-10^ | 1.53x10^-4^ | 1.29x10^-2^ | *PYM, DGKA, SILV, CDK2, RAB5B, SUOX, ZNFN1A4, RPS26* |
| 118 | **f** | new EA | 78,768,401 | 1,138,385 | 94.04 | 1.226 | 6.95x10^-8^ | 2.93x10^-1^ | 1.93x10^-6^ | *SYT1, LOC338756, PAWR, PPP1R12A, LOC120872, LOC400055, FLJ90579, LOC387870* |
| 119 | **g** | new EA | 81,776,709 | - | 96.66 | - | 7.78x10^-6^ | 1.65x10^-1^ | 3.61x10^-4^ |  |
| 120 | **h** | new EA | 87,570,142 | 1,351,831 | 99.14 | 0.821 | 2.00x10^-7^ | 5.09x10^-1^ | 7.73x10^-6^ | *FLJ35821, LOC440106, KIAA0373, FLJ13615, SMILE, KITLG, MRPS6P4, DUSP6* |
| 121 | **i** | old | 95,213,628 | 70,206 | 107.62 | 0.093 | 1.11x10^-8^ | 1.97x10^-4^ | 3.52x10^-1^ |  |

**Table S3 (cont.)** Chromosome 13

| **Peak Number** | **Peak Name** | **Selection type** | **Location (bp)** | **Range (bp)** | **Location (cM)** | **Range (cM)** | **λ(Ĥ_EA_)** | **λ(Ĥ_AA_)** | **λ(S^2^F_ST_)** | **Genes Included** |
| --- | --- | --- | --- | --- | --- | --- | --- | --- | --- | --- |
| 122 | **a** | new EA | 24,978,902 | 43,274 | 14.32 | 0.100 | 3.52x10^-5^ | 1.05x10^-2^ | 4.61x10^-5^ |  |
| 123 | **b** | old | 40,957,177 | 463,213 | 43.39 | 0.455 | 4.31x10^-8^ | 2.88x10^-12^ | 4.16x10^-1^ | *MTRF1, FLJ22054, LOC387922, OR7E31P, OR7E36P, OR7E155P, OR7E37P, RGC32, KIAA0564* |
| 124 | **c** | new AA | 42,816,872 | 12,251 | 45.68 | 0.018 | 3.56x10^-2^ | 5.47x10^-4^ | 3.80x10^-4^ |  |
| 125 | **d** | new EA | 44,454,114 | 99,266 | 47.92 | 0.142 | 6.19x10^-7^ | 6.49x10^-1^ | 3.44x10^-5^ | *NUFIP1, KIAA1704* |
| 126 | **e** | new EA | 47,777,009 | 420,431 | 51.79 | 0.278 | 4.53x10^-12^ | 1.01x10^-1^ | 1.42x10^-8^ | *VDRIP, ITM2B, RB1, P2RY5, CHC1L* |
| 127 | **f** | new EA | 71,722,516 | 953,637 | 65.26 | 1.511 | 6.18x10^-8^ | 5.05x10^-1^ | 2.60x10^-5^ | *DACH1, LOC440145* |
| 128 | **g** | old | 93,801,957 | 112,343 | 86.11 | 0.194 | 2.78x10^-6^ | 2.66x10^-5^ | 1.83x10^-1^ | *GPC6* |
| 129 | **h** | old | 97,442,952 | 605 | 91.22 | 0.001 | 1.27x10^-5^ | 6.40x10^-5^ | 9.45x10^-2^ |  |

**Table S3 (cont.)** Chromosome 14

| **Peak Number** | **Peak Name** | **Selection type** | **Location (bp)** | **Range (bp)** | **Location (cM)** | **Range (cM)** | **λ(Ĥ_EA_)** | **λ(Ĥ_AA_)** | **λ(S^2^F_ST_)** | **Genes Included** |
| --- | --- | --- | --- | --- | --- | --- | --- | --- | --- | --- |
| 130 | **a** | new EA | 56,809,815 | 14,136 | 57.55 | 0.014 | 1.99x10^-5^ | 3.29x10^-1^ | 2.16x10^-14^ | *C14orf108* |
| 131 | **b** | old | 58,458,966 | 248,173 | 59.17 | 0.223 | 3.33x10^-6^ | 3.29x10^-8^ | 9.43x10^-1^ | *LOC387991, LOC440181* |
| 132 | **c** | new in both | 60,814,984 | 517,343 | 61.25 | 0.399 | 5.51x10^-13^ | 4.44x10^-4^ | 1.77x10^-8^ | *SLC38A6, TMEM30B, PRKCH* |
| 133 | **d** | new EA | 63,292,531 | 388,072 | 63.35 | 0.314 | 3.74x10^-6^ | 6.89x10^-1^ | 1.10x10^-4^ | *GCATP, C14orf150, HSPEP2, SGPP1, EIF2S2P, SYNE2* |
| 134 | **e** | new EA | 66,236,136 | 1,493,262 | 65.17 | 1.145 | 6.53x10^-9^ | 4.17x10^-1^ | 1.81x10^-6^ | *NSEP1P, LOC440184, GPHN, C14orf54, LOC260329, MPP5, ATP6V1D, EIF2S1, PLEK2* |
| 135 | **f** | old | 71,827,846 | 1,042 | 71.12 | 0.001 | 2.27x10^-5^ | 1.75x10^-4^ | 1.80x10^-1^ |  |

**Table S3 (cont.)** Chromosome 15

| **Peak Number** | **Peak Name** | **Selection type** | **Location (bp)** | **Range (bp)** | **Location (cM)** | **Range (cM)** | **λ(Ĥ_EA_)** | **λ(Ĥ_AA_)** | **λ(S^2^F_ST_)** | **Genes Included** |
| --- | --- | --- | --- | --- | --- | --- | --- | --- | --- | --- |
| 136 | **a** | new EA | 23,097,946 | 135,032 | 9.70 | 0.323 | 2.48x10^-5^ | 1.12x10^-2^ | 2.09x10^-5^ | *HBII^-4^38B, UBE3A* |
| 137 | **b** | new EA | 27,184,680 | 68,866 | 19.42 | 0.165 | 5.71x10^-7^ | 2.38x10^-2^ | 9.73x10^-9^ | *APBA2* |
| 138 | **c** | new EA | 42,231,163 | 751,421 | 44.02 | 0.365 | 3.16x10^-8^ | 8.70x10^-3^ | 2.16x10^-4^ | *ELL3, SERF2, HYPK, MFAP1, FLJ12973, MGC14161, ACTBP7, H63, HSPC129* |
| 139 | **d** | new EA | 67,155,853 | - | 72.61 | - | 2.21x10^-5^ | 1.82x10^-1^ | 2.06x10^-4^ |  |
| 140 | **e** | new EA | 70,466,010 | 742,530 | 77.56 | 0.743 | 2.82x10^-12^ | 1.65x10^-3^ | 6.33x10^-7^ | *MYO9A, SENP8, LOC196996, PKM2, LOC400388, LOC56965, BRUNOL6, HEXA, LOC400389, LOC338949, ARIH1, GOLGA6, LOC123346, BBS4, ADP-GK* |

**Table S3 (cont.) Ch**romosome 16

| **Peak Number** | **Peak Name** | **Selection type** | **Location (bp)** | **Range (bp)** | **Location (cM)** | **Range (cM)** | **λ(Ĥ_EA_)** | **λ(Ĥ_AA_)** | **λ(S^2^F_ST_)** | **Genes Included** |
| --- | --- | --- | --- | --- | --- | --- | --- | --- | --- | --- |
| 141 | **a** | new AA | 4,256,169 | - | 8.05 | - | 8.24x10^-2^ | 2.55x10^-4^ | 7.52x10^-6^ |  |
| 142 | **b** | new AA | 14,387,485 | 246,127 | 32.99 | 0.421 | 3.20x10^-3^ | 7.25x10^-5^ | 2.99x10^-5^ | *MRTF-B, LOC388214, PARN* |
| 143 | **c** | old | 14,983,949 | 1,439,056 | 35.35 | 2.373 | 1.86x10^-5^ | 1.96x10^-8^ | 4.56x10^-3^ | *MRTF-B, LOC388214, PARN, BFAR, PLA2G10, PM5, PKD1P3, NPIP, KIAA0251, NTAN1, RRN3, LOC441749, PKD1P6, LOC440341, FLJ39599, BC008967, LKAP, NDE1* |

**Table S3 (cont.)** Chromosome 17

| **Peak Number** | **Peak Name** | **Selection type** | **Location (bp)** | **Range (bp)** | **Location (cM)** | **Range (cM)** | **λ(Ĥ_EA_)** | **λ(Ĥ_AA_)** | **λ(S^2^F_ST_)** | **Genes Included** |
| --- | --- | --- | --- | --- | --- | --- | --- | --- | --- | --- |
| 144 | **a** | old | 5,355,837 | 146,478 | 14.56 | 0.371 | 7.24x10^-7^ | 8.71x10^-5^ | 2.72x10^-1^ | *C1QBP, DHX33, F-LANa, MIS12, LOC440398, NALP1* |
| 145 | **b** | new EA | 19,260,539 | 1,864 | 48.77 | 0.001 | 1.18x10^-5^ | 2.22x10^-1^ | 8.88x10^-6^ | *ZNF179* |
| 146 | **c** | new in both | 26,385,188 | 269,667 | 53.58 | 0.204 | 3.75x10^-5^ | 5.81x10^-4^ | 7.94x10^-8^ | *FLJ22729, RNF135, CENTA2, LOC400590, NF1* |
| 147 | **d** | old | 37,346,118 | 62,475 | 70.53 | 0.000 | 3.16x10^-7^ | 4.70x10^-4^ | 3.52x10^-1^ |  |
| 148 | **e** | new EA | 38,868,315 | 66,668 | 71.76 | 0.049 | 1.03x10^-5^ | 1.88x10^-1^ | 3.50x10^-5^ |  |
| 149 | **f** | new EA | 55,896,078 | 1,676,885 | 89.29 | 1.540 | 3.33x10^-9^ | 2.73x10^-2^ | 3.54x10^-5^ | *CLTC, Bit1, VMP1, TUBD1, RPS6KB1, LOC51136, LOC390805, LOC400609, LOC441797, ABC1, LOC440449, LOC441798, CA4, USP32, LOC124773, LOC284166, APPBP2, LOC388406, PPM1D, BCAS3* |

**Table S3 (cont.)** **Chromosome 18**

| **Peak Number** | **Peak Name** | **Selection type** | **Location (bp)** | **Range (bp)** | **Location (cM)** | **Range (cM)** | **λ(Ĥ_EA_)** | **λ(Ĥ_AA_)** | **λ(S^2^F_ST_)** | **Genes Included** |
| --- | --- | --- | --- | --- | --- | --- | --- | --- | --- | --- |
| 150 | **a** | old | 22,039,207 | 142,199 | 48.32 | 0.196 | 1.95x10^-6^ | 3.26x10^-4^ | 1.02x10^-2^ | *MGC26605, TAF4B* |
| 151 | **b** | new EA | 32,977,250 | 64,761 | 58.20 | 0.076 | 1.37x10^-5^ | 3.10x10^-3^ | 2.80x10^-4^ |  |
| 152 | **c** | new EA | 52,376,945 | 1,000,035 | 77.75 | 1.153 | 1.55x10^-7^ | 1.06x10^-1^ | 3.25x10^-7^ | *TXNL1, WDR7, LOC440494* |
| 153 | **d** | old | 58,598,121 | 45,051 | 87.43 | 0.058 | 8.63x10^-6^ | 2.43x10^-5^ | 3.55x10^-1^ |  |

**Table S3 (cont.)** Chromosome 19

| **Peak Number** | **Peak Name** | **Selection Type** | **Location (bp)** | **Range (bp)** | **Location (cM)** | **Range (cM)** | **λ(Ĥ_EA_)** | **λ(Ĥ_AA_)** | **λ(S^2^F_ST_)** | **Genes Included** |
| --- | --- | --- | --- | --- | --- | --- | --- | --- | --- | --- |
| 154 | **a** | new EA | 11,418,225 | 429,044 | 30.88 | 0.642 | 5.42x10^-6^ | 5.37x10^-1^ | 5.32x10^-10^ | *LOC55908, DOCK6, TM4-B, RAB3D, UNQ501, LOC126075, LPPR2, FLJ35119, EPOR, RGL3, MGC20983, PRKCSH, ELAVL3, LOC115950, SITPEC, CNN1, MGC4549, ACP5, LOC441837, ZNF627, LOC342970* |
| 155 | **b** | new EA | 44,528,505 | 33,725 | 65.06 | 0.042 | 1.16x10^-5^ | 1.32x10^-2^ | 3.65x10^-7^ | *GMFG, FLJ10211, ZFP36, CLG* |

**Table S3 (cont.)** **Chromosome 20**

| **Peak Number** | **Peak Name** | **Selection type** | **Location (bp)** | **Range (bp)** | **Location (cM)** | **Range (cM)** | **λ(Ĥ_EA_)** | **λ(Ĥ_AA_)** | **λ(S^2^F_ST_)** | **Genes Included** |
| --- | --- | --- | --- | --- | --- | --- | --- | --- | --- | --- |
| 156 | **a** | new AA | 7,849,387 | 3,591 | 25.07 | 0.010 | 2.56x10^-3^ | 2.98x10^-5^ | 3.74x10^-4^ |  |
| 157 | **b** | new EA | 22,370,861 | 306,841 | 51.95 | 0.224 | 6.28x10^-7^ | 9.71x10^-1^ | 3.83x10^-4^ | *LOC284788, C20orf56, FOXA2* |
| 158 | **c** | old | 38,349,225 | 4,684 | 62.06 | 0.005 | 1.42x10^-5^ | 1.83x10^-4^ | 3.25x10^-2^ |  |
| 159 | **d** | old | 61,973,760 | 7,305 | 104.63 | 0.014 | 3.03x10^-5^ | 2.82x10^-5^ | 9.90x10^-1^ |  |

**Table S3 (cont.)** Chromosome 21

| **Peak Number** | **Peak Name** | **Selection type** | **Location (bp)** | **Range (bp)** | **Location (cM)** | **Range (cM)** | **λ(Ĥ_EA_)** | **λ(Ĥ_AA_)** | **λ(S^2^F_ST_)** | **Genes Included** |
| --- | --- | --- | --- | --- | --- | --- | --- | --- | --- | --- |
| 160 | **a** | old | 15,747,958 | 15,839 | 7.76 | 0.036 | 2.10x10^-9^ | 2.30x10^-4^ | 6.63x10^-2^ |  |
| 161 | **b** | new EA | 16,497,420 | - | 9.48 | - | 4.25x10^-6^ | 3.45x10^-2^ | 3.61x10^-4^ |  |
| 162 | **c** | old | 21,022,362 | 114,073 | 18.97 | 0.168 | 6.35x10^-15^ | 1.36x10^-8^ | 3.10x10^-1^ |  |
| 163 | **d** | old | 26,699,076 | 11,526 | 27.09 | 0.021 | 3.26x10^-5^ | 2.91x10^-5^ | 1.16x10^-2^ |  |
| 164 | **e** | old | 27,700,719 | 235,222 | 28.80 | 0.333 | 8.92x10^-11^ | 5.00x10^-4^ | 7.47x10^-1^ | *EIF4A1P, RPL10P1* |
| 165 | **f** | new EA | 29,932,975 | 322,908 | 31.37 | 0.299 | 4.74x10^-9^ | 6.98x10^-3^ | 4.52x10^-12^ | *GRIK1* |
| 166 | **g** | old | 31,588,861 | 138,358 | 33.49 | 0.282 | 1.00x10^-7^ | 1.35x10^-4^ | 9.01x10^-1^ |  |
| 167 | **h** | new AA | 33,841,228 | 413,878 | 37.65 | 0.739 | 1.35x10^-1^ | 6.45x10^-10^ | 1.04x10^-5^ | *IFNAR1, IFNGR2, C21orf4, RPS5L, C21orf55, GART, SON, DONSON, CRYZL1, ITSN1* |
| 168 | **i** | old | 37,724,893 | 30,688 | 45.44 | 0.051 | 1.38x10^-5^ | 1.80x10^-9^ | 7.77x10^-1^ |  |
| 169 | **j** | old | 38,807,632 | 72,417 | 47.91 | 0.167 | 2.16x10^-8^ | 4.64x10^-5^ | 1.47x10^-1^ |  |
| 170 | **k** | old | 43,206,725 | - | 61.31 | - | 2.28x10^-5^ | 6.08x10^-6^ | 3.57x10^-1^ |  |
| 171 | **l** | old | 45,022,806 | 873 | 67.36 | 0.003 | 2.24x10^-5^ | 1.83x10^-6^ | 6.71x10^-1^ |  |

**Table S3 (cont.)** Chromosome 22

| **Peak Number** | **Peak Name** | **Selection type** | **Location (bp)** | **Range (bp)** | **Location (cM)** | **Range (cM)** | **λ(Ĥ_EA_)** | **λ(Ĥ_AA_)** | **λ(S^2^F_ST_)** | **Genes Included** |
| --- | --- | --- | --- | --- | --- | --- | --- | --- | --- | --- |
| 172 | **a** | old | 16,009,502 | 21,751 | (6.06) | 0.075 | 3.01x10^-5^ | 1.23x10^-6^ | 2.25x10^-2^ |  |
| 173 | **b** | old | 17,618,403 | 94,241 | (0.62) | 0.325 | 7.06x10^-13^ | 3.22x10^-8^ | 4.13x10^-1^ | *CLTCL1* |
| 174 | **c** | old | 26,901,511 | 377,156 | 31.68 | 1.424 | 1.29x10^-19^ | 2.07x10^-11^ | 8.94x10^-1^ | *KIAA1043* |
| 175 | **d** | new EA | 33,891,400 | 44,287 | 39.68 | 0.074 | 2.56x10^-22^ | 9.81x10^-1^ | 1.19x10^-8^ |  |
| 176 | **e** | new in both | 40,367,112 | 400,817 | 49.44 | 0.129 | 1.00x10^-7^ | 1.76x10^-5^ | 2.91x10^-5^ | *TOB2, PHF5A, ACO2, POLR3H, PIPPIN, PMM1, D15Wsu75e, G22P1, NHP2L1, FLJ23584, HMG17L2, FLJ22349, SREBF2* |
| 177 | **f** | new AA | 44,087,807 | 110,832 | 58.43 | 0.439 | 7.17x10^-1^ | 3.22x10^-7^ | 4.94x10^-10^ | *C22orf8, SMC1L2, C22orf11* |
| 178 | **g** | old | 48,320,554 | 21,755 | 75.10 | 0.086 | 6.89x10^-11^ | 5.08x10^-5^ | 2.96x10^-1^ | *LOC440835* |

**Table S3 (cont.)** **Chromosome X^†^**

| **Peak Number** | **Peak Name** | **Selection type** | **Location (bp)** | **Range (bp)** | **Location (cM)** | **Range (cM)** | **λ(Ĥ_EA_)** | **λ(Ĥ_AA_)** | **λ(S^2^F_ST_)** | **Genes Included** |
| --- | --- | --- | --- | --- | --- | --- | --- | --- | --- | --- |
| 179 | **a** | new AA | 66,498,139 | 2,676,779 | 82.15 | 1.424 | 1.31x10^-2^ | 2.81x10^-5^ | 1.82x10^-12^ | *HEPH, LOC392486, LOC402408, XEDAR, AR, LOC442457, OPHN1, PGK1P1, MGC21416, LOC392487, STARD8, LOC389866, EFNB1* |
| 180 | **b** | new EA | 104,488,529 | 384,427 | 104.18 | 0.076 | 8.15x10^-7^ | 8.51x10^-3^ | 3.14x10^-5^ |  |

**^†^** The lack of selected sites in the chromosome X is adversely impacted by the scarcity of markers genotyped (Table S2) and the low power derived from genotyping hemizygous males.
