# Supplementary material for: Identifying Selected Regions from Heterozygosity and Divergence Using a Light-Coverage Genomic Dataset from Two Human Populations
Source: PLoS One. 2008 Mar 5;3(3):e1712. doi: 10.1371/journal.pone.0001712 (PMC2248624; doi:10.1371/journal.pone.0001712)
Supplement: Table S4 — Locations of the cross-validated regions. (0.95 MB DOC) [file pone.0001712.s005.doc]

Table S4. Locations of the cross-validated regions.

| **Chromosome** | **Region name** | **Beginning (bp)†** | **End (bp)** | **Author‡** | **Peak name^^** |
| --- | --- | --- | --- | --- | --- |
| **1** | **1.01** | **32,190,887** | **32,500,000** |  |  |
|  |  | 32,000,000 | 32,500,000 | Hapmap |  |
|  |  | 32,190,887 | 32,909,278 | Oleksyk | d |
|  | **1.02** | **35,109,198** | **35,115,000** |  |  |
|  |  | 35,100,000 | 35,300,000 | Hapmap II |  |
|  |  | 35,109,198 | 35,164,815 | Sabeti |  |
|  | **1.03** | **35,116,691** | **35,164,815** |  |  |
|  |  | 35,100,000 | 35,300,000 | Hapmap II |  |
|  |  | 35,116,691 | 36,614,746 | Oleksyk | e |
|  |  | 35,109,198 | 35,164,815 | Sabeti |  |
|  | **1.04** | **35,165,000** | **35,215,000** |  |  |
|  |  | 35,100,000 | 35,300,000 | Hapmap II |  |
|  |  | 35,116,691 | 36,614,746 | Oleksyk | e |
|  | **1.05** | **35,220,000** | **35,300,000** |  |  |
|  |  | 35,220,000 | 36,210,000 | Carlson |  |
|  |  | 35,100,000 | 35,300,000 | Hapmap II |  |
|  |  | 35,116,691 | 36,614,746 | Oleksyk | e |
|  | **1.06** | **35,305,000** | **36,210,000** |  |  |
|  |  | 35,220,000 | 36,210,000 | Carlson |  |
|  |  | 35,116,691 | 36,614,746 | Oleksyk | e |
|  | **1.07** | **72,310,000** | **72,400,000** |  |  |
|  |  | 72,310,000 | 72,790,000 | Carlson |  |
|  |  | 72,300,000 | 72,400,000 | Voght |  |
|  | **1.08** | **73,100,000** | **73,200,000** |  |  |
|  |  | 73,050,000 | 73,650,000 | Hapmap II |  |
|  |  | 73,200,000 | 73,200,000 | Voght |  |
|  | **1.09** | **73,240,438** | **73,240,438** |  |  |
|  |  | 73,240,438 | 73,240,438 | Akey |  |
|  |  | 73,050,000 | 73,650,000 | Hapmap II |  |
|  | **1.10** | **73,300,000** | **73,300,000** |  |  |
|  |  | 73,050,000 | 73,650,000 | Hapmap II |  |
|  |  | 73,200,000 | 73,300,000 | Voght |  |
|  | **1.11** | **74,856,066** | **74,856,066** |  |  |
|  |  | 74,856,066 | 74,856,066 | Akey |  |
|  |  | 74,800,000 | 74,900,000 | Voght |  |
|  | **1.12** | **75,914,948** | **75,914,948** |  |  |
|  |  | 75,914,948 | 75,914,948 | Akey |  |
|  |  | 75,900,000 | 76,000,000 | Voght |  |
|  | **1.13** | **92,220,000** | **92,300,000** |  |  |
|  |  | 92,220,000 | 93,030,000 | Carlson |  |
|  |  | 92,200,000 | 92,300,000 | Voght |  |
|  |  | 92,220,000 | 93,030,000 | Carlson |  |
|  |  | 92,850,000 | 93,050,000 | Hapmap II |  |
| **2** | **2.01** | **21,300,000** | **21,400,000** |  |  |
|  |  | 21,034,271 | 25,071,304 | Huttley |  |
|  |  | 21,300,000 | 21,400,000 | Voght |  |
|  | **2.02** | **21,650,000** | **21,695,000** |  |  |
|  |  | 21,650,000 | 21,750,000 | Hapmap II |  |
|  |  | 21,034,271 | 25,071,304 | Huttley |  |
|  | **2.03** | **21,700,000** | **21,750,000** |  |  |
|  |  | 21,650,000 | 21,750,000 | Hapmap II |  |
|  |  | 21,034,271 | 25,071,304 | Huttley |  |
|  |  | 21,700,000 | 21,800,000 | Voght |  |
|  | **2.04** | **21,755,000** | **21,795,000** |  |  |
|  |  | 21,034,271 | 25,071,304 | Huttley |  |
|  |  | 21,700,000 | 21,800,000 | Voght |  |
|  | **2.05** | **21,800,000** | **21,800,000** |  |  |
|  |  | 21,034,271 | 25,071,304 | Huttley |  |
|  |  | 21,800,000 | 21,800,000 | Nielsen |  |
|  |  | 21,700,000 | 21,800,000 | Voght |  |
|  | **2.07** | **24,500,000** | **24,500,000** |  |  |
|  |  | 21,034,271 | 25,071,304 | Huttley |  |
|  |  | 24,500,000 | 24,500,000 | Nielsen |  |
|  | **2.08** | **24,650,000** | **24,695,000** |  |  |
|  |  | 24,650,000 | 24,850,000 | Hapmap II |  |
|  |  | 21,034,271 | 25,071,304 | Huttley |  |
|  | **2.09** | **24,700,000** | **24,800,000** |  |  |
|  |  | 24,650,000 | 24,850,000 | Hapmap II |  |
|  |  | 21,034,271 | 25,071,304 | Huttley |  |
|  |  | 24,800,000 | 24,800,000 | Voght |  |
|  | **2.10** | **24,805,000** | **24,850,000** |  |  |
|  |  | 24,650,000 | 24,850,000 | Hapmap II |  |
|  |  | 21,034,271 | 25,071,304 | Huttley |  |
|  | **2.11** | **24,900,000** | **24,900,000** |  |  |
|  |  | 21,034,271 | 25,071,304 | Huttley |  |
|  |  | 24,900,000 | 24,900,000 | Voght |  |
|  | **2.12** | **25,000,000** | **25,000,000** |  |  |
|  |  | 21,034,271 | 25,071,304 | Huttley |  |
|  |  | 24,900,000 | 25,000,000 | Voght |  |
|  | **2.13** | **39,400,000** | **39,400,000** |  |  |
|  |  | 39,400,000 | 39,400,000 | Nielsen |  |
|  |  | 39,131,004 | 39,552,390 | Oleksyk | a |
|  |  | 39,400,000 | 39,500,000 | Voght |  |
|  | **2.14** | **39,405,000** | **39,500,000** |  |  |
|  |  | 39,131,004 | 39,552,390 | Oleksyk | a |
|  |  | 39,400,000 | 39,500,000 | Voght |  |
|  | **2.15** | **71,568,199** | **72,305,000** |  |  |
|  |  | 70,374,253 | 73,327,337 | Huttley |  |
|  |  | 71,568,199 | 72,333,815 | Oleksyk | b |
|  | **2.16** | **72,305,454** | **72,333,815** |  |  |
|  |  | 70,374,253 | 73,327,337 | Huttley |  |
|  |  | 71,568,199 | 72,333,815 | Oleksyk | b |
|  |  | 72,305,454 | 72,927,242 | Sabeti |  |
|  | **2.17** | **72,335,000** | **72,475,000** |  |  |
|  |  | 70,374,253 | 73,327,337 | Huttley |  |
|  |  | 72,305,454 | 72,927,242 | Sabeti |  |
|  | **2.18** | **72,478,112** | **72,478,112** |  |  |
|  |  | 70,374,253 | 73,327,337 | Huttley |  |
|  |  | 71,568,199 | 72,478,112 | Oleksyk | c |
|  |  | 72,305,454 | 72,927,242 | Sabeti |  |
|  | **2.19** | **72,480,000** | **72,695,000** |  |  |
|  |  | 70,374,253 | 73,327,337 | Huttley |  |
|  |  | 72,305,454 | 72,927,242 | Sabeti |  |
|  | **2.20** | **72,700,000** | **72,800,000** |  |  |
|  |  | 70,374,253 | 73,327,337 | Huttley |  |
|  |  | 72,305,454 | 72,927,242 | Sabeti |  |
|  |  | 72,700,000 | 72,800,000 | Voght |  |
|  | **2.21** | **72,805,000** | **72,927,242** |  |  |
|  |  | 70,374,253 | 73,327,337 | Huttley |  |
|  |  | 72,305,454 | 72,927,242 | Sabeti |  |
|  | **2.22** | **73,287,030** | **73,287,030** |  |  |
|  |  | 73,287,030 | 73,287,030 | Akey |  |
|  |  | 70,374,253 | 73,327,337 | Huttley |  |
|  | **2.23** | **73,900,000** | **73,950,000** |  |  |
|  |  | 73,800,000 | 73,950,000 | Hapmap II |  |
|  |  | 73,900,000 | 74,000,000 | Voght |  |
|  | **2.24** | **74,700,000** | **74,700,000** |  |  |
|  |  | 74,700,000 | 74,700,000 | Nielsen |  |
|  |  | 74,452,572 | 74,917,357 | Oleksyk | d |
|  |  | 74,700,000 | 74,800,000 | Voght |  |
|  | **2.25** | **74,705,000** | **74,800,000** |  |  |
|  |  | 74,452,572 | 74,917,357 | Oleksyk | d |
|  |  | 74,700,000 | 74,800,000 | Voght |  |
|  | **2.26** | **83,400,000** | **83,500,000** |  |  |
|  |  | 83,300,000 | 83,550,000 | Hapmap II |  |
|  |  | 83,500,000 | 83,500,000 | Voght |  |
|  | **2.27** | **84,700,000** | **84,700,000** |  |  |
|  |  | 84,540,000 | 84,810,000 | Carlson |  |
|  |  | 84,700,000 | 84,700,000 | Nielsen |  |
|  | **2.28** | **84,900,000** | **84,905,000** |  |  |
|  |  | 84,880,258 | 84,921,349 | Oleksyk | e |
|  |  | 84,900,000 | 85,000,000 | Voght |  |
|  | **2.29** | **84,910,000** | **84,910,000** |  |  |
|  |  | 84,540,000 | 84,910,000 | Carlson |  |
|  |  | 84,880,258 | 84,921,349 | Oleksyk | e |
|  |  | 84,900,000 | 85,000,000 | Voght |  |
|  | **2.30** | **84,915,000** | **84,921,349** |  |  |
|  |  | 84,880,258 | 84,921,349 | Oleksyk | e |
|  |  | 84,900,000 | 85,000,000 | Voght |  |
|  | **2.31** | **95,300,000** | **95,310,019** |  |  |
|  |  | 95,300,000 | 95,940,000 | Carlson |  |
|  |  | 95,298,156 | 95,310,019 | Wang |  |
|  | **2.32** | **95,315,946** | **95,333,477** |  |  |
|  |  | 95,300,000 | 95,940,000 | Carlson |  |
|  |  | 95,315,946 | 95,333,477 | Wang |  |
|  | **2.33** | **95,424,957** | **95,441,810** |  |  |
|  |  | 95,300,000 | 95,940,000 | Carlson |  |
|  |  | 95,424,957 | 95,441,810 | Wang |  |
|  | **2.34** | **95,447,852** | **95,536,581** |  |  |
|  |  | 95,300,000 | 95,940,000 | Carlson |  |
|  |  | 95,447,852 | 95,536,581 | Wang |  |
|  | **2.35** | **95,838,050** | **95,838,050** |  |  |
|  |  | 95,838,050 | 95,838,050 | Akey |  |
|  |  | 95,300,000 | 95,940,000 | Carlson |  |
|  | **2.36** | **96,334,661** | **96,393,283** |  |  |
|  |  | 96,250,000 | 96,750,000 | Hapmap |  |
|  |  | 96,334,661 | 96,393,283 | Oleksyk | f |
|  | **2.37** | **108,300,000** | **108,345,000** |  |  |
|  |  | 108,250,000 | 109,100,000 | Hapmap II |  |
|  |  | 108,300,000 | 108,400,000 | Voght |  |
|  | **2.38** | **108,350,000** | **108,400,000** |  |  |
|  |  | 108,350,000 | 109,120,000 | Carlson |  |
|  |  | 108,250,000 | 109,100,000 | Hapmap II |  |
|  |  | 108,300,000 | 108,400,000 | Voght |  |
|  | **2.39** | **108,405,000** | **108,405,000** |  |  |
|  |  | 108,350,000 | 109,120,000 | Carlson |  |
|  |  | 108,250,000 | 109,100,000 | Hapmap II |  |
|  | **2.40** | **108,408,653** | **108,495,000** |  |  |
|  |  | 108,350,000 | 109,120,000 | Carlson |  |
|  |  | 108,250,000 | 109,100,000 | Hapmap II |  |
|  |  | 108,408,653 | 108,971,124 | Sabeti |  |
|  | **2.41** | **108,495,423** | **108,495,423** |  |  |
|  |  | 108,495,423 | 108,495,423 | Akey |  |
|  |  | 108,350,000 | 109,120,000 | Carlson |  |
|  |  | 108,250,000 | 109,100,000 | Hapmap II |  |
|  |  | 108,408,653 | 108,971,124 | Sabeti |  |
|  | **2.42** | **108,500,000** | **108,600,000** |  |  |
|  |  | 108,350,000 | 109,120,000 | Carlson |  |
|  |  | 108,250,000 | 109,100,000 | Hapmap II |  |
|  |  | 108,408,653 | 108,971,124 | Sabeti |  |
|  |  | 108,600,000 | 108,600,000 | Voght |  |
|  | **2.43** | **108,605,000** | **108,695,000** |  |  |
|  |  | 108,350,000 | 109,120,000 | Carlson |  |
|  |  | 108,250,000 | 109,100,000 | Hapmap II |  |
|  |  | 108,408,653 | 108,971,124 | Sabeti |  |
|  | **2.44** | **108,700,000** | **108,700,000** |  |  |
|  |  | 108,350,000 | 109,120,000 | Carlson |  |
|  |  | 108,250,000 | 109,100,000 | Hapmap II |  |
|  |  | 108,408,653 | 108,971,124 | Sabeti |  |
|  |  | 108,700,000 | 108,700,000 | Voght |  |
|  | **2.45** | **108,705,000** | **108,795,000** |  |  |
|  |  | 108,350,000 | 109,120,000 | Carlson |  |
|  |  | 108,250,000 | 109,100,000 | Hapmap II |  |
|  |  | 108,408,653 | 108,971,124 | Sabeti |  |
|  | **2.46** | **108,800,000** | **108,800,000** |  |  |
|  |  | 108,350,000 | 109,120,000 | Carlson |  |
|  |  | 108,250,000 | 109,100,000 | Hapmap II |  |
|  |  | 108,408,653 | 108,971,124 | Sabeti |  |
|  |  | 108,700,000 | 108,800,000 | Voght |  |
|  | **2.47** | **108,805,000** | **108,971,124** |  |  |
|  |  | 108,350,000 | 109,120,000 | Carlson |  |
|  |  | 108,250,000 | 109,100,000 | Hapmap II |  |
|  |  | 108,408,653 | 108,971,124 | Sabeti |  |
|  | **2.48** | **108,975,000** | **108,995,000** |  |  |
|  |  | 108,350,000 | 109,120,000 | Carlson |  |
|  |  | 108,250,000 | 109,100,000 | Hapmap II |  |
|  | **2.49** | **109,000,000** | **109,100,000** |  |  |
|  |  | 108,350,000 | 109,120,000 | Carlson |  |
|  |  | 108,250,000 | 109,100,000 | Hapmap II |  |
|  |  | 109,100,000 | 109,100,000 | Voght |  |
|  | **2.50** | **121,550,000** | **121,700,000** |  |  |
|  |  | 121,550,000 | 121,700,000 | Hapmap II |  |
|  |  | 121,524,221 | 122,017,375 | Oleksyk | h |
|  | **2.51** | **122,000,000** | **122,000,000** |  |  |
|  |  | 122,000,000 | 122,000,000 | Nielsen |  |
|  |  | 121,524,221 | 122,017,375 | Oleksyk | h |
|  | **2.52** | **132,000,000** | **135,000,000** |  |  |
|  |  | 135,000,000 | 136,550,000 | Hapmap II |  |
|  |  | 135,000,000 | 135,000,000 | Nielsen |  |
|  | **2.53** | **135,500,000** | **135,600,000** |  |  |
|  |  | 135,000,000 | 136,550,000 | Hapmap II |  |
|  |  | 135,600,000 | 135,600,000 | Voght |  |
|  | **2.54** | **135,700,000** | **135,700,000** |  |  |
|  |  | 135,000,000 | 136,550,000 | Hapmap II |  |
|  |  | 135,700,000 | 135,700,000 | Voght |  |
|  | **2.55** | **135,800,000** | **135,800,000** |  |  |
|  |  | 135,000,000 | 136,550,000 | Hapmap II |  |
|  |  | 135,800,000 | 135,800,000 | Voght |  |
|  | **2.56** | **135,900,000** | **135,900,000** |  |  |
|  |  | 135,000,000 | 136,550,000 | Hapmap II |  |
|  |  | 135,900,000 | 135,900,000 | Voght |  |
|  | **2.57** | **136,000,000** | **136,000,000** |  |  |
|  |  | 135,000,000 | 136,550,000 | Hapmap II |  |
|  |  | 136,000,000 | 136,000,000 | Nielsen |  |
|  |  | 136,000,000 | 136,000,000 | Voght |  |
|  | **2.58** | **136,100,000** | **136,100,000** |  |  |
|  |  | 135,000,000 | 136,550,000 | Hapmap II |  |
|  |  | 136,100,000 | 136,100,000 | Voght |  |
|  | **2.59** | **136,200,000** | **136,200,000** |  |  |
|  |  | 135,000,000 | 136,550,000 | Hapmap II |  |
|  |  | 136,200,000 | 136,200,000 | Voght |  |
|  | **2.60** | **136,300,000** | **136,300,000** |  |  |
|  |  | 135,000,000 | 136,550,000 | Hapmap II |  |
|  |  | 136,300,000 | 136,300,000 | Voght |  |
|  | **2.61** | **136,400,000** | **136,400,000** |  |  |
|  |  | 135,000,000 | 136,550,000 | Hapmap II |  |
|  |  | 136,300,000 | 136,400,000 | Voght |  |
|  | **2.62** | **136,500,000** | **136,550,000** |  |  |
|  |  | 135,000,000 | 136,550,000 | Hapmap II |  |
|  |  | 136,500,000 | 136,600,000 | Voght |  |
|  | **2.63** | **136,700,000** | **136,700,000** |  |  |
|  |  | 136,700,000 | 137,250,000 | Hapmap |  |
|  |  | 136,700,000 | 136,700,000 | Voght |  |
|  | **2.64** | **136,800,000** | **136,900,000** |  |  |
|  | **2.65** | **136,800,000** | **136,900,000** |  |  |
|  |  | 136,700,000 | 137,250,000 | Hapmap |  |
|  |  | 136,700,000 | 137,250,000 | Hapmap |  |
|  |  | 136,900,000 | 136,900,000 | Voght |  |
|  |  | 136,900,000 | 136,900,000 | Voght |  |
|  | **2.66** | **137,000,000** | **137,000,000** |  |  |
|  |  | 136,700,000 | 137,250,000 | Hapmap |  |
|  |  | 137,000,000 | 137,250,000 | Hapmap II |  |
|  |  | 136,900,000 | 137,000,000 | Voght |  |
|  | **2.67** | **137,005,000** | **137,095,000** |  |  |
|  |  | 136,700,000 | 137,250,000 | Hapmap |  |
|  |  | 137,000,000 | 137,250,000 | Hapmap II |  |
|  | **2.68** | **137,100,000** | **137,200,000** |  |  |
|  |  | 136,700,000 | 137,250,000 | Hapmap |  |
|  |  | 137,000,000 | 137,250,000 | Hapmap II |  |
|  |  | 137,200,000 | 137,200,000 | Voght |  |
|  | **2.69** | **137,205,000** | **137,250,000** |  |  |
|  |  | 136,700,000 | 137,250,000 | Hapmap |  |
|  |  | 137,000,000 | 137,250,000 | Hapmap II |  |
|  | **2.70** | **148,000,000** | **148,000,000** |  |  |
|  |  | 148,000,000 | 148,000,000 | Nielsen |  |
|  |  | 148,000,000 | 148,100,000 | Voght |  |
|  | **2.71** | **152,700,000** | **152,716,949** |  |  |
|  |  | 152,252,757 | 152,716,949 | Oleksyk | i |
|  |  | 152,700,000 | 152,800,000 | Voght |  |
|  | **2.72** | **158,000,000** | **158,000,000** |  |  |
|  |  | 157,950,000 | 158,050,000 | Hapmap II |  |
|  |  | 158,000,000 | 158,000,000 | Nielsen |  |
|  | **2.73** | **162,855,843** | **162,995,000** |  |  |
|  |  | 162,820,000 | 163,240,000 | Carlson |  |
|  |  | 162,855,843 | 163,362,927 | Oleksyk | k |
|  | **2.74** | **163,000,000** | **163,000,000** |  |  |
|  |  | 162,820,000 | 163,240,000 | Carlson |  |
|  |  | 163,000,000 | 163,000,000 | Nielsen |  |
|  |  | 162,855,843 | 163,362,927 | Oleksyk | k |
|  | **2.75** | **163,005,000** | **163,240,000** |  |  |
|  |  | 162,820,000 | 163,240,000 | Carlson |  |
|  |  | 162,855,843 | 163,362,927 | Oleksyk | k |
|  | **2.76** | **177,390,000** | **177,730,000** |  |  |
|  |  | 177,390,000 | 177,730,000 | Carlson |  |
|  |  | 177,317,730 | 178,285,258 | Sabeti |  |
|  | **2.77** | **177,800,000** | **177,900,000** |  |  |
|  |  | 177,317,730 | 178,285,258 | Sabeti |  |
|  |  | 177,800,000 | 177,900,000 | Voght |  |
|  | **2.78** | **178,250,000** | **178,285,258** |  |  |
|  |  | 178,250,000 | 178,450,000 | Hapmap II |  |
|  |  | 177,317,730 | 178,285,258 | Sabeti |  |
|  | **2.79** | **193,000,000** | **193,000,000** |  |  |
|  |  | 192,950,000 | 193,050,000 | Hapmap II |  |
|  |  | 193,000,000 | 193,000,000 | Nielsen |  |
|  | **2.80** | **194,650,000** | **194,900,000** |  |  |
|  |  | 194,650,000 | 194,990,000 | Carlson |  |
|  |  | 194,650,000 | 194,900,000 | Hapmap II |  |
|  | **2.81** | **219,309,386** | **219,315,616** |  |  |
|  |  | 219,309,386 | 219,315,616 | Oleksyk | l |
|  |  | 219,300,000 | 219,400,000 | Voght |  |
| **3** | **3.01** | **25,800,000** | **25,895,000** |  |  |
|  |  | 25,700,000 | 26,250,000 | Carlson |  |
|  |  | 25,800,000 | 26,300,000 | Hapmap II |  |
|  | **3.02** | **25,900,000** | **26,000,000** |  |  |
|  |  | 25,700,000 | 26,250,000 | Carlson |  |
|  |  | 25,800,000 | 26,300,000 | Hapmap II |  |
|  |  | 26,000,000 | 26,000,000 | Voght |  |
|  | **3.03** | **26,005,000** | **26,095,000** |  |  |
|  |  | 25,700,000 | 26,250,000 | Carlson |  |
|  |  | 25,800,000 | 26,300,000 | Hapmap II |  |
|  | **3.04** | **26,100,000** | **26,100,000** |  |  |
|  |  | 25,700,000 | 26,250,000 | Carlson |  |
|  |  | 25,800,000 | 26,300,000 | Hapmap II |  |
|  |  | 26,000,000 | 26,100,000 | Voght |  |
|  | **3.05** | **26,105,000** | **26,230,000** |  |  |
|  |  | 25,700,000 | 26,250,000 | Carlson |  |
|  |  | 25,800,000 | 26,300,000 | Hapmap II |  |
|  | **3.06** | **26,230,802** | **26,239,053** |  |  |
|  |  | 25,700,000 | 26,250,000 | Carlson |  |
|  |  | 25,800,000 | 26,300,000 | Hapmap II |  |
|  |  | 26,230,802 | 26,239,053 | Sabeti |  |
|  | **3.07** | **26,240,000** | **26,250,000** |  |  |
|  |  | 25,700,000 | 26,250,000 | Carlson |  |
|  |  | 25,800,000 | 26,300,000 | Hapmap II |  |
|  | **3.08** | **46,335,592** | **46,335,592** |  |  |
|  |  | 46,335,592 | 46,335,592 | Akey |  |
|  |  | 46,286,786 | 46,476,846 | Oleksyk | c |
|  | **3.09** | **49,400,000** | **49,500,000** |  |  |
|  |  | 49,300,000 | 49,650,000 | Hapmap II |  |
|  |  | 49,400,000 | 49,500,000 | Voght |  |
|  | **3.10** | **49,600,000** | **49,650,000** |  |  |
|  |  | 49,300,000 | 49,650,000 | Hapmap II |  |
|  |  | 49,600,000 | 49,700,000 | Voght |  |
|  | **3.11** | **50,600,000** | **50,700,000** |  |  |
|  |  | 50,570,145 | 51,432,075 | Oleksyk | d |
|  |  | 50,700,000 | 50,700,000 | Voght |  |
|  | **3.12** | **50,800,000** | **50,800,000** |  |  |
|  |  | 50,570,145 | 51,432,075 | Oleksyk | d |
|  |  | 50,700,000 | 50,800,000 | Voght |  |
|  | **3.13** | **56,550,000** | **56,600,000** |  |  |
|  |  | 56,550,000 | 56,700,000 | Hapmap II |  |
|  |  | 56,600,000 | 56,600,000 | Voght |  |
|  | **3.14** | **56,700,000** | **56,700,000** |  |  |
|  |  | 56,550,000 | 56,700,000 | Hapmap II |  |
|  |  | 56,600,000 | 56,700,000 | Voght |  |
|  | **3.15** | **90,250,000** | **90,300,000** |  |  |
|  |  | 90,250,000 | 90,750,000 | Hapmap |  |
|  |  | 90,150,000 | 90,300,000 | Hapmap II |  |
|  | **3.16** | **98,750,000** | **98,828,098** |  |  |
|  |  | 98,750,000 | 99,250,000 | Hapmap |  |
|  |  | 98,058,107 | 98,828,098 | Oleksyk | f |
|  | **3.17** | **140,600,000** | **140,600,000** |  |  |
|  |  | 140,600,000 | 140,700,000 | Hapmap II |  |
|  |  | 140,500,000 | 140,600,000 | Voght |  |
| **4** | **4.01** | **33,200,000** | **33,300,000** |  |  |
|  |  | 32,930,000 | 33,560,000 | Carlson |  |
|  |  | 33,200,000 | 33,300,000 | Voght |  |
|  | **4.02** | **34,000,000** | **34,195,000** |  |  |
|  |  | 34,000,000 | 34,500,000 | Hapmap |  |
|  |  | 33,600,000 | 34,700,000 | Hapmap II |  |
|  | **4.03** | **34,200,000** | **34,400,000** |  |  |
|  |  | 34,000,000 | 34,500,000 | Hapmap |  |
|  |  | 33,600,000 | 34,700,000 | Hapmap II |  |
|  |  | 34,400,000 | 34,400,000 | Voght |  |
|  | **4.05** | **34,405,000** | **34,495,000** |  |  |
|  |  | 34,000,000 | 34,500,000 | Hapmap |  |
|  |  | 33,600,000 | 34,700,000 | Hapmap II |  |
|  | **4.06** | **34,500,000** | **34,500,000** |  |  |
|  |  | 34,000,000 | 34,500,000 | Hapmap |  |
|  |  | 33,600,000 | 34,700,000 | Hapmap II |  |
|  |  | 34,500,000 | 34,500,000 | Voght |  |
|  | **4.07** | **34,600,000** | **34,600,000** |  |  |
|  |  | 33,600,000 | 34,700,000 | Hapmap II |  |
|  |  | 34,500,000 | 34,600,000 | Voght |  |
|  | **4.08** | **41,670,000** | **41,700,000** |  |  |
|  |  | 41,670,000 | 42,050,000 | Carlson |  |
|  |  | 41,600,000 | 41,700,000 | Voght |  |
|  | **4.09** | **41,800,000** | **41,895,000** |  |  |
|  |  | 41,670,000 | 42,050,000 | Carlson |  |
|  |  | 41,800,000 | 41,900,000 | Voght |  |
|  | **4.10** | **41,900,000** | **41,980,000** |  |  |
|  |  | 41,670,000 | 42,050,000 | Carlson |  |
|  |  | 41,900,000 | 42,050,000 | Hapmap II |  |
|  |  | 41,900,000 | 42,000,000 | Voght |  |
|  | **4.11** | **41,984,060** | **41,989,630** |  |  |
|  |  | 41,670,000 | 42,050,000 | Carlson |  |
|  |  | 41,900,000 | 42,050,000 | Hapmap II |  |
|  |  | 41,984,060 | 41,989,630 | Sabeti |  |
|  |  | 41,900,000 | 42,000,000 | Voght |  |
|  | **4.12** | **41,990,000** | **42,000,000** |  |  |
|  |  | 41,670,000 | 42,050,000 | Carlson |  |
|  |  | 41,900,000 | 42,050,000 | Hapmap II |  |
|  |  | 42,000,000 | 42,000,000 | Voght |  |
|  | **4.14** | **42,005,000** | **42,050,000** |  |  |
|  |  | 41,670,000 | 42,050,000 | Carlson |  |
|  |  | 41,900,000 | 42,050,000 | Hapmap II |  |
|  | **4.15** | **48,674,033** | **48,700,000** |  |  |
|  |  | 48,600,000 | 48,700,000 | Voght |  |
|  |  | 48,674,033 | 48,704,191 | Wang |  |
|  | **4.16** | **56,200,000** | **56,250,000** |  |  |
|  |  | 56,100,000 | 56,250,000 | Hapmap II |  |
|  |  | 56,200,000 | 56,300,000 | Voght |  |
|  | **4.17** | **100,700,000** | **100,800,000** |  |  |
|  |  | 100,000,000 | 101,000,000 | Hapmap II |  |
|  |  | 100,700,000 | 100,800,000 | Voght |  |
|  | **4.18** | **144,300,000** | **144,400,000** |  |  |
|  |  | 144,100,000 | 144,550,000 | Hapmap II |  |
|  |  | 144,300,000 | 144,400,000 | Voght |  |
|  | **4.19** | **144,500,000** | **144,550,000** |  |  |
|  |  | 144,100,000 | 144,550,000 | Hapmap II |  |
|  |  | 144,500,000 | 144,600,000 | Voght |  |
|  | **4.20** | **148,880,000** | **148,895,000** |  |  |
|  |  | 148,880,000 | 149,280,000 | Carlson |  |
|  |  | 148,835,482 | 149,328,142 | Oleksyk | d |
|  | **4.21** | **148,900,000** | **148,900,000** |  |  |
|  |  | 148,880,000 | 149,280,000 | Carlson |  |
|  |  | 148,835,482 | 149,328,142 | Oleksyk | d |
|  |  | 148,800,000 | 148,900,000 | Voght |  |
|  | **4.22** | **148,905,000** | **149,280,000** |  |  |
|  |  | 148,880,000 | 149,280,000 | Carlson |  |
|  |  | 148,835,482 | 149,328,142 | Oleksyk | d |
|  | **4.23** | **151,985,548** | **152,514,276** |  |  |
|  |  | 151,985,548 | 152,954,145 | Oleksyk | e |
|  |  | 151,763,438 | 152,514,276 | Wang |  |
|  | **4.24** | **152,900,000** | **152,954,145** |  |  |
|  |  | 151,985,548 | 152,954,145 | Oleksyk | e |
|  |  | 152,900,000 | 153,000,000 | Voght |  |
|  | **4.25** | **159,100,000** | **159,100,000** |  |  |
|  |  | 158,900,000 | 159,100,000 | Hapmap II |  |
|  |  | 159,100,000 | 159,200,000 | Voght |  |
| **5** | **5.01** | **64,900,000** | **64,900,000** |  |  |
|  |  | 64,850,000 | 65,100,000 | Hapmap II |  |
|  |  | 64,900,000 | 64,900,000 | Voght |  |
|  | **5.02** | **65,000,000** | **65,000,000** |  |  |
|  |  | 64,850,000 | 65,100,000 | Hapmap II |  |
|  |  | 64,900,000 | 65,000,000 | Voght |  |
|  | **5.03** | **93,200,000** | **93,300,000** |  |  |
|  |  | 92,937,508 | 93,775,891 | Oleksyk | c |
|  |  | 93,300,000 | 93,300,000 | Voght |  |
|  | **5.04** | **93,400,000** | **93,400,000** |  |  |
|  |  | 92,937,508 | 93,775,891 | Oleksyk | c |
|  |  | 93,400,000 | 93,400,000 | Voght |  |
|  | **5.05** | **93,500,000** | **93,500,000** |  |  |
|  |  | 92,937,508 | 93,775,891 | Oleksyk | c |
|  |  | 93,400,000 | 93,500,000 | Voght |  |
|  | **5.06** | **110,150,000** | **110,200,000** |  |  |
|  |  | 110,150,000 | 110,300,000 | Hapmap II |  |
|  |  | 110,200,000 | 110,200,000 | Voght |  |
|  | **5.07** | **110,300,000** | **110,300,000** |  |  |
|  |  | 110,150,000 | 110,300,000 | Hapmap II |  |
|  |  | 110,200,000 | 110,300,000 | Voght |  |
|  | **5.08** | **117,381,470** | **117,679,927** |  |  |
|  |  | 117,360,000 | 117,700,000 | Carlson |  |
|  |  | 117,381,470 | 117,679,927 | Sabeti |  |
|  | **5.09** | **142,119,542** | **142,125,869** |  |  |
|  |  | 141,975,961 | 142,234,863 | Oleksyk | f |
|  |  | 142,119,542 | 142,125,869 | Sabeti |  |
|  | **5.10** | **142,200,000** | **142,234,863** |  |  |
|  |  | 141,975,961 | 142,234,863 | Oleksyk | f |
|  |  | 142,200,000 | 142,300,000 | Voght |  |
| **6** | **6.01** | **27,526,506** | **27,548,858** |  |  |
|  |  | 27,500,000 | 27,600,000 | Voght |  |
|  |  | 27,526,506 | 27,548,858 | Wang |  |
|  | **6.02** | **31,767,710** | **31,773,431** |  |  |
|  |  | 31,457,335 | 32,721,719 | Huttley |  |
|  |  | 31,767,710 | 31,773,431 | Oleksyk | d |
|  | **6.03** | **48,300,000** | **48,300,000** |  |  |
|  |  | 48,300,000 | 48,400,000 | Hapmap II |  |
|  |  | 48,200,000 | 48,300,000 | Voght |  |
|  | **6.04** | **75,100,000** | **75,200,000** |  |  |
|  |  | 75,060,000 | 75,360,000 | Carlson |  |
|  |  | 75,100,000 | 75,200,000 | Voght |  |
|  | **6.05** | **84,500,000** | **84,795,000** |  |  |
|  |  | 84,500,000 | 84,800,000 | Carlson |  |
|  |  | 84,476,512 | 84,904,563 | Oleksyk | h |
|  | **6.06** | **84,800,000** | **84,800,000** |  |  |
|  |  | 84,500,000 | 84,800,000 | Carlson |  |
|  |  | 84,800,000 | 85,000,000 | Hapmap II |  |
|  |  | 84,476,512 | 84,904,563 | Oleksyk | h |
|  | **6.07** | **84,805,000** | **84,904,563** |  |  |
|  |  | 84,800,000 | 85,000,000 | Hapmap II |  |
|  |  | 84,476,512 | 84,904,563 | Oleksyk | h |
|  | **6.08** | **127,100,000** | **127,130,000** |  |  |
|  |  | 126,650,000 | 127,130,000 | Carlson |  |
|  |  | 127,100,000 | 127,200,000 | Voght |  |
|  | **6.09** | **130,550,000** | **130,600,000** |  |  |
|  |  | 130,550,000 | 130,650,000 | Hapmap II |  |
|  |  | 130,600,000 | 130,600,000 | Voght |  |
|  | **6.10** | **140,500,000** | **140,600,000** |  |  |
|  |  | 140,500,000 | 141,000,000 | Hapmap |  |
|  |  | 140,500,000 | 140,600,000 | Voght |  |
| **7** | **7.01** | **20,100,000** | **20,200,000** |  |  |
|  |  | 20,100,000 | 20,250,000 | Hapmap II |  |
|  |  | 20,100,000 | 20,200,000 | Voght |  |
|  | **7.02** | **38,957,855** | **38,957,855** |  |  |
|  |  | 38,957,855 | 38,957,855 | Akey |  |
|  |  | 38,034,744 | 44,738,233 | Huttley |  |
|  | **7.03** | **39,026,383** | **39,026,383** |  |  |
|  |  | 39,026,383 | 39,026,383 | Akey |  |
|  |  | 38,034,744 | 44,738,233 | Huttley |  |
|  | **7.04** | **68,500,000** | **68,600,000** |  |  |
|  |  | 68,500,000 | 68,600,000 | Voght |  |
|  |  | 68,476,436 | 69,331,674 | Wang |  |
|  | **7.06** | **98,460,000** | **98,595,000** |  |  |
|  |  | 98,460,000 | 99,080,000 | Carlson |  |
|  |  | 98,290,168 | 99,123,455 | Oleksyk | b |
|  | **7.07** | **98,600,000** | **98,700,000** |  |  |
|  |  | 98,460,000 | 99,080,000 | Carlson |  |
|  |  | 98,290,168 | 99,123,455 | Oleksyk | b |
|  |  | 98,600,000 | 98,700,000 | Voght |  |
|  | **7.08** | **98,705,000** | **99,080,000** |  |  |
|  |  | 98,460,000 | 99,080,000 | Carlson |  |
|  |  | 98,290,168 | 99,123,455 | Oleksyk | b |
|  | **7.09** | **111,750,000** | **111,800,000** |  |  |
|  |  | 111,750,000 | 111,950,000 | Hapmap II |  |
|  |  | 111,800,000 | 111,800,000 | Voght |  |
|  | **7.10** | **111,900,000** | **111,900,000** |  |  |
|  |  | 111,750,000 | 111,950,000 | Hapmap II |  |
|  |  | 111,900,000 | 111,900,000 | Voght |  |
|  | **7.11** | **119,900,000** | **120,000,000** |  |  |
|  |  | 119,456,534 | 128,796,639 | Huttley |  |
|  |  | 119,900,000 | 120,000,000 | Voght |  |
|  | **7.12** | **121,722,275** | **121,741,319** |  |  |
|  |  | 119,456,534 | 128,796,639 | Huttley |  |
|  |  | 121,722,275 | 121,741,319 | Oleksyk | e |
|  | **7.13** | **124,100,000** | **124,120,000** |  |  |
|  |  | 124,100,000 | 124,250,000 | Hapmap II |  |
|  |  | 119,456,534 | 128,796,639 | Huttley |  |
|  | **7.14** | **124,124,434** | **124,250,000** |  |  |
|  |  | 124,100,000 | 124,250,000 | Hapmap II |  |
|  |  | 119,456,534 | 128,796,639 | Huttley |  |
|  |  | 124,124,434 | 124,339,791 | Wang |  |
|  | **7.15** | **124,255,000** | **124,339,791** |  |  |
|  |  | 119,456,534 | 128,796,639 | Huttley |  |
|  |  | 124,124,434 | 124,339,791 | Wang |  |
|  | **7.16** | **126,400,000** | **126,500,000** |  |  |
|  |  | 119,456,534 | 128,796,639 | Huttley |  |
|  |  | 126,400,000 | 126,500,000 | Voght |  |
|  | **7.17** | **126,700,000** | **126,800,000** |  |  |
|  |  | 119,456,534 | 128,796,639 | Huttley |  |
|  |  | 126,700,000 | 126,800,000 | Voght |  |
|  | **7.18** | **142,030,000** | **142,150,000** |  |  |
|  |  | 142,030,000 | 142,360,000 | Carlson |  |
|  |  | 141,500,000 | 142,150,000 | Hapmap II |  |
| **8** | **8.01** | **9,500,000** | **9,500,000** |  |  |
|  |  | 9,500,000 | 9,900,000 | Hapmap II |  |
|  |  | 9,500,000 | 9,500,000 | Voght |  |
|  | **8.02** | **9,600,000** | **9,600,000** |  |  |
|  |  | 9,500,000 | 9,900,000 | Hapmap II |  |
|  |  | 9,600,000 | 9,600,000 | Voght |  |
|  | **8.03** | **9,700,000** | **9,700,000** |  |  |
|  |  | 9,500,000 | 9,900,000 | Hapmap II |  |
|  |  | 9,600,000 | 9,700,000 | Voght |  |
|  | **8.04** | **11,200,000** | **11,300,000** |  |  |
|  |  | 11,200,000 | 11,300,000 | Hapmap II |  |
|  |  | 11,200,000 | 11,300,000 | Voght |  |
|  | **8.05** | **42,500,000** | **42,600,000** |  |  |
|  |  | 42,184,151 | 43,074,597 | Oleksyk | b |
|  |  | 42,500,000 | 42,600,000 | Voght |  |
|  | **8.06** | **50,150,493** | **50,150,619** |  |  |
|  |  | 50,100,000 | 50,200,000 | Voght |  |
|  |  | 50,150,493 | 50,150,619 | Wang |  |
|  | **8.07** | **50,300,000** | **50,300,000** |  |  |
|  |  | 50,300,000 | 50,400,000 | Hapmap II |  |
|  |  | 50,200,000 | 50,300,000 | Voght |  |
|  | **8.08** | **50,600,000** | **50,700,000** |  |  |
|  |  | 50,580,000 | 51,170,000 | Carlson |  |
|  |  | 50,700,000 | 50,700,000 | Voght |  |
|  | **8.09** | **50,800,000** | **50,800,000** |  |  |
|  |  | 50,580,000 | 51,170,000 | Carlson |  |
|  |  | 50,700,000 | 50,800,000 | Voght |  |
|  | **8.10** | **50,874,299** | **51,045,000** |  |  |
|  |  | 50,580,000 | 51,170,000 | Carlson |  |
|  |  | 50,874,299 | 51,756,638 | Wang |  |
|  | **8.11** | **51,050,000** | **51,170,000** |  |  |
|  |  | 50,580,000 | 51,170,000 | Carlson |  |
|  |  | 51,050,000 | 52,150,000 | Hapmap II |  |
|  |  | 50,874,299 | 51,756,638 | Wang |  |
|  | **8.12** | **51,175,000** | **51,195,000** |  |  |
|  |  | 51,050,000 | 52,150,000 | Hapmap II |  |
|  |  | 50,874,299 | 51,756,638 | Wang |  |
|  | **8.13** | **51,200,000** | **51,300,000** |  |  |
|  |  | 51,050,000 | 52,150,000 | Hapmap II |  |
|  |  | 51,300,000 | 51,300,000 | Voght |  |
|  |  | 50,874,299 | 51,756,638 | Wang |  |
|  | **8.15** | **51,305,000** | **51,395,000** |  |  |
|  |  | 51,050,000 | 52,150,000 | Hapmap II |  |
|  |  | 50,874,299 | 51,756,638 | Wang |  |
|  | **8.16** | **51,400,000** | **51,400,000** |  |  |
|  |  | 51,050,000 | 52,150,000 | Hapmap II |  |
|  |  | 51,400,000 | 51,400,000 | Voght |  |
|  |  | 50,874,299 | 51,756,638 | Wang |  |
|  | **8.17** | **51,405,000** | **51,495,000** |  |  |
|  |  | 51,050,000 | 52,150,000 | Hapmap II |  |
|  |  | 50,874,299 | 51,756,638 | Wang |  |
|  | **8.18** | **51,500,000** | **51,500,000** |  |  |
|  |  | 51,050,000 | 52,150,000 | Hapmap II |  |
|  |  | 51,500,000 | 51,500,000 | Voght |  |
|  |  | 50,874,299 | 51,756,638 | Wang |  |
|  | **8.19** | **51,505,000** | **51,595,000** |  |  |
|  |  | 51,050,000 | 52,150,000 | Hapmap II |  |
|  |  | 50,874,299 | 51,756,638 | Wang |  |
|  | **8.20** | **51,600,000** | **51,600,000** |  |  |
|  |  | 51,050,000 | 52,150,000 | Hapmap II |  |
|  |  | 51,600,000 | 51,600,000 | Voght |  |
|  |  | 50,874,299 | 51,756,638 | Wang |  |
|  | **8.21** | **51,605,000** | **51,695,000** |  |  |
|  |  | 51,050,000 | 52,150,000 | Hapmap II |  |
|  |  | 50,874,299 | 51,756,638 | Wang |  |
|  | **8.22** | **51,700,000** | **51,700,000** |  |  |
|  |  | 51,050,000 | 52,150,000 | Hapmap II |  |
|  |  | 51,700,000 | 51,700,000 | Voght |  |
|  |  | 50,874,299 | 51,756,638 | Wang |  |
|  | **8.23** | **51,705,000** | **51,756,638** |  |  |
|  |  | 51,050,000 | 52,150,000 | Hapmap II |  |
|  |  | 50,874,299 | 51,756,638 | Wang |  |
|  | **8.24** | **51,800,000** | **51,800,000** |  |  |
|  |  | 51,050,000 | 52,150,000 | Hapmap II |  |
|  |  | 51,800,000 | 51,800,000 | Voght |  |
|  | **8.25** | **51,900,000** | **51,900,000** |  |  |
|  |  | 51,050,000 | 52,150,000 | Hapmap II |  |
|  |  | 51,900,000 | 51,900,000 | Voght |  |
|  | **8.26** | **52,000,000** | **52,000,000** |  |  |
|  |  | 51,050,000 | 52,150,000 | Hapmap II |  |
|  |  | 51,900,000 | 52,000,000 | Voght |  |
|  | **8.27** | **52,600,000** | **52,600,000** |  |  |
|  |  | 52,600,000 | 53,050,000 | Hapmap II |  |
|  |  | 52,600,000 | 52,600,000 | Voght |  |
|  | **8.28** | **52,700,000** | **52,700,000** |  |  |
|  |  | 52,600,000 | 53,050,000 | Hapmap II |  |
|  |  | 52,600,000 | 52,700,000 | Voght |  |
|  | **8.29** | **52,800,000** | **52,900,000** |  |  |
|  |  | 52,600,000 | 53,050,000 | Hapmap II |  |
|  |  | 52,900,000 | 52,900,000 | Voght |  |
|  | **8.30** | **53,000,000** | **53,000,000** |  |  |
|  |  | 52,600,000 | 53,050,000 | Hapmap II |  |
|  |  | 52,900,000 | 53,000,000 | Voght |  |
|  | **8.31** | **112,000,000** | **112,050,000** |  |  |
|  |  | 111,900,000 | 112,050,000 | Hapmap II |  |
|  |  | 112,000,000 | 112,100,000 | Voght |  |
|  | **8.32** | **113,200,000** | **113,300,000** |  |  |
|  |  | 113,200,000 | 113,300,000 | Voght |  |
|  |  | 113,191,740 | 114,405,825 | Wang |  |
|  | **8.33** | **113,400,000** | **113,500,000** |  |  |
|  |  | 113,500,000 | 113,500,000 | Voght |  |
|  |  | 113,191,740 | 114,405,825 | Wang |  |
|  | **8.34** | **113,600,000** | **113,600,000** |  |  |
|  |  | 113,500,000 | 113,600,000 | Voght |  |
|  |  | 113,191,740 | 114,405,825 | Wang |  |
|  | **8.35** | **113,700,000** | **113,800,000** |  |  |
|  |  | 113,700,000 | 113,800,000 | Voght |  |
|  |  | 113,191,740 | 114,405,825 | Wang |  |
|  | **8.36** | **114,100,000** | **114,200,000** |  |  |
|  |  | 114,100,000 | 114,200,000 | Voght |  |
|  |  | 113,191,740 | 114,405,825 | Wang |  |
|  | **8.37** | **114,400,000** | **114,405,825** |  |  |
|  |  | 114,400,000 | 114,500,000 | Voght |  |
|  |  | 113,191,740 | 114,405,825 | Wang |  |
| **9** | **9.01** | **12,600,000** | **12,600,000** |  |  |
|  |  | 12,600,000 | 12,700,000 | Hapmap II |  |
|  |  | 12,600,000 | 12,600,000 | Voght |  |
|  | **9.02** | **12,700,000** | **12,700,000** |  |  |
|  |  | 12,600,000 | 12,700,000 | Hapmap II |  |
|  |  | 12,600,000 | 12,700,000 | Voght |  |
|  | **9.03** | **24,350,000** | **24,400,000** |  |  |
|  |  | 24,350,000 | 24,450,000 | Hapmap II |  |
|  |  | 24,400,000 | 24,400,000 | Voght |  |
|  | **9.04** | **96,333,572** | **96,333,572** |  |  |
|  |  | 96,333,572 | 96,333,572 | Akey |  |
|  |  | 96,180,190 | 96,333,949 | Oleksyk | e |
|  | **9.05** | **97,267,362** | **97,267,362** |  |  |
|  |  | 97,267,362 | 97,267,362 | Akey |  |
|  |  | 97,200,000 | 97,300,000 | Voght |  |
|  | **9.06** | **107,136,294** | **107,179,093** |  |  |
|  |  | 107,136,294 | 107,179,093 | Oleksyk | g |
|  |  | 107,100,000 | 107,200,000 | Voght |  |
| **10** | **10.01** | **2,986,576** | **2,988,247** |  |  |
|  |  | 2,950,000 | 3,100,000 | Hapmap II |  |
|  |  | 2,986,576 | 2,988,247 | Sabeti |  |
|  | **10.02** | **31,800,000** | **31,800,000** |  |  |
|  |  | 31,733,523 | 31,842,656 | Oleksyk | b |
|  |  | 31,700,000 | 31,800,000 | Voght |  |
|  | **10.03** | **33,100,000** | **33,175,796** |  |  |
|  |  | 33,100,000 | 33,200,000 | Voght |  |
|  |  | 32,860,657 | 33,175,796 | Wang |  |
|  | **10.04** | **74,336,071** | **74,336,071** |  |  |
|  |  | 74,000,000 | 75,250,000 | Hapmap |  |
|  |  | 74,336,071 | 74,664,519 | Oleksyk | g |
|  | **10.05** | **74,340,000** | **74,664,519** |  |  |
|  |  | 74,340,000 | 75,090,000 | Carlson |  |
|  |  | 74,000,000 | 75,250,000 | Hapmap |  |
|  |  | 74,336,071 | 74,664,519 | Oleksyk | g |
|  | **10.06** | **74,665,000** | **74,795,000** |  |  |
|  |  | 74,340,000 | 75,090,000 | Carlson |  |
|  |  | 74,000,000 | 75,250,000 | Hapmap |  |
|  | **10.07** | **74,800,000** | **74,900,000** |  |  |
|  |  | 74,340,000 | 75,090,000 | Carlson |  |
|  |  | 74,000,000 | 75,250,000 | Hapmap |  |
|  |  | 74,800,000 | 74,900,000 | Voght |  |
|  | **10.08** | **74,905,000** | **75,090,000** |  |  |
|  |  | 74,340,000 | 75,090,000 | Carlson |  |
|  |  | 74,000,000 | 75,250,000 | Hapmap |  |
|  | **10.09** | **75,102,484** | **75,224,380** |  |  |
|  |  | 74,000,000 | 75,250,000 | Hapmap |  |
|  |  | 75,102,484 | 75,224,380 | Wang |  |
|  | **10.10** | **75,226,127** | **75,250,000** |  |  |
|  |  | 74,000,000 | 75,250,000 | Hapmap |  |
|  |  | 75,226,127 | 75,255,181 | Wang |  |
|  | **10.11** | **75,500,000** | **75,600,000** |  |  |
|  |  | 75,500,000 | 75,600,000 | Voght |  |
|  |  | 75,255,568 | 75,813,663 | Wang |  |
|  | **10.12** | **93,548,281** | **93,548,281** |  |  |
|  |  | 93,548,281 | 93,548,281 | Akey |  |
|  |  | 93,473,959 | 93,715,421 | Wang |  |
|  | **10.13** | **93,600,000** | **93,700,000** |  |  |
|  |  | 93,600,000 | 93,700,000 | Voght |  |
|  |  | 93,473,959 | 93,715,421 | Wang |  |
|  | **10.14** | **100,500,000** | **100,600,000** |  |  |
|  |  | 100,600,000 | 100,600,000 | Voght |  |
|  |  | 99,883,462 | 100,660,206 | Wang |  |
|  | **10.15** | **109,700,000** | **109,800,000** |  |  |
|  |  | 109,650,000 | 109,800,000 | Hapmap II |  |
|  |  | 109,700,000 | 109,800,000 | Voght |  |
| **11** | **11.01** | **25,300,000** | **25,400,000** |  |  |
|  |  | 25,250,000 | 25,600,000 | Hapmap II |  |
|  |  | 25,300,000 | 25,400,000 | Voght |  |
|  | **11.02** | **38,400,000** | **38,500,000** |  |  |
|  |  | 38,400,000 | 38,750,000 | Hapmap II |  |
|  |  | 38,400,000 | 38,500,000 | Voght |  |
|  | **11.04** | **55,554,471** | **55,579,355** |  |  |
|  |  | 55,340,000 | 55,750,000 | Carlson |  |
|  |  | 55,554,471 | 55,579,355 | Wang |  |
|  | **11.05** | **55,617,360** | **55,618,289** |  |  |
|  |  | 55,340,000 | 55,750,000 | Carlson |  |
|  |  | 55,617,360 | 55,618,289 | Wang |  |
|  | **11.06** | **55,700,000** | **55,750,000** |  |  |
|  |  | 55,340,000 | 55,750,000 | Carlson |  |
|  |  | 55,700,000 | 55,800,000 | Voght |  |
|  | **11.07** | **66,900,000** | **66,981,170** |  |  |
|  |  | 66,433,942 | 66,981,170 | Oleksyk | d |
|  |  | 66,900,000 | 67,000,000 | Voght |  |
|  | **11.08** | **119,600,000** | **119,700,000** |  |  |
|  |  | 119,550,000 | 119,700,000 | Hapmap II |  |
|  |  | 119,600,000 | 119,700,000 | Voght |  |
| **12** | **12.01** | **19,244,750** | **19,300,000** |  |  |
|  |  | 19,244,750 | 19,498,561 | Oleksyk | b |
|  |  | 19,200,000 | 19,300,000 | Voght |  |
|  | **12.02** | **21,800,000** | **21,900,000** |  |  |
|  |  | 21,800,000 | 21,900,000 | Hapmap II |  |
|  |  | 21,800,000 | 21,900,000 | Voght |  |
|  | **12.03** | **39,666,569** | **39,700,000** |  |  |
|  |  | 39,666,569 | 39,734,697 | Oleksyk | c |
|  |  | 39,600,000 | 39,700,000 | Voght |  |
|  | **12.04** | **39,800,000** | **39,900,000** |  |  |
|  |  | 39,800,000 | 39,950,000 | Hapmap II |  |
|  |  | 39,800,000 | 39,900,000 | Voght |  |
|  | **12.05** | **43,900,000** | **44,000,000** |  |  |
|  |  | 43,496,926 | 44,461,736 | Oleksyk | d |
|  |  | 43,900,000 | 44,000,000 | Voght |  |
|  | **12.06** | **45,350,000** | **45,400,000** |  |  |
|  |  | 45,350,000 | 45,500,000 | Hapmap II |  |
|  |  | 45,300,000 | 45,400,000 | Voght |  |
|  | **12.07** | **75,300,000** | **75,400,000** |  |  |
|  |  | 75,300,000 | 75,400,000 | Hapmap II |  |
|  |  | 75,300,000 | 75,400,000 | Voght |  |
|  | **12.08** | **78,000,000** | **78,000,000** |  |  |
|  |  | 78,000,000 | 78,650,000 | Hapmap II |  |
|  |  | 78,000,000 | 78,000,000 | Voght |  |
|  | **12.09** | **78,100,000** | **78,100,000** |  |  |
|  |  | 78,000,000 | 78,650,000 | Hapmap II |  |
|  |  | 78,100,000 | 78,100,000 | Voght |  |
|  | **12.10** | **78,199,208** | **78,199,208** |  |  |
|  |  | 78,000,000 | 78,650,000 | Hapmap II |  |
|  |  | 78,199,208 | 79,337,593 | Oleksyk | f |
|  | **12.11** | **78,199,208** | **78,200,000** |  |  |
|  |  | 78,000,000 | 78,650,000 | Hapmap II |  |
|  |  | 78,199,208 | 79,337,593 | Oleksyk | f |
|  |  | 78,200,000 | 78,200,000 | Voght |  |
|  | **12.12** | **78,205,000** | **78,295,000** |  |  |
|  |  | 78,000,000 | 78,650,000 | Hapmap II |  |
|  |  | 78,199,208 | 79,337,593 | Oleksyk | f |
|  | **12.13** | **78,300,000** | **78,300,000** |  |  |
|  |  | 78,000,000 | 78,650,000 | Hapmap II |  |
|  |  | 78,199,208 | 79,337,593 | Oleksyk | f |
|  |  | 78,200,000 | 78,300,000 | Voght |  |
|  | **12.14** | **78,305,000** | **78,395,000** |  |  |
|  |  | 78,000,000 | 78,650,000 | Hapmap II |  |
|  |  | 78,199,208 | 79,337,593 | Oleksyk | f |
|  | **12.15** | **78,400,000** | **78,500,000** |  |  |
|  |  | 78,000,000 | 78,650,000 | Hapmap II |  |
|  |  | 78,199,208 | 79,337,593 | Oleksyk | f |
|  |  | 78,500,000 | 78,500,000 | Voght |  |
|  | **12.16** | **78,505,000** | **78,595,000** |  |  |
|  |  | 78,000,000 | 78,650,000 | Hapmap II |  |
|  |  | 78,199,208 | 79,337,593 | Oleksyk | f |
|  | **12.17** | **78,600,000** | **78,600,000** |  |  |
|  |  | 78,000,000 | 78,650,000 | Hapmap II |  |
|  |  | 78,199,208 | 79,337,593 | Oleksyk | f |
|  |  | 78,500,000 | 78,600,000 | Voght |  |
|  | **12.18** | **78,605,000** | **78,650,000** |  |  |
|  |  | 78,000,000 | 78,650,000 | Hapmap II |  |
|  |  | 78,199,208 | 79,337,593 | Oleksyk | f |
|  | **12.19** | **78,757,457** | **78,827,321** |  |  |
|  |  | 78,199,208 | 79,337,593 | Oleksyk | f |
|  |  | 78,757,457 | 78,827,321 | Sabeti |  |
|  | **12.20** | **79,200,000** | **79,300,000** |  |  |
|  |  | 78,199,208 | 79,337,593 | Oleksyk | f |
|  |  | 79,200,000 | 79,300,000 | Voght |  |
|  | **12.21** | **86,894,226** | **87,360,000** |  |  |
|  |  | 86,840,000 | 87,360,000 | Carlson |  |
|  |  | 86,894,226 | 88,246,057 | Oleksyk | h |
|  | **12.22** | **87,400,000** | **87,485,000** |  |  |
|  |  | 86,894,226 | 88,246,057 | Oleksyk | h |
|  |  | 87,400,000 | 87,500,000 | Voght |  |
|  | **12.23** | **87,490,000** | **87,500,000** |  |  |
|  |  | 87,490,000 | 87,840,000 | Carlson |  |
|  |  | 86,894,226 | 88,246,057 | Oleksyk | h |
|  |  | 87,400,000 | 87,500,000 | Voght |  |
|  | **12.24** | **87,505,000** | **87,840,000** |  |  |
|  |  | 87,490,000 | 87,840,000 | Carlson |  |
|  |  | 86,894,226 | 88,246,057 | Oleksyk | h |
| **13** | **13.01** | **62,700,000** | **62,760,000** |  |  |
|  |  | 62,440,000 | 62,760,000 | Carlson |  |
|  |  | 62,700,000 | 62,850,000 | Hapmap II |  |
| **14** | **14.01** | **58,376,472** | **58,395,000** |  |  |
|  |  | 58,334,879 | 58,583,052 | Oleksyk | b |
|  |  | 58,376,472 | 58,520,318 | Wang |  |
|  | **14.02** | **58,400,000** | **58,500,000** |  |  |
|  |  | 58,334,879 | 58,583,052 | Oleksyk | b |
|  |  | 58,500,000 | 58,500,000 | Voght |  |
|  |  | 58,376,472 | 58,520,318 | Wang |  |
|  | **14.03** | **58,505,000** | **58,520,318** |  |  |
|  |  | 58,334,879 | 58,583,052 | Oleksyk | b |
|  |  | 58,376,472 | 58,520,318 | Wang |  |
|  | **14.04** | **60,149,309** | **60,149,309** |  |  |
|  |  | 60,149,309 | 60,149,309 | Akey |  |
|  |  | 60,100,000 | 60,200,000 | Voght |  |
|  | **14.05** | **65,489,505** | **65,495,000** |  |  |
|  |  | 65,000,000 | 65,500,000 | Hapmap |  |
|  |  | 65,489,505 | 66,982,767 | Oleksyk | e |
|  | **14.06** | **65,500,000** | **65,500,000** |  |  |
|  |  | 65,000,000 | 65,500,000 | Hapmap |  |
|  |  | 65,489,505 | 66,982,767 | Oleksyk | e |
|  |  | 65,500,000 | 65,600,000 | Voght |  |
|  | **14.07** | **65,505,000** | **65,600,000** |  |  |
|  |  | 65,489,505 | 66,982,767 | Oleksyk | e |
|  |  | 65,500,000 | 65,600,000 | Voght |  |
|  | **14.08** | **65,700,000** | **65,800,000** |  |  |
|  |  | 65,489,505 | 66,982,767 | Oleksyk | e |
|  |  | 65,700,000 | 65,800,000 | Voght |  |
|  | **14.09** | **67,741,615** | **67,741,615** |  |  |
|  |  | 67,741,615 | 67,741,615 | Akey |  |
|  |  | 67,310,000 | 67,770,000 | Carlson |  |
| **15** | **15.01** | **46,300,000** | **46,400,000** |  |  |
|  |  | 46,155,214 | 46,657,748 | Sabeti |  |
|  |  | 46,300,000 | 46,400,000 | Voght |  |
|  | **15.02** | **61,600,000** | **61,700,000** |  |  |
|  |  | 61,550,000 | 62,070,000 | Carlson |  |
|  |  | 61,700,000 | 61,700,000 | Voght |  |
|  | **15.03** | **61,748,992** | **61,795,000** |  |  |
|  |  | 61,550,000 | 62,070,000 | Carlson |  |
|  |  | 61,748,992 | 61,848,071 | Sabeti |  |
|  | **15.04** | **61,800,000** | **61,800,000** |  |  |
|  |  | 61,550,000 | 62,070,000 | Carlson |  |
|  |  | 61,748,992 | 61,848,071 | Sabeti |  |
|  |  | 61,800,000 | 61,800,000 | Voght |  |
|  | **15.05** | **61,805,000** | **61,848,071** |  |  |
|  |  | 61,550,000 | 62,070,000 | Carlson |  |
|  |  | 61,748,992 | 61,848,071 | Sabeti |  |
|  | **15.06** | **61,900,000** | **61,900,000** |  |  |
|  |  | 61,550,000 | 62,070,000 | Carlson |  |
|  |  | 61,800,000 | 61,900,000 | Voght |  |
|  | **15.07** | **62,150,000** | **62,200,000** |  |  |
|  |  | 62,150,000 | 62,300,000 | Hapmap II |  |
|  |  | 62,100,000 | 62,200,000 | Voght |  |
|  | **15.08** | **70,200,000** | **70,300,000** |  |  |
|  |  | 70,094,745 | 70,837,275 | Oleksyk | e |
|  |  | 70,200,000 | 70,300,000 | Voght |  |
| **16** | **16.01** | **10,869,626** | **10,869,626** |  |  |
|  |  | 10,869,626 | 10,869,626 | Akey |  |
|  |  | 8,773,580 | 12,482,931 | Huttley |  |
|  | **16.02** | **14,450,000** | **14,455,000** |  |  |
|  |  | 14,450,000 | 14,550,000 | Hapmap II |  |
|  |  | 14,264,421 | 14,510,548 | Oleksyk | b |
|  | **16.03** | **14,460,000** | **14,510,548** |  |  |
|  |  | 14,460,000 | 14,760,000 | Carlson |  |
|  |  | 14,450,000 | 14,550,000 | Hapmap II |  |
|  |  | 14,264,421 | 14,510,548 | Oleksyk | b |
|  | **16.04** | **14,515,000** | **14,550,000** |  |  |
|  |  | 14,460,000 | 14,760,000 | Carlson |  |
|  |  | 14,450,000 | 14,550,000 | Hapmap II |  |
|  | **16.05** | **22,900,000** | **22,950,000** |  |  |
|  |  | 22,850,000 | 22,950,000 | Hapmap II |  |
|  |  | 22,900,000 | 23,000,000 | Voght |  |
|  | **16.06** | **64,200,000** | **64,350,000** |  |  |
|  |  | 64,200,000 | 64,350,000 | Hapmap II |  |
|  |  | 64,165,845 | 64,452,865 | Sabeti |  |
|  | **16.07** | **65,590,000** | **65,600,000** |  |  |
|  |  | 65,590,000 | 66,060,000 | Carlson |  |
|  |  | 65,500,000 | 65,600,000 | Voght |  |
| **17** | **17.01** | **5,282,598** | **5,300,000** |  |  |
|  |  | 5,282,598 | 5,429,076 | Oleksyk | a |
|  |  | 5,200,000 | 5,300,000 | Voght |  |
|  | **17.02** | **37,314,880** | **37,377,355** |  |  |
|  |  | 36,978,510 | 45,195,549 | Huttley |  |
|  |  | 37,314,880 | 37,377,355 | Oleksyk | d |
|  | **17.03** | **38,834,981** | **38,901,649** |  |  |
|  |  | 36,978,510 | 45,195,549 | Huttley |  |
|  |  | 38,834,981 | 38,901,649 | Oleksyk | e |
|  | **17.04** | **44,600,000** | **44,700,000** |  |  |
|  |  | 36,978,510 | 45,195,549 | Huttley |  |
|  |  | 44,700,000 | 44,700,000 | Voght |  |
|  | **17.05** | **44,800,000** | **44,800,000** |  |  |
|  |  | 36,978,510 | 45,195,549 | Huttley |  |
|  |  | 44,700,000 | 44,800,000 | Voght |  |
|  | **17.06** | **56,150,000** | **56,415,000** |  |  |
|  |  | 56,150,000 | 56,450,000 | Hapmap II |  |
|  |  | 55,057,635 | 56,734,520 | Oleksyk | f |
|  | **17.07** | **56,419,222** | **56,450,000** |  |  |
|  |  | 56,150,000 | 56,450,000 | Hapmap II |  |
|  |  | 55,057,635 | 56,734,520 | Oleksyk | f |
|  |  | 56,419,222 | 56,515,445 | Sabeti |  |
|  | **17.08** | **56,455,000** | **56,515,445** |  |  |
|  |  | 55,057,635 | 56,734,520 | Oleksyk | f |
|  |  | 56,419,222 | 56,515,445 | Sabeti |  |
|  | **17.09** | **61,750,000** | **61,800,000** |  |  |
|  |  | 61,750,000 | 61,850,000 | Hapmap II |  |
|  |  | 61,700,000 | 61,800,000 | Voght |  |
| **18** | **18.01** | **7,500,000** | **7,600,000** |  |  |
|  |  | 7,500,000 | 7,650,000 | Hapmap II |  |
|  |  | 7,500,000 | 7,600,000 | Voght |  |
|  | **18.02** | **28,800,000** | **28,895,000** |  |  |
|  |  | 28,640,000 | 29,150,000 | Carlson |  |
|  |  | 28,800,000 | 29,200,000 | Hapmap II |  |
|  | **18.03** | **28,900,000** | **29,000,000** |  |  |
|  |  | 28,640,000 | 29,150,000 | Carlson |  |
|  |  | 28,800,000 | 29,200,000 | Hapmap II |  |
|  |  | 28,900,000 | 29,000,000 | Voght |  |
|  | **18.04** | **29,005,000** | **29,150,000** |  |  |
|  |  | 28,640,000 | 29,150,000 | Carlson |  |
|  |  | 28,800,000 | 29,200,000 | Hapmap II |  |
|  | **18.05** | **29,170,000** | **29,170,000** |  |  |
|  |  | 28,640,000 | 29,170,000 | Carlson |  |
|  |  | 28,800,000 | 29,200,000 | Hapmap II |  |
|  | **18.06** | **39,200,000** | **39,250,000** |  |  |
|  |  | 38,800,000 | 39,250,000 | Hapmap II |  |
|  |  | 39,200,000 | 39,300,000 | Voght |  |
|  | **18.07** | **52,867,583** | **52,876,962** |  |  |
|  |  | 52,867,583 | 59,688,961 | Huttley |  |
|  |  | 51,876,927 | 52,876,962 | Oleksyk | c |
|  | **18.08** | **58,575,595** | **58,620,646** |  |  |
|  |  | 52,867,583 | 59,688,961 | Huttley |  |
|  |  | 58,575,595 | 58,620,646 | Oleksyk | d |
|  | **18.09** | **69,000,000** | **69,050,000** |  |  |
|  |  | 68,900,000 | 69,050,000 | Hapmap II |  |
|  |  | 69,000,000 | 69,100,000 | Voght |  |
|  | **18.10** | **70,800,000** | **70,900,000** |  |  |
|  |  | 70,800,000 | 70,900,000 | Hapmap II |  |
|  |  | 70,800,000 | 70,900,000 | Voght |  |
| **19** | **19.01** | **38,200,000** | **38,300,000** |  |  |
|  |  | 37,518,384 | 38,803,281 | Huttley |  |
|  |  | 38,200,000 | 38,300,000 | Voght |  |
|  | **19.02** | **42,700,000** | **42,720,601** |  |  |
|  |  | 42,700,000 | 42,800,000 | Voght |  |
|  |  | 42,689,538 | 42,720,601 | Wang |  |
|  | **19.04** | **43,500,000** | **43,600,000** |  |  |
|  |  | 43,400,000 | 43,600,000 | Hapmap II |  |
|  |  | 43,500,000 | 43,600,000 | Voght |  |
|  | **19.05** | **47,750,000** | **47,920,000** |  |  |
|  |  | 47,540,000 | 47,920,000 | Carlson |  |
|  |  | 47,750,000 | 48,250,000 | Hapmap |  |
| **20** | **20.01** | **33,864,022** | **33,900,000** |  |  |
|  |  | 33,700,000 | 33,900,000 | Hapmap II |  |
|  |  | 33,864,022 | 33,980,565 | Wang |  |
| **22** | **22.01** | **26,712,933** | **26,795,000** |  |  |
|  |  | 26,700,000 | 27,190,000 | Carlson |  |
|  |  | 26,712,933 | 27,090,089 | Oleksyk | c |
|  | **22.02** | **26,800,000** | **26,900,000** |  |  |
|  |  | 26,700,000 | 27,190,000 | Carlson |  |
|  |  | 26,712,933 | 27,090,089 | Oleksyk | c |
|  |  | 26,800,000 | 26,900,000 | Voght |  |
|  | **22.03** | **26,905,000** | **27,090,089** |  |  |
|  |  | 26,700,000 | 27,190,000 | Carlson |  |
|  |  | 26,712,933 | 27,090,089 | Oleksyk | c |
|  | **22.04** | **34,800,000** | **34,900,000** |  |  |
|  |  | 34,800,000 | 35,100,000 | Hapmap II |  |
|  |  | 34,023,587 | 34,961,386 | Huttley |  |
|  |  | 34,900,000 | 34,900,000 | Voght |  |
|  | **22.05** | **34,905,000** | **34,961,386** |  |  |
|  |  | 34,800,000 | 35,100,000 | Hapmap II |  |
|  |  | 34,023,587 | 34,961,386 | Huttley |  |
|  | **22.06** | **35,000,000** | **35,000,000** |  |  |
|  |  | 34,800,000 | 35,100,000 | Hapmap II |  |
|  |  | 34,900,000 | 35,000,000 | Voght |  |
|  | **22.07** | **37,941,616** | **37,941,616** |  |  |
|  |  | 37,941,616 | 37,941,616 | Akey |  |
|  |  | 37,900,000 | 38,000,000 | Voght |  |
| **X** | **X.01** | **13,909,076** | **14,000,000** |  |  |
|  |  | 13,900,000 | 14,000,000 | Voght |  |
|  |  | 13,909,076 | 14,111,360 | Wang |  |
|  | **X.02** | **18,881,880** | **19,050,000** |  |  |
|  |  | 18,850,000 | 19,050,000 | Hapmap II |  |
|  |  | 18,881,880 | 19,138,487 | Sabeti |  |
|  | **X.03** | **20,000,000** | **20,100,000** |  |  |
|  |  | 19,200,000 | 21,300,000 | Hapmap |  |
|  |  | 20,000,000 | 20,100,000 | Voght |  |
|  | **X.04** | **34,900,000** | **34,900,000** |  |  |
|  |  | 34,900,000 | 35,350,000 | Hapmap II |  |
|  |  | 34,800,000 | 34,900,000 | Voght |  |
|  | **X.05** | **35,600,000** | **35,700,000** |  |  |
|  |  | 35,400,000 | 37,500,000 | Hapmap |  |
|  |  | 35,700,000 | 35,700,000 | Voght |  |
|  | **X.06** | **35,759,035** | **35,795,000** |  |  |
|  |  | 35,400,000 | 37,500,000 | Hapmap |  |
|  |  | 35,759,035 | 35,939,638 | Sabeti |  |
|  | **X.07** | **35,800,000** | **35,800,000** |  |  |
|  |  | 35,400,000 | 37,500,000 | Hapmap |  |
|  |  | 35,759,035 | 35,939,638 | Sabeti |  |
|  |  | 35,800,000 | 35,800,000 | Voght |  |
|  | **X.08** | **35,805,000** | **35,895,000** |  |  |
|  |  | 35,400,000 | 37,500,000 | Hapmap |  |
|  |  | 35,759,035 | 35,939,638 | Sabeti |  |
|  | **X.09** | **35,900,000** | **35,900,000** |  |  |
|  |  | 35,400,000 | 37,500,000 | Hapmap |  |
|  |  | 35,759,035 | 35,939,638 | Sabeti |  |
|  |  | 35,800,000 | 35,900,000 | Voght |  |
|  | **X.10** | **35,905,000** | **35,939,638** |  |  |
|  |  | 35,400,000 | 37,500,000 | Hapmap |  |
|  |  | 35,759,035 | 35,939,638 | Sabeti |  |
|  | **X.11** | **36,239,501** | **36,347,420** |  |  |
|  |  | 35,400,000 | 37,500,000 | Hapmap |  |
|  |  | 36,239,501 | 36,347,420 | Wang |  |
|  | **X.12** | **36,461,698** | **36,475,000** |  |  |
|  |  | 35,400,000 | 37,500,000 | Hapmap |  |
|  |  | 36,461,698 | 36,567,625 | Wang |  |
|  | **X.13** | **36,476,826** | **36,521,901** |  |  |
|  |  | 35,400,000 | 37,500,000 | Hapmap |  |
|  |  | 36,476,826 | 36,521,901 | Sabeti |  |
|  |  | 36,461,698 | 36,567,625 | Wang |  |
|  | **X.14** | **36,525,000** | **36,567,625** |  |  |
|  |  | 35,400,000 | 37,500,000 | Hapmap |  |
|  |  | 36,461,698 | 36,567,625 | Wang |  |
|  | **X.15** | **37,069,665** | **37,500,000** |  |  |
|  |  | 35,400,000 | 37,500,000 | Hapmap |  |
|  |  | 37,069,665 | 37,555,024 | Sabeti |  |
|  | **X.16** | **59,910,280** | **59,910,280** |  |  |
|  |  | 59,910,280 | 59,910,280 | Akey |  |
|  |  | 57,700,000 | 61,850,000 | Hapmap II |  |
|  | **X.17** | **59,978,912** | **59,978,912** |  |  |
|  |  | 59,978,912 | 59,978,912 | Akey |  |
|  |  | 57,700,000 | 61,850,000 | Hapmap II |  |
|  | **X.18** | **61,800,000** | **61,850,000** |  |  |
|  |  | 61,800,000 | 64,500,000 | Hapmap |  |
|  |  | 61,800,000 | 61,850,000 | Hapmap II |  |
|  | **X.19** | **62,804,285** | **62,804,285** |  |  |
|  |  | 62,804,285 | 62,804,285 | Akey |  |
|  |  | 61,800,000 | 64,500,000 | Hapmap |  |
|  | **X.20** | **62,818,088** | **62,818,088** |  |  |
|  |  | 62,818,088 | 62,818,088 | Akey |  |
|  |  | 61,800,000 | 64,500,000 | Hapmap |  |
|  | **X.21** | **62,850,103** | **62,850,103** |  |  |
|  |  | 62,850,103 | 62,850,103 | Akey |  |
|  |  | 61,800,000 | 64,500,000 | Hapmap |  |
|  | **X.22** | **62,924,341** | **62,924,341** |  |  |
|  |  | 62,924,341 | 62,924,341 | Akey |  |
|  |  | 61,800,000 | 64,500,000 | Hapmap |  |
|  | **X.23** | **63,800,000** | **63,900,000** |  |  |
|  |  | 61,800,000 | 64,500,000 | Hapmap |  |
|  |  | 63,900,000 | 63,900,000 | Voght |  |
|  | **X.24** | **64,000,000** | **64,000,000** |  |  |
|  |  | 61,800,000 | 64,500,000 | Hapmap |  |
|  |  | 64,000,000 | 64,000,000 | Voght |  |
|  | **X.25** | **64,100,000** | **64,100,000** |  |  |
|  |  | 61,800,000 | 64,500,000 | Hapmap |  |
|  |  | 64,000,000 | 64,100,000 | Voght |  |
|  | **X.26** | **65,200,000** | **65,200,000** |  |  |
|  |  | 61,800,000 | 65,200,000 | Hapmap II |  |
|  |  | 65,159,749 | 67,836,528 | Oleksyk | a |
|  | **X.27** | **66,000,000** | **66,100,000** |  |  |
|  |  | 65,159,749 | 67,836,528 | Oleksyk | a |
|  |  | 66,000,000 | 66,100,000 | Voght |  |
|  | **X.28** | **66,129,506** | **66,195,000** |  |  |
|  |  | 65,159,749 | 67,836,528 | Oleksyk | a |
|  |  | 66,129,506 | 66,520,438 | Wang |  |
|  | **X.29** | **66,200,000** | **66,500,000** |  |  |
|  |  | 66,200,000 | 66,500,000 | Hapmap II |  |
|  |  | 65,159,749 | 67,836,528 | Oleksyk | a |
|  |  | 66,129,506 | 66,520,438 | Wang |  |
|  | **X.30** | **66,505,000** | **66,520,438** |  |  |
|  |  | 65,159,749 | 67,836,528 | Oleksyk | a |
|  |  | 66,129,506 | 66,520,438 | Wang |  |
|  | **X.31** | **104,400,000** | **104,680,742** |  |  |
|  |  | 104,400,000 | 106,500,000 | Hapmap |  |
|  |  | 104,296,315 | 104,680,742 | Oleksyk | b |
|  | **X.32** | **104,955,826** | **105,001,082** |  |  |
|  |  | 104,400,000 | 106,500,000 | Hapmap |  |
|  |  | 104,955,826 | 105,001,082 | Wang |  |
|  | **X.33** | **106,114,888** | **106,114,888** |  |  |
|  |  | 106,114,888 | 106,114,888 | Akey |  |
|  |  | 104,400,000 | 106,500,000 | Hapmap |  |
|  | **X.34** | **106,170,703** | **106,454,568** |  |  |
|  |  | 104,400,000 | 106,500,000 | Hapmap |  |
|  |  | 106,170,703 | 106,454,568 | Wang |  |
|  | **X.35** | **109,767,056** | **110,700,000** |  |  |
|  |  | 108,900,000 | 110,700,000 | Hapmap |  |
|  |  | 109,767,056 | 111,117,626 | Sabeti |  |

^†^ Note all locations were converted into base pair (bp) positions from Build 35 of the human genome. The overlapping coordinates highlighted in bold are exclusive: only the overlapping portions are indicated). The inclusive range can be obtained by selectiong the lowest beginning and the highest end coordinate of the the overlapping regions indicated below.

^‡^ The studies referenced are Huttley et al. [20], Akey et al. [21], Carlson et al. [22], Nielsen et al. [23], International HapMap Consortium[24] , Voight et al. [25], Wang et al. [26], the second generation Hapmap [27], and Sabeti et al.[28].

^^ Peak name refers to the peak name nomenclature in this study, same as in Figures 5 and 6.
