# Supplementary material for: Identifying Selected Regions from Heterozygosity and Divergence Using a Light-Coverage Genomic Dataset from Two Human Populations
Source: PLoS One. 2008 Mar 5;3(3):e1712. doi: 10.1371/journal.pone.0001712 (PMC2248624; doi:10.1371/journal.pone.0001712)
Supplement: Table S5 — Locations of all the discovered regions and sites used in this scan and other studies (3.00 MB DOC) [file pone.0001712.s006.doc]

Table S5. Locations of all the discovered regions and sites used in this scan and seven other studies^†^

| **Chromo-some** | **Beginning‡ (bp)** | **End (bp)** | **Study^^** | **Peak name^♦^** | **Cross-validated^^** | **Region name^^** | **Populations** |
| --- | --- | --- | --- | --- | --- | --- | --- |
| 1 | 2,300,000 | 2,400,000 | Voght | . | ○ |  | Africans |
| 1 | 4,102,098 | 4,102,098 | Akey | . | ○ |  |  |
| 1 | 6,264,281 | 6,267,893 | Oleksyk | a | ○ |  | Europeans |
| 1 | 8,400,000 | 8,500,000 | Voght | . | ○ |  | Asians |
| 1 | 22,117,017 | 22,559,660 | Oleksyk | b | ○ |  | (old) Africans & Europeans |
| 1 | 23,374,093 | 23,374,093 | Akey | . | ○ |  |  |
| 1 | 26,400,000 | 26,500,000 | Voght | . | ○ |  | Asians |
| 1 | 26,520,230 | 26,520,230 | Akey | . | ○ |  |  |
| 1 | 26,906,739 | 26,920,354 | Oleksyk | c | ○ |  | (old) Africans & Europeans |
| 1 | 29,900,000 | 30,000,000 | Voght | . | ○ |  | Asians |
| 1 | 30,359,744 | 30,366,699 | Sabeti | . | ○ |  | Europeans |
| 1 | 32,000,000 | 32,500,000 | Hapmap | . | ● | 1.01 |  |
| 1 | 32,190,887 | 32,909,278 | Oleksyk | d | ● | 1.01 | (old) Africans & Europeans |
| 1 | 35,100,000 | 35,300,000 | Hapmap II | . | ● | 1.02 | Europeans |
| 1 | 35,100,000 | 35,300,000 | Hapmap II | . | ● | 1.03 | Europeans |
| 1 | 35,100,000 | 35,300,000 | Hapmap II | . | ● | 1.04 | Europeans |
| 1 | 35,100,000 | 35,300,000 | Hapmap II | . | ● | 1.05 | Europeans |
| 1 | 35,109,198 | 35,164,815 | Sabeti | . | ● | 1.02 | Europeans |
| 1 | 35,109,198 | 35,164,815 | Sabeti | . | ● | 1.03 | Europeans |
| 1 | 35,116,691 | 36,614,746 | Oleksyk | e | ● | 1.03 | Europeans |
| 1 | 35,116,691 | 36,614,746 | Oleksyk | e | ● | 1.04 | Europeans |
| 1 | 35,116,691 | 36,614,746 | Oleksyk | e | ● | 1.05 | Europeans |
| 1 | 35,116,691 | 36,614,746 | Oleksyk | e | ● | 1.06 | Europeans |
| 1 | 35,220,000 | 36,210,000 | Carlson | . | ● | 1.05 |  |
| 1 | 35,220,000 | 36,210,000 | Carlson | . | ● | 1.06 |  |
| 1 | 37,103,964 | 37,103,964 | Akey | . | ○ |  |  |
| 1 | 40,000,000 | 40,100,000 | Voght | . | ○ |  | Africans |
| 1 | 40,100,000 | 40,200,000 | Voght | . | ○ |  | Asians |
| 1 | 41,080,000 | 41,470,000 | Carlson | . | ○ |  |  |
| 1 | 44,027,241 | 44,112,552 | Oleksyk | f | ○ |  | Europeans |
| 1 | 45,639,202 | 45,871,713 | Wang | . | ○ |  | All populations |
| 1 | 46,786,452 | 46,786,452 | Akey | . | ○ |  |  |
| 1 | 48,108,118 | 48,108,118 | Akey | . | ○ |  |  |
| 1 | 48,419,459 | 48,881,303 | Wang | . | ○ |  | All populations |
| 1 | 52,128,099 | 52,815,909 | Oleksyk | g | ○ |  | (old) Africans & Europeans |
| 1 | 53,779,175 | 54,067,861 | Oleksyk | h | ○ |  | Europeans |
| 1 | 55,500,000 | 55,600,000 | Voght | . | ○ |  | Europeans |
| 1 | 63,600,000 | 63,700,000 | Voght | . | ○ |  | Asians |
| 1 | 64,600,000 | 64,700,000 | Voght | . | ○ |  | Europeans |
| 1 | 65,300,000 | 65,400,000 | Voght | . | ○ |  | Asians |
| 1 | 65,400,000 | 65,500,000 | Voght | . | ○ |  | Asians |
| 1 | 65,800,000 | 65,900,000 | Hapmap II | . | ○ |  | Asians |
| 1 | 68,400,000 | 68,550,000 | Hapmap II | . | ○ |  | Europeans |
| 1 | 70,200,000 | 70,400,000 | Hapmap II | . | ○ |  | Africans |
| 1 | 72,300,000 | 72,400,000 | Voght | . | ● | 1.07 | Asians |
| 1 | 72,310,000 | 72,790,000 | Carlson | . | ● | 1.07 |  |
| 1 | 73,050,000 | 73,650,000 | Hapmap II | . | ● | 1.08 | Asians |
| 1 | 73,050,000 | 73,650,000 | Hapmap II | . | ● | 1.09 | Asians |
| 1 | 73,050,000 | 73,650,000 | Hapmap II | . | ● | 1.10 | Asians |
| 1 | 73,100,000 | 73,200,000 | Voght | . | ○ |  | Asians |
| 1 | 73,200,000 | 73,200,000 | Voght | . | ● | 1.08 |  |
| 1 | 73,200,000 | 73,300,000 | Voght | . | ● | 1.10 | Asians |
| 1 | 73,240,438 | 73,240,438 | Akey | . | ● | 1.09 |  |
| 1 | 74,100,000 | 74,200,000 | Voght | . | ○ |  | Asians |
| 1 | 74,800,000 | 74,900,000 | Voght | . | ● | 1.11 | Asians |
| 1 | 74,856,066 | 74,856,066 | Akey | . | ● | 1.11 |  |
| 1 | 74,900,000 | 75,000,000 | Voght | . | ○ |  | Asians |
| 1 | 75,100,000 | 75,200,000 | Voght | . | ○ |  | Asians |
| 1 | 75,200,000 | 75,300,000 | Voght | . | ○ |  | Asians |
| 1 | 75,400,000 | 75,500,000 | Voght | . | ○ |  | Asians |
| 1 | 75,500,000 | 75,600,000 | Voght | . | ○ |  | Asians |
| 1 | 75,900,000 | 76,000,000 | Voght | . | ● | 1.12 | Asians |
| 1 | 75,914,948 | 75,914,948 | Akey | . | ● | 1.12 |  |
| 1 | 76,200,000 | 76,300,000 | Hapmap II | . | ○ |  | Asians |
| 1 | 76,800,000 | 76,900,000 | Voght | . | ○ |  | Europeans |
| 1 | 78,100,516 | 78,100,516 | Akey | . | ○ |  |  |
| 1 | 78,800,000 | 78,900,000 | Voght | . | ○ |  | Europeans |
| 1 | 82,800,000 | 82,950,000 | Hapmap II | . | ○ |  | Asians |
| 1 | 87,010,000 | 87,320,000 | Carlson | . | ○ |  |  |
| 1 | 90,450,000 | 90,600,000 | Hapmap II | . | ○ |  | Europeans |
| 1 | 92,200,000 | 92,300,000 | Voght | . | ● | 1.13 | Africans |
| 1 | 92,220,000 | 93,030,000 | Carlson | . | ● | 1.13 |  |
| 1 | 92,220,000 | 93,030,000 | Carlson | . | ○ |  |  |
| 1 | 92,850,000 | 93,050,000 | Hapmap II | . | ○ |  | Asians |
| 1 | 94,050,000 | 94,150,000 | Hapmap II | . | ○ |  | Asians |
| 1 | 98,436,307 | 98,436,307 | Akey | . | ○ |  |  |
| 1 | 99,409,173 | 99,409,173 | Akey | . | ○ |  |  |
| 1 | 99,930,488 | 99,930,488 | Akey | . | ○ |  |  |
| 1 | 106,350,000 | 106,500,000 | Hapmap II | . | ○ |  | Africans |
| 1 | 109,763,587 | 109,763,587 | Akey | . | ○ |  |  |
| 1 | 113,200,000 | 113,300,000 | Voght | . | ○ |  | Asians |
| 1 | 113,500,000 | 113,600,000 | Voght | . | ○ |  | Asians |
| 1 | 113,600,000 | 113,700,000 | Voght | . | ○ |  | Africans |
| 1 | 115,129,044 | 115,129,044 | Akey | . | ○ |  |  |
| 1 | 115,169,882 | 115,169,882 | Akey | . | ○ |  |  |
| 1 | 117,300,000 | 117,400,000 | Voght | . | ○ |  | Europeans |
| 1 | 118,500,000 | 118,600,000 | Voght | . | ○ |  | Asians |
| 1 | 118,900,000 | 119,000,000 | Voght | . | ○ |  | Europeans |
| 1 | 119,327,685 | 119,327,685 | Akey | . | ○ |  |  |
| 1 | 152,200,000 | 152,300,000 | Voght | . | ○ |  | Asians |
| 1 | 152,300,000 | 152,400,000 | Voght | . | ○ |  | Asians |
| 1 | 152,400,000 | 152,500,000 | Voght | . | ○ |  | Asians |
| 1 | 152,421,035 | 152,427,817 | Wang | . | ○ |  | All populations |
| 1 | 156,554,765 | 156,554,765 | Akey | . | ○ |  |  |
| 1 | 157,359,782 | 157,359,782 | Hapmap II | . | ○ |  | Europeans |
| 1 | 157,850,000 | 157,950,000 | Hapmap II | . | ○ |  | Africans |
| 1 | 158,200,000 | 158,300,000 | Voght | . | ○ |  | Africans |
| 1 | 159,200,000 | 159,300,000 | Voght | . | ○ |  | Europeans |
| 1 | 160,219,288 | 160,755,021 | Wang | . | ○ |  | All populations |
| 1 | 162,763,939 | 162,763,939 | Akey | . | ○ |  |  |
| 1 | 165,850,000 | 166,100,000 | Hapmap II | . | ○ |  | Asians |
| 1 | 166,300,000 | 166,400,000 | Voght | . | ○ |  | Asians |
| 1 | 166,400,000 | 166,500,000 | Voght | . | ○ |  | Asians |
| 1 | 167,900,242 | 167,900,242 | Hapmap II | . | ○ |  | Europeans |
| 1 | 169,450,000 | 169,550,000 | Hapmap II | . | ○ |  | Asians |
| 1 | 169,900,000 | 170,000,000 | Voght | . | ○ |  | Asians |
| 1 | 170,000,000 | 170,100,000 | Voght | . | ○ |  | Asians |
| 1 | 170,100,000 | 170,200,000 | Voght | . | ○ |  | Asians |
| 1 | 170,200,000 | 170,300,000 | Voght | . | ○ |  | Asians |
| 1 | 170,569,189 | 170,574,438 | Wang | . | ○ |  | All populations |
| 1 | 170,678,392 | 170,678,392 | Akey | . | ○ |  |  |
| 1 | 175,129,749 | 175,170,302 | Oleksyk | i | ○ |  | (old) Africans & Europeans |
| 1 | 180,900,000 | 181,000,000 | Voght | . | ○ |  | Asians |
| 1 | 183,700,000 | 183,800,000 | Voght | . | ○ |  | Europeans |
| 1 | 183,700,000 | 183,800,000 | Voght | . | ○ |  | Asians |
| 1 | 186,500,000 | 186,650,000 | Hapmap II | . | ○ |  | Europeans |
| 1 | 190,900,000 | 191,000,000 | Voght | . | ○ |  | Asians |
| 1 | 191,600,000 | 191,700,000 | Voght | . | ○ |  | Europeans |
| 1 | 191,700,000 | 191,800,000 | Voght | . | ○ |  | Europeans |
| 1 | 193,450,000 | 193,550,000 | Hapmap II | . | ○ |  | Africans |
| 1 | 216,200,000 | 216,300,000 | Hapmap II | . | ○ |  | Europeans |
| 1 | 216,900,000 | 217,000,000 | Voght | . | ○ |  | Europeans |
| 1 | 222,100,000 | 222,200,000 | Voght | . | ○ |  | Africans |
| 1 | 224,200,000 | 224,300,000 | Voght | . | ○ |  | Asians |
| 1 | 224,300,000 | 224,400,000 | Voght | . | ○ |  | Asians |
| 1 | 224,400,000 | 224,500,000 | Voght | . | ○ |  | Asians |
| 2 | 7,900,000 | 8,050,000 | Hapmap II | . | ○ |  | Europeans |
| 2 | 9,315,721 | 9,315,721 | Sabeti | . | ○ |  | Asians |
| 2 | 9,700,000 | 9,800,000 | Hapmap II | . | ○ |  | Asians |
| 2 | 17,200,000 | 17,300,000 | Voght | . | ○ |  | Asians |
| 2 | 17,300,000 | 17,400,000 | Voght | . | ○ |  | Asians |
| 2 | 17,947,044 | 17,947,044 | Akey | . | ○ |  |  |
| 2 | 17,969,112 | 17,969,112 | Akey | . | ○ |  |  |
| 2 | 18,400,000 | 18,500,000 | Voght | . | ○ |  | Europeans |
| 2 | 21,034,271 | 25,071,304 | Huttley | . | ● | 2.01 |  |
| 2 | 21,034,271 | 25,071,304 | Huttley | . | ● | 2.02 |  |
| 2 | 21,034,271 | 25,071,304 | Huttley | . | ● | 2.03 |  |
| 2 | 21,034,271 | 25,071,304 | Huttley | . | ● | 2.04 |  |
| 2 | 21,034,271 | 25,071,304 | Huttley | . | ● | 2.06 |  |
| 2 | 21,034,271 | 25,071,304 | Huttley | . | ● | 2.06 |  |
| 2 | 21,034,271 | 25,071,304 | Huttley | . | ● | 2.07 |  |
| 2 | 21,034,271 | 25,071,304 | Huttley | . | ● | 2.08 |  |
| 2 | 21,034,271 | 25,071,304 | Huttley | . | ● | 2.09 |  |
| 2 | 21,034,271 | 25,071,304 | Huttley | . | ● | 2.10 |  |
| 2 | 21,034,271 | 25,071,304 | Huttley | . | ● | 2.11 |  |
| 2 | 21,034,271 | 25,071,304 | Huttley | . | ● | 2.12 |  |
| 2 | 21,300,000 | 21,400,000 | Voght | . | ● | 2.01 | Africans |
| 2 | 21,650,000 | 21,750,000 | Hapmap II | . | ● | 2.02 | Africans |
| 2 | 21,650,000 | 21,750,000 | Hapmap II | . | ● | 2.03 | Africans |
| 2 | 21,700,000 | 21,800,000 | Voght | . | ● | 2.03 | Africans |
| 2 | 21,700,000 | 21,800,000 | Voght | . | ● | 2.04 | Asians |
| 2 | 21,700,000 | 21,800,000 | Voght | . | ● | 2.06 | Asians |
| 2 | 21,700,000 | 21,800,000 | Voght | . | ● | 2.06 | Asians |
| 2 | 21,800,000 | 21,800,000 | Nielsen | . | ● | 2.06 |  |
| 2 | 21,800,000 | 21,800,000 | Nielsen | . | ● | 2.06 |  |
| 2 | 24,500,000 | 24,500,000 | Nielsen | . | ● | 2.07 |  |
| 2 | 24,650,000 | 24,850,000 | Hapmap II | . | ● | 2.08 | Africans |
| 2 | 24,650,000 | 24,850,000 | Hapmap II | . | ● | 2.09 | Africans |
| 2 | 24,650,000 | 24,850,000 | Hapmap II | . | ● | 2.10 | Africans |
| 2 | 24,700,000 | 24,800,000 | Voght | . | ○ |  | Africans |
| 2 | 24,800,000 | 24,800,000 | Voght | . | ● | 2.09 |  |
| 2 | 24,800,000 | 24,900,000 | Voght | . | ○ |  | Africans |
| 2 | 24,900,000 | 24,900,000 | Voght | . | ● | 2.11 |  |
| 2 | 24,900,000 | 25,000,000 | Voght | . | ● | 2.12 | Africans |
| 2 | 37,400,000 | 37,500,000 | Voght | . | ○ |  | Asians |
| 2 | 38,110,333 | 38,110,333 | Akey | . | ○ |  |  |
| 2 | 39,131,004 | 39,552,390 | Oleksyk | a | ● | 2.13 | (new) Africans & Europeans |
| 2 | 39,131,004 | 39,552,390 | Oleksyk | a | ● | 2.14 | (new) Africans & Europeans |
| 2 | 39,400,000 | 39,400,000 | Nielsen | . | ● | 2.13 |  |
| 2 | 39,400,000 | 39,500,000 | Voght | . | ● | 2.13 | Asians |
| 2 | 39,400,000 | 39,500,000 | Voght | . | ● | 2.14 | Asians |
| 2 | 41,600,000 | 41,700,000 | Voght | . | ○ |  | Africans |
| 2 | 41,700,000 | 41,800,000 | Voght | . | ○ |  | Asians |
| 2 | 43,531,578 | 43,531,578 | Akey | . | ○ |  |  |
| 2 | 43,600,000 | 43,700,000 | Voght | . | ○ |  | Europeans |
| 2 | 43,900,000 | 44,000,000 | Voght | . | ○ |  | Asians |
| 2 | 51,700,000 | 51,700,000 | Nielsen | . | ○ |  |  |
| 2 | 56,249,405 | 56,249,405 | Akey | . | ○ |  |  |
| 2 | 63,323,500 | 63,639,675 | Wang | . | ○ |  | All populations |
| 2 | 64,300,000 | 64,400,000 | Voght | . | ○ |  | Africans |
| 2 | 65,747,719 | 65,747,719 | Akey | . | ○ |  |  |
| 2 | 68,800,000 | 68,800,000 | Nielsen | . | ○ |  |  |
| 2 | 69,500,000 | 69,600,000 | Voght | . | ○ |  | Africans |
| 2 | 70,374,253 | 73,327,337 | Huttley | . | ● | 2.15 |  |
| 2 | 70,374,253 | 73,327,337 | Huttley | . | ● | 2.16 |  |
| 2 | 70,374,253 | 73,327,337 | Huttley | . | ● | 2.17 |  |
| 2 | 70,374,253 | 73,327,337 | Huttley | . | ● | 2.18 |  |
| 2 | 70,374,253 | 73,327,337 | Huttley | . | ● | 2.19 |  |
| 2 | 70,374,253 | 73,327,337 | Huttley | . | ● | 2.20 |  |
| 2 | 70,374,253 | 73,327,337 | Huttley | . | ● | 2.21 |  |
| 2 | 70,374,253 | 73,327,337 | Huttley | . | ● | 2.22 |  |
| 2 | 71,568,199 | 72,333,815 | Oleksyk | b | ● | 2.15 | (old) Africans & Europeans |
| 2 | 71,568,199 | 72,333,815 | Oleksyk | b | ● | 2.16 | (old) Africans & Europeans |
| 2 | 71,568,199 | 72,478,112 | Oleksyk | c | ● | 2.18 | Europeans |
| 2 | 72,305,454 | 72,927,242 | Sabeti | . | ● | 2.16 | Asians |
| 2 | 72,305,454 | 72,927,242 | Sabeti | . | ● | 2.17 | Asians |
| 2 | 72,305,454 | 72,927,242 | Sabeti | . | ● | 2.18 | Asians |
| 2 | 72,305,454 | 72,927,242 | Sabeti | . | ● | 2.19 | Asians |
| 2 | 72,305,454 | 72,927,242 | Sabeti | . | ● | 2.20 | Asians |
| 2 | 72,305,454 | 72,927,242 | Sabeti | . | ● | 2.21 | Asians |
| 2 | 72,700,000 | 72,800,000 | Voght | . | ● | 2.20 | Africans |
| 2 | 73,287,030 | 73,287,030 | Akey | . | ● | 2.22 |  |
| 2 | 73,800,000 | 73,950,000 | Hapmap II | . | ● | 2.23 | Europeans |
| 2 | 73,900,000 | 74,000,000 | Voght | . | ● | 2.23 | Europeans |
| 2 | 74,452,572 | 74,917,357 | Oleksyk | d | ● | 2.24 | Europeans |
| 2 | 74,452,572 | 74,917,357 | Oleksyk | d | ● | 2.25 | Europeans |
| 2 | 74,700,000 | 74,700,000 | Nielsen | . | ● | 2.24 |  |
| 2 | 74,700,000 | 74,800,000 | Voght | . | ● | 2.24 | Europeans |
| 2 | 74,700,000 | 74,800,000 | Voght | . | ● | 2.25 | Europeans |
| 2 | 76,300,000 | 76,400,000 | Voght | . | ○ |  | Europeans |
| 2 | 78,400,000 | 78,500,000 | Voght | . | ○ |  | Africans |
| 2 | 78,500,000 | 78,600,000 | Voght | . | ○ |  | Africans |
| 2 | 80,500,000 | 80,600,000 | Voght | . | ○ |  | Europeans |
| 2 | 82,000,000 | 82,100,000 | Voght | . | ○ |  | Europeans |
| 2 | 82,100,000 | 82,200,000 | Voght | . | ○ |  | Europeans |
| 2 | 83,100,000 | 83,200,000 | Voght | . | ○ |  | Asians |
| 2 | 83,300,000 | 83,550,000 | Hapmap II | . | ● | 2.26 | Asians |
| 2 | 83,400,000 | 83,500,000 | Voght | . | ○ |  | Asians |
| 2 | 83,500,000 | 83,500,000 | Voght | . | ● | 2.26 |  |
| 2 | 83,500,000 | 83,600,000 | Voght | . | ○ |  | Asians |
| 2 | 84,470,000 | 84,810,000 | Carlson | . | ○ |  |  |
| 2 | 84,540,000 | 84,810,000 | Carlson | . | ● | 2.27 |  |
| 2 | 84,540,000 | 84,910,000 | Carlson | . | ● | 2.29 |  |
| 2 | 84,700,000 | 84,700,000 | Nielsen | . | ● | 2.27 |  |
| 2 | 84,880,258 | 84,921,349 | Oleksyk | e | ● | 2.28 | (old) Africans & Europeans |
| 2 | 84,880,258 | 84,921,349 | Oleksyk | e | ● | 2.29 | (old) Africans & Europeans |
| 2 | 84,880,258 | 84,921,349 | Oleksyk | e | ● | 2.30 | (old) Africans & Europeans |
| 2 | 84,900,000 | 85,000,000 | Voght | . | ● | 2.28 | Asians |
| 2 | 84,900,000 | 85,000,000 | Voght | . | ● | 2.29 | Asians |
| 2 | 84,900,000 | 85,000,000 | Voght | . | ● | 2.30 | Asians |
| 2 | 86,600,000 | 86,700,000 | Voght | . | ○ |  | Asians |
| 2 | 86,700,000 | 86,800,000 | Voght | . | ○ |  | Africans |
| 2 | 88,900,000 | 89,000,000 | Voght | . | ○ |  | Africans |
| 2 | 89,300,000 | 89,450,000 | Hapmap II | . | ○ |  | Europeans |
| 2 | 95,298,156 | 95,310,019 | Wang | . | ● | 2.31 | All populations |
| 2 | 95,300,000 | 95,940,000 | Carlson | . | ● | 2.31 |  |
| 2 | 95,300,000 | 95,940,000 | Carlson | . | ● | 2.32 |  |
| 2 | 95,300,000 | 95,940,000 | Carlson | . | ● | 2.33 |  |
| 2 | 95,300,000 | 95,940,000 | Carlson | . | ● | 2.34 |  |
| 2 | 95,300,000 | 95,940,000 | Carlson | . | ● | 2.35 |  |
| 2 | 95,315,946 | 95,333,477 | Wang | . | ● | 2.32 | All populations |
| 2 | 95,424,957 | 95,441,810 | Wang | . | ● | 2.33 | All populations |
| 2 | 95,447,852 | 95,536,581 | Wang | . | ● | 2.34 | All populations |
| 2 | 95,838,050 | 95,838,050 | Akey | . | ● | 2.35 |  |
| 2 | 96,250,000 | 96,750,000 | Hapmap | . | ● | 2.36 |  |
| 2 | 96,334,661 | 96,393,283 | Oleksyk | f | ● | 2.36 | Africans |
| 2 | 98,400,000 | 98,500,000 | Voght | . | ○ |  | Africans |
| 2 | 99,500,000 | 99,600,000 | Voght | . | ○ |  | Africans |
| 2 | 99,600,000 | 99,700,000 | Voght | . | ○ |  | Africans |
| 2 | 100,872,641 | 100,872,641 | Akey | . | ○ |  |  |
| 2 | 107,600,000 | 107,700,000 | Voght | . | ○ |  | Asians |
| 2 | 107,700,000 | 107,800,000 | Voght | . | ○ |  | Asians |
| 2 | 108,250,000 | 109,100,000 | Hapmap II | . | ● | 2.37 | Asians |
| 2 | 108,250,000 | 109,100,000 | Hapmap II | . | ● | 2.38 | Asians |
| 2 | 108,250,000 | 109,100,000 | Hapmap II | . | ● | 2.39 | Asians |
| 2 | 108,250,000 | 109,100,000 | Hapmap II | . | ● | 2.40 | Asians |
| 2 | 108,250,000 | 109,100,000 | Hapmap II | . | ● | 2.41 | Asians |
| 2 | 108,250,000 | 109,100,000 | Hapmap II | . | ● | 2.42 | Asians |
| 2 | 108,250,000 | 109,100,000 | Hapmap II | . | ● | 2.43 | Asians |
| 2 | 108,250,000 | 109,100,000 | Hapmap II | . | ● | 2.44 | Asians |
| 2 | 108,250,000 | 109,100,000 | Hapmap II | . | ● | 2.45 | Asians |
| 2 | 108,250,000 | 109,100,000 | Hapmap II | . | ● | 2.46 | Asians |
| 2 | 108,250,000 | 109,100,000 | Hapmap II | . | ● | 2.47 | Asians |
| 2 | 108,250,000 | 109,100,000 | Hapmap II | . | ● | 2.48 | Asians |
| 2 | 108,250,000 | 109,100,000 | Hapmap II | . | ● | 2.49 | Asians |
| 2 | 108,300,000 | 108,400,000 | Voght | . | ● | 2.37 | Asians |
| 2 | 108,300,000 | 108,400,000 | Voght | . | ● | 2.38 | Asians |
| 2 | 108,350,000 | 109,120,000 | Carlson | . | ● | 2.38 |  |
| 2 | 108,350,000 | 109,120,000 | Carlson | . | ● | 2.39 |  |
| 2 | 108,350,000 | 109,120,000 | Carlson | . | ● | 2.40 |  |
| 2 | 108,350,000 | 109,120,000 | Carlson | . | ● | 2.41 |  |
| 2 | 108,350,000 | 109,120,000 | Carlson | . | ● | 2.42 |  |
| 2 | 108,350,000 | 109,120,000 | Carlson | . | ● | 2.43 |  |
| 2 | 108,350,000 | 109,120,000 | Carlson | . | ● | 2.44 |  |
| 2 | 108,350,000 | 109,120,000 | Carlson | . | ● | 2.45 |  |
| 2 | 108,350,000 | 109,120,000 | Carlson | . | ● | 2.46 |  |
| 2 | 108,350,000 | 109,120,000 | Carlson | . | ● | 2.47 |  |
| 2 | 108,350,000 | 109,120,000 | Carlson | . | ● | 2.48 |  |
| 2 | 108,350,000 | 109,120,000 | Carlson | . | ● | 2.49 |  |
| 2 | 108,408,653 | 108,971,124 | Sabeti | . | ● | 2.40 | Asians |
| 2 | 108,408,653 | 108,971,124 | Sabeti | . | ● | 2.41 | Asians |
| 2 | 108,408,653 | 108,971,124 | Sabeti | . | ● | 2.42 | Asians |
| 2 | 108,408,653 | 108,971,124 | Sabeti | . | ● | 2.43 | Asians |
| 2 | 108,408,653 | 108,971,124 | Sabeti | . | ● | 2.44 | Asians |
| 2 | 108,408,653 | 108,971,124 | Sabeti | . | ● | 2.45 | Asians |
| 2 | 108,408,653 | 108,971,124 | Sabeti | . | ● | 2.46 | Asians |
| 2 | 108,408,653 | 108,971,124 | Sabeti | . | ● | 2.47 | Asians |
| 2 | 108,495,423 | 108,495,423 | Akey | . | ● | 2.41 |  |
| 2 | 108,500,000 | 108,600,000 | Voght | . | ○ |  | Asians |
| 2 | 108,600,000 | 108,600,000 | Voght | . | ● | 2.42 |  |
| 2 | 108,600,000 | 108,700,000 | Voght | . | ○ |  | Asians |
| 2 | 108,700,000 | 108,700,000 | Voght | . | ● | 2.44 |  |
| 2 | 108,700,000 | 108,800,000 | Voght | . | ● | 2.46 | Asians |
| 2 | 109,000,000 | 109,100,000 | Voght | . | ○ |  | Asians |
| 2 | 109,100,000 | 109,100,000 | Voght | . | ● | 2.49 |  |
| 2 | 109,100,000 | 109,200,000 | Voght | . | ○ |  | Asians |
| 2 | 114,303,711 | 114,424,296 | Oleksyk | g | ○ |  | Europeans |
| 2 | 116,000,000 | 116,000,000 | Nielsen | . | ○ |  |  |
| 2 | 121,524,221 | 122,017,375 | Oleksyk | h | ● | 2.50 | Europeans |
| 2 | 121,524,221 | 122,017,375 | Oleksyk | h | ● | 2.51 | Europeans |
| 2 | 121,550,000 | 121,700,000 | Hapmap II | . | ● | 2.50 | Europeans |
| 2 | 122,000,000 | 122,000,000 | Nielsen | . | ● | 2.51 |  |
| 2 | 122,200,000 | 122,300,000 | Voght | . | ○ |  | Europeans |
| 2 | 122,400,000 | 122,500,000 | Voght | . | ○ |  | Europeans |
| 2 | 125,800,000 | 125,900,000 | Voght | . | ○ |  | Asians |
| 2 | 125,900,000 | 126,000,000 | Voght | . | ○ |  | Asians |
| 2 | 126,100,000 | 126,200,000 | Voght | . | ○ |  | Asians |
| 2 | 126,200,000 | 126,300,000 | Voght | . | ○ |  | Asians |
| 2 | 126,300,000 | 126,400,000 | Voght | . | ○ |  | Asians |
| 2 | 127,000,000 | 127,100,000 | Voght | . | ○ |  | Asians |
| 2 | 132,000,000 | 132,000,000 | Nielsen | . | ○ |  |  |
| 2 | 135,000,000 | 135,000,000 | Nielsen | . | ● | 2.52 |  |
| 2 | 135,000,000 | 136,550,000 | Hapmap II | . | ● | 2.52 | Europeans |
| 2 | 135,000,000 | 136,550,000 | Hapmap II | . | ● | 2.53 | Europeans |
| 2 | 135,000,000 | 136,550,000 | Hapmap II | . | ● | 2.54 | Europeans |
| 2 | 135,000,000 | 136,550,000 | Hapmap II | . | ● | 2.55 | Europeans |
| 2 | 135,000,000 | 136,550,000 | Hapmap II | . | ● | 2.56 | Europeans |
| 2 | 135,000,000 | 136,550,000 | Hapmap II | . | ● | 2.57 | Europeans |
| 2 | 135,000,000 | 136,550,000 | Hapmap II | . | ● | 2.58 | Europeans |
| 2 | 135,000,000 | 136,550,000 | Hapmap II | . | ● | 2.59 | Europeans |
| 2 | 135,000,000 | 136,550,000 | Hapmap II | . | ● | 2.60 | Europeans |
| 2 | 135,000,000 | 136,550,000 | Hapmap II | . | ● | 2.61 | Europeans |
| 2 | 135,000,000 | 136,550,000 | Hapmap II | . | ● | 2.62 | Europeans |
| 2 | 135,500,000 | 135,600,000 | Voght | . | ○ |  | Europeans |
| 2 | 135,600,000 | 135,600,000 | Voght | . | ● | 2.53 |  |
| 2 | 135,600,000 | 135,700,000 | Voght | . | ○ |  | Europeans |
| 2 | 135,700,000 | 135,700,000 | Voght | . | ● | 2.54 |  |
| 2 | 135,700,000 | 135,800,000 | Voght | . | ○ |  | Europeans |
| 2 | 135,800,000 | 135,800,000 | Voght | . | ● | 2.55 |  |
| 2 | 135,800,000 | 135,900,000 | Voght | . | ○ |  | Europeans |
| 2 | 135,900,000 | 135,900,000 | Voght | . | ● | 2.56 |  |
| 2 | 135,900,000 | 136,000,000 | Voght | . | ○ |  | Europeans |
| 2 | 136,000,000 | 136,000,000 | Nielsen | . | ● | 2.57 |  |
| 2 | 136,000,000 | 136,000,000 | Voght | . | ● | 2.57 |  |
| 2 | 136,000,000 | 136,100,000 | Voght | . | ○ |  | Europeans |
| 2 | 136,100,000 | 136,100,000 | Voght | . | ● | 2.58 |  |
| 2 | 136,100,000 | 136,200,000 | Voght | . | ○ |  | Europeans |
| 2 | 136,200,000 | 136,200,000 | Voght | . | ● | 2.59 |  |
| 2 | 136,200,000 | 136,300,000 | Voght | . | ○ |  | Europeans |
| 2 | 136,300,000 | 136,300,000 | Voght | . | ● | 2.60 |  |
| 2 | 136,300,000 | 136,400,000 | Voght | . | ● | 2.61 | Europeans |
| 2 | 136,500,000 | 136,600,000 | Voght | . | ● | 2.62 | Europeans |
| 2 | 136,600,000 | 136,700,000 | Voght | . | ○ |  | Europeans |
| 2 | 136,700,000 | 136,700,000 | Voght | . | ● | 2.63 |  |
| 2 | 136,700,000 | 136,800,000 | Voght | . | ○ |  | Europeans |
| 2 | 136,700,000 | 137,250,000 | Hapmap | . | ● | 2.63 |  |
| 2 | 136,700,000 | 137,250,000 | Hapmap | . | ● | 2.65 |  |
| 2 | 136,700,000 | 137,250,000 | Hapmap | . | ● | 2.65 |  |
| 2 | 136,700,000 | 137,250,000 | Hapmap | . | ● | 2.66 |  |
| 2 | 136,700,000 | 137,250,000 | Hapmap | . | ● | 2.67 |  |
| 2 | 136,700,000 | 137,250,000 | Hapmap | . | ● | 2.68 |  |
| 2 | 136,700,000 | 137,250,000 | Hapmap | . | ● | 2.69 |  |
| 2 | 136,800,000 | 136,900,000 | Voght | . | ○ |  | Europeans |
| 2 | 136,800,000 | 136,900,000 | Voght | . | ○ |  | Asians |
| 2 | 136,900,000 | 136,900,000 | Voght | . | ● | 2.65 |  |
| 2 | 136,900,000 | 136,900,000 | Voght | . | ● | 2.65 |  |
| 2 | 136,900,000 | 137,000,000 | Voght | . | ● | 2.66 | Europeans |
| 2 | 137,000,000 | 137,250,000 | Hapmap II | . | ● | 2.66 | Europeans |
| 2 | 137,000,000 | 137,250,000 | Hapmap II | . | ● | 2.67 | Europeans |
| 2 | 137,000,000 | 137,250,000 | Hapmap II | . | ● | 2.68 | Europeans |
| 2 | 137,000,000 | 137,250,000 | Hapmap II | . | ● | 2.69 | Europeans |
| 2 | 137,100,000 | 137,200,000 | Voght | . | ○ |  | Europeans |
| 2 | 137,200,000 | 137,200,000 | Voght | . | ● | 2.68 |  |
| 2 | 137,200,000 | 137,300,000 | Voght | . | ○ |  | Europeans |
| 2 | 137,400,000 | 137,500,000 | Voght | . | ○ |  | Europeans |
| 2 | 137,500,000 | 137,600,000 | Voght | . | ○ |  | Europeans |
| 2 | 137,800,000 | 137,900,000 | Voght | . | ○ |  | Europeans |
| 2 | 137,900,000 | 138,000,000 | Voght | . | ○ |  | Europeans |
| 2 | 139,000,000 | 139,000,000 | Nielsen | . | ○ |  |  |
| 2 | 144,000,000 | 144,000,000 | Nielsen | . | ○ |  |  |
| 2 | 148,000,000 | 148,000,000 | Nielsen | . | ● | 2.70 |  |
| 2 | 148,000,000 | 148,100,000 | Voght | . | ● | 2.70 | Europeans |
| 2 | 152,252,757 | 152,716,949 | Oleksyk | i | ● | 2.71 | Europeans |
| 2 | 152,700,000 | 152,800,000 | Voght | . | ● | 2.71 | Europeans |
| 2 | 152,800,000 | 152,900,000 | Voght | . | ○ |  | Europeans |
| 2 | 153,700,000 | 153,800,000 | Voght | . | ○ |  | Asians |
| 2 | 154,800,000 | 154,900,000 | Voght | . | ○ |  | Asians |
| 2 | 156,301,490 | 156,301,490 | Akey | . | ○ |  |  |
| 2 | 157,950,000 | 158,050,000 | Hapmap II | . | ● | 2.72 | Europeans |
| 2 | 158,000,000 | 158,000,000 | Nielsen | . | ● | 2.72 |  |
| 2 | 158,104,248 | 158,691,339 | Oleksyk | j | ○ |  | Europeans |
| 2 | 158,900,000 | 159,000,000 | Voght | . | ○ |  | Europeans |
| 2 | 159,100,000 | 159,250,000 | Hapmap II | . | ○ |  | Asians |
| 2 | 159,300,000 | 159,400,000 | Voght | . | ○ |  | Asians |
| 2 | 160,200,000 | 160,300,000 | Voght | . | ○ |  | Europeans |
| 2 | 162,820,000 | 163,240,000 | Carlson | . | ● | 2.73 |  |
| 2 | 162,820,000 | 163,240,000 | Carlson | . | ● | 2.74 |  |
| 2 | 162,820,000 | 163,240,000 | Carlson | . | ● | 2.75 |  |
| 2 | 162,855,843 | 163,362,927 | Oleksyk | k | ● | 2.73 | Europeans |
| 2 | 162,855,843 | 163,362,927 | Oleksyk | k | ● | 2.74 | Europeans |
| 2 | 162,855,843 | 163,362,927 | Oleksyk | k | ● | 2.75 | Europeans |
| 2 | 163,000,000 | 163,000,000 | Nielsen | . | ● | 2.74 |  |
| 2 | 164,000,000 | 164,000,000 | Nielsen | . | ○ |  |  |
| 2 | 168,100,000 | 168,200,000 | Voght | . | ○ |  | Europeans |
| 2 | 170,600,000 | 170,700,000 | Voght | . | ○ |  | Europeans |
| 2 | 176,000,000 | 176,000,000 | Nielsen | . | ○ |  |  |
| 2 | 176,500,000 | 176,600,000 | Voght | . | ○ |  | Europeans |
| 2 | 177,317,730 | 178,285,258 | Sabeti | . | ● | 2.76 | Europeans & Asians |
| 2 | 177,317,730 | 178,285,258 | Sabeti | . | ● | 2.77 | Europeans & Asians |
| 2 | 177,317,730 | 178,285,258 | Sabeti | . | ● | 2.78 | Europeans & Asians |
| 2 | 177,390,000 | 177,730,000 | Carlson | . | ● | 2.76 |  |
| 2 | 177,800,000 | 177,900,000 | Voght | . | ● | 2.77 | Asians |
| 2 | 178,250,000 | 178,450,000 | Hapmap II | . | ● | 2.78 | Europeans |
| 2 | 182,700,000 | 182,800,000 | Voght | . | ○ |  | Africans |
| 2 | 186,900,000 | 187,000,000 | Voght | . | ○ |  | Africans |
| 2 | 187,300,000 | 187,400,000 | Voght | . | ○ |  | Africans |
| 2 | 189,140,000 | 189,570,000 | Carlson | . | ○ |  |  |
| 2 | 190,200,000 | 190,300,000 | Voght | . | ○ |  | Europeans |
| 2 | 192,950,000 | 193,050,000 | Hapmap II | . | ● | 2.79 | Africans |
| 2 | 193,000,000 | 193,000,000 | Nielsen | . | ● | 2.79 |  |
| 2 | 193,300,000 | 193,400,000 | Voght | . | ○ |  | Africans |
| 2 | 194,650,000 | 194,900,000 | Hapmap II | . | ● | 2.80 | Africans |
| 2 | 194,650,000 | 194,990,000 | Carlson | . | ● | 2.80 |  |
| 2 | 195,000,000 | 195,100,000 | Voght | . | ○ |  | Africans |
| 2 | 195,100,000 | 195,200,000 | Voght | . | ○ |  | Africans |
| 2 | 195,200,000 | 195,300,000 | Voght | . | ○ |  | Africans |
| 2 | 195,300,000 | 195,400,000 | Voght | . | ○ |  | Africans |
| 2 | 195,402,140 | 195,402,140 | Akey | . | ○ |  |  |
| 2 | 196,000,000 | 196,000,000 | Nielsen | . | ○ |  |  |
| 2 | 196,700,000 | 196,800,000 | Voght | . | ○ |  | Europeans |
| 2 | 196,800,000 | 196,900,000 | Voght | . | ○ |  | Europeans |
| 2 | 197,200,000 | 197,300,000 | Hapmap II | . | ○ |  | Asians |
| 2 | 197,700,000 | 197,800,000 | Voght | . | ○ |  | Europeans |
| 2 | 199,300,000 | 199,400,000 | Voght | . | ○ |  | Asians |
| 2 | 200,000,000 | 200,100,000 | Voght | . | ○ |  | Europeans |
| 2 | 201,000,000 | 201,100,000 | Voght | . | ○ |  | Asians |
| 2 | 201,100,000 | 201,200,000 | Voght | . | ○ |  | Africans |
| 2 | 201,300,000 | 201,400,000 | Voght | . | ○ |  | Africans |
| 2 | 203,700,000 | 203,800,000 | Voght | . | ○ |  | Africans |
| 2 | 205,700,000 | 205,800,000 | Voght | . | ○ |  | Europeans |
| 2 | 206,028,349 | 206,043,667 | Sabeti | . | ○ |  | Europeans |
| 2 | 206,400,000 | 206,500,000 | Voght | . | ○ |  | Europeans |
| 2 | 213,400,000 | 213,500,000 | Voght | . | ○ |  | Asians |
| 2 | 219,300,000 | 219,400,000 | Voght | . | ● | 2.81 | Asians |
| 2 | 219,309,386 | 219,315,616 | Oleksyk | l | ● | 2.81 | Europeans |
| 2 | 223,000,000 | 223,000,000 | Nielsen | . | ○ |  |  |
| 2 | 225,900,000 | 226,000,000 | Voght | . | ○ |  | Africans |
| 2 | 226,450,000 | 226,600,000 | Hapmap II | . | ○ |  | Europeans |
| 2 | 228,000,000 | 228,000,000 | Nielsen | . | ○ |  |  |
| 2 | 230,800,000 | 230,900,000 | Voght | . | ○ |  | Asians |
| 2 | 232,457,351 | 232,901,925 | Oleksyk | m | ○ |  | (old) Africans & Europeans |
| 2 | 238,144,810 | 238,161,079 | Sabeti | . | ○ |  | Europeans |
| 2 | 238,429,085 | 238,476,547 | Oleksyk | n | ○ |  | Europeans |
| 3 | 4,500,000 | 4,600,000 | Voght | . | ○ |  | Africans |
| 3 | 10,700,000 | 10,800,000 | Voght | . | ○ |  | Europeans |
| 3 | 12,500,000 | 12,600,000 | Voght | . | ○ |  | Asians |
| 3 | 14,886,156 | 14,886,156 | Akey | . | ○ |  |  |
| 3 | 16,321,403 | 16,321,403 | Akey | . | ○ |  |  |
| 3 | 17,450,000 | 17,550,000 | Hapmap II | . | ○ |  | Asians |
| 3 | 17,570,000 | 17,890,000 | Carlson | . | ○ |  |  |
| 3 | 25,700,000 | 26,250,000 | Carlson | . | ● | 3.01 |  |
| 3 | 25,700,000 | 26,250,000 | Carlson | . | ● | 3.02 |  |
| 3 | 25,700,000 | 26,250,000 | Carlson | . | ● | 3.03 |  |
| 3 | 25,700,000 | 26,250,000 | Carlson | . | ● | 3.04 |  |
| 3 | 25,700,000 | 26,250,000 | Carlson | . | ● | 3.05 |  |
| 3 | 25,700,000 | 26,250,000 | Carlson | . | ● | 3.06 |  |
| 3 | 25,700,000 | 26,250,000 | Carlson | . | ● | 3.07 |  |
| 3 | 25,800,000 | 26,300,000 | Hapmap II | . | ● | 3.01 | Europeans & Asians |
| 3 | 25,800,000 | 26,300,000 | Hapmap II | . | ● | 3.02 | Europeans & Asians |
| 3 | 25,800,000 | 26,300,000 | Hapmap II | . | ● | 3.03 | Europeans & Asians |
| 3 | 25,800,000 | 26,300,000 | Hapmap II | . | ● | 3.04 | Europeans & Asians |
| 3 | 25,800,000 | 26,300,000 | Hapmap II | . | ● | 3.05 | Europeans & Asians |
| 3 | 25,800,000 | 26,300,000 | Hapmap II | . | ● | 3.06 | Europeans & Asians |
| 3 | 25,800,000 | 26,300,000 | Hapmap II | . | ● | 3.07 | Europeans & Asians |
| 3 | 25,900,000 | 26,000,000 | Voght | . | ○ |  | Europeans |
| 3 | 26,000,000 | 26,000,000 | Voght | . | ● | 3.02 |  |
| 3 | 26,000,000 | 26,100,000 | Voght | . | ● | 3.04 | Europeans |
| 3 | 26,230,802 | 26,239,053 | Sabeti | . | ● | 3.06 | Asians |
| 3 | 27,300,000 | 27,400,000 | Voght | . | ○ |  | Asians |
| 3 | 30,007,474 | 30,007,474 | Oleksyk | a | ○ |  | Europeans |
| 3 | 36,150,000 | 36,250,000 | Hapmap II | . | ○ |  | Europeans |
| 3 | 38,780,394 | 38,814,393 | Oleksyk | b | ○ |  | Africans |
| 3 | 44,300,000 | 44,400,000 | Voght | . | ○ |  | Asians |
| 3 | 46,000,000 | 46,100,000 | Voght | . | ○ |  | Africans |
| 3 | 46,286,786 | 46,476,846 | Oleksyk | c | ● | 3.08 | (old) Africans & Europeans |
| 3 | 46,335,592 | 46,335,592 | Akey | . | ● | 3.08 |  |
| 3 | 48,600,000 | 48,700,000 | Voght | . | ○ |  | Asians |
| 3 | 48,800,000 | 48,900,000 | Voght | . | ○ |  | Asians |
| 3 | 49,300,000 | 49,650,000 | Hapmap II | . | ● | 3.09 | Asians |
| 3 | 49,300,000 | 49,650,000 | Hapmap II | . | ● | 3.10 | Asians |
| 3 | 49,400,000 | 49,500,000 | Voght | . | ● | 3.09 | Asians |
| 3 | 49,600,000 | 49,700,000 | Voght | . | ● | 3.10 | Asians |
| 3 | 49,800,000 | 49,900,000 | Voght | . | ○ |  | Asians |
| 3 | 50,570,145 | 51,432,075 | Oleksyk | d | ● | 3.11 | (old) Africans & Europeans |
| 3 | 50,570,145 | 51,432,075 | Oleksyk | d | ● | 3.12 | (old) Africans & Europeans |
| 3 | 50,600,000 | 50,700,000 | Voght | . | ○ |  | Asians |
| 3 | 50,700,000 | 50,700,000 | Voght | . | ● | 3.11 |  |
| 3 | 50,700,000 | 50,800,000 | Voght | . | ● | 3.12 | Asians |
| 3 | 55,400,000 | 55,500,000 | Voght | . | ○ |  | Asians |
| 3 | 56,500,000 | 56,600,000 | Voght | . | ○ |  | Africans |
| 3 | 56,550,000 | 56,700,000 | Hapmap II | . | ● | 3.13 | Africans |
| 3 | 56,550,000 | 56,700,000 | Hapmap II | . | ● | 3.14 | Africans |
| 3 | 56,600,000 | 56,600,000 | Voght | . | ● | 3.13 |  |
| 3 | 56,600,000 | 56,700,000 | Voght | . | ● | 3.14 | Africans |
| 3 | 63,808,090 | 63,956,464 | Oleksyk | e | ○ |  | (old) Africans & Europeans |
| 3 | 65,400,000 | 65,500,000 | Voght | . | ○ |  | Africans |
| 3 | 66,400,000 | 66,500,000 | Voght | . | ○ |  | Asians |
| 3 | 68,300,000 | 68,400,000 | Voght | . | ○ |  | Europeans |
| 3 | 72,650,000 | 72,750,000 | Hapmap II | . | ○ |  | Europeans |
| 3 | 74,700,000 | 74,800,000 | Voght | . | ○ |  | Europeans |
| 3 | 79,150,000 | 79,250,000 | Hapmap II | . | ○ |  | Africans |
| 3 | 80,700,000 | 80,800,000 | Voght | . | ○ |  | Asians |
| 3 | 83,700,000 | 83,800,000 | Voght | . | ○ |  | Europeans |
| 3 | 86,500,000 | 86,600,000 | Voght | . | ○ |  | Africans |
| 3 | 87,300,000 | 87,400,000 | Hapmap II | . | ○ |  | Africans |
| 3 | 88,300,000 | 88,400,000 | Voght | . | ○ |  | Europeans |
| 3 | 89,690,000 | 90,110,000 | Carlson | . | ○ |  |  |
| 3 | 90,150,000 | 90,300,000 | Hapmap II | . | ● | 3.15 | Asians |
| 3 | 90,250,000 | 90,750,000 | Hapmap | . | ● | 3.15 |  |
| 3 | 98,058,107 | 98,828,098 | Oleksyk | f | ● | 3.16 | (new) Africans & Europeans |
| 3 | 98,750,000 | 99,250,000 | Hapmap | . | ● | 3.16 |  |
| 3 | 106,100,000 | 106,250,000 | Hapmap II | . | ○ |  | Asians |
| 3 | 108,500,000 | 108,600,000 | Voght | . | ○ |  | Africans |
| 3 | 108,600,000 | 108,700,000 | Voght | . | ○ |  | Asians |
| 3 | 108,754,249 | 108,994,687 | Sabeti | . | ○ |  | Asians |
| 3 | 113,538,529 | 113,831,322 | Oleksyk | g | ○ |  | Europeans |
| 3 | 117,300,000 | 117,400,000 | Voght | . | ○ |  | Europeans |
| 3 | 117,400,000 | 117,500,000 | Voght | . | ○ |  | Europeans |
| 3 | 122,000,000 | 122,100,000 | Voght | . | ○ |  | Africans |
| 3 | 122,100,000 | 122,200,000 | Voght | . | ○ |  | Africans |
| 3 | 122,200,000 | 122,300,000 | Voght | . | ○ |  | Africans |
| 3 | 122,568,114 | 122,568,114 | Akey | . | ○ |  |  |
| 3 | 122,900,000 | 123,000,000 | Voght | . | ○ |  | Asians |
| 3 | 123,800,000 | 123,900,000 | Voght | . | ○ |  | Europeans |
| 3 | 123,900,000 | 124,000,000 | Voght | . | ○ |  | Europeans |
| 3 | 125,100,000 | 125,200,000 | Voght | . | ○ |  | Europeans |
| 3 | 127,050,000 | 127,150,000 | Hapmap II | . | ○ |  | Asians |
| 3 | 127,600,000 | 127,700,000 | Voght | . | ○ |  | Asians |
| 3 | 131,400,000 | 131,500,000 | Voght | . | ○ |  | Europeans |
| 3 | 131,500,000 | 131,600,000 | Voght | . | ○ |  | Europeans |
| 3 | 133,500,000 | 133,600,000 | Voght | . | ○ |  | Europeans |
| 3 | 134,450,000 | 134,550,000 | Hapmap II | . | ○ |  | Africans |
| 3 | 135,800,000 | 135,900,000 | Voght | . | ○ |  | Asians |
| 3 | 136,000,000 | 136,100,000 | Voght | . | ○ |  | Asians |
| 3 | 140,500,000 | 140,600,000 | Voght | . | ● | 3.17 | Asians |
| 3 | 140,600,000 | 140,700,000 | Hapmap II | . | ● | 3.17 | Asians |
| 3 | 141,775,234 | 142,180,639 | Oleksyk | h | ○ |  | Europeans |
| 3 | 144,676,848 | 144,676,848 | Akey | . | ○ |  |  |
| 3 | 144,720,539 | 144,720,539 | Akey | . | ○ |  |  |
| 3 | 146,500,000 | 146,600,000 | Voght | . | ○ |  | Africans |
| 3 | 146,750,000 | 146,900,000 | Hapmap II | . | ○ |  | Africans |
| 3 | 157,905,009 | 157,919,623 | Oleksyk | i | ○ |  | (old) Africans & Europeans |
| 3 | 159,148,805 | 159,583,495 | Wang | . | ○ |  | All populations |
| 3 | 160,897,171 | 160,984,375 | Oleksyk | j | ○ |  | Europeans |
| 3 | 161,800,000 | 161,900,000 | Voght | . | ○ |  | Africans |
| 3 | 162,300,000 | 162,400,000 | Hapmap II | . | ○ |  | Asians |
| 3 | 165,000,000 | 165,100,000 | Voght | . | ○ |  | Africans |
| 3 | 165,100,000 | 165,200,000 | Voght | . | ○ |  | Africans |
| 3 | 165,100,000 | 165,200,000 | Voght | . | ○ |  | Asians |
| 3 | 165,250,000 | 165,400,000 | Hapmap II | . | ○ |  | Africans |
| 3 | 170,105,819 | 170,118,002 | Oleksyk | k | ○ |  | Africans |
| 3 | 174,500,000 | 174,600,000 | Voght | . | ○ |  | Asians |
| 3 | 176,500,000 | 176,600,000 | Voght | . | ○ |  | Asians |
| 3 | 179,600,000 | 179,700,000 | Voght | . | ○ |  | Africans |
| 3 | 189,650,000 | 189,800,000 | Hapmap II | . | ○ |  | Asians |
| 3 | 190,046,474 | 190,078,374 | Oleksyk | l | ○ |  | Africans |
| 3 | 194,192,214 | 194,192,214 | Akey | . | ○ |  |  |
| 3 | 194,598,223 | 194,598,223 | Akey | . | ○ |  |  |
| 3 | 197,000,000 | 197,150,000 | Hapmap II | . | ○ |  | Africans |
| 3 | 199,000,000 | 199,100,000 | Voght | . | ○ |  | Europeans |
| 4 | 6,427,841 | 6,427,841 | Akey | . | ○ |  |  |
| 4 | 9,504,175 | 9,504,175 | Akey | . | ○ |  |  |
| 4 | 14,700,000 | 14,800,000 | Voght | . | ○ |  | Africans |
| 4 | 20,650,000 | 20,950,000 | Hapmap II | . | ○ |  | Africans |
| 4 | 21,600,000 | 21,700,000 | Voght | . | ○ |  | Africans |
| 4 | 22,500,000 | 22,600,000 | Voght | . | ○ |  | Africans |
| 4 | 26,400,000 | 26,500,000 | Voght | . | ○ |  | Africans |
| 4 | 28,100,000 | 28,200,000 | Voght | . | ○ |  | Asians |
| 4 | 28,400,000 | 28,500,000 | Voght | . | ○ |  | Asians |
| 4 | 28,623,959 | 28,623,959 | Akey | . | ○ |  |  |
| 4 | 30,000,000 | 30,100,000 | Voght | . | ○ |  | Europeans |
| 4 | 32,930,000 | 33,560,000 | Carlson | . | ● | 4.01 |  |
| 4 | 33,200,000 | 33,300,000 | Voght | . | ● | 4.01 | Asians |
| 4 | 33,600,000 | 34,700,000 | Hapmap II | . | ● | 4.02 | All populations |
| 4 | 33,600,000 | 34,700,000 | Hapmap II | . | ● | 4.04 | All populations |
| 4 | 33,600,000 | 34,700,000 | Hapmap II | . | ● | 4.04 | All populations |
| 4 | 33,600,000 | 34,700,000 | Hapmap II | . | ● | 4.05 | All populations |
| 4 | 33,600,000 | 34,700,000 | Hapmap II | . | ● | 4.06 | All populations |
| 4 | 33,600,000 | 34,700,000 | Hapmap II | . | ● | 4.07 | All populations |
| 4 | 34,000,000 | 34,500,000 | Hapmap | . | ● | 4.02 |  |
| 4 | 34,000,000 | 34,500,000 | Hapmap | . | ● | 4.04 |  |
| 4 | 34,000,000 | 34,500,000 | Hapmap | . | ● | 4.04 |  |
| 4 | 34,000,000 | 34,500,000 | Hapmap | . | ● | 4.05 |  |
| 4 | 34,000,000 | 34,500,000 | Hapmap | . | ● | 4.06 |  |
| 4 | 34,200,000 | 34,300,000 | Voght | . | ○ |  | Europeans |
| 4 | 34,300,000 | 34,400,000 | Voght | . | ○ |  | Africans |
| 4 | 34,300,000 | 34,400,000 | Voght | . | ○ |  | Europeans |
| 4 | 34,400,000 | 34,400,000 | Voght | . | ● | 4.04 |  |
| 4 | 34,400,000 | 34,400,000 | Voght | . | ● | 4.04 |  |
| 4 | 34,400,000 | 34,500,000 | Voght | . | ○ |  | Europeans |
| 4 | 34,500,000 | 34,500,000 | Voght | . | ● | 4.06 |  |
| 4 | 34,500,000 | 34,600,000 | Voght | . | ● | 4.07 | Europeans |
| 4 | 41,300,000 | 41,400,000 | Hapmap II | . | ○ |  | Europeans & Asians |
| 4 | 41,600,000 | 41,700,000 | Voght | . | ● | 4.08 | Asians |
| 4 | 41,670,000 | 42,050,000 | Carlson | . | ● | 4.08 |  |
| 4 | 41,670,000 | 42,050,000 | Carlson | . | ● | 4.09 |  |
| 4 | 41,670,000 | 42,050,000 | Carlson | . | ● | 4.10 |  |
| 4 | 41,670,000 | 42,050,000 | Carlson | . | ● | 4.11 |  |
| 4 | 41,670,000 | 42,050,000 | Carlson | . | ● | 4.13 |  |
| 4 | 41,670,000 | 42,050,000 | Carlson | . | ● | 4.13 |  |
| 4 | 41,670,000 | 42,050,000 | Carlson | . | ● | 4.14 |  |
| 4 | 41,800,000 | 41,900,000 | Voght | . | ● | 4.09 | Europeans |
| 4 | 41,900,000 | 42,000,000 | Voght | . | ● | 4.10 | Africans |
| 4 | 41,900,000 | 42,000,000 | Voght | . | ● | 4.11 | Europeans |
| 4 | 41,900,000 | 42,050,000 | Hapmap II | . | ● | 4.10 | Asians |
| 4 | 41,900,000 | 42,050,000 | Hapmap II | . | ● | 4.11 | Asians |
| 4 | 41,900,000 | 42,050,000 | Hapmap II | . | ● | 4.13 | Asians |
| 4 | 41,900,000 | 42,050,000 | Hapmap II | . | ● | 4.13 | Asians |
| 4 | 41,900,000 | 42,050,000 | Hapmap II | . | ● | 4.14 | Asians |
| 4 | 41,984,060 | 41,989,630 | Sabeti | . | ● | 4.11 | Asians |
| 4 | 42,000,000 | 42,000,000 | Voght | . | ● | 4.13 |  |
| 4 | 42,000,000 | 42,000,000 | Voght | . | ● | 4.13 |  |
| 4 | 42,000,000 | 42,100,000 | Voght | . | ○ |  | Europeans |
| 4 | 44,600,000 | 44,700,000 | Voght | . | ○ |  | Africans |
| 4 | 44,600,000 | 44,700,000 | Voght | . | ○ |  | Asians |
| 4 | 46,400,000 | 46,500,000 | Voght | . | ○ |  | Africans |
| 4 | 48,400,000 | 48,500,000 | Voght | . | ○ |  | Africans |
| 4 | 48,600,000 | 48,700,000 | Voght | . | ● | 4.15 | Africans |
| 4 | 48,674,033 | 48,704,191 | Wang | . | ● | 4.15 | All populations |
| 4 | 48,800,000 | 48,900,000 | Voght | . | ○ |  | Africans |
| 4 | 48,900,000 | 49,000,000 | Voght | . | ○ |  | Africans |
| 4 | 56,100,000 | 56,250,000 | Hapmap II | . | ● | 4.16 | Africans |
| 4 | 56,200,000 | 56,300,000 | Voght | . | ● | 4.16 | Africans |
| 4 | 61,100,000 | 61,200,000 | Voght | . | ○ |  | Europeans |
| 4 | 61,200,000 | 61,300,000 | Voght | . | ○ |  | Europeans |
| 4 | 72,200,000 | 72,300,000 | Voght | . | ○ |  | Europeans |
| 4 | 81,900,000 | 82,000,000 | Voght | . | ○ |  | Europeans |
| 4 | 82,200,000 | 82,300,000 | Voght | . | ○ |  | Europeans |
| 4 | 82,400,000 | 82,500,000 | Voght | . | ○ |  | Europeans |
| 4 | 85,700,000 | 85,850,000 | Hapmap II | . | ○ |  | Europeans |
| 4 | 87,680,164 | 87,680,164 | Akey | . | ○ |  |  |
| 4 | 88,002,290 | 88,111,041 | Oleksyk | a | ○ |  | (old) Africans & Europeans |
| 4 | 93,850,000 | 94,050,000 | Hapmap II | . | ○ |  | Asians |
| 4 | 98,938,421 | 99,522,785 | Wang | . | ○ |  | All populations |
| 4 | 100,000,000 | 101,000,000 | Hapmap II | . | ● | 4.17 | Asians |
| 4 | 100,700,000 | 100,800,000 | Voght | . | ● | 4.17 | Asians |
| 4 | 104,300,000 | 104,400,000 | Voght | . | ○ |  | Asians |
| 4 | 104,750,000 | 104,900,000 | Hapmap II | . | ○ |  | Europeans |
| 4 | 106,873,417 | 106,937,944 | Oleksyk | b | ○ |  | Europeans |
| 4 | 107,200,000 | 107,300,000 | Voght | . | ○ |  | Europeans |
| 4 | 107,500,000 | 107,600,000 | Voght | . | ○ |  | Africans |
| 4 | 108,100,000 | 108,200,000 | Voght | . | ○ |  | Africans |
| 4 | 111,599,516 | 111,599,516 | Akey | . | ○ |  |  |
| 4 | 119,100,000 | 119,200,000 | Voght | . | ○ |  | Africans |
| 4 | 119,450,443 | 119,609,926 | Oleksyk | c | ○ |  | (old) Africans & Europeans |
| 4 | 123,550,000 | 123,650,000 | Hapmap II | . | ○ |  | Africans |
| 4 | 132,900,000 | 133,000,000 | Hapmap II | . | ○ |  | Europeans |
| 4 | 133,600,000 | 133,700,000 | Voght | . | ○ |  | Africans |
| 4 | 139,300,000 | 139,400,000 | Voght | . | ○ |  | Asians |
| 4 | 144,100,000 | 144,550,000 | Hapmap II | . | ● | 4.18 | Asians |
| 4 | 144,100,000 | 144,550,000 | Hapmap II | . | ● | 4.19 | Asians |
| 4 | 144,300,000 | 144,400,000 | Voght | . | ● | 4.18 | Asians |
| 4 | 144,500,000 | 144,600,000 | Voght | . | ● | 4.19 | Asians |
| 4 | 145,300,000 | 145,400,000 | Hapmap II | . | ○ |  | Europeans |
| 4 | 148,450,000 | 148,600,000 | Hapmap II | . | ○ |  | Africans |
| 4 | 148,700,000 | 148,800,000 | Voght | . | ○ |  | Africans |
| 4 | 148,700,000 | 148,800,000 | Voght | . | ○ |  | Europeans |
| 4 | 148,800,000 | 148,900,000 | Voght | . | ● | 4.21 | Africans |
| 4 | 148,835,482 | 149,328,142 | Oleksyk | d | ● | 4.20 | Europeans |
| 4 | 148,835,482 | 149,328,142 | Oleksyk | d | ● | 4.21 | Europeans |
| 4 | 148,835,482 | 149,328,142 | Oleksyk | d | ● | 4.22 | Europeans |
| 4 | 148,880,000 | 149,280,000 | Carlson | . | ● | 4.20 |  |
| 4 | 148,880,000 | 149,280,000 | Carlson | . | ● | 4.21 |  |
| 4 | 148,880,000 | 149,280,000 | Carlson | . | ● | 4.22 |  |
| 4 | 151,763,438 | 152,514,276 | Wang | . | ● | 4.23 | All populations |
| 4 | 151,985,548 | 152,954,145 | Oleksyk | e | ● | 4.23 | Europeans |
| 4 | 151,985,548 | 152,954,145 | Oleksyk | e | ● | 4.24 | Europeans |
| 4 | 152,900,000 | 153,000,000 | Voght | . | ● | 4.24 | Africans |
| 4 | 153,400,000 | 153,500,000 | Voght | . | ○ |  | Asians |
| 4 | 156,339,723 | 156,339,723 | Akey | . | ○ |  |  |
| 4 | 157,600,000 | 157,700,000 | Voght | . | ○ |  | Africans |
| 4 | 158,900,000 | 159,100,000 | Hapmap II | . | ● | 4.25 | Asians |
| 4 | 159,100,000 | 159,200,000 | Voght | . | ● | 4.25 | Asians |
| 4 | 159,400,000 | 159,500,000 | Voght | . | ○ |  | Asians |
| 4 | 159,500,000 | 159,600,000 | Voght | . | ○ |  | Asians |
| 4 | 162,500,000 | 162,600,000 | Voght | . | ○ |  | Asians |
| 4 | 163,950,000 | 164,100,000 | Hapmap II | . | ○ |  | Asians |
| 4 | 170,800,000 | 170,900,000 | Voght | . | ○ |  | Asians |
| 4 | 170,900,000 | 171,000,000 | Voght | . | ○ |  | Asians |
| 4 | 171,800,000 | 171,950,000 | Hapmap II | . | ○ |  | Asians |
| 4 | 176,600,000 | 176,750,000 | Hapmap II | . | ○ |  | Europeans |
| 4 | 177,000,000 | 177,100,000 | Voght | . | ○ |  | Asians |
| 4 | 177,589,005 | 177,589,005 | Akey | . | ○ |  |  |
| 4 | 177,613,455 | 177,613,455 | Akey | . | ○ |  |  |
| 4 | 177,628,224 | 177,628,224 | Akey | . | ○ |  |  |
| 4 | 186,800,000 | 186,900,000 | Voght | . | ○ |  | Asians |
| 4 | 186,900,000 | 187,000,000 | Voght | . | ○ |  | Europeans |
| 4 | 190,900,000 | 191,050,000 | Hapmap II | . | ○ |  | Asians |
| 5 | 11,886,256 | 11,893,734 | Sabeti | . | ○ |  | Europeans |
| 5 | 15,200,000 | 15,300,000 | Voght | . | ○ |  | Africans |
| 5 | 16,757,419 | 16,757,419 | Akey | . | ○ |  |  |
| 5 | 21,700,000 | 21,800,000 | Voght | . | ○ |  | Europeans |
| 5 | 21,800,000 | 21,900,000 | Voght | . | ○ |  | Europeans |
| 5 | 21,900,000 | 22,000,000 | Voght | . | ○ |  | Europeans |
| 5 | 22,000,000 | 22,100,000 | Voght | . | ○ |  | Europeans |
| 5 | 24,300,000 | 24,550,000 | Hapmap II | . | ○ |  | Europeans |
| 5 | 26,918,229 | 26,918,229 | Oleksyk | a | ○ |  | Africans |
| 5 | 35,049,032 | 35,049,032 | Akey | . | ○ |  |  |
| 5 | 35,062,335 | 35,062,335 | Akey | . | ○ |  |  |
| 5 | 37,300,000 | 37,400,000 | Voght | . | ○ |  | Africans |
| 5 | 45,307,474 | 45,741,721 | Wang | . | ○ |  | All populations |
| 5 | 54,900,000 | 55,000,000 | Voght | . | ○ |  | Europeans |
| 5 | 64,700,000 | 64,800,000 | Voght | . | ○ |  | Asians |
| 5 | 64,800,000 | 64,900,000 | Voght | . | ○ |  | Asians |
| 5 | 64,850,000 | 65,100,000 | Hapmap II | . | ● | 5.01 | Asians |
| 5 | 64,850,000 | 65,100,000 | Hapmap II | . | ● | 5.02 | Asians |
| 5 | 64,900,000 | 64,900,000 | Voght | . | ● | 5.01 |  |
| 5 | 64,900,000 | 65,000,000 | Voght | . | ● | 5.02 | Asians |
| 5 | 66,800,000 | 66,900,000 | Voght | . | ○ |  | Africans |
| 5 | 66,900,000 | 67,000,000 | Voght | . | ○ |  | Africans |
| 5 | 72,831,125 | 72,836,164 | Oleksyk | b | ○ |  | Europeans |
| 5 | 80,800,000 | 80,900,000 | Voght | . | ○ |  | Asians |
| 5 | 80,900,000 | 81,000,000 | Voght | . | ○ |  | Asians |
| 5 | 82,500,000 | 82,600,000 | Voght | . | ○ |  | Africans |
| 5 | 91,600,000 | 91,700,000 | Voght | . | ○ |  | Asians |
| 5 | 92,937,508 | 93,775,891 | Oleksyk | c | ● | 5.03 | (old) Africans & Europeans |
| 5 | 92,937,508 | 93,775,891 | Oleksyk | c | ● | 5.04 | (old) Africans & Europeans |
| 5 | 92,937,508 | 93,775,891 | Oleksyk | c | ● | 5.05 | (old) Africans & Europeans |
| 5 | 93,200,000 | 93,300,000 | Voght | . | ○ |  | Asians |
| 5 | 93,300,000 | 93,300,000 | Voght | . | ● | 5.03 |  |
| 5 | 93,300,000 | 93,400,000 | Voght | . | ○ |  | Asians |
| 5 | 93,400,000 | 93,400,000 | Voght | . | ● | 5.04 |  |
| 5 | 93,400,000 | 93,500,000 | Voght | . | ● | 5.05 | Europeans |
| 5 | 101,800,000 | 101,900,000 | Voght | . | ○ |  | Africans |
| 5 | 106,893,432 | 106,893,432 | Akey | . | ○ |  |  |
| 5 | 108,500,000 | 108,600,000 | Voght | . | ○ |  | Asians |
| 5 | 109,100,000 | 109,200,000 | Voght | . | ○ |  | Africans |
| 5 | 109,200,000 | 109,300,000 | Voght | . | ○ |  | Africans |
| 5 | 109,300,000 | 109,400,000 | Voght | . | ○ |  | Africans |
| 5 | 109,800,000 | 109,900,000 | Voght | . | ○ |  | Europeans |
| 5 | 109,900,000 | 110,000,000 | Voght | . | ○ |  | Europeans |
| 5 | 110,100,000 | 110,200,000 | Voght | . | ○ |  | Europeans |
| 5 | 110,150,000 | 110,300,000 | Hapmap II | . | ● | 5.06 | Europeans |
| 5 | 110,150,000 | 110,300,000 | Hapmap II | . | ● | 5.07 | Europeans |
| 5 | 110,200,000 | 110,200,000 | Voght | . | ● | 5.06 |  |
| 5 | 110,200,000 | 110,300,000 | Voght | . | ● | 5.07 | Europeans |
| 5 | 112,200,000 | 112,300,000 | Voght | . | ○ |  | Asians |
| 5 | 112,350,000 | 112,550,000 | Hapmap II | . | ○ |  | Europeans |
| 5 | 112,800,000 | 112,900,000 | Voght | . | ○ |  | Asians |
| 5 | 112,900,000 | 113,000,000 | Voght | . | ○ |  | Asians |
| 5 | 117,360,000 | 117,700,000 | Carlson | . | ● | 5.08 |  |
| 5 | 117,381,470 | 117,679,927 | Sabeti | . | ● | 5.08 | Asians |
| 5 | 120,550,000 | 120,950,000 | Hapmap II | . | ○ |  | Asians |
| 5 | 124,257,854 | 124,257,854 | Akey | . | ○ |  |  |
| 5 | 127,700,000 | 127,800,000 | Voght | . | ○ |  | Asians |
| 5 | 129,463,313 | 129,463,313 | Akey | . | ○ |  |  |
| 5 | 132,267,080 | 132,267,080 | Oleksyk | d | ○ |  | Africans |
| 5 | 138,800,000 | 138,900,000 | Voght | . | ○ |  | Europeans |
| 5 | 140,422,393 | 140,459,806 | Oleksyk | e | ○ |  | (old) Africans & Europeans |
| 5 | 141,975,961 | 142,234,863 | Oleksyk | f | ● | 5.09 | Europeans |
| 5 | 141,975,961 | 142,234,863 | Oleksyk | f | ● | 5.10 | Europeans |
| 5 | 142,119,542 | 142,125,869 | Sabeti | . | ● | 5.09 | Europeans |
| 5 | 142,200,000 | 142,300,000 | Voght | . | ● | 5.10 | Europeans |
| 5 | 145,751,178 | 145,751,178 | Akey | . | ○ |  |  |
| 5 | 146,200,000 | 146,300,000 | Voght | . | ○ |  | Europeans |
| 5 | 146,300,000 | 146,400,000 | Voght | . | ○ |  | Europeans |
| 5 | 157,200,000 | 157,300,000 | Voght | . | ○ |  | Europeans |
| 5 | 159,381,093 | 159,393,888 | Oleksyk | g | ○ |  | (old) Africans & Europeans |
| 5 | 160,803,080 | 160,850,531 | Oleksyk | h | ○ |  | (old) Africans & Europeans |
| 5 | 170,400,000 | 170,500,000 | Hapmap II | . | ○ |  | Asians |
| 6 | 2,112,224 | 2,154,576 | Oleksyk | a | ○ |  | (old) Africans & Europeans |
| 6 | 3,020,552 | 3,215,673 | Oleksyk | b | ○ |  | Europeans |
| 6 | 12,027,400 | 12,062,171 | Oleksyk | c | ○ |  | Europeans |
| 6 | 13,100,000 | 13,200,000 | Voght | . | ○ |  | Asians |
| 6 | 15,400,000 | 15,500,000 | Voght | . | ○ |  | Asians |
| 6 | 15,600,000 | 15,700,000 | Voght | . | ○ |  | Europeans |
| 6 | 18,700,000 | 18,850,000 | Hapmap II | . | ○ |  | Asians |
| 6 | 19,000,000 | 19,100,000 | Voght | . | ○ |  | Asians |
| 6 | 22,225,486 | 22,225,486 | Akey | . | ○ |  |  |
| 6 | 23,241,768 | 23,241,768 | Akey | . | ○ |  |  |
| 6 | 23,261,428 | 23,261,428 | Akey | . | ○ |  |  |
| 6 | 23,261,660 | 23,261,660 | Akey | . | ○ |  |  |
| 6 | 26,300,000 | 26,400,000 | Voght | . | ○ |  | Asians |
| 6 | 26,744,497 | 26,767,741 | Wang | . | ○ |  | All populations |
| 6 | 27,500,000 | 27,600,000 | Voght | . | ● | 6.01 | Africans |
| 6 | 27,526,506 | 27,548,858 | Wang | . | ● | 6.01 | All populations |
| 6 | 28,217,695 | 28,231,775 | Wang | . | ○ |  | All populations |
| 6 | 28,301,049 | 28,309,239 | Wang | . | ○ |  | All populations |
| 6 | 28,600,000 | 28,700,000 | Voght | . | ○ |  | Africans |
| 6 | 28,800,000 | 28,900,000 | Voght | . | ○ |  | Africans |
| 6 | 28,900,000 | 29,000,000 | Voght | . | ○ |  | Africans |
| 6 | 28,900,000 | 29,000,000 | Voght | . | ○ |  | Europeans |
| 6 | 30,200,000 | 30,300,000 | Voght | . | ○ |  | Europeans |
| 6 | 31,457,335 | 32,721,719 | Huttley | . | ● | 6.02 |  |
| 6 | 31,767,710 | 31,773,431 | Oleksyk | d | ● | 6.02 | (old) Africans & Europeans |
| 6 | 33,100,000 | 33,200,000 | Voght | . | ○ |  | Africans |
| 6 | 33,550,000 | 33,700,000 | Hapmap II | . | ○ |  | Africans |
| 6 | 34,600,000 | 34,700,000 | Voght | . | ○ |  | Europeans |
| 6 | 34,700,000 | 34,800,000 | Voght | . | ○ |  | Europeans |
| 6 | 34,800,000 | 34,900,000 | Voght | . | ○ |  | Europeans |
| 6 | 35,144,375 | 35,164,421 | Oleksyk | e | ○ |  | Europeans |
| 6 | 39,500,000 | 39,600,000 | Voght | . | ○ |  | Europeans |
| 6 | 39,600,000 | 39,700,000 | Voght | . | ○ |  | Europeans |
| 6 | 39,735,467 | 39,735,467 | Akey | . | ○ |  |  |
| 6 | 42,348,347 | 42,509,060 | Oleksyk | f | ○ |  | Europeans |
| 6 | 43,300,000 | 43,400,000 | Voght | . | ○ |  | Europeans |
| 6 | 44,843,324 | 45,392,525 | Wang | . | ○ |  | All populations |
| 6 | 46,200,000 | 46,300,000 | Voght | . | ○ |  | Europeans |
| 6 | 47,350,000 | 47,850,000 | Hapmap II | . | ○ |  | Asians |
| 6 | 48,200,000 | 48,300,000 | Voght | . | ● | 6.03 | Africans |
| 6 | 48,300,000 | 48,400,000 | Hapmap II | . | ● | 6.03 | Asians |
| 6 | 55,800,000 | 55,900,000 | Voght | . | ○ |  | Asians |
| 6 | 62,386,701 | 62,992,968 | Wang | . | ○ |  | All populations |
| 6 | 63,500,000 | 63,650,000 | Hapmap II | . | ○ |  | Africans |
| 6 | 63,700,000 | 63,800,000 | Voght | . | ○ |  | Africans |
| 6 | 70,100,000 | 70,250,000 | Hapmap II | . | ○ |  | Africans |
| 6 | 71,100,000 | 71,200,000 | Voght | . | ○ |  | Africans |
| 6 | 72,700,000 | 72,800,000 | Voght | . | ○ |  | Africans |
| 6 | 72,800,000 | 72,900,000 | Voght | . | ○ |  | Africans |
| 6 | 74,950,000 | 75,050,000 | Hapmap II | . | ○ |  | Africans |
| 6 | 75,060,000 | 75,360,000 | Carlson | . | ● | 6.04 |  |
| 6 | 75,100,000 | 75,200,000 | Voght | . | ● | 6.04 | Africans |
| 6 | 75,580,000 | 75,890,000 | Carlson | . | ○ |  |  |
| 6 | 77,900,000 | 78,000,000 | Hapmap II | . | ○ |  | Africans |
| 6 | 80,731,567 | 80,732,187 | Oleksyk | g | ○ |  | (old) Africans & Europeans |
| 6 | 81,800,000 | 81,950,000 | Hapmap II | . | ○ |  | Europeans |
| 6 | 83,400,000 | 83,850,000 | Hapmap II | . | ○ |  | Asians |
| 6 | 84,476,512 | 84,904,563 | Oleksyk | h | ● | 6.05 | Europeans |
| 6 | 84,476,512 | 84,904,563 | Oleksyk | h | ● | 6.06 | Europeans |
| 6 | 84,476,512 | 84,904,563 | Oleksyk | h | ● | 6.07 | Europeans |
| 6 | 84,500,000 | 84,800,000 | Carlson | . | ● | 6.05 |  |
| 6 | 84,500,000 | 84,800,000 | Carlson | . | ● | 6.06 |  |
| 6 | 84,800,000 | 85,000,000 | Hapmap II | . | ● | 6.06 | Europeans |
| 6 | 84,800,000 | 85,000,000 | Hapmap II | . | ● | 6.07 | Europeans |
| 6 | 88,274,380 | 88,788,327 | Oleksyk | i | ○ |  | (old) Africans & Europeans |
| 6 | 91,700,000 | 91,800,000 | Voght | . | ○ |  | Europeans |
| 6 | 93,600,000 | 93,700,000 | Voght | . | ○ |  | Asians |
| 6 | 100,800,000 | 100,900,000 | Voght | . | ○ |  | Africans |
| 6 | 101,063,331 | 101,422,632 | Wang | . | ○ |  | All populations |
| 6 | 101,352,483 | 101,374,747 | Wang | . | ○ |  | All populations |
| 6 | 102,200,000 | 102,300,000 | Voght | . | ○ |  | Europeans |
| 6 | 102,300,000 | 102,400,000 | Voght | . | ○ |  | Europeans |
| 6 | 105,600,000 | 105,980,000 | Carlson | . | ○ |  |  |
| 6 | 108,368,909 | 108,811,458 | Oleksyk | j | ○ |  | Europeans |
| 6 | 111,700,000 | 111,800,000 | Voght | . | ○ |  | Asians |
| 6 | 111,851,511 | 111,890,107 | Oleksyk | k | ○ |  | (old) Africans & Europeans |
| 6 | 120,000,000 | 120,100,000 | Voght | . | ○ |  | Europeans |
| 6 | 120,551,678 | 120,965,630 | Oleksyk | l | ○ |  | Africans |
| 6 | 121,200,000 | 121,300,000 | Voght | . | ○ |  | Europeans |
| 6 | 122,400,000 | 122,500,000 | Voght | . | ○ |  | Africans |
| 6 | 122,800,000 | 122,950,000 | Hapmap II | . | ○ |  | Africans |
| 6 | 123,600,000 | 123,700,000 | Voght | . | ○ |  | Asians |
| 6 | 125,800,000 | 125,900,000 | Voght | . | ○ |  | Asians |
| 6 | 125,950,000 | 126,100,000 | Hapmap II | . | ○ |  | Asians |
| 6 | 126,500,000 | 126,600,000 | Voght | . | ○ |  | Africans |
| 6 | 126,650,000 | 127,130,000 | Carlson | . | ● | 6.08 |  |
| 6 | 127,100,000 | 127,200,000 | Voght | . | ● | 6.08 | Africans |
| 6 | 128,672,348 | 128,775,386 | Oleksyk | m | ○ |  | (old) Africans & Europeans |
| 6 | 130,500,000 | 130,600,000 | Voght | . | ○ |  | Africans |
| 6 | 130,550,000 | 130,650,000 | Hapmap II | . | ● | 6.09 | Africans |
| 6 | 130,600,000 | 130,600,000 | Voght | . | ● | 6.09 |  |
| 6 | 130,600,000 | 130,700,000 | Voght | . | ○ |  | Africans |
| 6 | 133,750,297 | 133,759,712 | Oleksyk | n | ○ |  | Europeans |
| 6 | 135,663,041 | 136,424,290 | Sabeti | . | ○ |  | Europeans |
| 6 | 137,362,560 | 137,391,856 | Oleksyk | o | ○ |  | Europeans |
| 6 | 140,500,000 | 140,600,000 | Voght | . | ● | 6.10 | Asians |
| 6 | 140,500,000 | 141,000,000 | Hapmap | . | ● | 6.10 |  |
| 6 | 143,759,436 | 143,759,436 | Akey | . | ○ |  |  |
| 6 | 143,766,464 | 143,766,464 | Akey | . | ○ |  |  |
| 6 | 143,771,617 | 143,771,617 | Akey | . | ○ |  |  |
| 6 | 146,552,787 | 146,571,631 | Oleksyk | p | ○ |  | Europeans |
| 6 | 158,600,000 | 158,700,000 | Voght | . | ○ |  | Africans |
| 6 | 159,933,698 | 159,946,410 | Oleksyk | q | ○ |  | Europeans |
| 6 | 166,122,820 | 166,122,820 | Akey | . | ○ |  |  |
| 6 | 169,600,000 | 169,700,000 | Voght | . | ○ |  | Europeans |
| 6 | 169,870,380 | 169,999,340 | Oleksyk | r | ○ |  | Africans |
| 6 | 177,889,561 | 177,889,561 | Akey | . | ○ |  |  |
| 7 | 5,296,445 | 5,296,445 | Akey | . | ○ |  |  |
| 7 | 10,000,000 | 10,100,000 | Voght | . | ○ |  | Asians |
| 7 | 17,700,000 | 17,800,000 | Voght | . | ○ |  | Africans |
| 7 | 18,000,000 | 18,100,000 | Voght | . | ○ |  | Asians |
| 7 | 20,100,000 | 20,200,000 | Voght | . | ● | 7.01 | Africans |
| 7 | 20,100,000 | 20,250,000 | Hapmap II | . | ● | 7.01 | Africans |
| 7 | 24,716,955 | 24,716,955 | Akey | . | ○ |  |  |
| 7 | 30,600,000 | 30,700,000 | Voght | . | ○ |  | Europeans |
| 7 | 31,092,832 | 31,092,832 | Akey | . | ○ |  |  |
| 7 | 31,290,369 | 31,290,369 | Akey | . | ○ |  |  |
| 7 | 31,600,000 | 31,700,000 | Voght | . | ○ |  | Europeans |
| 7 | 33,768,342 | 33,768,342 | Akey | . | ○ |  |  |
| 7 | 36,480,696 | 36,480,696 | Akey | . | ○ |  |  |
| 7 | 36,600,000 | 36,700,000 | Voght | . | ○ |  | Europeans |
| 7 | 38,034,744 | 44,738,233 | Huttley | . | ● | 7.02 |  |
| 7 | 38,034,744 | 44,738,233 | Huttley | . | ● | 7.03 |  |
| 7 | 38,957,855 | 38,957,855 | Akey | . | ● | 7.02 |  |
| 7 | 39,026,383 | 39,026,383 | Akey | . | ● | 7.03 |  |
| 7 | 56,097,175 | 56,097,175 | Akey | . | ○ |  |  |
| 7 | 64,800,000 | 64,900,000 | Voght | . | ○ |  | Asians |
| 7 | 65,000,000 | 65,100,000 | Voght | . | ○ |  | Asians |
| 7 | 65,300,000 | 65,400,000 | Voght | . | ○ |  | Africans |
| 7 | 65,400,000 | 65,500,000 | Voght | . | ○ |  | Africans |
| 7 | 65,500,000 | 65,600,000 | Voght | . | ○ |  | Asians |
| 7 | 68,137,506 | 69,331,674 | Wang | . | ○ |  | All populations |
| 7 | 68,476,436 | 69,331,674 | Wang | . | ● | 7.05 | All populations |
| 7 | 68,476,436 | 69,331,674 | Wang | . | ● | 7.05 | All populations |
| 7 | 68,476,436 | 69,669,971 | Wang | . | ○ |  | All populations |
| 7 | 68,500,000 | 68,600,000 | Voght | . | ● | 7.05 | Africans |
| 7 | 68,500,000 | 68,600,000 | Voght | . | ● | 7.05 | Europeans |
| 7 | 73,450,000 | 74,750,000 | Hapmap II | . | ○ |  | Asians |
| 7 | 74,817,831 | 74,817,831 | Hapmap II | . | ○ |  | Europeans |
| 7 | 84,700,000 | 84,800,000 | Voght | . | ○ |  | Asians |
| 7 | 84,800,000 | 84,900,000 | Voght | . | ○ |  | Asians |
| 7 | 85,600,000 | 85,700,000 | Voght | . | ○ |  | Asians |
| 7 | 85,900,000 | 86,000,000 | Voght | . | ○ |  | Asians |
| 7 | 86,000,000 | 86,100,000 | Voght | . | ○ |  | Asians |
| 7 | 88,000,000 | 88,100,000 | Hapmap II | . | ○ |  | Africans |
| 7 | 89,900,000 | 90,000,000 | Voght | . | ○ |  | Africans |
| 7 | 90,528,622 | 90,697,530 | Wang | . | ○ |  | All populations |
| 7 | 91,182,221 | 91,352,018 | Wang | . | ○ |  | All populations |
| 7 | 91,519,405 | 91,672,914 | Wang | . | ○ |  | All populations |
| 7 | 91,700,000 | 91,800,000 | Voght | . | ○ |  | Africans |
| 7 | 92,264,101 | 92,390,953 | Oleksyk | a | ○ |  | Africans |
| 7 | 97,500,000 | 97,600,000 | Voght | . | ○ |  | Europeans |
| 7 | 98,290,168 | 99,123,455 | Oleksyk | b | ● | 7.06 | Europeans |
| 7 | 98,290,168 | 99,123,455 | Oleksyk | b | ● | 7.07 | Europeans |
| 7 | 98,290,168 | 99,123,455 | Oleksyk | b | ● | 7.08 | Europeans |
| 7 | 98,460,000 | 99,080,000 | Carlson | . | ● | 7.06 |  |
| 7 | 98,460,000 | 99,080,000 | Carlson | . | ● | 7.07 |  |
| 7 | 98,460,000 | 99,080,000 | Carlson | . | ● | 7.08 |  |
| 7 | 98,600,000 | 98,700,000 | Voght | . | ● | 7.07 | Europeans |
| 7 | 102,000,000 | 102,100,000 | Voght | . | ○ |  | Europeans |
| 7 | 102,100,000 | 102,200,000 | Voght | . | ○ |  | Europeans |
| 7 | 103,854,954 | 103,854,954 | Oleksyk | c | ○ |  | (old) Africans & Europeans |
| 7 | 104,450,000 | 104,550,000 | Hapmap II | . | ○ |  | Europeans |
| 7 | 105,600,000 | 105,750,000 | Hapmap II | . | ○ |  | Asians |
| 7 | 106,800,392 | 106,800,392 | Akey | . | ○ |  |  |
| 7 | 109,302,987 | 109,603,469 | Wang | . | ○ |  | All populations |
| 7 | 109,863,641 | 110,164,152 | Wang | . | ○ |  | All populations |
| 7 | 110,300,000 | 110,400,000 | Voght | . | ○ |  | Europeans |
| 7 | 110,400,000 | 110,500,000 | Voght | . | ○ |  | Africans |
| 7 | 111,700,000 | 111,800,000 | Voght | . | ○ |  | Asians |
| 7 | 111,750,000 | 111,950,000 | Hapmap II | . | ● | 7.09 | Asians |
| 7 | 111,750,000 | 111,950,000 | Hapmap II | . | ● | 7.10 | Asians |
| 7 | 111,800,000 | 111,800,000 | Voght | . | ● | 7.09 |  |
| 7 | 111,800,000 | 111,900,000 | Voght | . | ○ |  | Asians |
| 7 | 111,900,000 | 111,900,000 | Voght | . | ● | 7.10 |  |
| 7 | 111,900,000 | 112,000,000 | Voght | . | ○ |  | Asians |
| 7 | 113,526,800 | 114,206,060 | Oleksyk | d | ○ |  | (old) Africans & Europeans |
| 7 | 116,600,000 | 116,700,000 | Voght | . | ○ |  | Africans |
| 7 | 117,453,562 | 117,453,562 | Akey | . | ○ |  |  |
| 7 | 117,500,000 | 117,600,000 | Voght | . | ○ |  | Africans |
| 7 | 117,600,000 | 117,700,000 | Voght | . | ○ |  | Africans |
| 7 | 118,000,000 | 118,100,000 | Voght | . | ○ |  | Africans |
| 7 | 118,600,000 | 118,700,000 | Voght | . | ○ |  | Africans |
| 7 | 119,182,999 | 119,182,999 | Akey | . | ○ |  |  |
| 7 | 119,456,534 | 128,796,639 | Huttley | . | ● | 7.11 |  |
| 7 | 119,456,534 | 128,796,639 | Huttley | . | ● | 7.12 |  |
| 7 | 119,456,534 | 128,796,639 | Huttley | . | ● | 7.13 |  |
| 7 | 119,456,534 | 128,796,639 | Huttley | . | ● | 7.14 |  |
| 7 | 119,456,534 | 128,796,639 | Huttley | . | ● | 7.15 |  |
| 7 | 119,456,534 | 128,796,639 | Huttley | . | ● | 7.16 |  |
| 7 | 119,456,534 | 128,796,639 | Huttley | . | ● | 7.17 |  |
| 7 | 119,900,000 | 120,000,000 | Voght | . | ● | 7.11 | Europeans |
| 7 | 121,722,275 | 121,741,319 | Oleksyk | e | ● | 7.12 | (old) Africans & Europeans |
| 7 | 124,100,000 | 124,250,000 | Hapmap II | . | ● | 7.13 | Europeans |
| 7 | 124,100,000 | 124,250,000 | Hapmap II | . | ● | 7.14 | Europeans |
| 7 | 124,124,434 | 124,339,791 | Wang | . | ● | 7.14 | All populations |
| 7 | 124,124,434 | 124,339,791 | Wang | . | ● | 7.15 | All populations |
| 7 | 126,400,000 | 126,500,000 | Voght | . | ● | 7.16 | Asians |
| 7 | 126,700,000 | 126,800,000 | Voght | . | ● | 7.17 | Asians |
| 7 | 128,842,048 | 128,842,048 | Akey | . | ○ |  |  |
| 7 | 129,092,612 | 129,119,783 | Oleksyk | f | ○ |  | (old) Africans & Europeans |
| 7 | 131,539,382 | 131,668,131 | Oleksyk | g | ○ |  | Europeans |
| 7 | 133,000,000 | 133,100,000 | Voght | . | ○ |  | Africans |
| 7 | 133,200,000 | 133,300,000 | Voght | . | ○ |  | Asians |
| 7 | 139,833,618 | 140,814,606 | Oleksyk | h | ○ |  | (new) Africans & Europeans |
| 7 | 141,500,000 | 142,150,000 | Hapmap II | . | ● | 7.18 | Asians & Africa |
| 7 | 142,030,000 | 142,360,000 | Carlson | . | ● | 7.18 |  |
| 7 | 146,821,391 | 146,821,391 | Akey | . | ○ |  |  |
| 8 | 9,400,000 | 9,500,000 | Voght | . | ○ |  | Africans |
| 8 | 9,500,000 | 9,500,000 | Voght | . | ● | 8.01 |  |
| 8 | 9,500,000 | 9,600,000 | Voght | . | ○ |  | Africans |
| 8 | 9,500,000 | 9,900,000 | Hapmap II | . | ● | 8.01 | Asians & Africa |
| 8 | 9,500,000 | 9,900,000 | Hapmap II | . | ● | 8.02 | Asians & Africa |
| 8 | 9,500,000 | 9,900,000 | Hapmap II | . | ● | 8.03 | Asians & Africa |
| 8 | 9,600,000 | 9,600,000 | Voght | . | ● | 8.02 |  |
| 8 | 9,600,000 | 9,700,000 | Voght | . | ● | 8.03 | Africans |
| 8 | 11,000,000 | 11,100,000 | Voght | . | ○ |  | Asians |
| 8 | 11,200,000 | 11,300,000 | Hapmap II | . | ● | 8.04 | Asians |
| 8 | 11,200,000 | 11,300,000 | Voght | . | ● | 8.04 | Asians |
| 8 | 11,629,989 | 11,629,989 | Akey | . | ○ |  |  |
| 8 | 27,372,852 | 27,372,852 | Akey | . | ○ |  |  |
| 8 | 27,375,802 | 27,375,802 | Akey | . | ○ |  |  |
| 8 | 30,728,620 | 30,998,749 | Oleksyk | a | ○ |  | Europeans |
| 8 | 31,800,000 | 31,900,000 | Voght | . | ○ |  | Asians |
| 8 | 31,900,000 | 32,000,000 | Voght | . | ○ |  | Asians |
| 8 | 42,184,151 | 43,074,597 | Oleksyk | b | ● | 8.05 | Europeans |
| 8 | 42,500,000 | 42,600,000 | Voght | . | ● | 8.05 | Asians |
| 8 | 48,100,000 | 48,200,000 | Voght | . | ○ |  | Asians |
| 8 | 48,223,498 | 48,698,433 | Wang | . | ○ |  | All populations |
| 8 | 49,100,000 | 49,200,000 | Voght | . | ○ |  | Africans |
| 8 | 50,100,000 | 50,200,000 | Voght | . | ● | 8.06 | Europeans |
| 8 | 50,150,493 | 50,150,619 | Wang | . | ● | 8.06 | All populations |
| 8 | 50,200,000 | 50,300,000 | Voght | . | ● | 8.07 | Africans |
| 8 | 50,300,000 | 50,400,000 | Hapmap II | . | ● | 8.07 | Africans |
| 8 | 50,580,000 | 51,170,000 | Carlson | . | ● | 8.08 |  |
| 8 | 50,580,000 | 51,170,000 | Carlson | . | ● | 8.09 |  |
| 8 | 50,580,000 | 51,170,000 | Carlson | . | ● | 8.10 |  |
| 8 | 50,580,000 | 51,170,000 | Carlson | . | ● | 8.11 |  |
| 8 | 50,600,000 | 50,700,000 | Voght | . | ○ |  | Africans |
| 8 | 50,700,000 | 50,700,000 | Voght | . | ● | 8.08 |  |
| 8 | 50,700,000 | 50,800,000 | Voght | . | ● | 8.09 | Africans |
| 8 | 50,874,299 | 51,756,638 | Wang | . | ● | 8.10 | All populations |
| 8 | 50,874,299 | 51,756,638 | Wang | . | ● | 8.11 | All populations |
| 8 | 50,874,299 | 51,756,638 | Wang | . | ● | 8.12 | All populations |
| 8 | 50,874,299 | 51,756,638 | Wang | . | ● | 8.14 | All populations |
| 8 | 50,874,299 | 51,756,638 | Wang | . | ● | 8.14 | All populations |
| 8 | 50,874,299 | 51,756,638 | Wang | . | ● | 8.15 | All populations |
| 8 | 50,874,299 | 51,756,638 | Wang | . | ● | 8.16 | All populations |
| 8 | 50,874,299 | 51,756,638 | Wang | . | ● | 8.17 | All populations |
| 8 | 50,874,299 | 51,756,638 | Wang | . | ● | 8.18 | All populations |
| 8 | 50,874,299 | 51,756,638 | Wang | . | ● | 8.19 | All populations |
| 8 | 50,874,299 | 51,756,638 | Wang | . | ● | 8.20 | All populations |
| 8 | 50,874,299 | 51,756,638 | Wang | . | ● | 8.21 | All populations |
| 8 | 50,874,299 | 51,756,638 | Wang | . | ● | 8.22 | All populations |
| 8 | 50,874,299 | 51,756,638 | Wang | . | ● | 8.23 | All populations |
| 8 | 51,050,000 | 52,150,000 | Hapmap II | . | ● | 8.11 | Europeans & Asians |
| 8 | 51,050,000 | 52,150,000 | Hapmap II | . | ● | 8.12 | Europeans & Asians |
| 8 | 51,050,000 | 52,150,000 | Hapmap II | . | ● | 8.14 | Europeans & Asians |
| 8 | 51,050,000 | 52,150,000 | Hapmap II | . | ● | 8.14 | Europeans & Asians |
| 8 | 51,050,000 | 52,150,000 | Hapmap II | . | ● | 8.15 | Europeans & Asians |
| 8 | 51,050,000 | 52,150,000 | Hapmap II | . | ● | 8.16 | Europeans & Asians |
| 8 | 51,050,000 | 52,150,000 | Hapmap II | . | ● | 8.17 | Europeans & Asians |
| 8 | 51,050,000 | 52,150,000 | Hapmap II | . | ● | 8.18 | Europeans & Asians |
| 8 | 51,050,000 | 52,150,000 | Hapmap II | . | ● | 8.19 | Europeans & Asians |
| 8 | 51,050,000 | 52,150,000 | Hapmap II | . | ● | 8.20 | Europeans & Asians |
| 8 | 51,050,000 | 52,150,000 | Hapmap II | . | ● | 8.21 | Europeans & Asians |
| 8 | 51,050,000 | 52,150,000 | Hapmap II | . | ● | 8.22 | Europeans & Asians |
| 8 | 51,050,000 | 52,150,000 | Hapmap II | . | ● | 8.23 | Europeans & Asians |
| 8 | 51,050,000 | 52,150,000 | Hapmap II | . | ● | 8.24 | Europeans & Asians |
| 8 | 51,050,000 | 52,150,000 | Hapmap II | . | ● | 8.25 | Europeans & Asians |
| 8 | 51,050,000 | 52,150,000 | Hapmap II | . | ● | 8.26 | Europeans & Asians |
| 8 | 51,200,000 | 51,300,000 | Voght | . | ○ |  | Europeans |
| 8 | 51,200,000 | 51,300,000 | Voght | . | ○ |  | Asians |
| 8 | 51,300,000 | 51,300,000 | Voght | . | ● | 8.14 |  |
| 8 | 51,300,000 | 51,300,000 | Voght | . | ● | 8.14 |  |
| 8 | 51,300,000 | 51,400,000 | Voght | . | ○ |  | Asians |
| 8 | 51,400,000 | 51,400,000 | Voght | . | ● | 8.16 |  |
| 8 | 51,400,000 | 51,500,000 | Voght | . | ○ |  | Europeans |
| 8 | 51,500,000 | 51,500,000 | Voght | . | ● | 8.18 |  |
| 8 | 51,500,000 | 51,600,000 | Voght | . | ○ |  | Europeans |
| 8 | 51,600,000 | 51,600,000 | Voght | . | ● | 8.20 |  |
| 8 | 51,600,000 | 51,700,000 | Voght | . | ○ |  | Europeans |
| 8 | 51,700,000 | 51,700,000 | Voght | . | ● | 8.22 |  |
| 8 | 51,700,000 | 51,800,000 | Voght | . | ○ |  | Europeans |
| 8 | 51,800,000 | 51,800,000 | Voght | . | ● | 8.24 |  |
| 8 | 51,800,000 | 51,900,000 | Voght | . | ○ |  | Europeans |
| 8 | 51,900,000 | 51,900,000 | Voght | . | ● | 8.25 |  |
| 8 | 51,900,000 | 52,000,000 | Voght | . | ● | 8.26 | Europeans |
| 8 | 52,400,000 | 52,500,000 | Voght | . | ○ |  | Europeans |
| 8 | 52,500,000 | 52,600,000 | Voght | . | ○ |  | Europeans |
| 8 | 52,600,000 | 52,600,000 | Voght | . | ● | 8.27 |  |
| 8 | 52,600,000 | 52,700,000 | Voght | . | ● | 8.28 | Europeans |
| 8 | 52,600,000 | 53,050,000 | Hapmap II | . | ● | 8.27 | Europeans |
| 8 | 52,600,000 | 53,050,000 | Hapmap II | . | ● | 8.28 | Europeans |
| 8 | 52,600,000 | 53,050,000 | Hapmap II | . | ● | 8.29 | Europeans |
| 8 | 52,600,000 | 53,050,000 | Hapmap II | . | ● | 8.30 | Europeans |
| 8 | 52,800,000 | 52,900,000 | Voght | . | ○ |  | Europeans |
| 8 | 52,900,000 | 52,900,000 | Voght | . | ● | 8.29 |  |
| 8 | 52,900,000 | 53,000,000 | Voght | . | ● | 8.30 | Europeans |
| 8 | 53,176,789 | 53,176,789 | Akey | . | ○ |  |  |
| 8 | 62,200,000 | 62,300,000 | Voght | . | ○ |  | Africans |
| 8 | 64,300,000 | 64,400,000 | Voght | . | ○ |  | Africans |
| 8 | 67,382,282 | 67,520,244 | Oleksyk | c | ○ |  | Europeans |
| 8 | 67,650,000 | 68,280,000 | Carlson | . | ○ |  |  |
| 8 | 71,229,922 | 71,297,215 | Oleksyk | d | ○ |  | (old) Africans & Europeans |
| 8 | 71,900,000 | 72,000,000 | Voght | . | ○ |  | Europeans |
| 8 | 76,500,000 | 76,600,000 | Voght | . | ○ |  | Asians |
| 8 | 76,900,000 | 77,000,000 | Voght | . | ○ |  | Africans |
| 8 | 79,100,000 | 79,200,000 | Voght | . | ○ |  | Africans |
| 8 | 81,400,000 | 81,500,000 | Voght | . | ○ |  | Europeans |
| 8 | 93,800,000 | 93,900,000 | Voght | . | ○ |  | Africans |
| 8 | 95,800,000 | 95,900,000 | Voght | . | ○ |  | Asians |
| 8 | 99,982,077 | 100,846,391 | Wang | . | ○ |  | All populations |
| 8 | 102,500,000 | 102,600,000 | Voght | . | ○ |  | Europeans |
| 8 | 104,200,000 | 104,300,000 | Voght | . | ○ |  | Africans |
| 8 | 104,500,000 | 104,600,000 | Voght | . | ○ |  | Africans |
| 8 | 104,600,000 | 104,700,000 | Voght | . | ○ |  | Africans |
| 8 | 104,700,000 | 104,800,000 | Voght | . | ○ |  | Africans |
| 8 | 109,100,000 | 109,200,000 | Voght | . | ○ |  | Africans |
| 8 | 109,535,107 | 109,535,107 | Akey | . | ○ |  |  |
| 8 | 110,651,542 | 110,676,140 | Oleksyk | e | ○ |  | (old) Africans & Europeans |
| 8 | 111,300,000 | 111,400,000 | Voght | . | ○ |  | Africans |
| 8 | 111,500,000 | 111,600,000 | Voght | . | ○ |  | Europeans |
| 8 | 111,900,000 | 112,050,000 | Hapmap II | . | ● | 8.31 | Europeans |
| 8 | 112,000,000 | 112,100,000 | Voght | . | ● | 8.31 | Europeans |
| 8 | 112,800,000 | 112,900,000 | Voght | . | ○ |  | Asians |
| 8 | 112,900,000 | 113,000,000 | Voght | . | ○ |  | Asians |
| 8 | 113,191,740 | 114,405,825 | Wang | . | ● | 8.32 | All populations |
| 8 | 113,191,740 | 114,405,825 | Wang | . | ● | 8.33 | All populations |
| 8 | 113,191,740 | 114,405,825 | Wang | . | ● | 8.34 | All populations |
| 8 | 113,191,740 | 114,405,825 | Wang | . | ● | 8.35 | All populations |
| 8 | 113,191,740 | 114,405,825 | Wang | . | ● | 8.36 | All populations |
| 8 | 113,191,740 | 114,405,825 | Wang | . | ● | 8.37 | All populations |
| 8 | 113,200,000 | 113,300,000 | Voght | . | ● | 8.32 | Asians |
| 8 | 113,400,000 | 113,500,000 | Voght | . | ○ |  | Asians |
| 8 | 113,500,000 | 113,500,000 | Voght | . | ● | 8.33 |  |
| 8 | 113,500,000 | 113,600,000 | Voght | . | ● | 8.34 | Asians |
| 8 | 113,700,000 | 113,800,000 | Voght | . | ● | 8.35 | Africans |
| 8 | 114,100,000 | 114,200,000 | Voght | . | ● | 8.36 | Europeans |
| 8 | 114,400,000 | 114,500,000 | Voght | . | ● | 8.37 | Africans |
| 8 | 121,371,917 | 121,371,917 | Akey | . | ○ |  |  |
| 8 | 129,600,000 | 129,700,000 | Voght | . | ○ |  | Asians |
| 8 | 133,800,000 | 133,900,000 | Voght | . | ○ |  | Africans |
| 8 | 139,500,000 | 139,600,000 | Voght | . | ○ |  | Europeans |
| 9 | 6,421,091 | 6,428,131 | Oleksyk | a | ○ |  | (old) Africans & Europeans |
| 9 | 11,800,000 | 11,900,000 | Hapmap II | . | ○ |  | Africans |
| 9 | 12,500,000 | 12,600,000 | Voght | . | ○ |  | Europeans |
| 9 | 12,600,000 | 12,600,000 | Voght | . | ● | 9.01 |  |
| 9 | 12,600,000 | 12,700,000 | Hapmap II | . | ● | 9.01 | Europeans |
| 9 | 12,600,000 | 12,700,000 | Hapmap II | . | ● | 9.02 | Europeans |
| 9 | 12,600,000 | 12,700,000 | Voght | . | ● | 9.02 | Europeans |
| 9 | 15,700,000 | 15,800,000 | Voght | . | ○ |  | Asians |
| 9 | 15,900,000 | 16,000,000 | Voght | . | ○ |  | Asians |
| 9 | 20,218,453 | 20,399,801 | Oleksyk | b | ○ |  | Europeans |
| 9 | 23,200,000 | 23,300,000 | Voght | . | ○ |  | Asians |
| 9 | 24,100,000 | 24,200,000 | Voght | . | ○ |  | Africans |
| 9 | 24,300,000 | 24,400,000 | Voght | . | ○ |  | Africans |
| 9 | 24,350,000 | 24,450,000 | Hapmap II | . | ● | 9.03 | Africans |
| 9 | 24,400,000 | 24,400,000 | Voght | . | ● | 9.03 |  |
| 9 | 24,400,000 | 24,500,000 | Voght | . | ○ |  | Africans |
| 9 | 26,700,000 | 26,800,000 | Voght | . | ○ |  | Asians |
| 9 | 26,800,000 | 26,900,000 | Voght | . | ○ |  | Asians |
| 9 | 26,900,000 | 27,000,000 | Voght | . | ○ |  | Asians |
| 9 | 33,800,000 | 33,900,000 | Voght | . | ○ |  | Europeans |
| 9 | 34,609,279 | 34,609,279 | Akey | . | ○ |  |  |
| 9 | 37,800,000 | 37,900,000 | Voght | . | ○ |  | Europeans |
| 9 | 40,500,696 | 40,503,974 | Wang | . | ○ |  | All populations |
| 9 | 42,850,000 | 44,200,000 | Hapmap II | . | ○ |  | All populations |
| 9 | 64,250,000 | 64,450,000 | Hapmap II | . | ○ |  | Europeans |
| 9 | 67,500,000 | 67,600,000 | Voght | . | ○ |  | Asians |
| 9 | 68,050,000 | 68,250,000 | Hapmap II | . | ○ |  | Asians |
| 9 | 71,579,556 | 71,584,739 | Oleksyk | c | ○ |  | (old) Africans & Europeans |
| 9 | 74,655,011 | 74,864,521 | Oleksyk | d | ○ |  | (old) Africans & Europeans |
| 9 | 77,200,000 | 77,300,000 | Voght | . | ○ |  | Africans |
| 9 | 87,200,000 | 87,300,000 | Voght | . | ○ |  | Africans |
| 9 | 87,900,000 | 88,050,000 | Hapmap II | . | ○ |  | Europeans |
| 9 | 89,600,000 | 89,700,000 | Voght | . | ○ |  | Europeans |
| 9 | 90,599,008 | 90,639,663 | Wang | . | ○ |  | All populations |
| 9 | 91,774,252 | 91,774,252 | Akey | . | ○ |  |  |
| 9 | 92,442,094 | 92,457,001 | Wang | . | ○ |  | All populations |
| 9 | 93,202,746 | 93,421,393 | Wang | . | ○ |  | All populations |
| 9 | 96,000,000 | 96,100,000 | Voght | . | ○ |  | Africans |
| 9 | 96,180,190 | 96,333,949 | Oleksyk | e | ● | 9.04 | (old) Africans & Europeans |
| 9 | 96,333,572 | 96,333,572 | Akey | . | ● | 9.04 |  |
| 9 | 97,200,000 | 97,300,000 | Voght | . | ● | 9.05 | Africans |
| 9 | 97,267,362 | 97,267,362 | Akey | . | ● | 9.05 |  |
| 9 | 97,700,000 | 97,850,000 | Hapmap II | . | ○ |  | Africans |
| 9 | 97,900,000 | 98,000,000 | Voght | . | ○ |  | Europeans |
| 9 | 98,400,000 | 98,500,000 | Voght | . | ○ |  | Asians |
| 9 | 98,600,000 | 98,700,000 | Voght | . | ○ |  | Africans |
| 9 | 103,300,000 | 103,400,000 | Voght | . | ○ |  | Europeans |
| 9 | 103,900,000 | 104,000,000 | Hapmap II | . | ○ |  | Asians |
| 9 | 104,300,000 | 104,400,000 | Voght | . | ○ |  | Asians |
| 9 | 105,305,857 | 105,427,693 | Oleksyk | f | ○ |  | (old) Africans & Europeans |
| 9 | 106,500,000 | 106,600,000 | Voght | . | ○ |  | Asians |
| 9 | 106,600,000 | 106,700,000 | Voght | . | ○ |  | Asians |
| 9 | 107,100,000 | 107,200,000 | Voght | . | ● | 9.06 | Europeans |
| 9 | 107,100,000 | 107,200,000 | Voght | . | ● | 9.06 | Asians |
| 9 | 107,136,294 | 107,179,093 | Oleksyk | g | ● | 9.06 | (old) Africans & Europeans |
| 9 | 107,200,000 | 107,300,000 | Voght | . | ○ |  | Europeans |
| 9 | 107,200,000 | 107,300,000 | Voght | . | ○ |  | Asians |
| 9 | 108,250,000 | 108,400,000 | Hapmap II | . | ○ |  | Asians |
| 9 | 112,297,732 | 112,301,693 | Oleksyk | h | ○ |  | (old) Africans & Europeans |
| 9 | 113,600,000 | 113,700,000 | Voght | . | ○ |  | Africans |
| 9 | 115,735,767 | 115,735,767 | Akey | . | ○ |  |  |
| 9 | 115,848,385 | 115,848,385 | Akey | . | ○ |  |  |
| 9 | 118,600,000 | 118,700,000 | Voght | . | ○ |  | Africans |
| 9 | 119,909,962 | 119,909,962 | Akey | . | ○ |  |  |
| 9 | 121,000,000 | 121,100,000 | Voght | . | ○ |  | Africans |
| 9 | 121,100,000 | 121,200,000 | Voght | . | ○ |  | Africans |
| 9 | 121,700,000 | 121,800,000 | Voght | . | ○ |  | Europeans |
| 9 | 121,800,000 | 121,900,000 | Voght | . | ○ |  | Europeans |
| 9 | 121,800,000 | 121,900,000 | Voght | . | ○ |  | Asians |
| 9 | 121,900,000 | 122,000,000 | Voght | . | ○ |  | Asians |
| 9 | 122,000,000 | 122,100,000 | Voght | . | ○ |  | Asians |
| 9 | 122,326,347 | 123,217,419 | Oleksyk | i | ○ |  | Europeans |
| 9 | 124,354,405 | 124,372,041 | Oleksyk | j | ○ |  | Europeans |
| 9 | 124,816,983 | 124,816,983 | Akey | . | ○ |  |  |
| 9 | 126,400,000 | 126,500,000 | Voght | . | ○ |  | Europeans |
| 9 | 126,600,000 | 126,700,000 | Voght | . | ○ |  | Europeans |
| 9 | 127,200,000 | 127,300,000 | Voght | . | ○ |  | Europeans |
| 9 | 127,900,000 | 128,200,000 | Hapmap II | . | ○ |  | Europeans & Asians |
| 9 | 129,800,000 | 129,900,000 | Voght | . | ○ |  | Asians |
| 9 | 135,100,000 | 135,200,000 | Voght | . | ○ |  | Africans |
| 9 | 137,000,000 | 137,150,000 | Hapmap II | . | ○ |  | Africans |
| 10 | . | . | Hapmap | . | ○ |  |  |
| 10 | 2,950,000 | 3,100,000 | Hapmap II | . | ● | 10.01 | Europeans & Asians |
| 10 | 2,986,576 | 2,988,247 | Sabeti | . | ● | 10.01 | Europeans |
| 10 | 5,774,554 | 5,774,554 | Akey | . | ○ |  |  |
| 10 | 6,800,000 | 6,900,000 | Voght | . | ○ |  | Europeans |
| 10 | 10,800,000 | 10,900,000 | Voght | . | ○ |  | Africans |
| 10 | 11,000,000 | 11,150,000 | Hapmap II | . | ○ |  | Africans |
| 10 | 15,937,275 | 15,937,275 | Akey | . | ○ |  |  |
| 10 | 18,923,671 | 18,923,671 | Oleksyk | a | ○ |  | (old) Africans & Europeans |
| 10 | 22,642,019 | 22,798,204 | Sabeti | . | ○ |  | Europeans & Asians |
| 10 | 23,500,000 | 23,600,000 | Voght | . | ○ |  | Asians |
| 10 | 31,600,000 | 31,700,000 | Voght | . | ○ |  | Africans |
| 10 | 31,700,000 | 31,800,000 | Voght | . | ● | 10.02 | Africans |
| 10 | 31,733,523 | 31,842,656 | Oleksyk | b | ● | 10.02 | Europeans |
| 10 | 32,860,657 | 33,175,796 | Wang | . | ● | 10.03 | All populations |
| 10 | 33,100,000 | 33,200,000 | Voght | . | ● | 10.03 | Asians |
| 10 | 35,221,302 | 35,221,302 | Oleksyk | c | ○ |  | Africans |
| 10 | 37,886,527 | 38,273,379 | Oleksyk | d | ○ |  | (old) Africans & Europeans |
| 10 | 38,387,270 | 38,449,193 | Wang | . | ○ |  | All populations |
| 10 | 42,900,000 | 43,000,000 | Voght | . | ○ |  | Asians |
| 10 | 49,976,028 | 50,282,315 | Oleksyk | e | ○ |  | (old) Africans & Europeans |
| 10 | 51,093,663 | 51,093,663 | Akey | . | ○ |  |  |
| 10 | 55,300,000 | 55,400,000 | Voght | . | ○ |  | Asians |
| 10 | 55,400,000 | 55,500,000 | Voght | . | ○ |  | Asians |
| 10 | 55,541,277 | 55,543,799 | Sabeti | . | ○ |  | Asians |
| 10 | 55,600,000 | 55,750,000 | Hapmap II | . | ○ |  | Asians |
| 10 | 58,900,000 | 59,000,000 | Voght | . | ○ |  | Europeans |
| 10 | 59,300,000 | 59,400,000 | Voght | . | ○ |  | Europeans |
| 10 | 60,700,000 | 60,850,000 | Hapmap II | . | ○ |  | Europeans |
| 10 | 65,100,000 | 65,200,000 | Voght | . | ○ |  | Europeans |
| 10 | 68,400,000 | 68,500,000 | Voght | . | ○ |  | Europeans |
| 10 | 68,630,000 | 68,930,000 | Carlson | . | ○ |  |  |
| 10 | 70,254,237 | 70,280,215 | Oleksyk | f | ○ |  | (old) Africans & Europeans |
| 10 | 72,483,368 | 72,483,368 | Akey | . | ○ |  |  |
| 10 | 73,600,000 | 73,700,000 | Voght | . | ○ |  | Europeans |
| 10 | 73,700,000 | 73,800,000 | Voght | . | ○ |  | Europeans |
| 10 | 74,000,000 | 75,250,000 | Hapmap | . | ● | 10.04 |  |
| 10 | 74,000,000 | 75,250,000 | Hapmap | . | ● | 10.05 |  |
| 10 | 74,000,000 | 75,250,000 | Hapmap | . | ● | 10.06 |  |
| 10 | 74,000,000 | 75,250,000 | Hapmap | . | ● | 10.07 |  |
| 10 | 74,000,000 | 75,250,000 | Hapmap | . | ● | 10.08 |  |
| 10 | 74,000,000 | 75,250,000 | Hapmap | . | ● | 10.09 |  |
| 10 | 74,000,000 | 75,250,000 | Hapmap | . | ● | 10.10 |  |
| 10 | 74,336,071 | 74,664,519 | Oleksyk | g | ● | 10.04 | Europeans |
| 10 | 74,336,071 | 74,664,519 | Oleksyk | g | ● | 10.05 | Europeans |
| 10 | 74,340,000 | 75,090,000 | Carlson | . | ● | 10.05 |  |
| 10 | 74,340,000 | 75,090,000 | Carlson | . | ● | 10.06 |  |
| 10 | 74,340,000 | 75,090,000 | Carlson | . | ● | 10.07 |  |
| 10 | 74,340,000 | 75,090,000 | Carlson | . | ● | 10.08 |  |
| 10 | 74,800,000 | 74,900,000 | Voght | . | ● | 10.07 | Europeans |
| 10 | 75,102,484 | 75,224,380 | Wang | . | ● | 10.09 | All populations |
| 10 | 75,226,127 | 75,255,181 | Wang | . | ● | 10.10 | All populations |
| 10 | 75,255,568 | 75,813,663 | Wang | . | ● | 10.11 | All populations |
| 10 | 75,500,000 | 75,600,000 | Voght | . | ● | 10.11 | Asians |
| 10 | 83,300,000 | 83,400,000 | Voght | . | ○ |  | Europeans |
| 10 | 84,000,000 | 84,100,000 | Hapmap II | . | ○ |  | Europeans |
| 10 | 86,500,000 | 86,600,000 | Voght | . | ○ |  | Africans |
| 10 | 87,773,257 | 87,788,988 | Oleksyk | h | ○ |  | (old) Africans & Europeans |
| 10 | 93,473,959 | 93,715,421 | Wang | . | ● | 10.12 | All populations |
| 10 | 93,473,959 | 93,715,421 | Wang | . | ● | 10.13 | All populations |
| 10 | 93,548,281 | 93,548,281 | Akey | . | ● | 10.12 |  |
| 10 | 93,600,000 | 93,700,000 | Voght | . | ● | 10.13 | Europeans |
| 10 | 94,200,000 | 94,300,000 | Voght | . | ○ |  | Asians |
| 10 | 94,300,000 | 94,400,000 | Voght | . | ○ |  | Asians |
| 10 | 94,500,000 | 94,600,000 | Voght | . | ○ |  | Asians |
| 10 | 94,950,000 | 95,050,000 | Hapmap II | . | ○ |  | Asians |
| 10 | 99,883,462 | 100,660,206 | Wang | . | ● | 10.14 | All populations |
| 10 | 100,500,000 | 100,600,000 | Voght | . | ○ |  | Europeans |
| 10 | 100,600,000 | 100,600,000 | Voght | . | ● | 10.14 |  |
| 10 | 100,600,000 | 100,700,000 | Voght | . | ○ |  | Asians |
| 10 | 101,800,000 | 101,900,000 | Voght | . | ○ |  | Africans |
| 10 | 101,900,000 | 102,000,000 | Voght | . | ○ |  | Africans |
| 10 | 102,200,000 | 102,400,000 | Hapmap II | . | ○ |  | Africans |
| 10 | 103,300,000 | 103,400,000 | Voght | . | ○ |  | Asians |
| 10 | 104,512,499 | 104,617,603 | Wang | . | ○ |  | All populations |
| 10 | 106,900,000 | 107,000,000 | Voght | . | ○ |  | Asians |
| 10 | 107,000,000 | 107,100,000 | Voght | . | ○ |  | Asians |
| 10 | 107,250,000 | 107,350,000 | Hapmap II | . | ○ |  | Asians |
| 10 | 109,400,000 | 109,500,000 | Voght | . | ○ |  | Asians |
| 10 | 109,650,000 | 109,800,000 | Hapmap II | . | ● | 10.15 | Asians |
| 10 | 109,700,000 | 109,800,000 | Voght | . | ● | 10.15 | Asians |
| 10 | 111,838,338 | 111,879,715 | Oleksyk | i | ○ |  | Europeans |
| 10 | 112,300,000 | 112,400,000 | Voght | . | ○ |  | Europeans |
| 10 | 112,400,000 | 112,500,000 | Voght | . | ○ |  | Europeans |
| 10 | 113,000,000 | 113,100,000 | Voght | . | ○ |  | Europeans |
| 10 | 117,800,000 | 117,900,000 | Voght | . | ○ |  | Europeans |
| 10 | 118,258,077 | 118,276,595 | Sabeti | . | ○ |  | Europeans |
| 10 | 121,561,497 | 121,938,286 | Oleksyk | j | ○ |  | (old) Africans & Europeans |
| 10 | 126,912,294 | 127,339,989 | Oleksyk | k | ○ |  | Europeans |
| 10 | 127,865,903 | 127,865,903 | Sabeti | . | ○ |  | Europeans |
| 10 | 134,806,178 | 134,965,405 | Oleksyk | l | ○ |  | (old) Africans & Europeans |
| 11 | 4,018,623 | 4,069,971 | Oleksyk | a | ○ |  | Europeans |
| 11 | 5,116,672 | 5,116,672 | Hapmap II | . | ○ |  | Africans |
| 11 | 6,643,083 | 6,643,083 | Akey | . | ○ |  |  |
| 11 | 10,650,000 | 10,800,000 | Hapmap II | . | ○ |  | Asians |
| 11 | 19,056,507 | 19,056,507 | Akey | . | ○ |  |  |
| 11 | 25,000,000 | 25,100,000 | Voght | . | ○ |  | Asians |
| 11 | 25,250,000 | 25,600,000 | Hapmap II | . | ● | 11.01 | Asians |
| 11 | 25,300,000 | 25,400,000 | Voght | . | ● | 11.01 | Asians |
| 11 | 28,096,168 | 28,319,252 | Wang | . | ○ |  | All populations |
| 11 | 28,400,000 | 28,500,000 | Voght | . | ○ |  | Europeans |
| 11 | 34,900,000 | 35,050,000 | Hapmap II | . | ○ |  | Europeans |
| 11 | 37,820,000 | 38,290,000 | Carlson | . | ○ |  |  |
| 11 | 37,980,000 | 38,360,000 | Carlson | . | ○ |  |  |
| 11 | 38,400,000 | 38,500,000 | Voght | . | ● | 11.03 | Africans |
| 11 | 38,400,000 | 38,500,000 | Voght | . | ● | 11.03 | Asians |
| 11 | 38,400,000 | 38,750,000 | Hapmap II | . | ● | 11.03 | Africans & Europeans |
| 11 | 38,400,000 | 38,750,000 | Hapmap II | . | ● | 11.03 | Africans & Europeans |
| 11 | 45,931,219 | 46,327,343 | Oleksyk | b | ○ |  | Europeans |
| 11 | 46,700,000 | 46,800,000 | Voght | . | ○ |  | Africans |
| 11 | 46,800,000 | 46,900,000 | Voght | . | ○ |  | Africans |
| 11 | 47,340,721 | 47,364,407 | Wang | . | ○ |  | All populations |
| 11 | 47,394,491 | 47,402,356 | Wang | . | ○ |  | All populations |
| 11 | 47,404,629 | 47,412,298 | Wang | . | ○ |  | All populations |
| 11 | 47,423,624 | 47,435,039 | Wang | . | ○ |  | All populations |
| 11 | 47,600,906 | 47,628,373 | Wang | . | ○ |  | All populations |
| 11 | 48,241,989 | 48,242,918 | Wang | . | ○ |  | All populations |
| 11 | 48,300,000 | 48,400,000 | Voght | . | ○ |  | Africans |
| 11 | 48,450,000 | 48,950,000 | Hapmap II | . | ○ |  | Asians |
| 11 | 49,132,496 | 49,194,531 | Wang | . | ○ |  | All populations |
| 11 | 49,930,551 | 49,931,480 | Wang | . | ○ |  | All populations |
| 11 | 51,268,027 | 51,268,971 | Wang | . | ○ |  | All populations |
| 11 | 55,340,000 | 55,750,000 | Carlson | . | ● | 11.04 |  |
| 11 | 55,340,000 | 55,750,000 | Carlson | . | ● | 11.05 |  |
| 11 | 55,340,000 | 55,750,000 | Carlson | . | ● | 11.06 |  |
| 11 | 55,554,471 | 55,579,355 | Wang | . | ● | 11.04 | All populations |
| 11 | 55,617,360 | 55,618,289 | Wang | . | ● | 11.05 | All populations |
| 11 | 55,700,000 | 55,800,000 | Voght | . | ● | 11.06 | Africans |
| 11 | 55,870,091 | 55,871,047 | Wang | . | ○ |  | All populations |
| 11 | 59,200,000 | 59,300,000 | Voght | . | ○ |  | Africans |
| 11 | 60,600,000 | 60,700,000 | Voght | . | ○ |  | Asians |
| 11 | 60,941,733 | 61,079,921 | Oleksyk | c | ○ |  | Europeans |
| 11 | 61,300,000 | 61,450,000 | Hapmap II | . | ○ |  | Asians |
| 11 | 63,450,000 | 63,550,000 | Hapmap II | . | ○ |  | Asians |
| 11 | 66,433,942 | 66,981,170 | Oleksyk | d | ● | 11.07 | Europeans |
| 11 | 66,900,000 | 67,000,000 | Voght | . | ● | 11.07 | Asians |
| 11 | 67,000,000 | 67,100,000 | Voght | . | ○ |  | Africans |
| 11 | 72,626,574 | 73,044,345 | Oleksyk | e | ○ |  | (old) Africans & Europeans |
| 11 | 73,343,743 | 73,343,743 | Akey | . | ○ |  |  |
| 11 | 74,300,000 | 74,400,000 | Voght | . | ○ |  | Africans |
| 11 | 75,200,000 | 75,300,000 | Voght | . | ○ |  | Africans |
| 11 | 81,000,000 | 81,100,000 | Voght | . | ○ |  | Asians |
| 11 | 81,300,000 | 81,750,000 | Hapmap II | . | ○ |  | Asians |
| 11 | 83,367,458 | 83,684,599 | Oleksyk | f | ○ |  | (old) Africans & Europeans |
| 11 | 88,598,773 | 88,716,979 | Wang | . | ○ |  | All populations |
| 11 | 89,218,976 | 89,229,896 | Wang | . | ○ |  | All populations |
| 11 | 89,820,413 | 89,820,413 | Akey | . | ○ |  |  |
| 11 | 94,742,937 | 95,156,184 | Oleksyk | g | ○ |  | Africans |
| 11 | 99,621,541 | 99,621,541 | Akey | . | ○ |  |  |
| 11 | 106,070,614 | 106,216,741 | Oleksyk | h | ○ |  | (new) Africans & Europeans |
| 11 | 108,064,763 | 108,200,879 | Oleksyk | i | ○ |  | (old) Africans & Europeans |
| 11 | 116,236,470 | 116,520,405 | Oleksyk | j | ○ |  | (new) Africans & Europeans |
| 11 | 118,418,664 | 118,418,664 | Akey | . | ○ |  |  |
| 11 | 118,429,849 | 118,429,849 | Akey | . | ○ |  |  |
| 11 | 119,550,000 | 119,700,000 | Hapmap II | . | ● | 11.08 | Europeans |
| 11 | 119,600,000 | 119,700,000 | Voght | . | ● | 11.08 | Europeans |
| 11 | 121,200,000 | 121,300,000 | Voght | . | ○ |  | Europeans |
| 11 | 122,645,551 | 122,645,551 | Akey | . | ○ |  |  |
| 11 | 126,631,282 | 126,631,282 | Akey | . | ○ |  |  |
| 11 | 126,659,012 | 126,659,012 | Akey | . | ○ |  |  |
| 11 | 128,600,000 | 128,700,000 | Voght | . | ○ |  | Europeans |
| 11 | 131,440,546 | 131,443,589 | Sabeti | . | ○ |  | Asians |
| 12 | 1,200,000 | 1,300,000 | Voght | . | ○ |  | Asians |
| 12 | 2,257,360 | 2,296,049 | Oleksyk | a | ○ |  | (old) Africans & Europeans |
| 12 | 2,500,000 | 2,600,000 | Voght | . | ○ |  | Europeans |
| 12 | 2,600,000 | 2,700,000 | Voght | . | ○ |  | Europeans |
| 12 | 2,850,000 | 2,950,000 | Hapmap II | . | ○ |  | Europeans |
| 12 | 5,612,061 | 5,612,061 | Akey | . | ○ |  |  |
| 12 | 11,049,799 | 11,049,799 | Akey | . | ○ |  |  |
| 12 | 13,478,886 | 13,478,886 | Akey | . | ○ |  |  |
| 12 | 18,400,000 | 18,500,000 | Hapmap II | . | ○ |  | Africans |
| 12 | 18,700,000 | 18,800,000 | Voght | . | ○ |  | Europeans |
| 12 | 18,900,000 | 19,000,000 | Voght | . | ○ |  | Europeans |
| 12 | 19,200,000 | 19,300,000 | Voght | . | ● | 12.01 | Europeans |
| 12 | 19,244,750 | 19,498,561 | Oleksyk | b | ● | 12.01 | Europeans |
| 12 | 20,000,000 | 20,100,000 | Voght | . | ○ |  | Europeans |
| 12 | 21,000,000 | 21,100,000 | Voght | . | ○ |  | Africans |
| 12 | 21,800,000 | 21,900,000 | Hapmap II | . | ● | 12.02 | Africans |
| 12 | 21,800,000 | 21,900,000 | Voght | . | ● | 12.02 | Africans |
| 12 | 22,370,000 | 22,700,000 | Carlson | . | ○ |  |  |
| 12 | 30,400,000 | 30,500,000 | Hapmap II | . | ○ |  | Africans |
| 12 | 34,550,000 | 36,150,000 | Hapmap II | . | ○ |  | Africans |
| 12 | 37,332,269 | 37,585,687 | Wang | . | ○ |  | All populations |
| 12 | 39,600,000 | 39,700,000 | Voght | . | ● | 12.03 | Asians |
| 12 | 39,666,569 | 39,734,697 | Oleksyk | c | ● | 12.03 | (old) Africans & Europeans |
| 12 | 39,800,000 | 39,900,000 | Voght | . | ● | 12.04 | Asians |
| 12 | 39,800,000 | 39,950,000 | Hapmap II | . | ● | 12.04 | Asians |
| 12 | 42,600,000 | 42,700,000 | Voght | . | ○ |  | Asians |
| 12 | 42,710,000 | 43,010,000 | Carlson | . | ○ |  |  |
| 12 | 43,496,926 | 44,461,736 | Oleksyk | d | ● | 12.05 | (old) Africans & Europeans |
| 12 | 43,900,000 | 44,000,000 | Voght | . | ● | 12.05 | Africans |
| 12 | 45,000,000 | 45,100,000 | Voght | . | ○ |  | Africans |
| 12 | 45,300,000 | 45,400,000 | Voght | . | ● | 12.06 | Africans |
| 12 | 45,350,000 | 45,500,000 | Hapmap II | . | ● | 12.06 | Africans |
| 12 | 49,376,005 | 49,425,162 | Wang | . | ○ |  | All populations |
| 12 | 54,583,892 | 54,725,640 | Oleksyk | e | ○ |  | (old) Africans & Europeans |
| 12 | 54,995,109 | 54,995,109 | Akey | . | ○ |  |  |
| 12 | 55,461,738 | 55,461,738 | Akey | . | ○ |  |  |
| 12 | 58,974,755 | 58,974,755 | Akey | . | ○ |  |  |
| 12 | 59,892,900 | 59,892,900 | Akey | . | ○ |  |  |
| 12 | 59,896,918 | 59,896,918 | Akey | . | ○ |  |  |
| 12 | 64,360,488 | 64,364,566 | Sabeti | . | ○ |  | Europeans |
| 12 | 65,900,000 | 66,000,000 | Voght | . | ○ |  | Europeans |
| 12 | 67,300,000 | 67,400,000 | Voght | . | ○ |  | Africans |
| 12 | 70,400,000 | 70,500,000 | Voght | . | ○ |  | Europeans |
| 12 | 73,600,000 | 73,700,000 | Voght | . | ○ |  | Africans |
| 12 | 75,300,000 | 75,400,000 | Hapmap II | . | ● | 12.07 | Europeans |
| 12 | 75,300,000 | 75,400,000 | Voght | . | ● | 12.07 | Europeans |
| 12 | 76,700,000 | 76,800,000 | Voght | . | ○ |  | Africans |
| 12 | 77,900,000 | 78,000,000 | Voght | . | ○ |  | Africans |
| 12 | 78,000,000 | 78,000,000 | Voght | . | ● | 12.08 |  |
| 12 | 78,000,000 | 78,100,000 | Voght | . | ○ |  | Africans |
| 12 | 78,000,000 | 78,650,000 | Hapmap II | . | ● | 12.08 | Africans |
| 12 | 78,000,000 | 78,650,000 | Hapmap II | . | ● | 12.09 | Africans |
| 12 | 78,000,000 | 78,650,000 | Hapmap II | . | ● | 12.10 | Africans |
| 12 | 78,000,000 | 78,650,000 | Hapmap II | . | ● | 12.11 | Africans |
| 12 | 78,000,000 | 78,650,000 | Hapmap II | . | ● | 12.12 | Africans |
| 12 | 78,000,000 | 78,650,000 | Hapmap II | . | ● | 12.13 | Africans |
| 12 | 78,000,000 | 78,650,000 | Hapmap II | . | ● | 12.14 | Africans |
| 12 | 78,000,000 | 78,650,000 | Hapmap II | . | ● | 12.15 | Africans |
| 12 | 78,000,000 | 78,650,000 | Hapmap II | . | ● | 12.16 | Africans |
| 12 | 78,000,000 | 78,650,000 | Hapmap II | . | ● | 12.17 | Africans |
| 12 | 78,000,000 | 78,650,000 | Hapmap II | . | ● | 12.18 | Africans |
| 12 | 78,100,000 | 78,100,000 | Voght | . | ● | 12.09 |  |
| 12 | 78,100,000 | 78,200,000 | Voght | . | ○ |  | Africans |
| 12 | 78,199,208 | 79,337,593 | Oleksyk | f | ● | 12.10 | Europeans |
| 12 | 78,199,208 | 79,337,593 | Oleksyk | f | ● | 12.11 | Europeans |
| 12 | 78,199,208 | 79,337,593 | Oleksyk | f | ● | 12.12 | Europeans |
| 12 | 78,199,208 | 79,337,593 | Oleksyk | f | ● | 12.13 | Europeans |
| 12 | 78,199,208 | 79,337,593 | Oleksyk | f | ● | 12.14 | Europeans |
| 12 | 78,199,208 | 79,337,593 | Oleksyk | f | ● | 12.15 | Europeans |
| 12 | 78,199,208 | 79,337,593 | Oleksyk | f | ● | 12.16 | Europeans |
| 12 | 78,199,208 | 79,337,593 | Oleksyk | f | ● | 12.17 | Europeans |
| 12 | 78,199,208 | 79,337,593 | Oleksyk | f | ● | 12.18 | Europeans |
| 12 | 78,199,208 | 79,337,593 | Oleksyk | f | ● | 12.19 | Europeans |
| 12 | 78,199,208 | 79,337,593 | Oleksyk | f | ● | 12.20 | Europeans |
| 12 | 78,200,000 | 78,200,000 | Voght | . | ● | 12.11 |  |
| 12 | 78,200,000 | 78,300,000 | Voght | . | ● | 12.13 | Africans |
| 12 | 78,400,000 | 78,500,000 | Voght | . | ○ |  | Africans |
| 12 | 78,500,000 | 78,500,000 | Voght | . | ● | 12.15 |  |
| 12 | 78,500,000 | 78,600,000 | Voght | . | ● | 12.17 | Africans |
| 12 | 78,757,457 | 78,827,321 | Sabeti | . | ● | 12.19 | Europeans |
| 12 | 79,200,000 | 79,300,000 | Voght | . | ● | 12.20 | Europeans |
| 12 | 80,100,000 | 80,200,000 | Voght | . | ○ |  | Europeans |
| 12 | 81,100,000 | 81,200,000 | Voght | . | ○ |  | Africans |
| 12 | 81,200,000 | 81,300,000 | Voght | . | ○ |  | Africans |
| 12 | 81,300,000 | 81,400,000 | Voght | . | ○ |  | Africans |
| 12 | 81,400,000 | 81,500,000 | Voght | . | ○ |  | Africans |
| 12 | 81,776,709 | 81,776,709 | Oleksyk | g | ○ |  | Europeans |
| 12 | 82,914,623 | 82,914,623 | Akey | . | ○ |  |  |
| 12 | 83,100,000 | 83,200,000 | Voght | . | ○ |  | Europeans |
| 12 | 83,679,961 | 83,679,961 | Akey | . | ○ |  |  |
| 12 | 84,874,992 | 85,735,113 | Wang | . | ○ |  | All populations |
| 12 | 86,000,000 | 86,100,000 | Voght | . | ○ |  | Africans |
| 12 | 86,840,000 | 87,360,000 | Carlson | . | ● | 12.21 |  |
| 12 | 86,894,226 | 88,246,057 | Oleksyk | h | ● | 12.21 | Europeans |
| 12 | 86,894,226 | 88,246,057 | Oleksyk | h | ● | 12.22 | Europeans |
| 12 | 86,894,226 | 88,246,057 | Oleksyk | h | ● | 12.23 | Europeans |
| 12 | 86,894,226 | 88,246,057 | Oleksyk | h | ● | 12.24 | Europeans |
| 12 | 87,400,000 | 87,500,000 | Voght | . | ● | 12.22 | Europeans |
| 12 | 87,400,000 | 87,500,000 | Voght | . | ● | 12.23 | Europeans |
| 12 | 87,490,000 | 87,840,000 | Carlson | . | ● | 12.23 |  |
| 12 | 87,490,000 | 87,840,000 | Carlson | . | ● | 12.24 |  |
| 12 | 91,600,000 | 91,700,000 | Voght | . | ○ |  | Asians |
| 12 | 91,700,000 | 91,800,000 | Voght | . | ○ |  | Europeans |
| 12 | 95,178,525 | 95,248,731 | Oleksyk | i | ○ |  | (old) Africans & Europeans |
| 12 | 109,750,000 | 109,950,000 | Hapmap II | . | ○ |  | Europeans |
| 12 | 111,100,000 | 111,200,000 | Voght | . | ○ |  | Africans |
| 12 | 117,241,967 | 117,241,967 | Akey | . | ○ |  |  |
| 12 | 117,292,613 | 117,292,613 | Akey | . | ○ |  |  |
| 12 | 125,600,000 | 125,750,000 | Hapmap II | . | ○ |  | Europeans |
| 13 | 19,040,000 | 19,390,000 | Carlson | . | ○ |  |  |
| 13 | 22,474,696 | 22,474,696 | Akey | . | ○ |  |  |
| 13 | 23,200,000 | 23,300,000 | Voght | . | ○ |  | Asians |
| 13 | 23,893,070 | 23,984,948 | Wang | . | ○ |  | All populations |
| 13 | 24,250,000 | 24,350,000 | Hapmap II | . | ○ |  | Asians |
| 13 | 24,600,000 | 24,700,000 | Voght | . | ○ |  | Africans |
| 13 | 24,957,265 | 25,000,539 | Oleksyk | a | ○ |  | Europeans |
| 13 | 40,725,570 | 41,188,783 | Oleksyk | b | ○ |  | (old) Africans & Europeans |
| 13 | 42,810,746 | 42,822,997 | Oleksyk | c | ○ |  | Africans |
| 13 | 44,404,481 | 44,503,747 | Oleksyk | d | ○ |  | Europeans |
| 13 | 46,300,000 | 46,400,000 | Voght | . | ○ |  | Africans |
| 13 | 47,566,793 | 47,987,224 | Oleksyk | e | ○ |  | Europeans |
| 13 | 50,251,300 | 50,251,300 | Akey | . | ○ |  |  |
| 13 | 50,293,792 | 50,293,792 | Akey | . | ○ |  |  |
| 13 | 50,400,000 | 50,500,000 | Voght | . | ○ |  | Africans |
| 13 | 50,700,000 | 50,800,000 | Voght | . | ○ |  | Africans |
| 13 | 55,400,000 | 55,500,000 | Voght | . | ○ |  | Africans |
| 13 | 55,600,000 | 55,700,000 | Voght | . | ○ |  | Africans |
| 13 | 55,700,000 | 55,800,000 | Voght | . | ○ |  | Africans |
| 13 | 56,500,000 | 57,100,000 | Hapmap II | . | ○ |  | Africans |
| 13 | 60,500,000 | 60,600,000 | Voght | . | ○ |  | Africans |
| 13 | 61,100,000 | 61,350,000 | Hapmap II | . | ○ |  | Asians |
| 13 | 62,440,000 | 62,760,000 | Carlson | . | ● | 13.01 |  |
| 13 | 62,700,000 | 62,850,000 | Hapmap II | . | ● | 13.01 | Asians |
| 13 | 63,300,000 | 63,400,000 | Voght | . | ○ |  | Africans |
| 13 | 66,100,000 | 66,200,000 | Voght | . | ○ |  | Africans |
| 13 | 67,150,000 | 67,350,000 | Hapmap II | . | ○ |  | Africans |
| 13 | 71,245,697 | 72,199,334 | Oleksyk | f | ○ |  | Europeans |
| 13 | 73,770,157 | 73,770,157 | Sabeti | . | ○ |  | Europeans |
| 13 | 74,000,000 | 74,100,000 | Voght | . | ○ |  | Africans |
| 13 | 74,100,000 | 74,200,000 | Voght | . | ○ |  | Africans |
| 13 | 75,100,000 | 75,250,000 | Hapmap II | . | ○ |  | Africans |
| 13 | 86,800,000 | 86,900,000 | Voght | . | ○ |  | Africans |
| 13 | 93,745,785 | 93,858,128 | Oleksyk | g | ○ |  | (old) Africans & Europeans |
| 13 | 97,442,649 | 97,443,254 | Oleksyk | h | ○ |  | (old) Africans & Europeans |
| 13 | 109,400,000 | 109,500,000 | Voght | . | ○ |  | Asians |
| 14 | 19,449,360 | 19,489,709 | Hapmap II | . | ○ |  | Africans & Europeans |
| 14 | 26,500,000 | 26,600,000 | Voght | . | ○ |  | Asians |
| 14 | 27,550,000 | 28,050,000 | Hapmap II | . | ○ |  | Asians |
| 14 | 30,200,000 | 30,300,000 | Voght | . | ○ |  | Africans |
| 14 | 33,400,000 | 33,500,000 | Voght | . | ○ |  | Europeans |
| 14 | 34,000,000 | 34,100,000 | Voght | . | ○ |  | Africans |
| 14 | 34,100,000 | 34,200,000 | Voght | . | ○ |  | Africans |
| 14 | 34,200,000 | 34,300,000 | Voght | . | ○ |  | Africans |
| 14 | 38,900,000 | 39,000,000 | Voght | . | ○ |  | Europeans |
| 14 | 44,280,000 | 44,700,000 | Carlson | . | ○ |  |  |
| 14 | 46,800,000 | 46,900,000 | Voght | . | ○ |  | Africans |
| 14 | 47,700,000 | 47,850,000 | Hapmap II | . | ○ |  | Africans |
| 14 | 48,439,791 | 48,439,791 | Akey | . | ○ |  |  |
| 14 | 50,958,956 | 50,958,956 | Akey | . | ○ |  |  |
| 14 | 55,340,617 | 55,340,617 | Akey | . | ○ |  |  |
| 14 | 56,802,747 | 56,816,883 | Oleksyk | a | ○ |  | Europeans |
| 14 | 58,052,755 | 58,327,511 | Wang | . | ○ |  | All populations |
| 14 | 58,334,879 | 58,583,052 | Oleksyk | b | ● | 14.01 | (old) Africans & Europeans |
| 14 | 58,334,879 | 58,583,052 | Oleksyk | b | ● | 14.02 | (old) Africans & Europeans |
| 14 | 58,334,879 | 58,583,052 | Oleksyk | b | ● | 14.03 | (old) Africans & Europeans |
| 14 | 58,376,472 | 58,520,318 | Wang | . | ● | 14.01 | All populations |
| 14 | 58,376,472 | 58,520,318 | Wang | . | ● | 14.02 | All populations |
| 14 | 58,376,472 | 58,520,318 | Wang | . | ● | 14.03 | All populations |
| 14 | 58,400,000 | 58,500,000 | Voght | . | ○ |  | Africans |
| 14 | 58,500,000 | 58,500,000 | Voght | . | ● | 14.02 |  |
| 14 | 58,500,000 | 58,600,000 | Voght | . | ○ |  | Africans |
| 14 | 58,600,000 | 58,700,000 | Voght | . | ○ |  | Africans |
| 14 | 58,800,000 | 58,900,000 | Voght | . | ○ |  | Africans |
| 14 | 60,100,000 | 60,200,000 | Voght | . | ● | 14.04 | Europeans |
| 14 | 60,149,309 | 60,149,309 | Akey | . | ● | 14.04 |  |
| 14 | 60,556,312 | 61,073,655 | Oleksyk | c | ○ |  | (new) Africans & Europeans |
| 14 | 61,600,910 | 61,600,910 | Akey | . | ○ |  |  |
| 14 | 63,098,495 | 63,486,567 | Oleksyk | d | ○ |  | Europeans |
| 14 | 65,000,000 | 65,500,000 | Hapmap | . | ● | 14.05 |  |
| 14 | 65,000,000 | 65,500,000 | Hapmap | . | ● | 14.06 |  |
| 14 | 65,489,505 | 66,982,767 | Oleksyk | e | ● | 14.05 | Europeans |
| 14 | 65,489,505 | 66,982,767 | Oleksyk | e | ● | 14.06 | Europeans |
| 14 | 65,489,505 | 66,982,767 | Oleksyk | e | ● | 14.07 | Europeans |
| 14 | 65,489,505 | 66,982,767 | Oleksyk | e | ● | 14.08 | Europeans |
| 14 | 65,500,000 | 65,600,000 | Voght | . | ● | 14.06 | Europeans |
| 14 | 65,500,000 | 65,600,000 | Voght | . | ● | 14.07 | Europeans |
| 14 | 65,700,000 | 65,800,000 | Voght | . | ● | 14.08 | Africans |
| 14 | 67,213,299 | 67,213,299 | Akey | . | ○ |  |  |
| 14 | 67,277,204 | 67,277,204 | Akey | . | ○ |  |  |
| 14 | 67,310,000 | 67,770,000 | Carlson | . | ● | 14.09 |  |
| 14 | 67,741,615 | 67,741,615 | Akey | . | ● | 14.09 |  |
| 14 | 69,950,000 | 70,050,000 | Hapmap II | . | ○ |  | Europeans |
| 14 | 71,827,325 | 71,828,367 | Oleksyk | f | ○ |  | (old) Africans & Europeans |
| 14 | 79,300,000 | 79,400,000 | Voght | . | ○ |  | Europeans |
| 14 | 90,976,835 | 90,976,835 | Akey | . | ○ |  |  |
| 14 | 100,300,000 | 100,400,000 | Voght | . | ○ |  | Europeans |
| 14 | 102,400,000 | 102,500,000 | Voght | . | ○ |  | Africans |
| 14 | 105,800,000 | 105,900,000 | Hapmap II | . | ○ |  | Africans |
| 15 | 22,885,366 | 22,885,366 | Akey | . | ○ |  |  |
| 15 | 23,030,430 | 23,165,462 | Oleksyk | a | ○ |  | Europeans |
| 15 | 25,800,000 | 25,900,000 | Voght | . | ○ |  | Europeans |
| 15 | 26,064,184 | 26,088,260 | Sabeti | . | ○ |  | Europeans |
| 15 | 26,900,000 | 27,000,000 | Voght | . | ○ |  | Europeans |
| 15 | 27,150,247 | 27,219,113 | Oleksyk | b | ○ |  | Europeans |
| 15 | 29,003,953 | 29,073,042 | Sabeti | . | ○ |  | Asians |
| 15 | 29,491,385 | 29,663,598 | Wang | . | ○ |  | All populations |
| 15 | 32,200,000 | 32,300,000 | Voght | . | ○ |  | Europeans |
| 15 | 33,500,000 | 33,600,000 | Voght | . | ○ |  | Asians |
| 15 | 38,600,000 | 38,700,000 | Voght | . | ○ |  | Asians |
| 15 | 40,753,789 | 40,928,968 | Wang | . | ○ |  | All populations |
| 15 | 40,953,583 | 41,114,276 | Wang | . | ○ |  | All populations |
| 15 | 41,541,729 | 41,593,146 | Wang | . | ○ |  | All populations |
| 15 | 41,855,452 | 42,606,873 | Oleksyk | c | ○ |  | Europeans |
| 15 | 43,000,000 | 43,150,000 | Hapmap II | . | ○ |  | Europeans |
| 15 | 46,155,214 | 46,657,748 | Sabeti | . | ● | 15.01 | Europeans |
| 15 | 46,300,000 | 46,400,000 | Voght | . | ● | 15.01 | Europeans |
| 15 | 47,178,569 | 47,336,525 | Wang | . | ○ |  | All populations |
| 15 | 53,250,000 | 53,700,000 | Hapmap II | . | ○ |  | Africans |
| 15 | 54,200,000 | 54,300,000 | Voght | . | ○ |  | Africans |
| 15 | 54,300,000 | 54,400,000 | Voght | . | ○ |  | Africans |
| 15 | 55,000,000 | 55,100,000 | Voght | . | ○ |  | Europeans |
| 15 | 56,831,269 | 56,831,269 | Akey | . | ○ |  |  |
| 15 | 61,550,000 | 62,070,000 | Carlson | . | ● | 15.02 |  |
| 15 | 61,550,000 | 62,070,000 | Carlson | . | ● | 15.03 |  |
| 15 | 61,550,000 | 62,070,000 | Carlson | . | ● | 15.04 |  |
| 15 | 61,550,000 | 62,070,000 | Carlson | . | ● | 15.05 |  |
| 15 | 61,550,000 | 62,070,000 | Carlson | . | ● | 15.06 |  |
| 15 | 61,600,000 | 61,700,000 | Voght | . | ○ |  | Asians |
| 15 | 61,700,000 | 61,700,000 | Voght | . | ● | 15.02 |  |
| 15 | 61,700,000 | 61,800,000 | Voght | . | ○ |  | Asians |
| 15 | 61,748,992 | 61,848,071 | Sabeti | . | ● | 15.03 | Asians |
| 15 | 61,748,992 | 61,848,071 | Sabeti | . | ● | 15.04 | Asians |
| 15 | 61,748,992 | 61,848,071 | Sabeti | . | ● | 15.05 | Asians |
| 15 | 61,800,000 | 61,800,000 | Voght | . | ● | 15.04 |  |
| 15 | 61,800,000 | 61,900,000 | Voght | . | ● | 15.06 | Asians |
| 15 | 62,100,000 | 62,200,000 | Voght | . | ● | 15.07 | Asians |
| 15 | 62,150,000 | 62,300,000 | Hapmap II | . | ● | 15.07 | Asians |
| 15 | 64,000,000 | 64,100,000 | Hapmap II | . | ○ |  | Asians |
| 15 | 65,108,106 | 65,108,106 | Akey | . | ○ |  |  |
| 15 | 67,155,853 | 67,155,853 | Oleksyk | d | ○ |  | Europeans |
| 15 | 70,094,745 | 70,837,275 | Oleksyk | e | ● | 15.08 | Europeans |
| 15 | 70,200,000 | 70,300,000 | Voght | . | ● | 15.08 | Europeans |
| 15 | 72,500,000 | 72,600,000 | Voght | . | ○ |  | Europeans |
| 15 | 72,600,000 | 72,700,000 | Voght | . | ○ |  | Europeans |
| 15 | 73,400,000 | 73,500,000 | Voght | . | ○ |  | Africans |
| 15 | 74,600,000 | 74,700,000 | Voght | . | ○ |  | Africans |
| 15 | 74,700,000 | 74,800,000 | Voght | . | ○ |  | Africans |
| 15 | 75,300,000 | 75,400,000 | Voght | . | ○ |  | Africans |
| 15 | 75,550,000 | 75,650,000 | Hapmap II | . | ○ |  | Africans |
| 15 | 80,200,000 | 80,300,000 | Voght | . | ○ |  | Asians |
| 15 | 82,780,000 | 83,180,000 | Carlson | . | ○ |  |  |
| 15 | 88,572,133 | 88,572,133 | Akey | . | ○ |  |  |
| 15 | 88,614,901 | 88,614,901 | Akey | . | ○ |  |  |
| 16 | 1,450,000 | 1,600,000 | Hapmap II | . | ○ |  | Europeans |
| 16 | 2,500,000 | 2,600,000 | Voght | . | ○ |  | Africans |
| 16 | 4,256,169 | 4,256,169 | Oleksyk | a | ○ |  | Africans |
| 16 | 4,961,272 | 4,961,272 | Akey | . | ○ |  |  |
| 16 | 8,773,580 | 12,482,931 | Huttley | . | ● | 16.01 |  |
| 16 | 10,869,626 | 10,869,626 | Akey | . | ● | 16.01 |  |
| 16 | 13,260,986 | 13,260,986 | Akey | . | ○ |  |  |
| 16 | 14,264,421 | 14,510,548 | Oleksyk | b | ● | 16.02 | Africans |
| 16 | 14,264,421 | 14,510,548 | Oleksyk | b | ● | 16.03 | Africans |
| 16 | 14,264,421 | 15,703,477 | Oleksyk | c | ○ |  | (old) Africans & Europeans |
| 16 | 14,450,000 | 14,550,000 | Hapmap II | . | ● | 16.02 | Africans |
| 16 | 14,450,000 | 14,550,000 | Hapmap II | . | ● | 16.03 | Africans |
| 16 | 14,450,000 | 14,550,000 | Hapmap II | . | ● | 16.04 | Africans |
| 16 | 14,460,000 | 14,760,000 | Carlson | . | ● | 16.03 |  |
| 16 | 14,460,000 | 14,760,000 | Carlson | . | ● | 16.04 |  |
| 16 | 17,300,000 | 17,450,000 | Hapmap II | . | ○ |  | Asians |
| 16 | 22,100,000 | 22,200,000 | Voght | . | ○ |  | Europeans |
| 16 | 22,850,000 | 22,950,000 | Hapmap II | . | ● | 16.05 | Africans |
| 16 | 22,900,000 | 23,000,000 | Voght | . | ● | 16.05 | Africans |
| 16 | 23,000,000 | 23,100,000 | Voght | . | ○ |  | Africans |
| 16 | 30,700,000 | 31,070,000 | Carlson | . | ○ |  |  |
| 16 | 31,400,000 | 31,950,000 | Hapmap II | . | ○ |  | Africans & Europeans |
| 16 | 34,050,000 | 45,100,000 | Hapmap II | . | ○ |  | All populations |
| 16 | 45,180,000 | 45,500,000 | Carlson | . | ○ |  |  |
| 16 | 46,030,000 | 46,340,000 | Carlson | . | ○ |  |  |
| 16 | 46,050,000 | 46,490,000 | Carlson | . | ○ |  |  |
| 16 | 47,000,000 | 48,250,000 | Hapmap | . | ○ |  |  |
| 16 | 54,466,001 | 54,466,001 | Akey | . | ○ |  |  |
| 16 | 59,400,000 | 59,500,000 | Voght | . | ○ |  | Africans |
| 16 | 64,165,845 | 64,452,865 | Sabeti | . | ● | 16.06 | Asians |
| 16 | 64,200,000 | 64,350,000 | Hapmap II | . | ● | 16.06 | Asians |
| 16 | 65,500,000 | 65,600,000 | Voght | . | ● | 16.07 | Asians |
| 16 | 65,590,000 | 66,060,000 | Carlson | . | ● | 16.07 |  |
| 16 | 67,750,000 | 68,250,000 | Hapmap | . | ○ |  |  |
| 16 | 72,400,000 | 72,500,000 | Voght | . | ○ |  | Asians |
| 16 | 72,500,000 | 72,600,000 | Voght | . | ○ |  | Asians |
| 16 | 74,100,000 | 74,550,000 | Hapmap II | . | ○ |  | Asians & Africa |
| 16 | 75,300,000 | 75,400,000 | Voght | . | ○ |  | Asians |
| 16 | 77,061,737 | 77,089,133 | Sabeti | . | ○ |  | Europeans |
| 16 | 77,971,979 | 77,971,979 | Akey | . | ○ |  |  |
| 16 | 78,200,000 | 78,300,000 | Voght | . | ○ |  | Europeans |
| 16 | 78,350,000 | 78,450,000 | Hapmap II | . | ○ |  | Europeans |
| 16 | 79,600,000 | 79,700,000 | Voght | . | ○ |  | Europeans |
| 16 | 79,980,694 | 79,980,694 | Akey | . | ○ |  |  |
| 17 | 3,416,640 | 3,416,640 | Akey | . | ○ |  |  |
| 17 | 3,700,000 | 3,800,000 | Voght | . | ○ |  | Africans |
| 17 | 3,800,000 | 3,900,000 | Voght | . | ○ |  | Africans |
| 17 | 4,100,000 | 4,200,000 | Voght | . | ○ |  | Asians |
| 17 | 5,200,000 | 5,300,000 | Voght | . | ● | 17.01 | Europeans |
| 17 | 5,282,598 | 5,429,076 | Oleksyk | a | ● | 17.01 | (old) Africans & Europeans |
| 17 | 18,400,000 | 18,500,000 | Hapmap II | . | ○ |  | Africans |
| 17 | 18,615,142 | 18,624,686 | Wang | . | ○ |  | All populations |
| 17 | 19,259,607 | 19,261,471 | Oleksyk | b | ○ |  | Europeans |
| 17 | 20,200,000 | 20,300,000 | Voght | . | ○ |  | Africans |
| 17 | 20,300,000 | 20,400,000 | Voght | . | ○ |  | Africans |
| 17 | 22,788,351 | 22,788,351 | Akey | . | ○ |  |  |
| 17 | 26,250,354 | 26,520,021 | Oleksyk | c | ○ |  | (new) Africans & Europeans |
| 17 | 28,400,000 | 28,500,000 | Voght | . | ○ |  | Asians |
| 17 | 28,500,000 | 28,600,000 | Voght | . | ○ |  | Europeans |
| 17 | 28,500,000 | 28,600,000 | Voght | . | ○ |  | Asians |
| 17 | 28,600,000 | 28,700,000 | Voght | . | ○ |  | Asians |
| 17 | 36,978,510 | 45,195,549 | Huttley | . | ● | 17.02 |  |
| 17 | 36,978,510 | 45,195,549 | Huttley | . | ● | 17.03 |  |
| 17 | 36,978,510 | 45,195,549 | Huttley | . | ● | 17.04 |  |
| 17 | 36,978,510 | 45,195,549 | Huttley | . | ● | 17.05 |  |
| 17 | 37,314,880 | 37,377,355 | Oleksyk | d | ● | 17.02 | (old) Africans & Europeans |
| 17 | 38,834,981 | 38,901,649 | Oleksyk | e | ● | 17.03 | Europeans |
| 17 | 44,600,000 | 44,700,000 | Voght | . | ○ |  | Europeans |
| 17 | 44,700,000 | 44,700,000 | Voght | . | ● | 17.04 |  |
| 17 | 44,700,000 | 44,800,000 | Voght | . | ● | 17.05 | Europeans |
| 17 | 46,685,443 | 46,982,190 | Wang | . | ○ |  | All populations |
| 17 | 48,900,000 | 49,000,000 | Voght | . | ○ |  | Asians |
| 17 | 49,000,000 | 49,100,000 | Voght | . | ○ |  | Asians |
| 17 | 49,303,652 | 49,303,652 | Akey | . | ○ |  |  |
| 17 | 51,920,787 | 51,920,787 | Akey | . | ○ |  |  |
| 17 | 53,305,194 | 53,357,191 | Sabeti | . | ○ |  | Asians |
| 17 | 55,057,635 | 56,734,520 | Oleksyk | f | ● | 17.06 | Europeans |
| 17 | 55,057,635 | 56,734,520 | Oleksyk | f | ● | 17.07 | Europeans |
| 17 | 55,057,635 | 56,734,520 | Oleksyk | f | ● | 17.08 | Europeans |
| 17 | 56,150,000 | 56,450,000 | Hapmap II | . | ● | 17.06 | Asians |
| 17 | 56,150,000 | 56,450,000 | Hapmap II | . | ● | 17.07 | Asians |
| 17 | 56,419,222 | 56,515,445 | Sabeti | . | ● | 17.07 | Europeans |
| 17 | 56,419,222 | 56,515,445 | Sabeti | . | ● | 17.08 | Europeans |
| 17 | 57,041,536 | 57,069,850 | Wang | . | ○ |  | All populations |
| 17 | 57,072,254 | 57,084,049 | Wang | . | ○ |  | All populations |
| 17 | 57,093,586 | 57,096,321 | Wang | . | ○ |  | All populations |
| 17 | 57,108,677 | 57,244,054 | Wang | . | ○ |  | All populations |
| 17 | 57,244,601 | 57,286,330 | Wang | . | ○ |  | All populations |
| 17 | 57,307,870 | 57,536,958 | Wang | . | ○ |  | All populations |
| 17 | 61,300,000 | 61,640,000 | Carlson | . | ○ |  |  |
| 17 | 61,700,000 | 61,800,000 | Voght | . | ● | 17.09 | Asians |
| 17 | 61,750,000 | 61,850,000 | Hapmap II | . | ● | 17.09 | Europeans |
| 17 | 63,800,000 | 63,900,000 | Voght | . | ○ |  | Europeans |
| 18 | 7,500,000 | 7,600,000 | Voght | . | ● | 18.01 | Europeans |
| 18 | 7,500,000 | 7,650,000 | Hapmap II | . | ● | 18.01 | Europeans |
| 18 | 11,900,000 | 12,000,000 | Voght | . | ○ |  | Europeans |
| 18 | 13,000,000 | 13,100,000 | Voght | . | ○ |  | Asians |
| 18 | 14,600,000 | 15,150,000 | Hapmap II | . | ○ |  | Europeans & Asians |
| 18 | 21,968,107 | 22,110,306 | Oleksyk | a | ○ |  | (old) Africans & Europeans |
| 18 | 24,400,000 | 24,500,000 | Voght | . | ○ |  | Africans |
| 18 | 24,400,000 | 24,500,000 | Voght | . | ○ |  | Europeans |
| 18 | 24,800,000 | 24,900,000 | Voght | . | ○ |  | Europeans |
| 18 | 28,630,000 | 29,170,000 | Carlson | . | ○ |  |  |
| 18 | 28,640,000 | 29,150,000 | Carlson | . | ● | 18.02 |  |
| 18 | 28,640,000 | 29,150,000 | Carlson | . | ● | 18.03 |  |
| 18 | 28,640,000 | 29,150,000 | Carlson | . | ● | 18.04 |  |
| 18 | 28,640,000 | 29,170,000 | Carlson | . | ● | 18.05 |  |
| 18 | 28,800,000 | 29,200,000 | Hapmap II | . | ● | 18.02 | Africans |
| 18 | 28,800,000 | 29,200,000 | Hapmap II | . | ● | 18.03 | Africans |
| 18 | 28,800,000 | 29,200,000 | Hapmap II | . | ● | 18.04 | Africans |
| 18 | 28,800,000 | 29,200,000 | Hapmap II | . | ● | 18.05 | Africans |
| 18 | 28,900,000 | 29,000,000 | Voght | . | ● | 18.03 | Africans |
| 18 | 29,400,000 | 29,500,000 | Voght | . | ○ |  | Asians |
| 18 | 30,300,000 | 30,400,000 | Voght | . | ○ |  | Asians |
| 18 | 32,944,869 | 33,009,630 | Oleksyk | b | ○ |  | Europeans |
| 18 | 35,300,000 | 35,400,000 | Voght | . | ○ |  | Africans |
| 18 | 38,800,000 | 39,250,000 | Hapmap II | . | ● | 18.06 | Europeans |
| 18 | 39,200,000 | 39,300,000 | Voght | . | ● | 18.06 | Africans |
| 18 | 40,900,000 | 41,000,000 | Voght | . | ○ |  | Africans |
| 18 | 51,876,927 | 52,876,962 | Oleksyk | c | ● | 18.07 | Europeans |
| 18 | 52,867,583 | 59,688,961 | Huttley | . | ● | 18.07 |  |
| 18 | 52,867,583 | 59,688,961 | Huttley | . | ● | 18.08 |  |
| 18 | 58,575,595 | 58,620,646 | Oleksyk | d | ● | 18.08 | (old) Africans & Europeans |
| 18 | 62,400,000 | 62,500,000 | Voght | . | ○ |  | Asians |
| 18 | 64,800,000 | 64,900,000 | Voght | . | ○ |  | Africans |
| 18 | 65,710,000 | 66,040,000 | Carlson | . | ○ |  |  |
| 18 | 67,903,481 | 67,903,481 | Akey | . | ○ |  |  |
| 18 | 68,900,000 | 69,050,000 | Hapmap II | . | ● | 18.09 | Europeans |
| 18 | 69,000,000 | 69,100,000 | Voght | . | ● | 18.09 | Europeans |
| 18 | 70,800,000 | 70,900,000 | Hapmap II | . | ● | 18.10 | Europeans |
| 18 | 70,800,000 | 70,900,000 | Voght | . | ● | 18.10 | Europeans |
| 18 | 75,700,000 | 75,800,000 | Voght | . | ○ |  | Africans |
| 18 | 77,426,719 | 77,426,719 | Akey | . | ○ |  |  |
| 19 | 5,772,234 | 5,772,234 | Akey | . | ○ |  |  |
| 19 | 7,021,948 | 7,021,948 | Akey | . | ○ |  |  |
| 19 | 9,300,000 | 9,400,000 | Voght | . | ○ |  | Africans |
| 19 | 11,203,703 | 11,632,747 | Oleksyk | a | ○ |  | Europeans |
| 19 | 18,818,031 | 18,818,031 | Akey | . | ○ |  |  |
| 19 | 19,300,000 | 19,400,000 | Voght | . | ○ |  | Africans |
| 19 | 21,300,000 | 21,400,000 | Voght | . | ○ |  | Africans |
| 19 | 37,518,384 | 38,803,281 | Huttley | . | ● | 19.01 |  |
| 19 | 38,200,000 | 38,300,000 | Voght | . | ● | 19.01 | Europeans |
| 19 | 42,261,222 | 42,312,502 | Wang | . | ○ |  | All populations |
| 19 | 42,517,420 | 42,547,197 | Wang | . | ○ |  | All populations |
| 19 | 42,553,899 | 42,575,806 | Wang | . | ○ |  | All populations |
| 19 | 42,553,899 | 42,575,806 | Wang | . | ○ |  | All populations |
| 19 | 42,593,899 | 42,650,179 | Wang | . | ○ |  | All populations |
| 19 | 42,651,822 | 42,668,082 | Wang | . | ○ |  | All populations |
| 19 | 42,689,538 | 42,720,601 | Wang | . | ● | 19.03 | All populations |
| 19 | 42,689,538 | 42,720,601 | Wang | . | ● | 19.03 | All populations |
| 19 | 42,700,000 | 42,800,000 | Voght | . | ● | 19.03 | Africans |
| 19 | 42,700,000 | 42,800,000 | Voght | . | ● | 19.03 | Africans |
| 19 | 42,800,000 | 42,900,000 | Voght | . | ○ |  | Africans |
| 19 | 43,400,000 | 43,600,000 | Hapmap II | . | ● | 19.04 | Africans |
| 19 | 43,500,000 | 43,600,000 | Voght | . | ● | 19.04 | Africans |
| 19 | 44,511,642 | 44,545,367 | Oleksyk | b | ○ |  | Europeans |
| 19 | 45,200,000 | 45,300,000 | Hapmap II | . | ○ |  | Europeans |
| 19 | 47,000,000 | 47,100,000 | Voght | . | ○ |  | Africans |
| 19 | 47,540,000 | 47,920,000 | Carlson | . | ● | 19.05 |  |
| 19 | 47,750,000 | 48,250,000 | Hapmap | . | ● | 19.05 |  |
| 19 | 55,589,757 | 55,589,757 | Akey | . | ○ |  |  |
| 19 | 58,485,228 | 58,485,228 | Akey | . | ○ |  |  |
| 19 | 64,521,699 | 64,521,699 | Akey | . | ○ |  |  |
| 19 | 66,755,141 | 66,755,141 | Akey | . | ○ |  |  |
| 19 | 74,395,564 | 74,395,564 | Akey | . | ○ |  |  |
| 20 | 1,500,000 | 1,600,000 | Voght | . | ○ |  | Africans |
| 20 | 2,000,000 | 2,100,000 | Voght | . | ○ |  | Africans |
| 20 | 6,850,000 | 7,000,000 | Hapmap II | . | ○ |  | Europeans |
| 20 | 7,847,591 | 7,851,182 | Oleksyk | a | ○ |  | Africans |
| 20 | 20,360,000 | 20,690,000 | Carlson | . | ○ |  |  |
| 20 | 20,360,000 | 20,720,000 | Carlson | . | ○ |  |  |
| 20 | 21,500,000 | 21,600,000 | Voght | . | ○ |  | Europeans |
| 20 | 21,800,000 | 21,900,000 | Voght | . | ○ |  | Europeans |
| 20 | 22,217,440 | 22,524,281 | Oleksyk | b | ○ |  | Europeans |
| 20 | 30,950,006 | 30,950,006 | Akey | . | ○ |  |  |
| 20 | 31,233,084 | 31,233,084 | Akey | . | ○ |  |  |
| 20 | 32,755,809 | 32,764,898 | Wang | . | ○ |  | All populations |
| 20 | 33,700,000 | 33,900,000 | Hapmap II | . | ● | 20.01 | Africans |
| 20 | 33,864,022 | 33,980,565 | Wang | . | ● | 20.01 | All populations |
| 20 | 34,018,254 | 34,129,109 | Wang | . | ○ |  | All populations |
| 20 | 34,600,000 | 34,700,000 | Voght | . | ○ |  | Asians |
| 20 | 34,700,000 | 34,800,000 | Voght | . | ○ |  | Asians |
| 20 | 34,800,000 | 34,900,000 | Voght | . | ○ |  | Europeans |
| 20 | 34,800,000 | 34,900,000 | Voght | . | ○ |  | Asians |
| 20 | 34,900,000 | 35,000,000 | Voght | . | ○ |  | Europeans |
| 20 | 34,900,000 | 35,000,000 | Voght | . | ○ |  | Asians |
| 20 | 35,000,000 | 35,100,000 | Voght | . | ○ |  | Africans |
| 20 | 35,100,000 | 35,200,000 | Voght | . | ○ |  | Asians |
| 20 | 35,200,000 | 35,300,000 | Voght | . | ○ |  | Asians |
| 20 | 35,501,910 | 35,501,910 | Akey | . | ○ |  |  |
| 20 | 35,850,000 | 35,950,000 | Hapmap II | . | ○ |  | Africans |
| 20 | 36,183,921 | 36,183,921 | Akey | . | ○ |  |  |
| 20 | 36,750,000 | 36,950,000 | Hapmap II | . | ○ |  | Africans |
| 20 | 38,000,000 | 38,100,000 | Voght | . | ○ |  | Africans |
| 20 | 38,100,000 | 38,200,000 | Voght | . | ○ |  | Africans |
| 20 | 38,346,883 | 38,351,567 | Oleksyk | c | ○ |  | (old) Africans & Europeans |
| 20 | 38,400,000 | 38,500,000 | Voght | . | ○ |  | Africans |
| 20 | 42,145,276 | 42,145,276 | Akey | . | ○ |  |  |
| 20 | 48,000,000 | 48,100,000 | Voght | . | ○ |  | Africans |
| 20 | 48,348,260 | 48,398,908 | Wang | . | ○ |  | All populations |
| 20 | 54,300,000 | 54,400,000 | Voght | . | ○ |  | Europeans |
| 20 | 55,563,784 | 55,563,784 | Akey | . | ○ |  |  |
| 20 | 60,536,991 | 60,536,991 | Akey | . | ○ |  |  |
| 20 | 61,970,107 | 61,977,412 | Oleksyk | d | ○ |  | (old) Africans & Europeans |
| 21 | 15,740,038 | 15,755,877 | Oleksyk | a | ○ |  | (old) Africans & Europeans |
| 21 | 16,497,420 | 16,497,420 | Oleksyk | b | ○ |  | Europeans |
| 21 | 20,965,325 | 21,079,398 | Oleksyk | c | ○ |  | (old) Africans & Europeans |
| 21 | 24,600,000 | 24,700,000 | Voght | . | ○ |  | Africans |
| 21 | 26,693,313 | 26,704,839 | Oleksyk | d | ○ |  | (old) Africans & Europeans |
| 21 | 27,583,108 | 27,818,330 | Oleksyk | e | ○ |  | (old) Africans & Europeans |
| 21 | 29,500,000 | 29,600,000 | Voght | . | ○ |  | Africans |
| 21 | 29,600,000 | 29,700,000 | Voght | . | ○ |  | Africans |
| 21 | 29,771,521 | 30,094,429 | Oleksyk | f | ○ |  | Europeans |
| 21 | 31,519,682 | 31,658,040 | Oleksyk | g | ○ |  | (old) Africans & Europeans |
| 21 | 32,900,000 | 33,000,000 | Voght | . | ○ |  | Europeans |
| 21 | 33,634,289 | 34,048,167 | Oleksyk | h | ○ |  | Africans |
| 21 | 37,124,863 | 37,124,863 | Akey | . | ○ |  |  |
| 21 | 37,709,549 | 37,740,237 | Oleksyk | i | ○ |  | (old) Africans & Europeans |
| 21 | 38,771,423 | 38,843,840 | Oleksyk | j | ○ |  | (old) Africans & Europeans |
| 21 | 43,100,000 | 43,200,000 | Voght | . | ○ |  | Africans |
| 21 | 43,206,725 | 43,206,725 | Oleksyk | k | ○ |  | (old) Africans & Europeans |
| 21 | 45,022,369 | 45,023,242 | Oleksyk | l | ○ |  | (old) Africans & Europeans |
| 21 | 45,400,000 | 45,500,000 | Voght | . | ○ |  | Africans |
| 21 | 46,000,000 | 46,100,000 | Voght | . | ○ |  | Europeans |
| 21 | 46,100,000 | 46,200,000 | Voght | . | ○ |  | Africans |
| 21 | 46,600,000 | 46,700,000 | Voght | . | ○ |  | Africans |
| 22 | 15,998,626 | 16,020,377 | Oleksyk | a | ○ |  | (old) Africans & Europeans |
| 22 | 17,571,282 | 17,665,523 | Oleksyk | b | ○ |  | (old) Africans & Europeans |
| 22 | 17,700,000 | 17,800,000 | Voght | . | ○ |  | Africans |
| 22 | 19,186,712 | 19,186,712 | Akey | . | ○ |  |  |
| 22 | 23,000,000 | 23,100,000 | Voght | . | ○ |  | Asians |
| 22 | 26,700,000 | 27,190,000 | Carlson | . | ● | 22.01 |  |
| 22 | 26,700,000 | 27,190,000 | Carlson | . | ● | 22.02 |  |
| 22 | 26,700,000 | 27,190,000 | Carlson | . | ● | 22.03 |  |
| 22 | 26,712,933 | 27,090,089 | Oleksyk | c | ● | 22.01 | (old) Africans & Europeans |
| 22 | 26,712,933 | 27,090,089 | Oleksyk | c | ● | 22.02 | (old) Africans & Europeans |
| 22 | 26,712,933 | 27,090,089 | Oleksyk | c | ● | 22.03 | (old) Africans & Europeans |
| 22 | 26,800,000 | 26,900,000 | Voght | . | ● | 22.02 | Africans |
| 22 | 29,000,000 | 29,100,000 | Voght | . | ○ |  | Europeans |
| 22 | 29,250,000 | 29,500,000 | Hapmap II | . | ○ |  | Africans |
| 22 | 29,600,000 | 29,700,000 | Voght | . | ○ |  | Africans |
| 22 | 30,100,000 | 30,200,000 | Voght | . | ○ |  | Africans |
| 22 | 30,216,815 | 30,339,089 | Wang | . | ○ |  | All populations |
| 22 | 32,350,000 | 32,650,000 | Hapmap II | . | ○ |  | Africans |
| 22 | 33,300,000 | 33,400,000 | Voght | . | ○ |  | Europeans |
| 22 | 33,400,000 | 33,500,000 | Voght | . | ○ |  | Europeans |
| 22 | 33,869,256 | 33,913,543 | Oleksyk | d | ○ |  | Europeans |
| 22 | 34,023,587 | 34,961,386 | Huttley | . | ● | 22.04 |  |
| 22 | 34,023,587 | 34,961,386 | Huttley | . | ● | 22.05 |  |
| 22 | 34,800,000 | 34,900,000 | Voght | . | ○ |  | Asians |
| 22 | 34,800,000 | 35,100,000 | Hapmap II | . | ● | 22.04 | Africans |
| 22 | 34,800,000 | 35,100,000 | Hapmap II | . | ● | 22.05 | Africans |
| 22 | 34,800,000 | 35,100,000 | Hapmap II | . | ● | 22.06 | Africans |
| 22 | 34,900,000 | 34,900,000 | Voght | . | ● | 22.04 |  |
| 22 | 34,900,000 | 35,000,000 | Voght | . | ● | 22.06 | Africans |
| 22 | 37,900,000 | 38,000,000 | Voght | . | ● | 22.07 | Asians |
| 22 | 37,941,616 | 37,941,616 | Akey | . | ● | 22.07 |  |
| 22 | 39,700,000 | 39,800,000 | Voght | . | ○ |  | Africans |
| 22 | 40,166,703 | 40,567,520 | Oleksyk | e | ○ |  | (new) Africans & Europeans |
| 22 | 41,500,000 | 41,600,000 | Voght | . | ○ |  | Africans |
| 22 | 44,032,391 | 44,143,223 | Oleksyk | f | ○ |  | Africans |
| 22 | 45,109,651 | 45,133,715 | Sabeti | . | ○ |  | Asians |
| 22 | 45,650,000 | 45,800,000 | Hapmap II | . | ○ |  | Asians |
| 22 | 48,309,676 | 48,331,431 | Oleksyk | g | ○ |  | (old) Africans & Europeans |
| 22 | 48,400,000 | 48,500,000 | Voght | . | ○ |  | Europeans |
| X | 6,599,002 | 6,734,356 | Wang | . | ○ |  | All populations |
| X | 6,826,697 | 6,925,883 | Wang | . | ○ |  | All populations |
| X | 7,637,285 | 7,637,285 | Akey | . | ○ |  |  |
| X | 7,686,527 | 7,686,527 | Akey | . | ○ |  |  |
| X | 7,694,438 | 7,694,438 | Akey | . | ○ |  |  |
| X | 7,703,758 | 7,703,758 | Akey | . | ○ |  |  |
| X | 7,711,667 | 7,711,667 | Akey | . | ○ |  |  |
| X | 13,900,000 | 14,000,000 | Voght | . | ● | 23.01 | Asians |
| X | 13,909,076 | 14,111,360 | Wang | . | ● | 23.01 | All populations |
| X | 18,850,000 | 19,050,000 | Hapmap II | . | ● | 23.02 | Europeans |
| X | 18,881,880 | 19,138,487 | Sabeti | . | ● | 23.02 | Europeans |
| X | 19,200,000 | 21,300,000 | Hapmap | . | ● | 23.03 |  |
| X | 20,000,000 | 20,100,000 | Voght | . | ● | 23.03 | Africans |
| X | 25,571,983 | 25,575,189 | Wang | . | ○ |  | All populations |
| X | 26,600,000 | 26,700,000 | Hapmap II | . | ○ |  | Asians |
| X | 29,700,000 | 29,800,000 | Voght | . | ○ |  | Europeans |
| X | 30,150,000 | 30,300,000 | Hapmap II | . | ○ |  | Asians |
| X | 32,300,000 | 32,400,000 | Hapmap II | . | ○ |  | Africans |
| X | 33,907,530 | 33,910,088 | Wang | . | ○ |  | All populations |
| X | 34,100,000 | 34,200,000 | Voght | . | ○ |  | Europeans |
| X | 34,322,357 | 34,324,460 | Wang | . | ○ |  | All populations |
| X | 34,600,000 | 34,700,000 | Voght | . | ○ |  | Africans |
| X | 34,600,000 | 34,700,000 | Voght | . | ○ |  | Europeans |
| X | 34,700,000 | 34,800,000 | Voght | . | ○ |  | Africans |
| X | 34,700,000 | 34,800,000 | Voght | . | ○ |  | Europeans |
| X | 34,800,000 | 34,900,000 | Voght | . | ● | 23.04 | Africans |
| X | 34,900,000 | 35,350,000 | Hapmap II | . | ● | 23.04 | Africans |
| X | 35,400,000 | 37,500,000 | Hapmap | . | ● | 23.05 |  |
| X | 35,400,000 | 37,500,000 | Hapmap | . | ● | 23.06 |  |
| X | 35,400,000 | 37,500,000 | Hapmap | . | ● | 23.07 |  |
| X | 35,400,000 | 37,500,000 | Hapmap | . | ● | 23.08 |  |
| X | 35,400,000 | 37,500,000 | Hapmap | . | ● | 23.09 |  |
| X | 35,400,000 | 37,500,000 | Hapmap | . | ● | 23.10 |  |
| X | 35,400,000 | 37,500,000 | Hapmap | . | ● | 23.11 |  |
| X | 35,400,000 | 37,500,000 | Hapmap | . | ● | 23.12 |  |
| X | 35,400,000 | 37,500,000 | Hapmap | . | ● | 23.13 |  |
| X | 35,400,000 | 37,500,000 | Hapmap | . | ● | 23.14 |  |
| X | 35,400,000 | 37,500,000 | Hapmap | . | ● | 23.15 |  |
| X | 35,600,000 | 35,700,000 | Voght | . | ○ |  | Europeans |
| X | 35,700,000 | 35,700,000 | Voght | . | ● | 23.05 |  |
| X | 35,700,000 | 35,800,000 | Voght | . | ○ |  | Europeans |
| X | 35,759,035 | 35,939,638 | Sabeti | . | ● | 23.06 | Europeans |
| X | 35,759,035 | 35,939,638 | Sabeti | . | ● | 23.07 | Europeans |
| X | 35,759,035 | 35,939,638 | Sabeti | . | ● | 23.08 | Europeans |
| X | 35,759,035 | 35,939,638 | Sabeti | . | ● | 23.09 | Europeans |
| X | 35,759,035 | 35,939,638 | Sabeti | . | ● | 23.10 | Europeans |
| X | 35,800,000 | 35,800,000 | Voght | . | ● | 23.07 |  |
| X | 35,800,000 | 35,900,000 | Voght | . | ● | 23.09 | Europeans |
| X | 36,239,501 | 36,347,420 | Wang | . | ● | 23.11 | All populations |
| X | 36,461,698 | 36,567,625 | Wang | . | ● | 23.12 | All populations |
| X | 36,461,698 | 36,567,625 | Wang | . | ● | 23.13 | All populations |
| X | 36,461,698 | 36,567,625 | Wang | . | ● | 23.14 | All populations |
| X | 36,476,826 | 36,521,901 | Sabeti | . | ● | 23.13 | Asians |
| X | 37,069,665 | 37,555,024 | Sabeti | . | ● | 23.15 | Europeans & Asians |
| X | 40,400,000 | 40,500,000 | Voght | . | ○ |  | Africans |
| X | 40,500,000 | 40,600,000 | Voght | . | ○ |  | Africans |
| X | 40,600,000 | 40,700,000 | Voght | . | ○ |  | Africans |
| X | 41,150,000 | 41,300,000 | Hapmap II | . | ○ |  | Africans |
| X | 50,553,552 | 50,562,256 | Wang | . | ○ |  | All populations |
| X | 50,710,762 | 50,714,157 | Wang | . | ○ |  | All populations |
| X | 53,176,837 | 53,176,837 | Akey | . | ○ |  |  |
| X | 57,700,000 | 61,850,000 | Hapmap II | . | ● | 23.16 | Europeans & Asians |
| X | 57,700,000 | 61,850,000 | Hapmap II | . | ● | 23.17 | Europeans & Asians |
| X | 59,910,280 | 59,910,280 | Akey | . | ● | 23.16 |  |
| X | 59,978,912 | 59,978,912 | Akey | . | ● | 23.17 |  |
| X | 61,800,000 | 61,850,000 | Hapmap II | . | ● | 23.18 |  |
| X | 61,800,000 | 64,500,000 | Hapmap | . | ● | 23.18 |  |
| X | 61,800,000 | 64,500,000 | Hapmap | . | ● | 23.19 |  |
| X | 61,800,000 | 64,500,000 | Hapmap | . | ● | 23.20 |  |
| X | 61,800,000 | 64,500,000 | Hapmap | . | ● | 23.21 |  |
| X | 61,800,000 | 64,500,000 | Hapmap | . | ● | 23.22 |  |
| X | 61,800,000 | 64,500,000 | Hapmap | . | ● | 23.23 |  |
| X | 61,800,000 | 64,500,000 | Hapmap | . | ● | 23.24 |  |
| X | 61,800,000 | 64,500,000 | Hapmap | . | ● | 23.25 |  |
| X | 61,800,000 | 65,200,000 | Hapmap II | . | ● | 23.26 | Europeans & Asians |
| X | 62,804,285 | 62,804,285 | Akey | . | ● | 23.19 |  |
| X | 62,818,088 | 62,818,088 | Akey | . | ● | 23.20 |  |
| X | 62,850,103 | 62,850,103 | Akey | . | ● | 23.21 |  |
| X | 62,924,341 | 62,924,341 | Akey | . | ● | 23.22 |  |
| X | 63,800,000 | 63,900,000 | Voght | . | ○ |  | Africans |
| X | 63,900,000 | 63,900,000 | Voght | . | ● | 23.23 |  |
| X | 63,900,000 | 64,000,000 | Voght | . | ○ |  | Africans |
| X | 64,000,000 | 64,000,000 | Voght | . | ● | 23.24 |  |
| X | 64,000,000 | 64,100,000 | Voght | . | ● | 23.25 | Africans |
| X | 65,159,749 | 67,836,528 | Oleksyk | a | ● | 23.26 | Africans |
| X | 65,159,749 | 67,836,528 | Oleksyk | a | ● | 23.27 | Africans |
| X | 65,159,749 | 67,836,528 | Oleksyk | a | ● | 23.28 | Africans |
| X | 65,159,749 | 67,836,528 | Oleksyk | a | ● | 23.29 | Africans |
| X | 65,159,749 | 67,836,528 | Oleksyk | a | ● | 23.30 | Africans |
| X | 66,000,000 | 66,100,000 | Voght | . | ● | 23.27 | Europeans |
| X | 66,129,506 | 66,520,438 | Wang | . | ● | 23.28 | All populations |
| X | 66,129,506 | 66,520,438 | Wang | . | ● | 23.29 | All populations |
| X | 66,129,506 | 66,520,438 | Wang | . | ● | 23.30 | All populations |
| X | 66,200,000 | 66,500,000 | Hapmap II | . | ● | 23.29 | Asians |
| X | 68,154,620 | 68,154,620 | Akey | . | ○ |  |  |
| X | 71,800,000 | 71,900,000 | Voght | . | ○ |  | Africans |
| X | 71,900,000 | 72,000,000 | Voght | . | ○ |  | Africans |
| X | 72,450,000 | 72,550,000 | Hapmap II | . | ○ |  | Asians |
| X | 76,766,175 | 76,768,467 | Wang | . | ○ |  | All populations |
| X | 79,000,000 | 79,100,000 | Voght | . | ○ |  | Africans |
| X | 79,213,496 | 79,309,920 | Wang | . | ○ |  | All populations |
| X | 79,482,110 | 79,506,955 | Wang | . | ○ |  | All populations |
| X | 79,737,810 | 79,871,378 | Wang | . | ○ |  | All populations |
| X | 81,600,000 | 82,200,000 | Hapmap | . | ○ |  |  |
| X | 82,500,000 | 82,600,000 | Voght | . | ○ |  | Europeans |
| X | 83,900,000 | 84,000,000 | Voght | . | ○ |  | Europeans |
| X | 87,250,000 | 87,400,000 | Hapmap II | . | ○ |  | Asians |
| X | 88,050,000 | 88,300,000 | Hapmap II | . | ○ |  | Asians |
| X | 97,400,000 | 97,500,000 | Voght | . | ○ |  | Africans |
| X | 97,800,000 | 97,900,000 | Voght | . | ○ |  | Europeans |
| X | 97,900,000 | 98,000,000 | Voght | . | ○ |  | Europeans |
| X | 98,400,000 | 99,000,000 | Hapmap II | . | ○ |  | Africans & Europeans |
| X | 100,500,000 | 100,600,000 | Voght | . | ○ |  | Africans |
| X | 103,212,014 | 103,783,679 | Wang | . | ○ |  | All populations |
| X | 103,800,000 | 103,900,000 | Voght | . | ○ |  | Asians |
| X | 104,296,315 | 104,680,742 | Oleksyk | b | ● | 23.31 | Europeans |
| X | 104,400,000 | 106,500,000 | Hapmap | . | ● | 23.31 |  |
| X | 104,400,000 | 106,500,000 | Hapmap | . | ● | 23.32 |  |
| X | 104,400,000 | 106,500,000 | Hapmap | . | ● | 23.33 |  |
| X | 104,400,000 | 106,500,000 | Hapmap | . | ● | 23.34 |  |
| X | 104,955,826 | 105,001,082 | Wang | . | ● | 23.32 | All populations |
| X | 106,114,888 | 106,114,888 | Akey | . | ● | 23.33 |  |
| X | 106,170,703 | 106,454,568 | Wang | . | ● | 23.34 | All populations |
| X | 108,900,000 | 110,700,000 | Hapmap | . | ● | 23.35 |  |
| X | 109,767,056 | 111,117,626 | Sabeti | . | ● | 23.35 | Asians |
| X | 113,291,719 | 113,296,616 | Sabeti | . | ○ |  | Asians |
| X | 121,100,000 | 121,200,000 | Hapmap II | . | ○ |  | Asians |
| X | 123,900,000 | 124,000,000 | Voght | . | ○ |  | Africans |
| X | 125,400,000 | 125,500,000 | Voght | . | ○ |  | Europeans |
| X | 125,600,000 | 125,700,000 | Voght | . | ○ |  | Europeans |
| X | 125,700,000 | 125,800,000 | Voght | . | ○ |  | Europeans |
| X | 125,800,000 | 125,900,000 | Voght | . | ○ |  | Europeans |
| X | 125,900,000 | 126,000,000 | Voght | . | ○ |  | Europeans |
| X | 126,000,000 | 126,100,000 | Voght | . | ○ |  | Europeans |
| X | 131,200,000 | 131,300,000 | Voght | . | ○ |  | Africans |
| X | 133,400,000 | 133,500,000 | Voght | . | ○ |  | Africans |
| X | 134,550,000 | 134,700,000 | Hapmap II | . | ○ |  | Africans |
| X | 136,100,000 | 136,200,000 | Voght | . | ○ |  | Asians |
| X | 141,796,760 | 141,804,088 | Sabeti | . | ○ |  | Europeans |
| X | 146,800,000 | 147,000,000 | Hapmap II | . | ○ |  | Europeans |
| X | 147,341,578 | 147,421,230 | Sabeti | . | ○ |  | Asians |
| X | 150,287,808 | 150,488,109 | Sabeti | . | ○ |  | Asians |
| X | 152,532,911 | 152,673,280 | Wang | . | ○ |  | All populations |
| X | 152,722,115 | 152,773,663 | Wang | . | ○ |  | All populations |
| X | 152,900,000 | 153,000,000 | Voght | . | ○ |  | Asians |
| X | 153,818,826 | 153,847,088 | Wang | . | ○ |  | All populations |
| X | 154,200,000 | 154,500,000 | Hapmap II | . | ○ |  | Asians |

^‡^Note all locations were converted into base pair (bp) positions from Build 35 of the human genome.

^†^ The studies referenced are Huttley et al. [20], Akey et al. [21], Carlson et al. [22], Nielsen et al. [23], the first generation Hapmap by the International HapMap Consortium[24], Voight et al. [25], and Wang et al. [26], the second generation Hapmap [27], and Sabeti et al.[28].

^♦^ Peak names refer to the peak name nomenclature in this study, same as in Figures 5 and 6

^^ Region name is the same as in Table S4.

^^ A study can be cross validated in more than one region. Therefore, some peaks are listed several times.
