# Supplementary figures and images for: Identifying Selected Regions from Heterozygosity and Divergence Using a Light-Coverage Genomic Dataset from Two Human Populations
Source: PLoS One. 2008 Mar 5;3(3):e1712. doi: 10.1371/journal.pone.0001712 (PMC2248624; doi:10.1371/journal.pone.0001712)

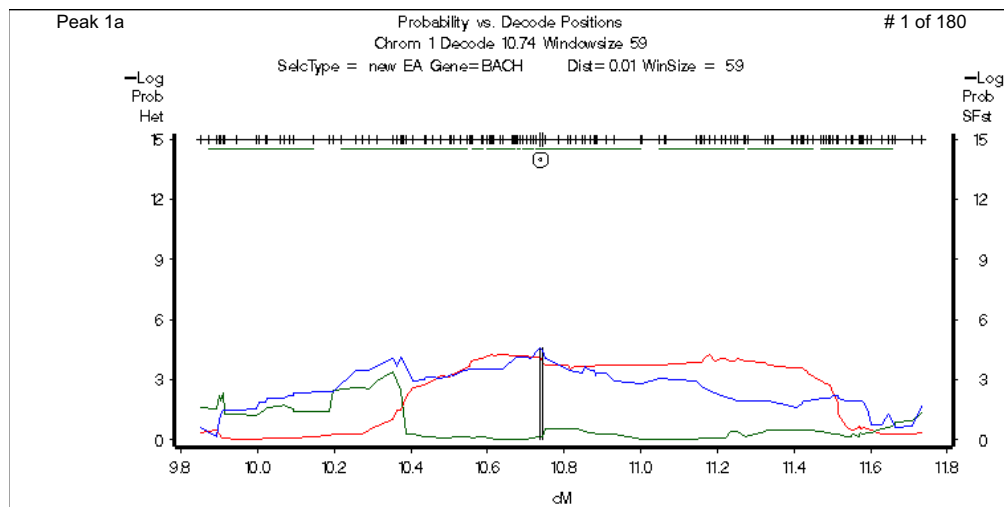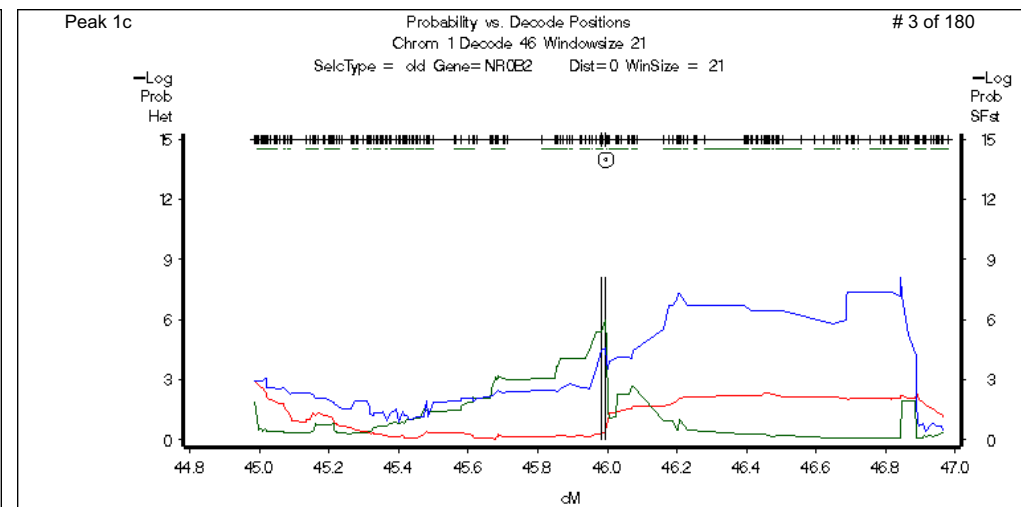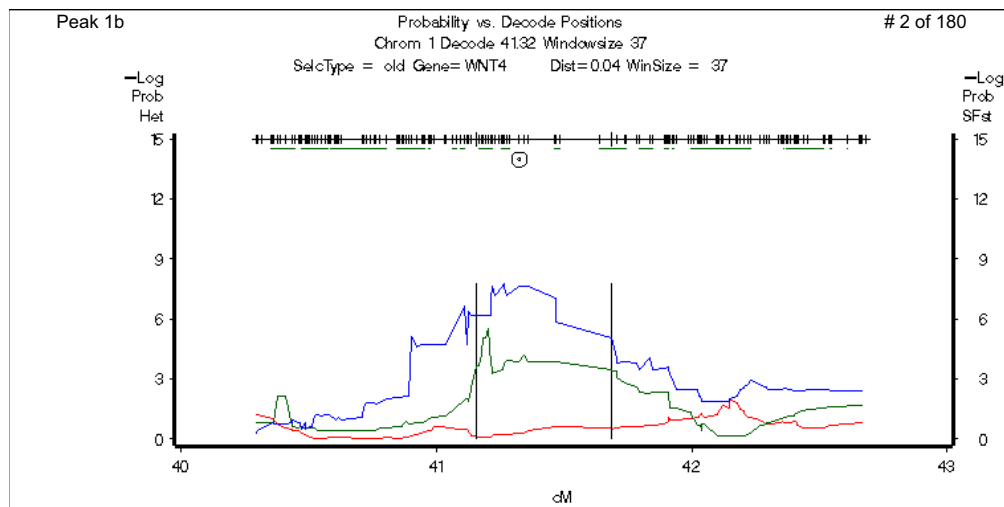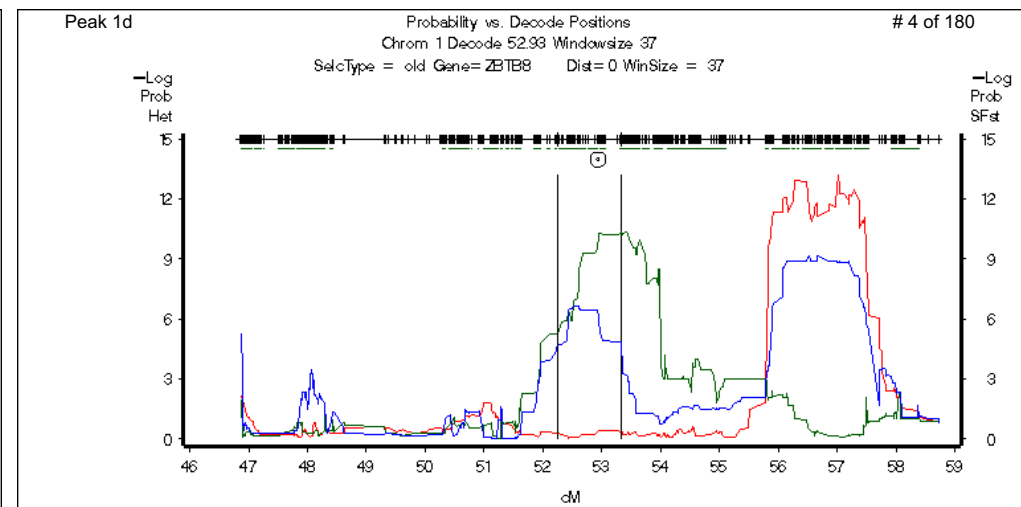

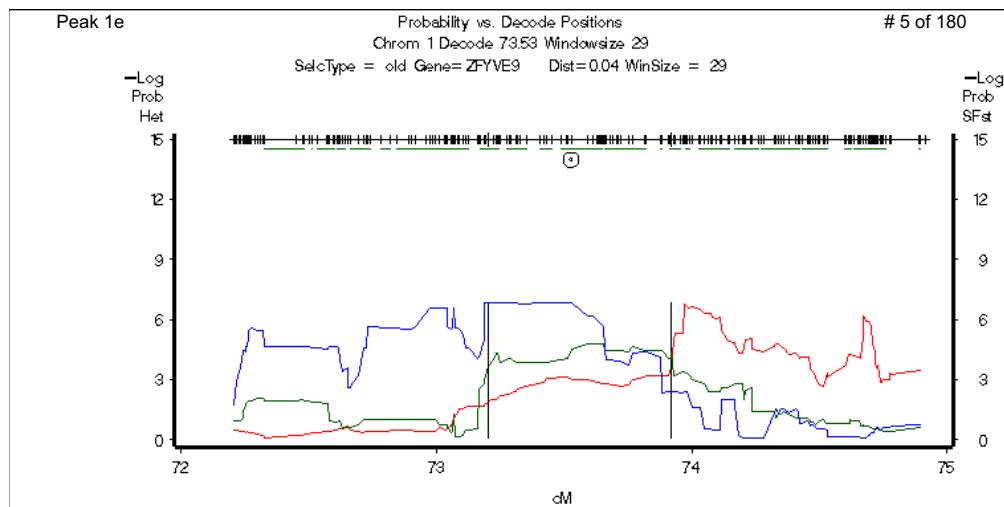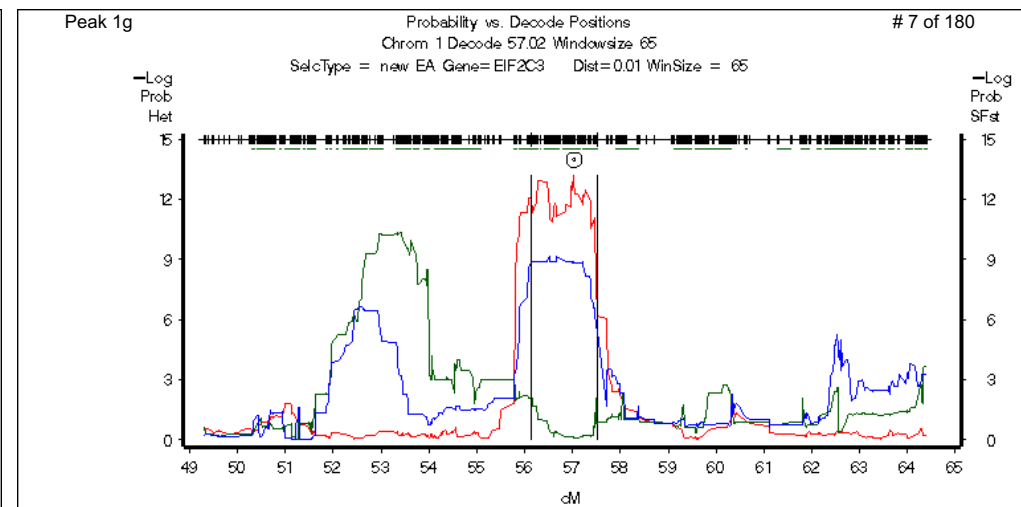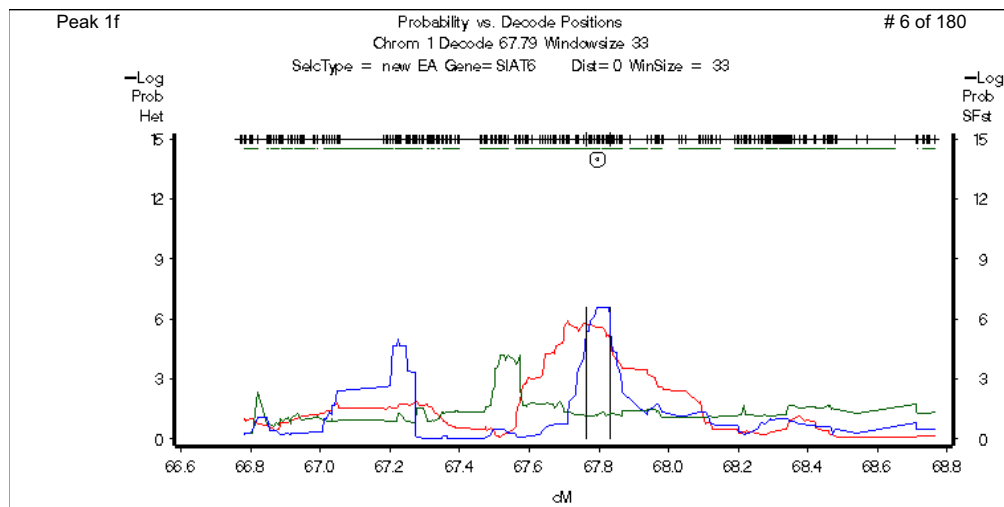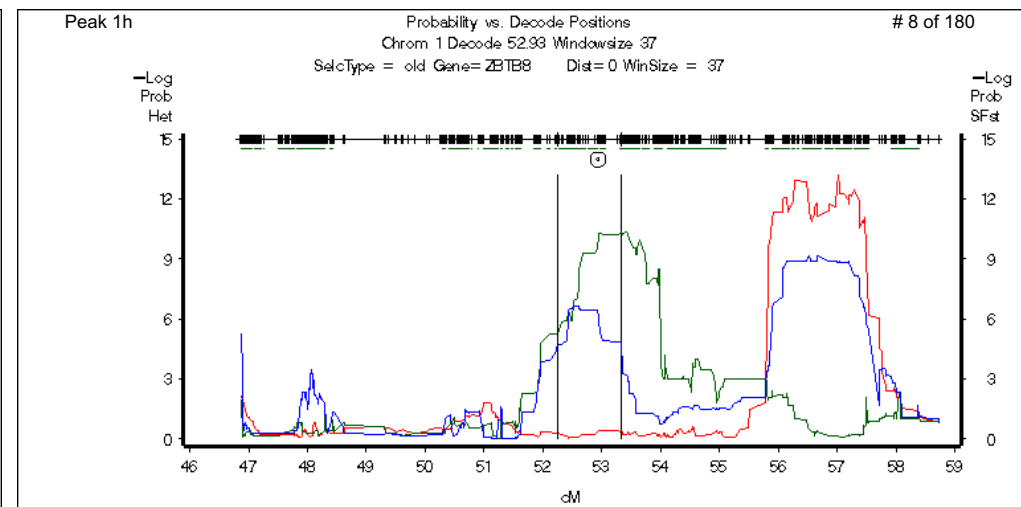

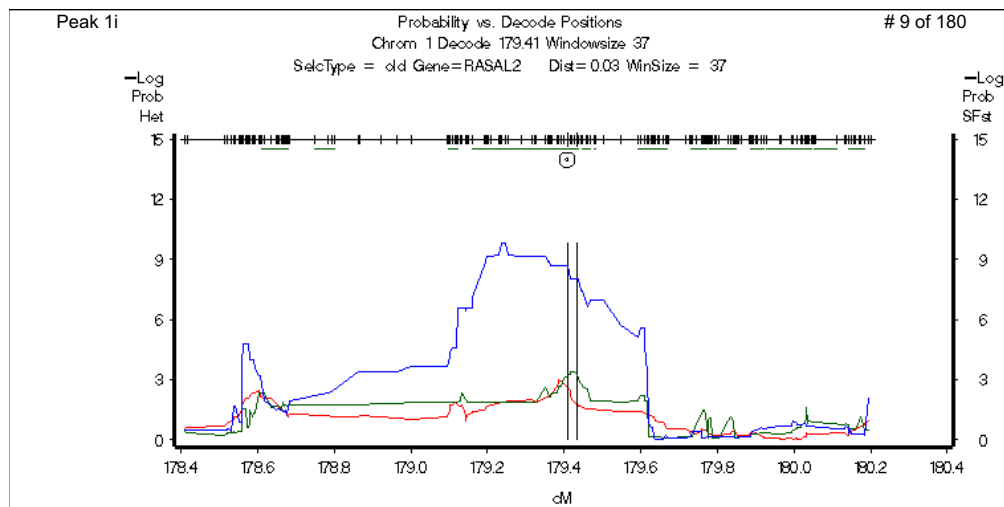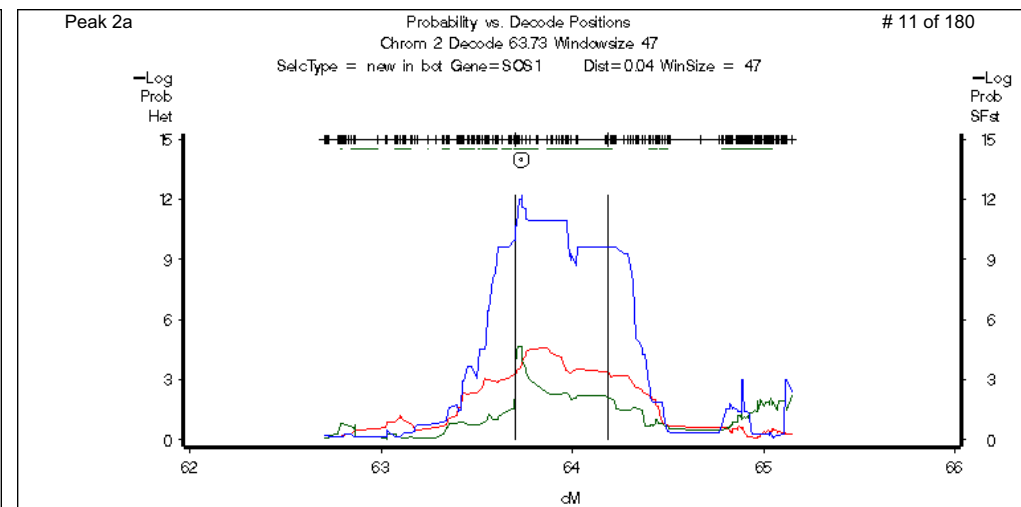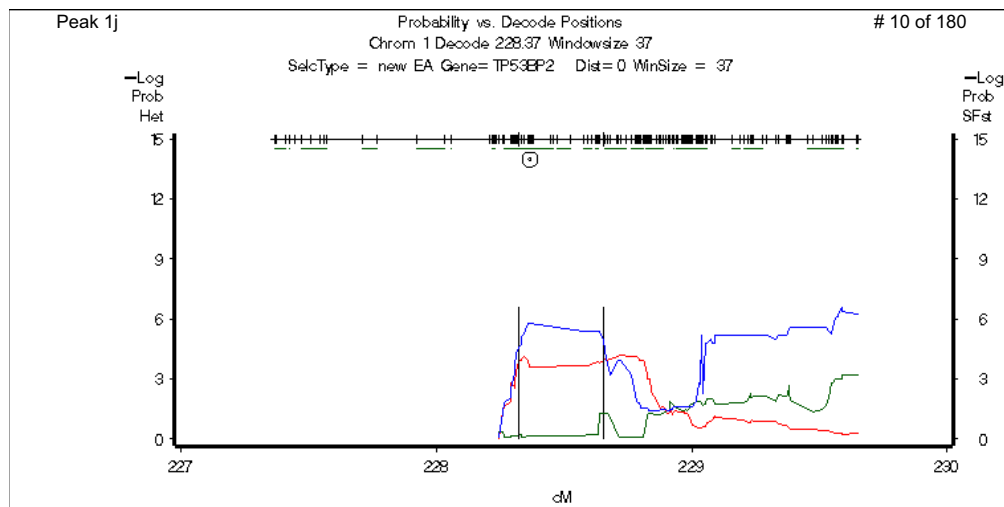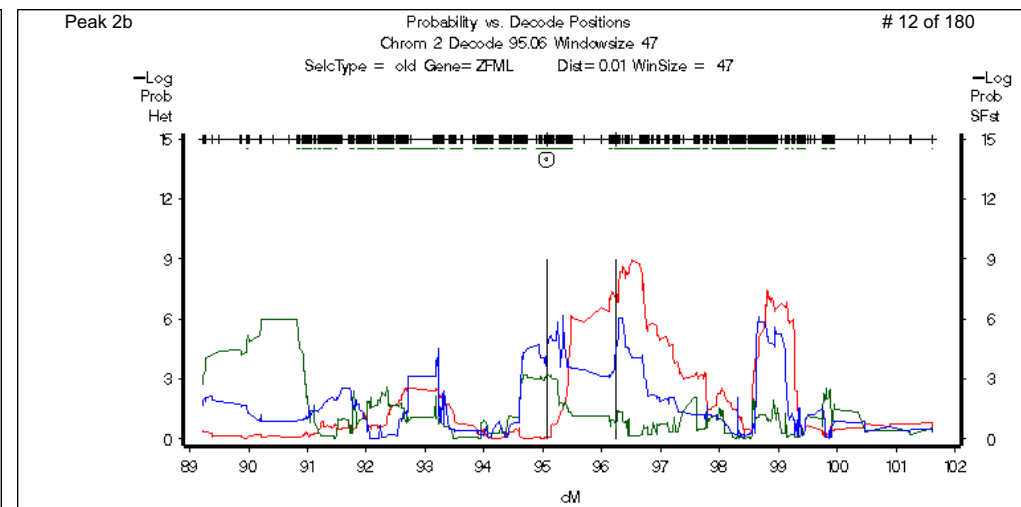

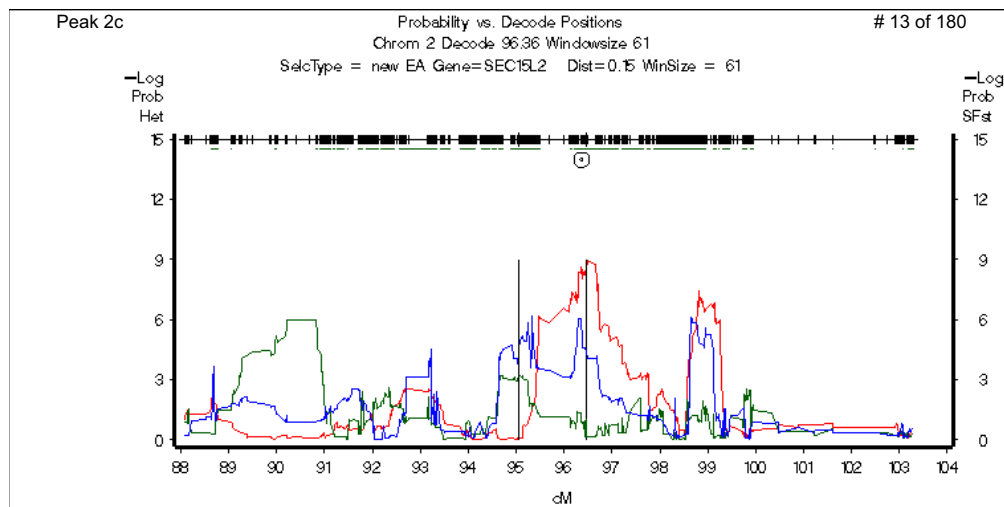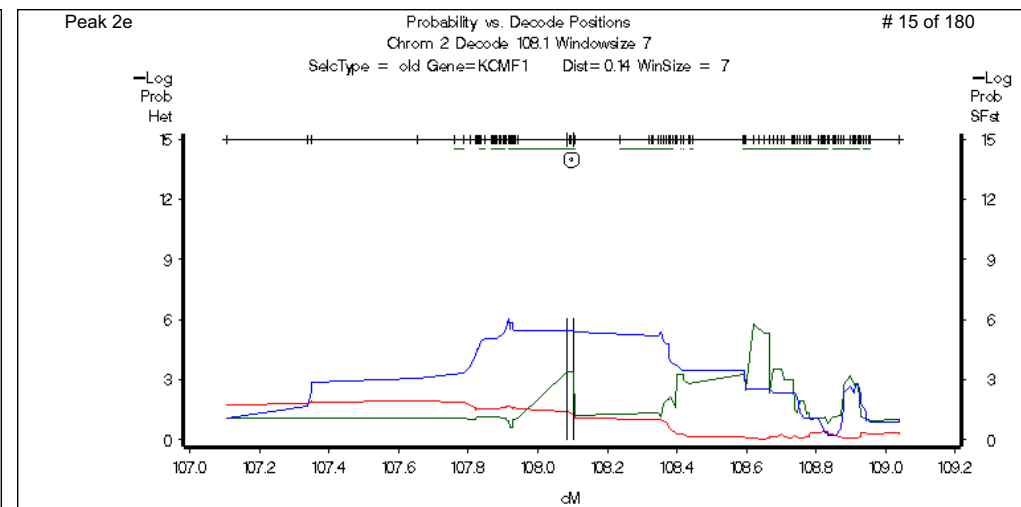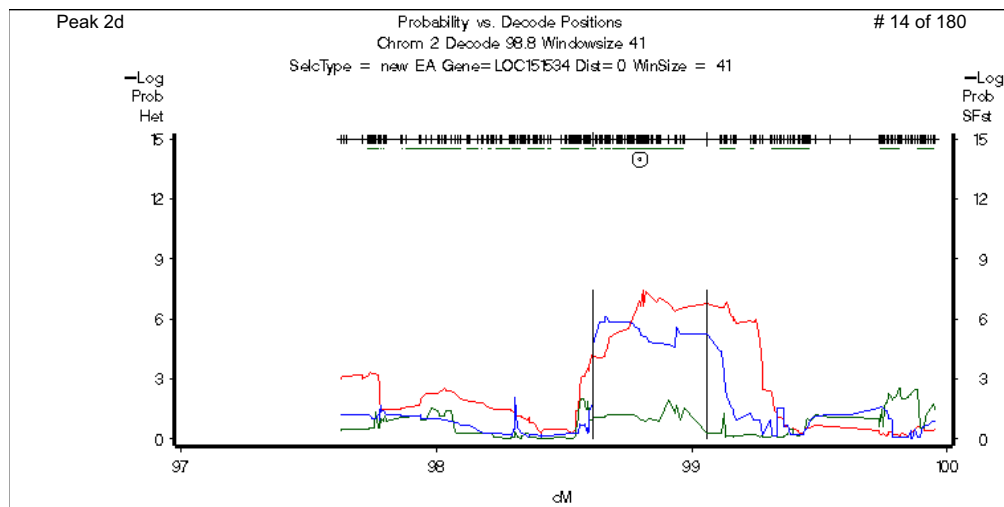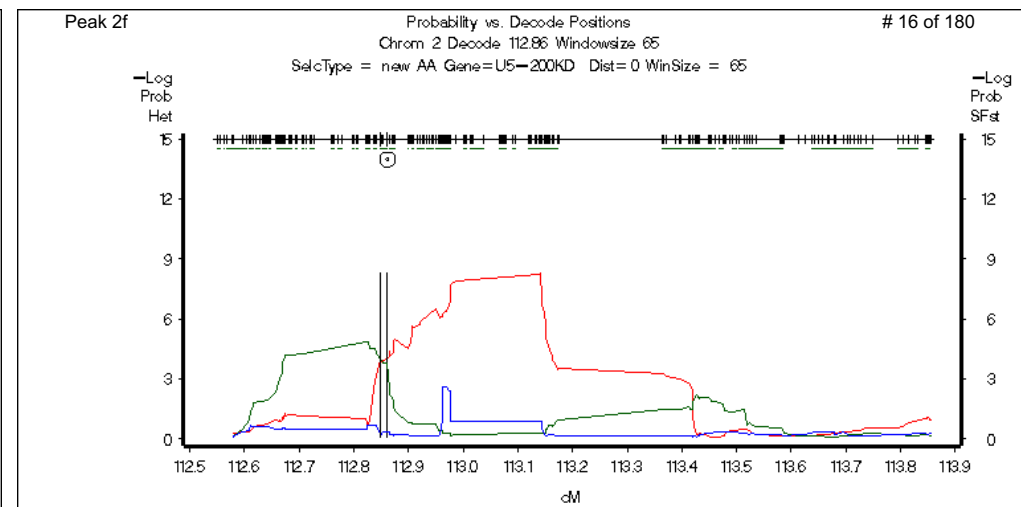

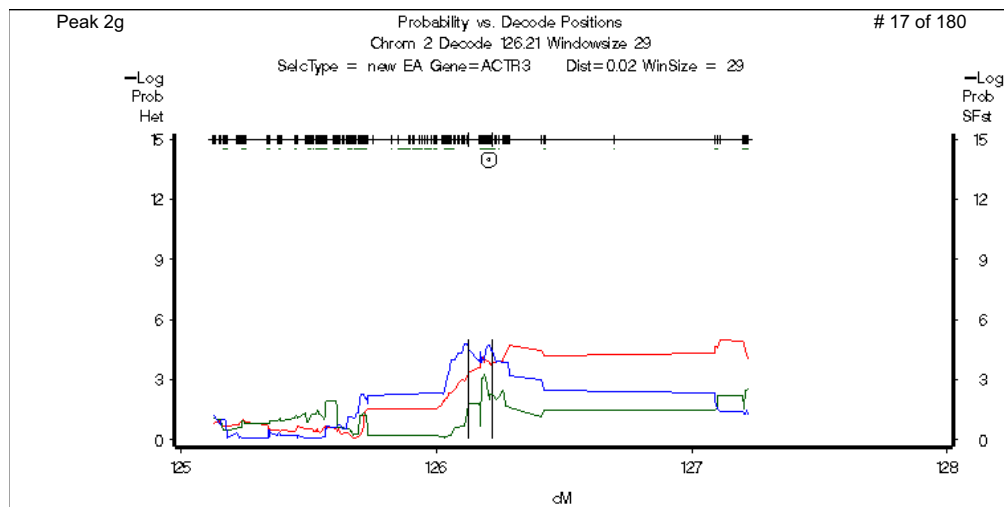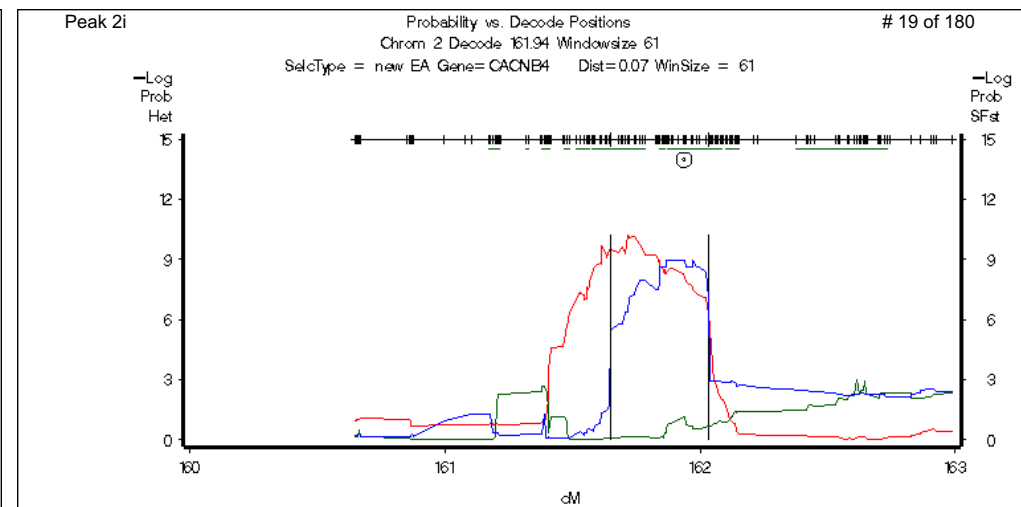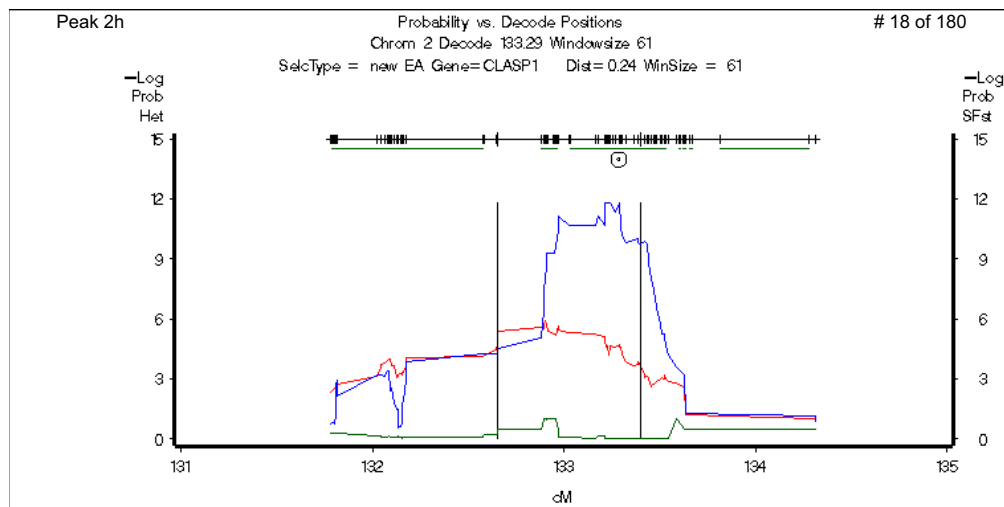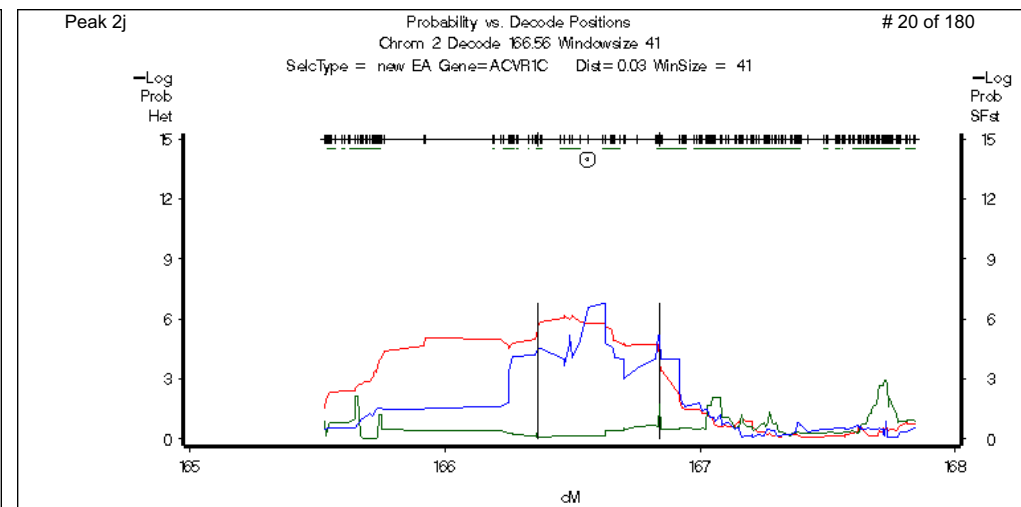

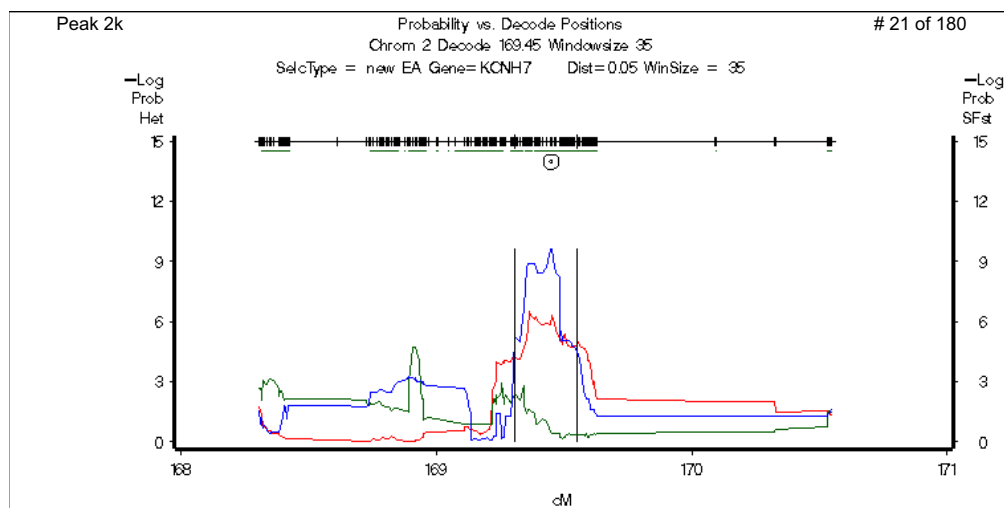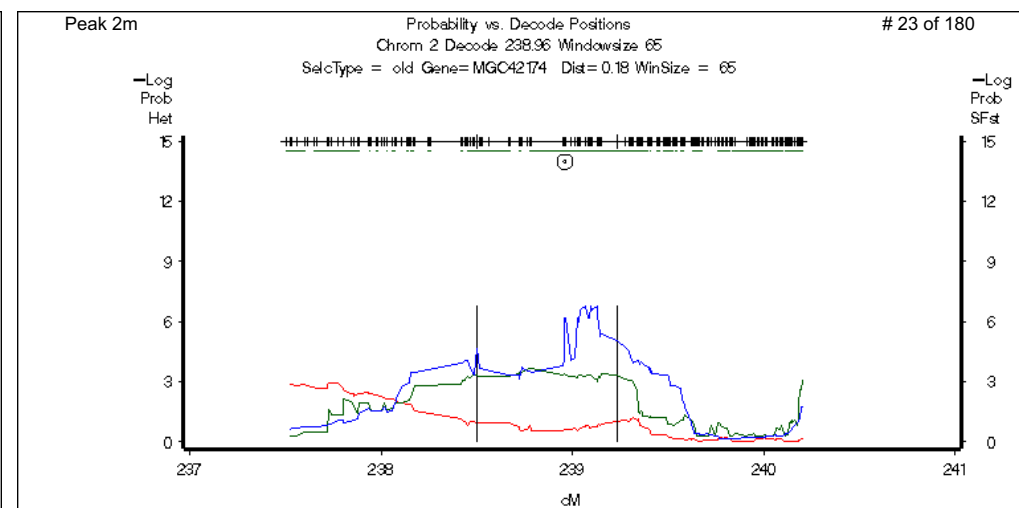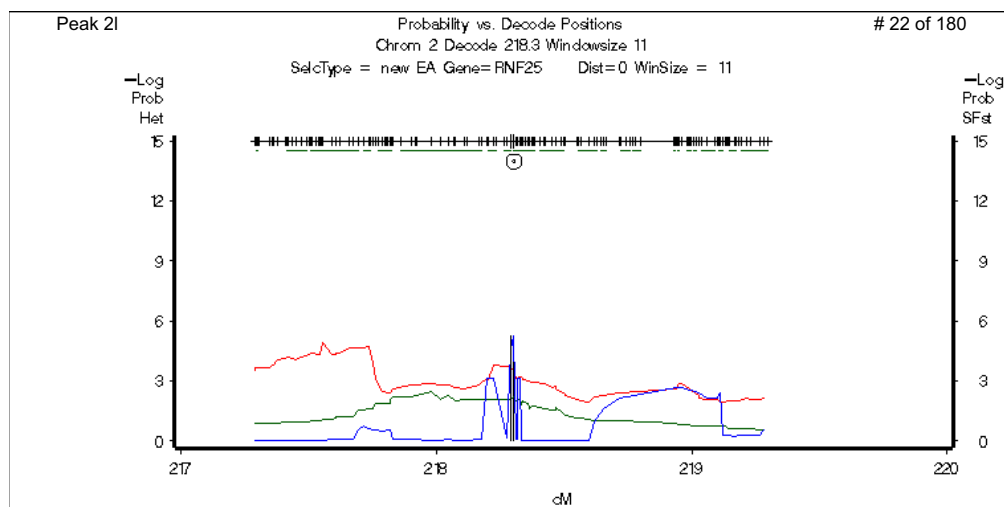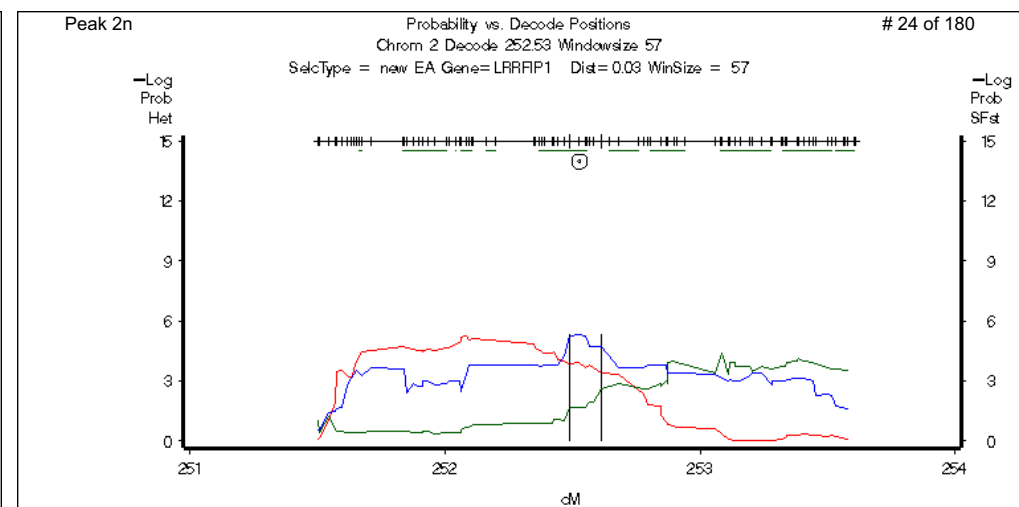

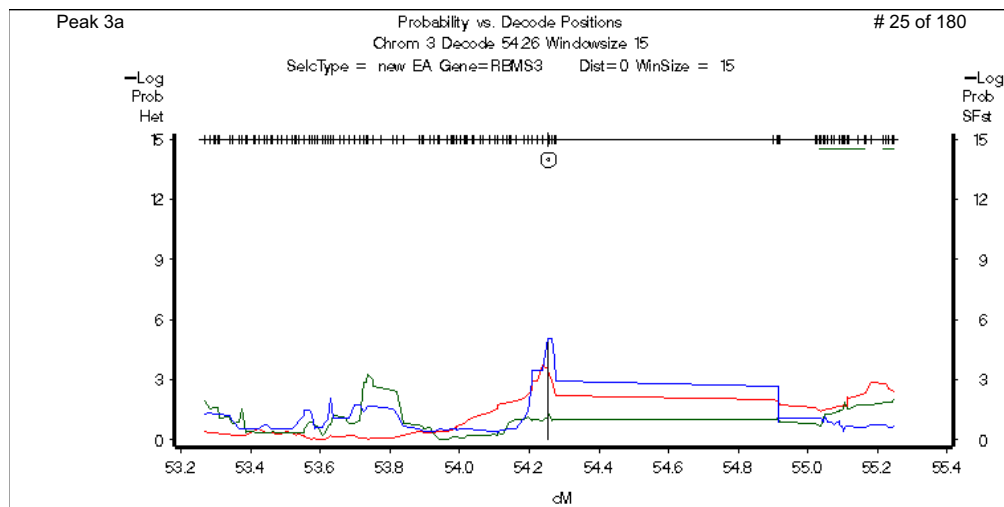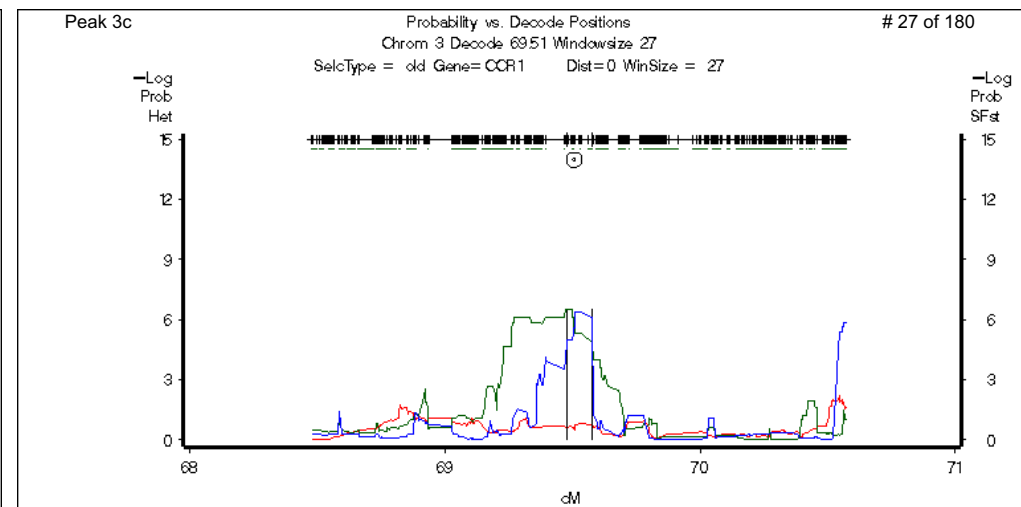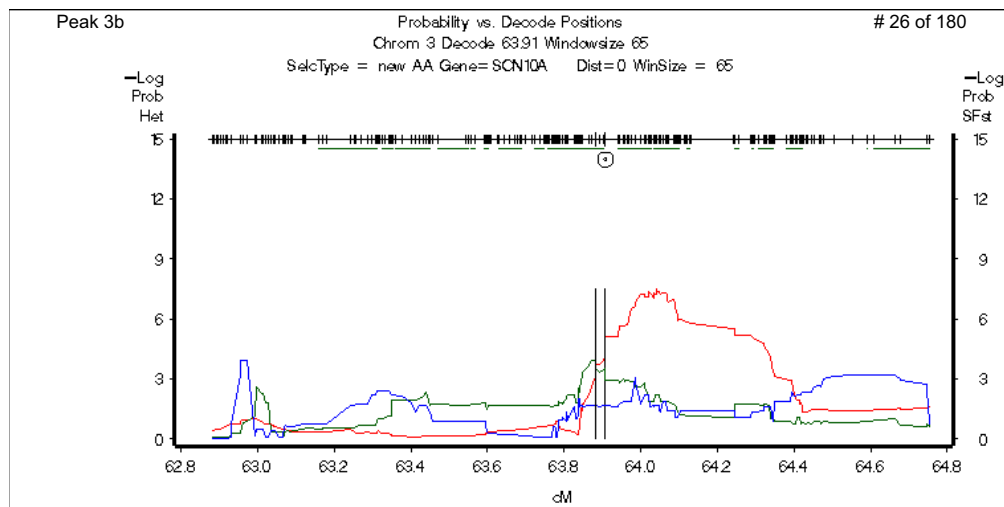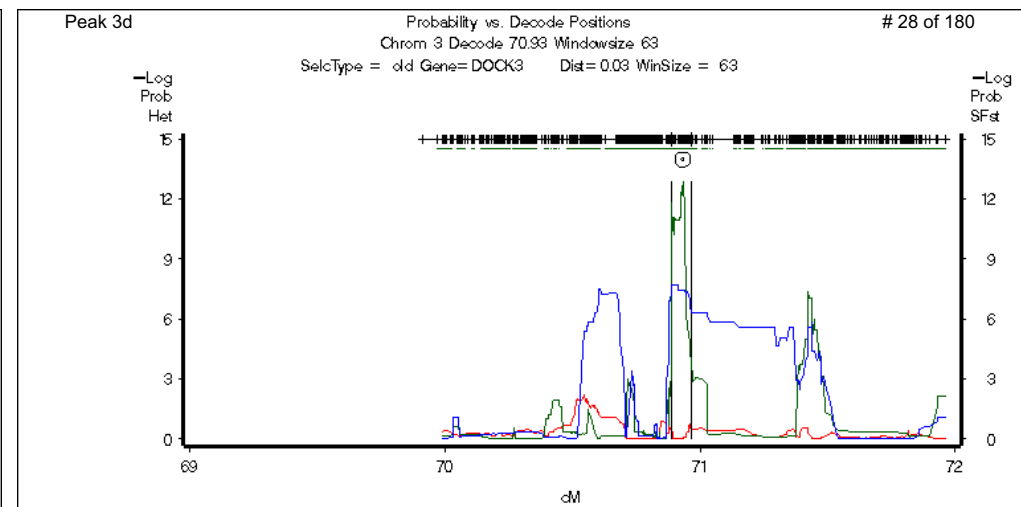

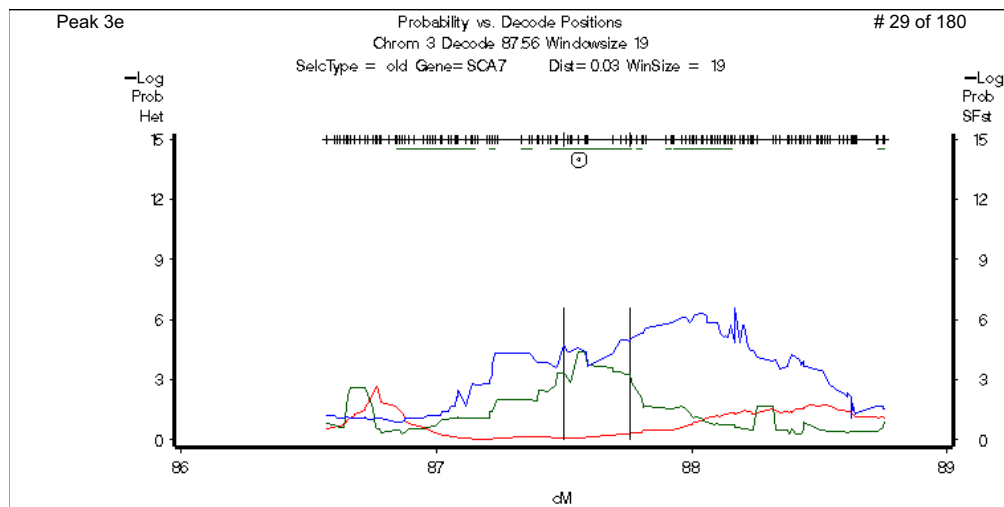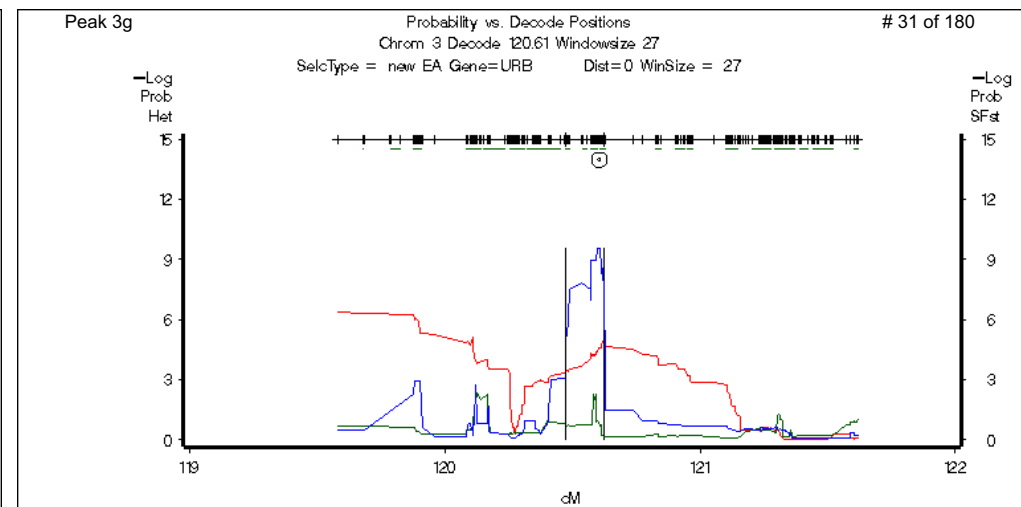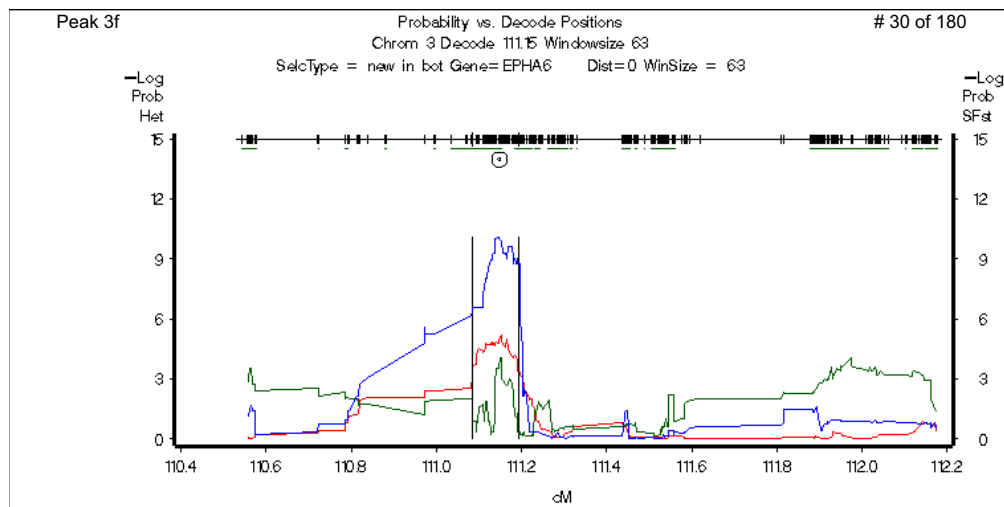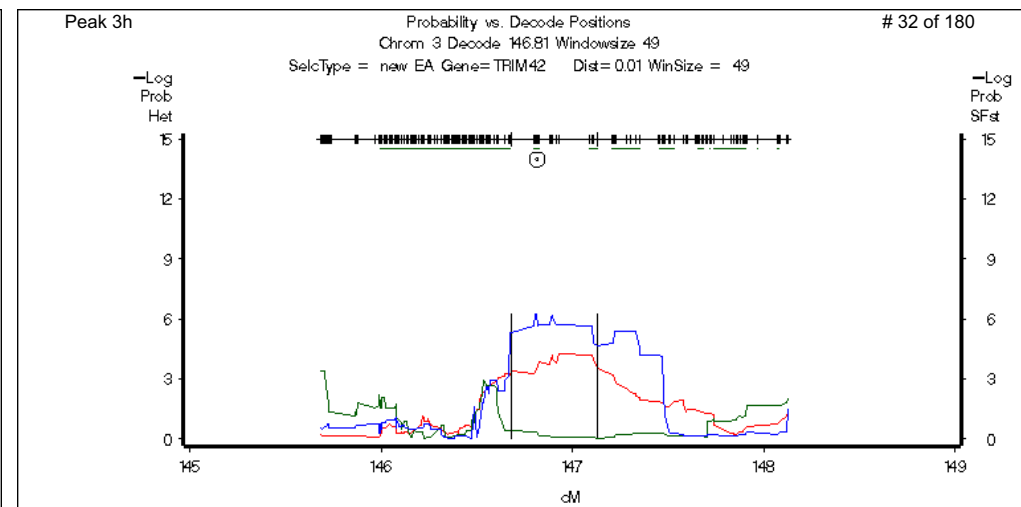

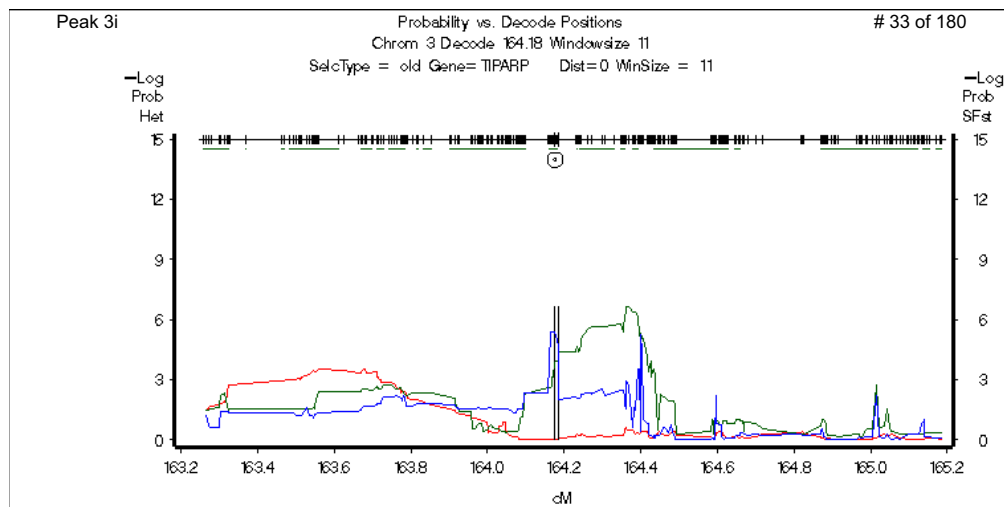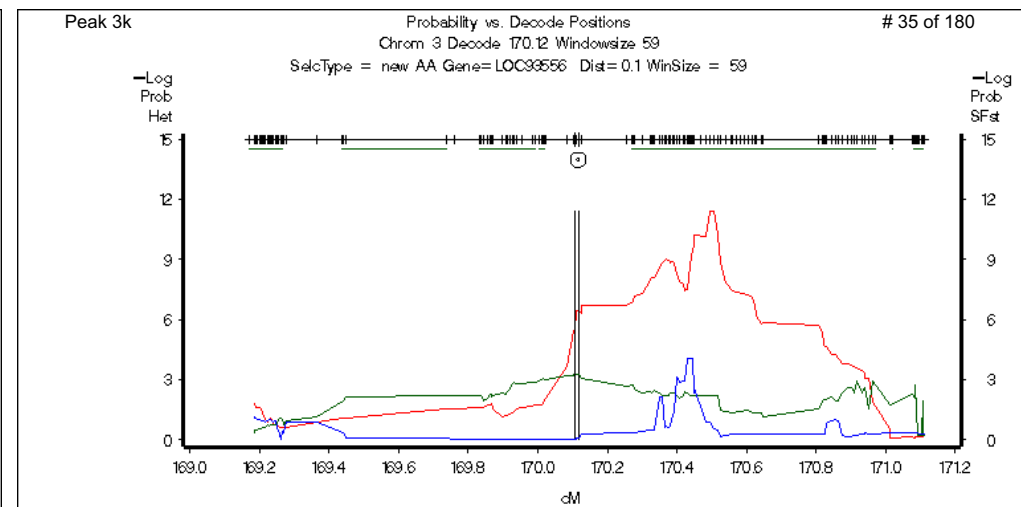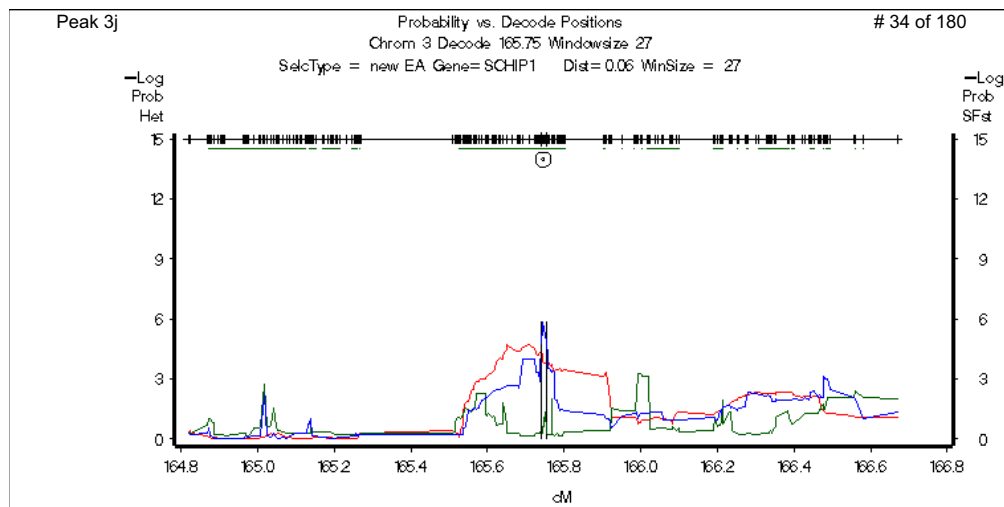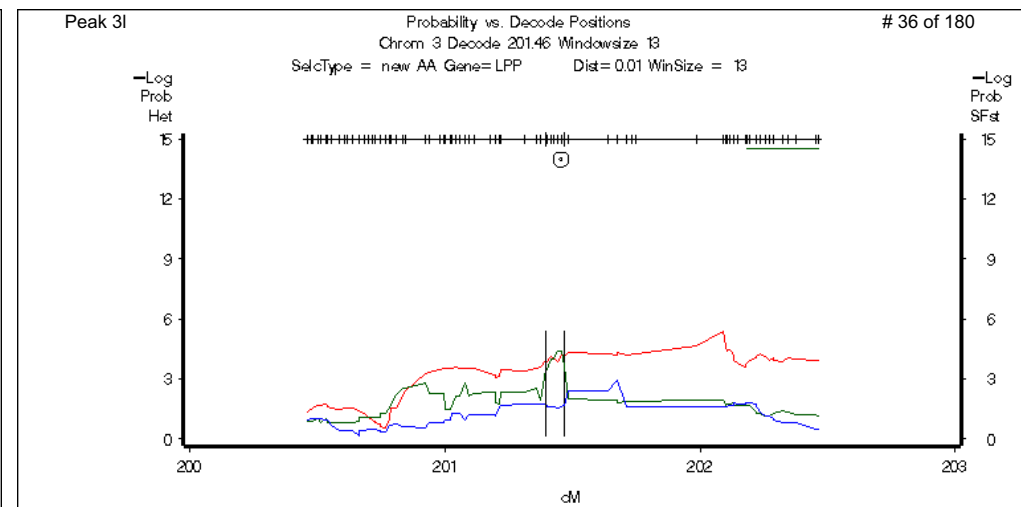

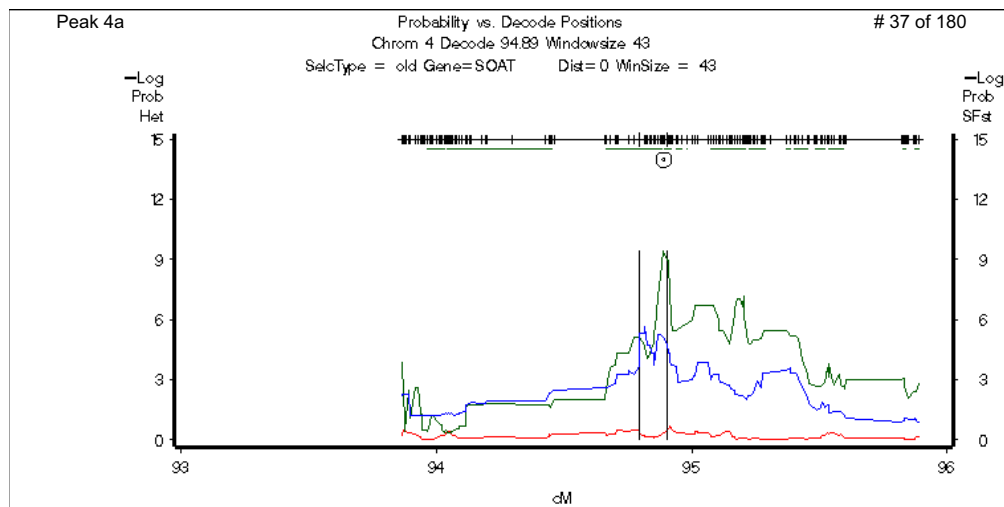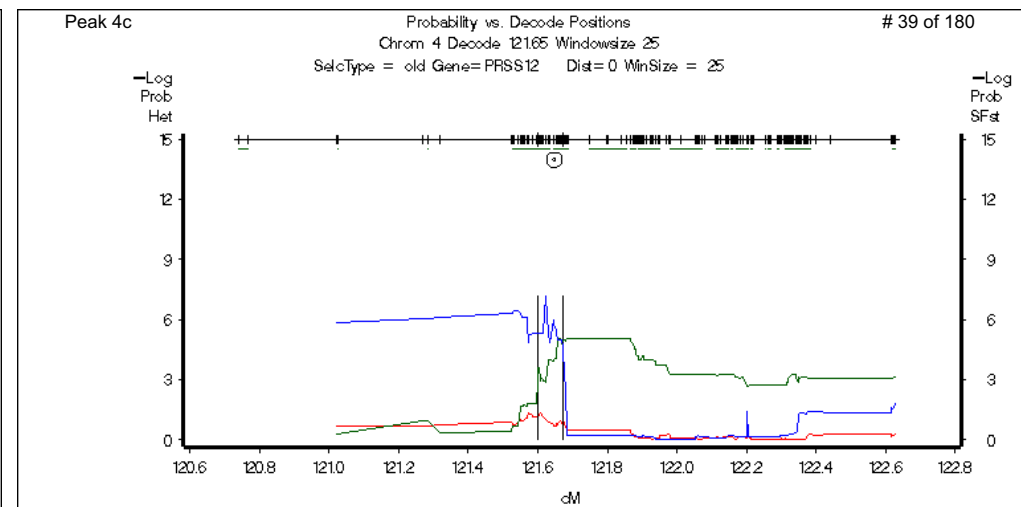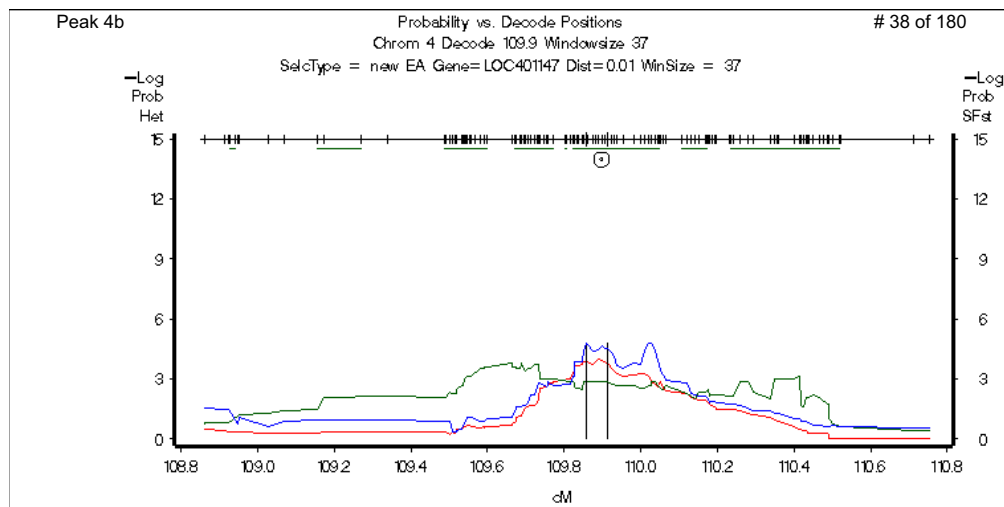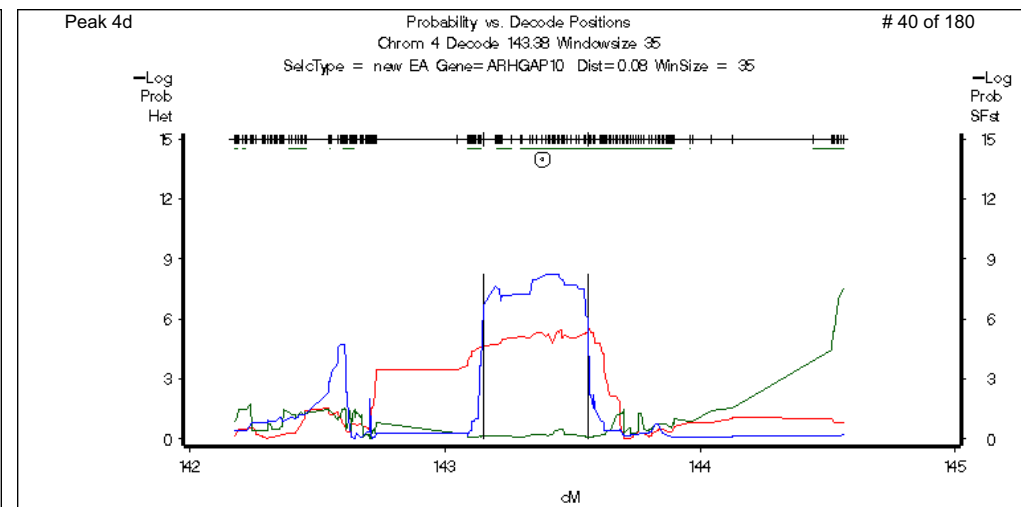

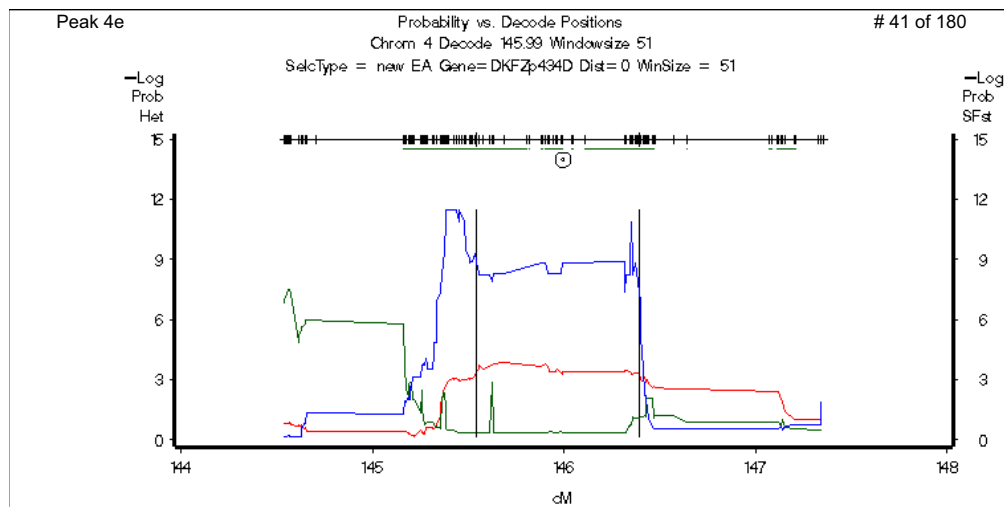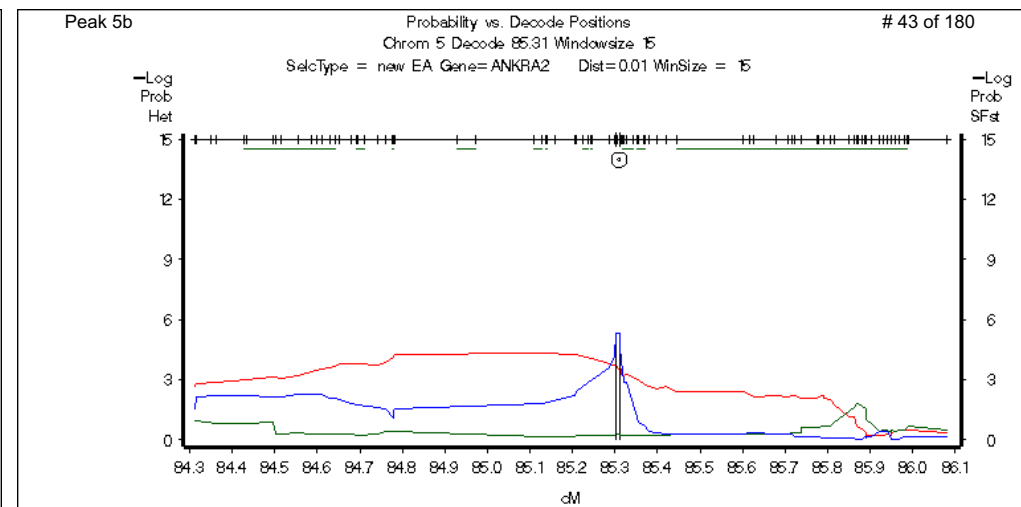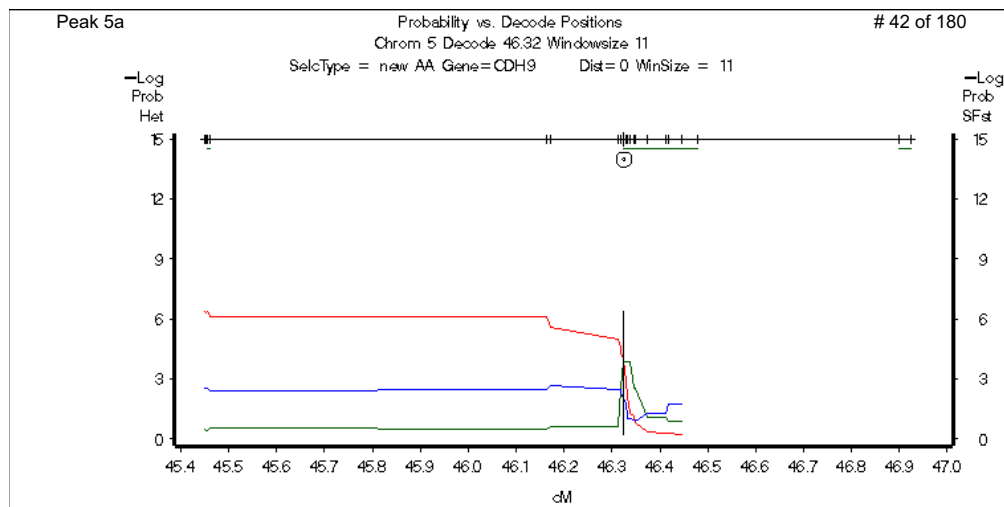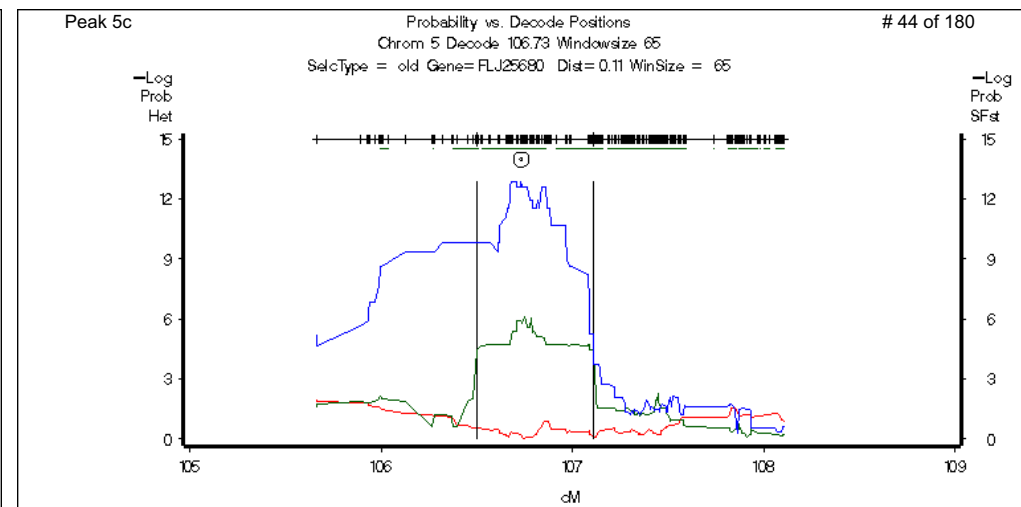

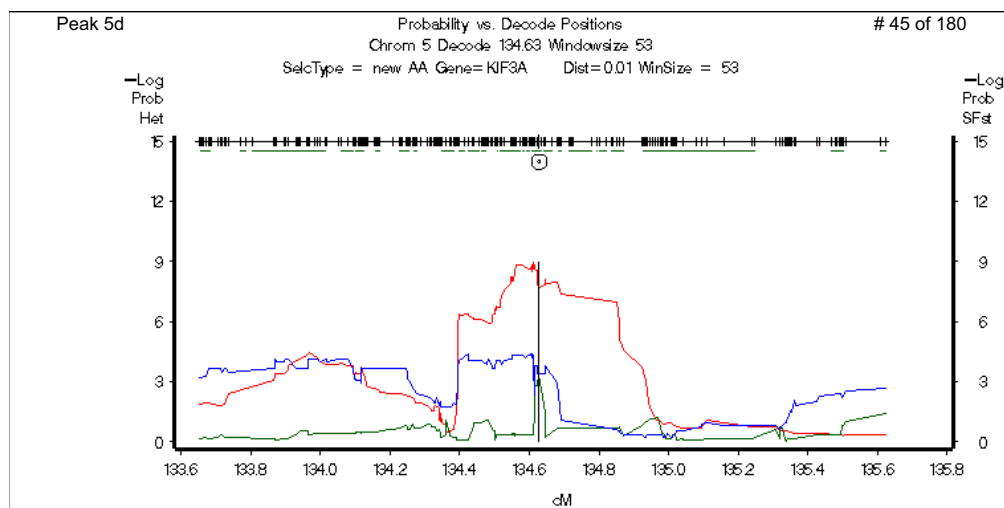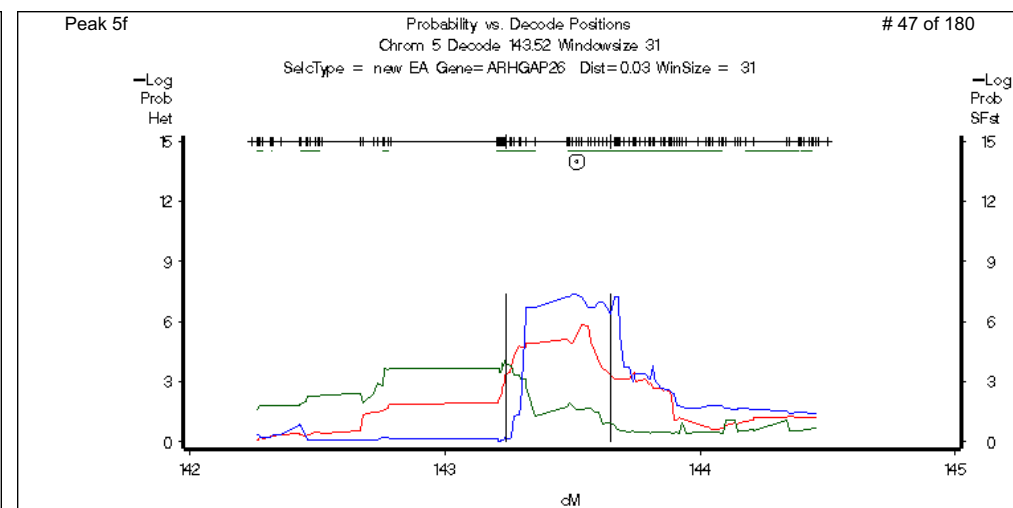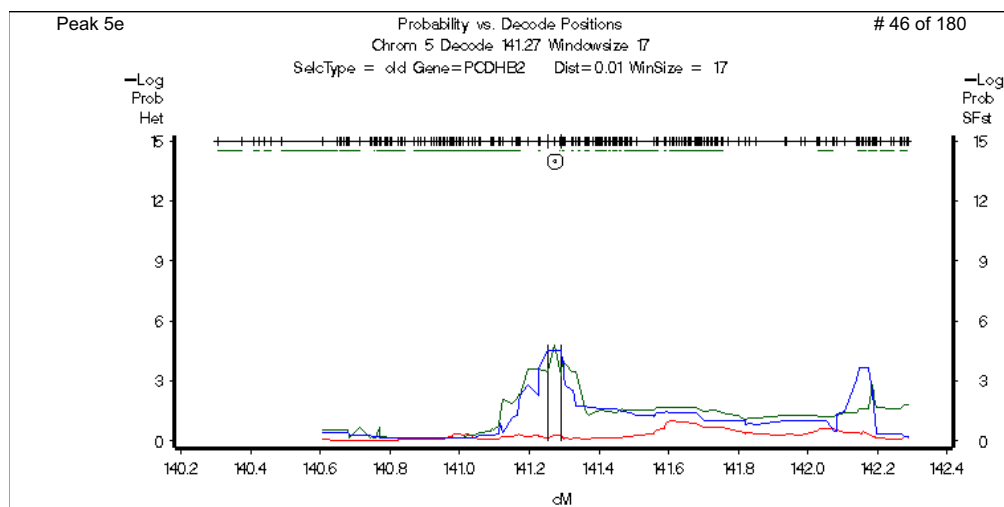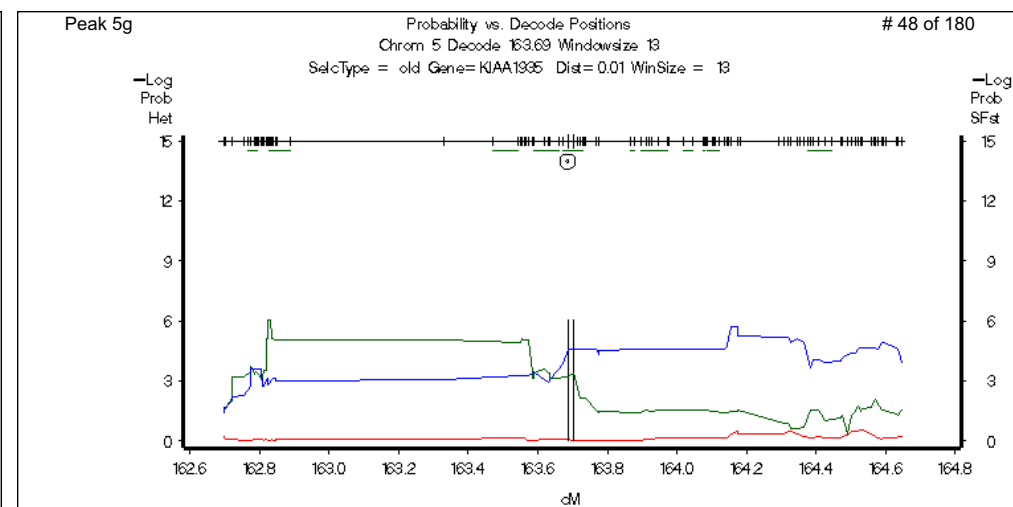

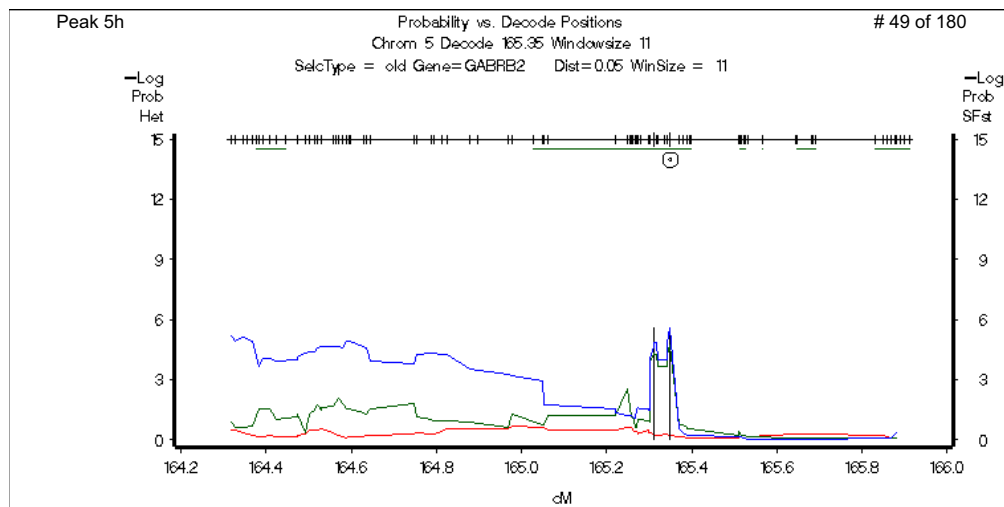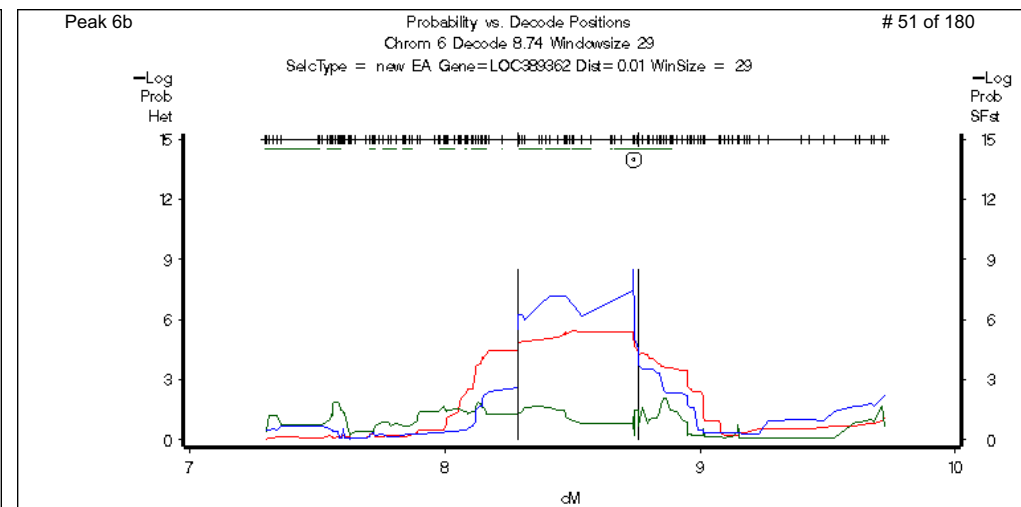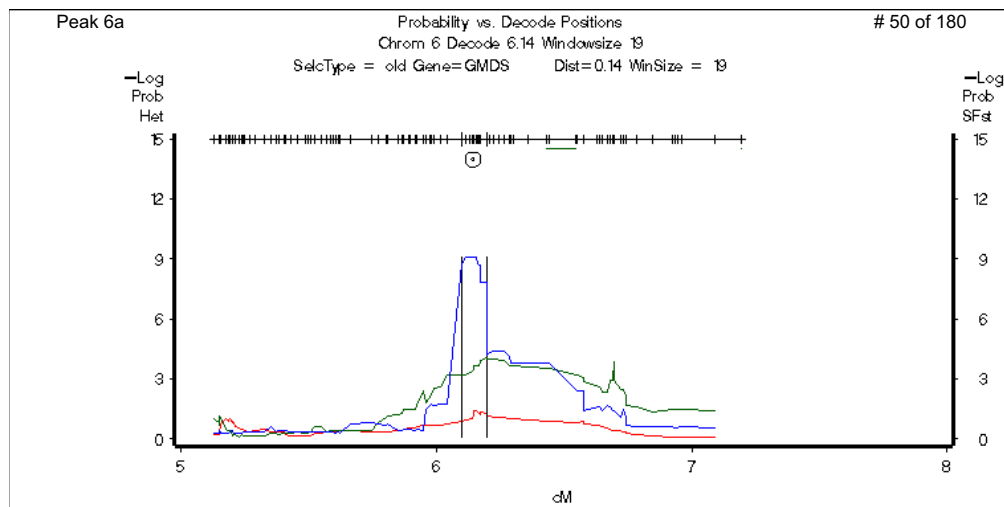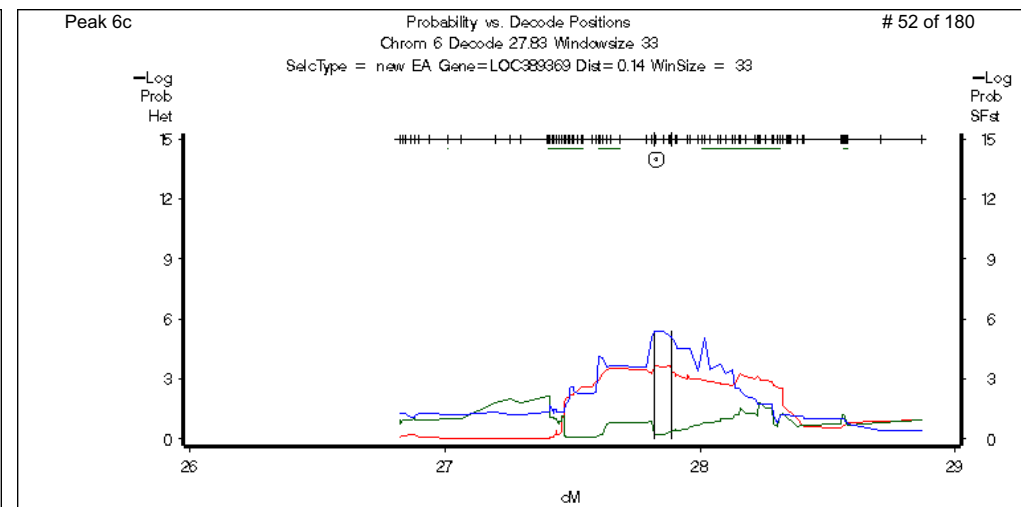

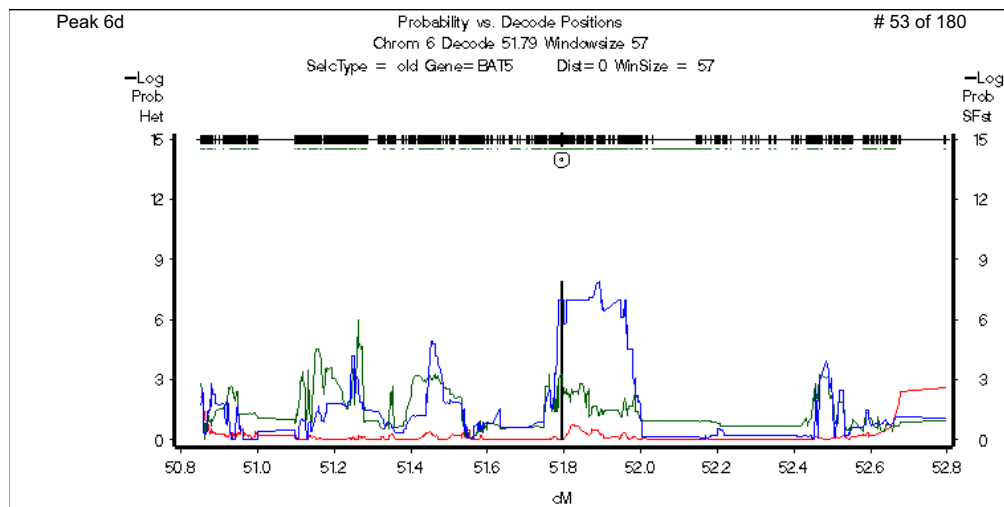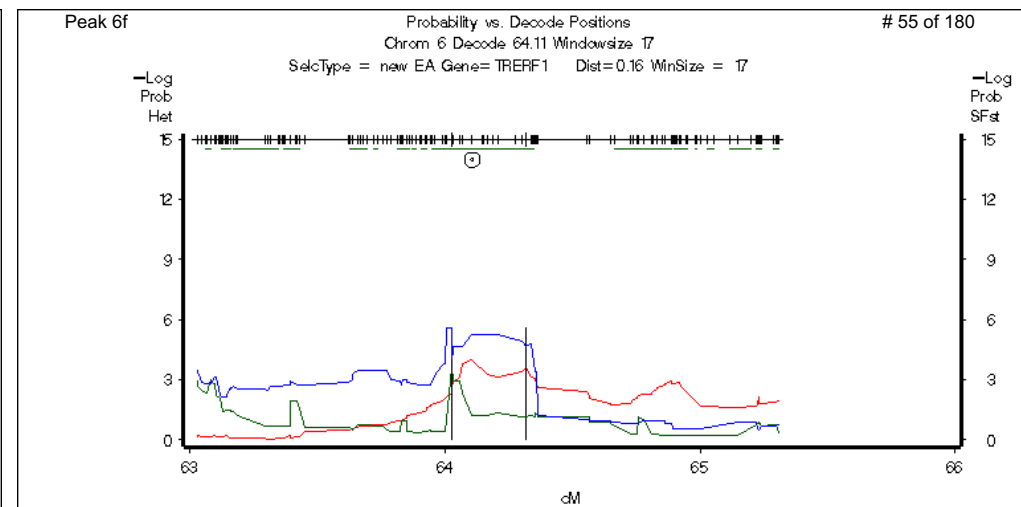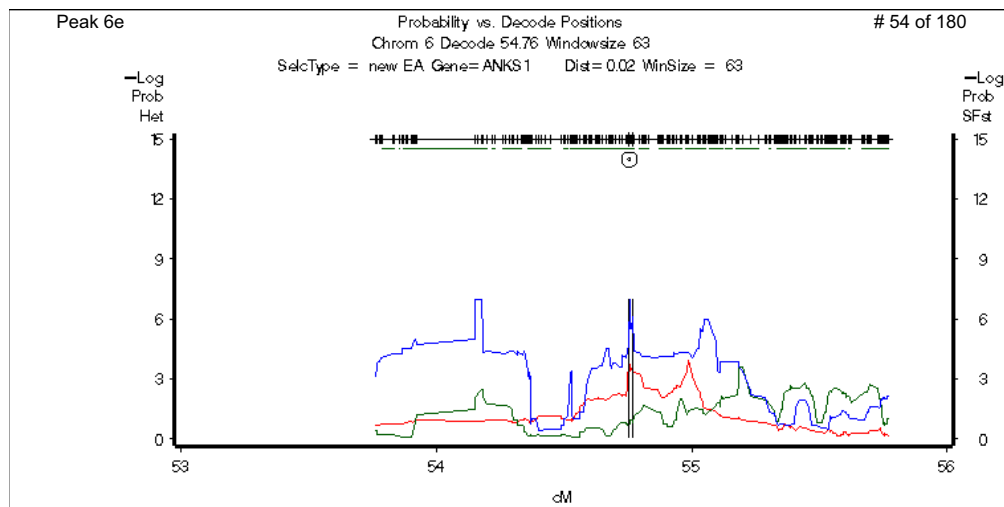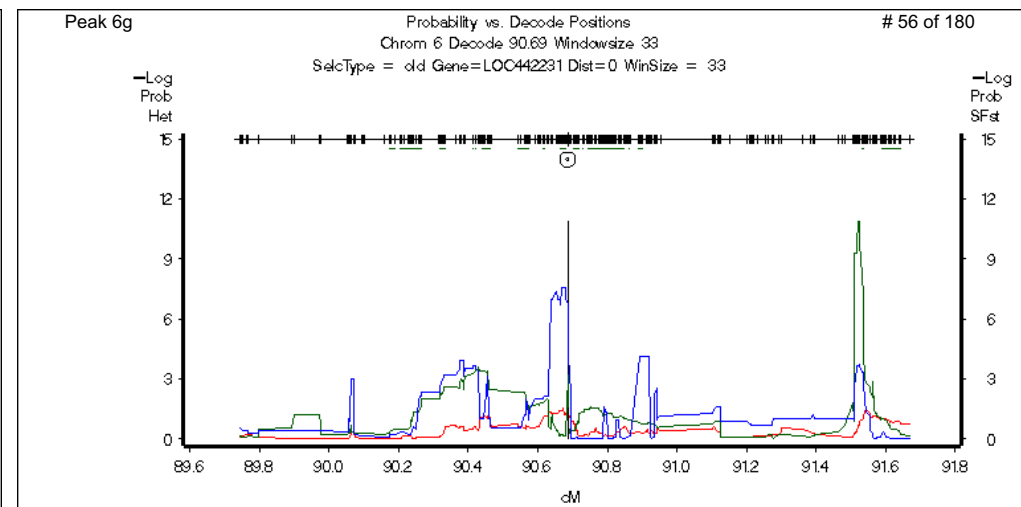

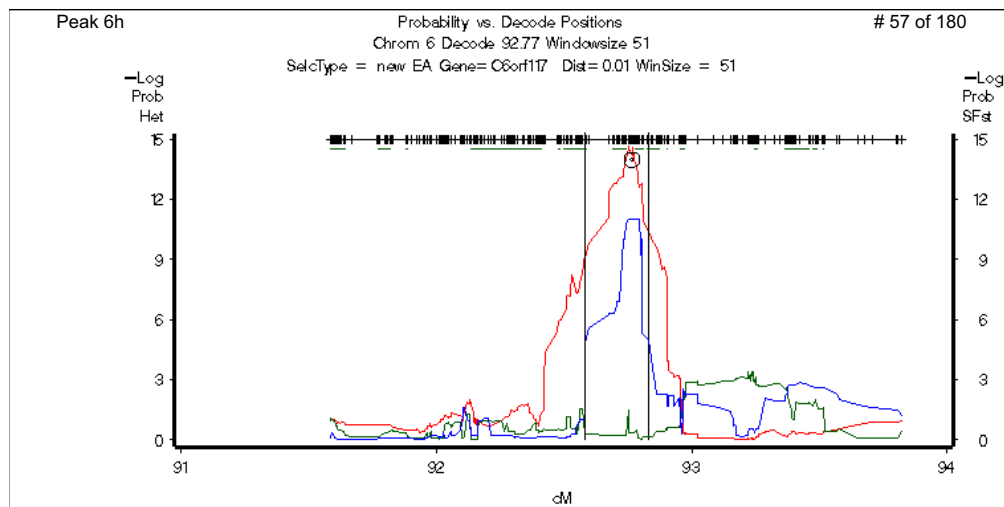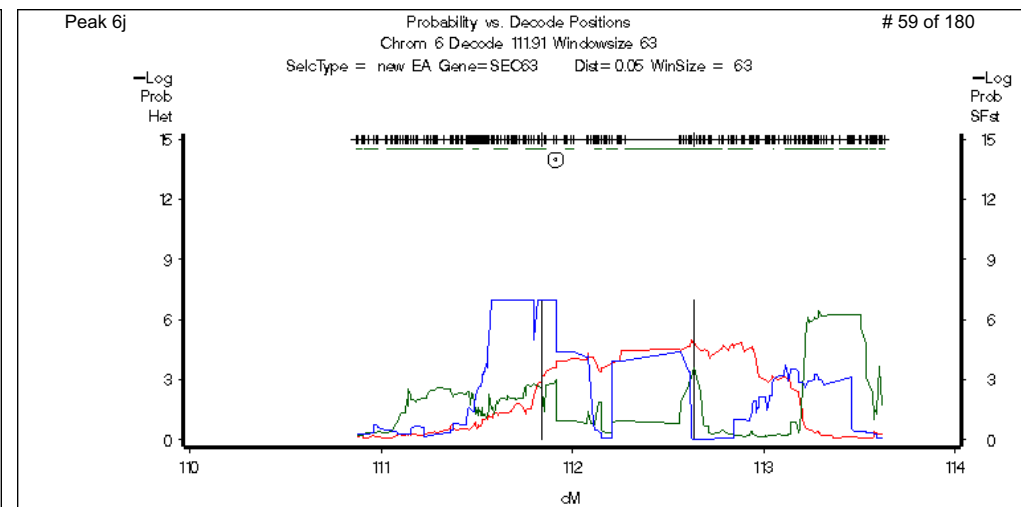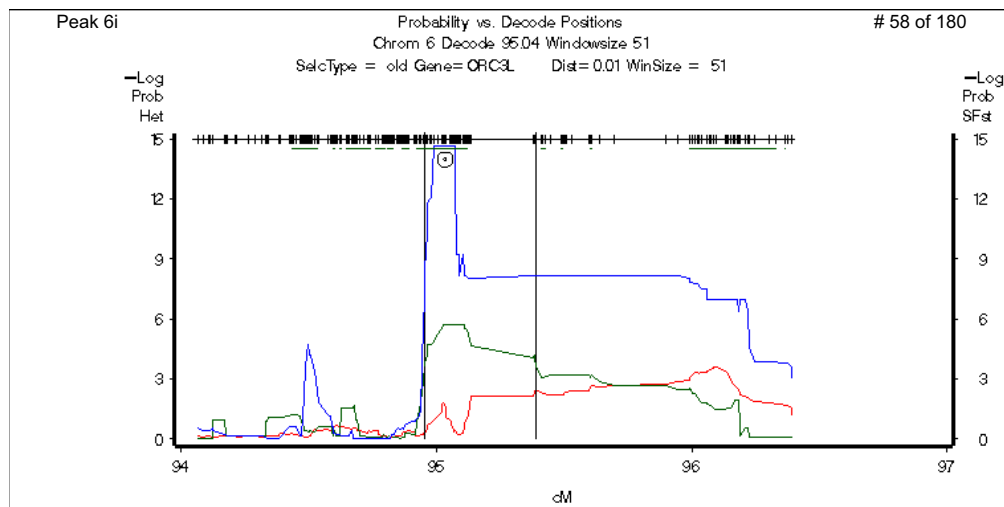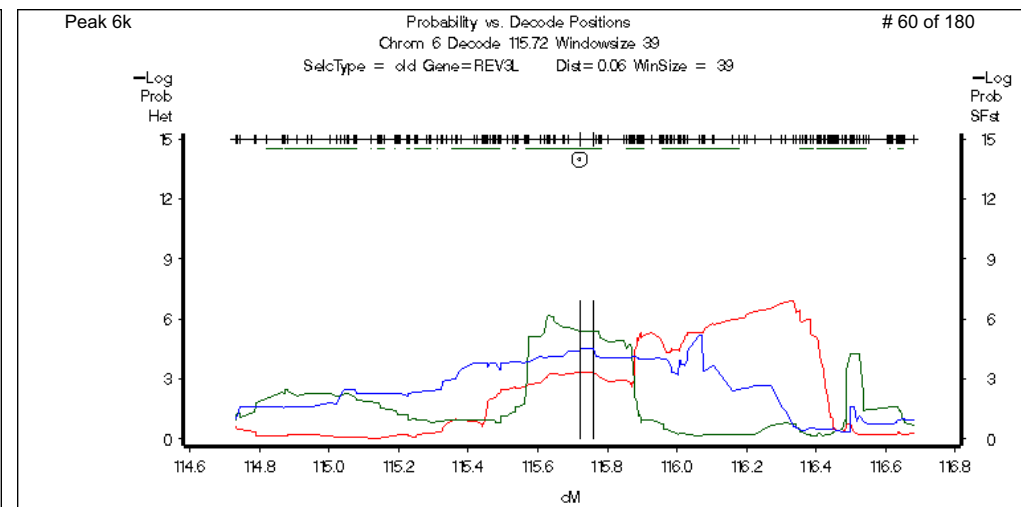

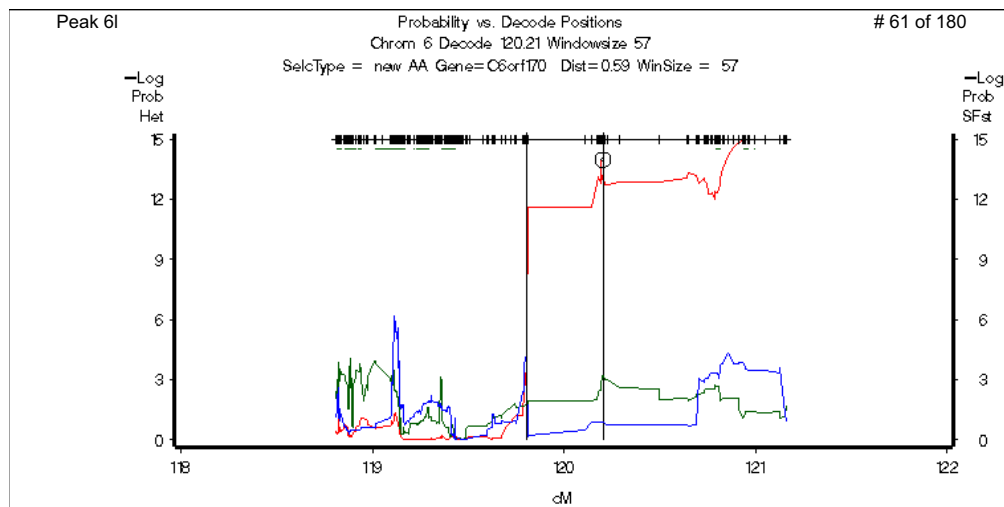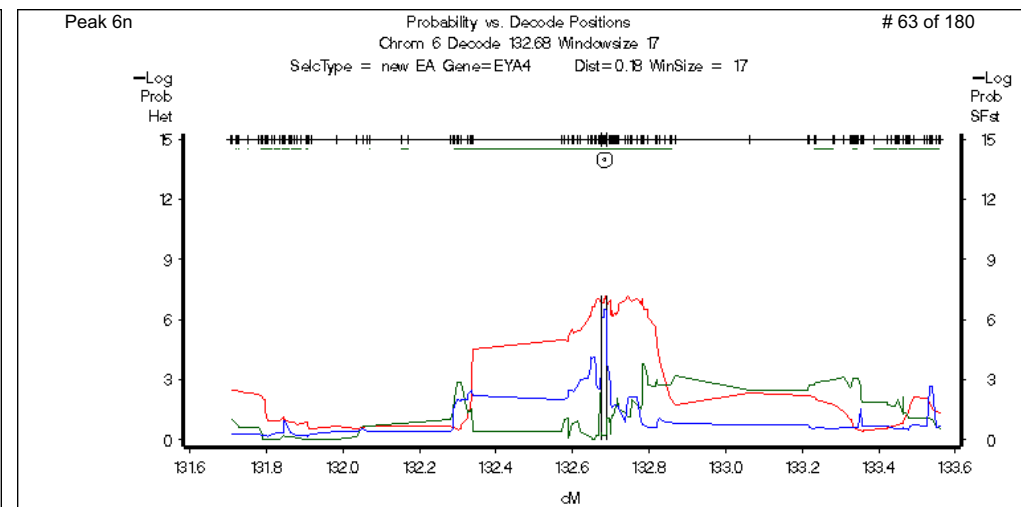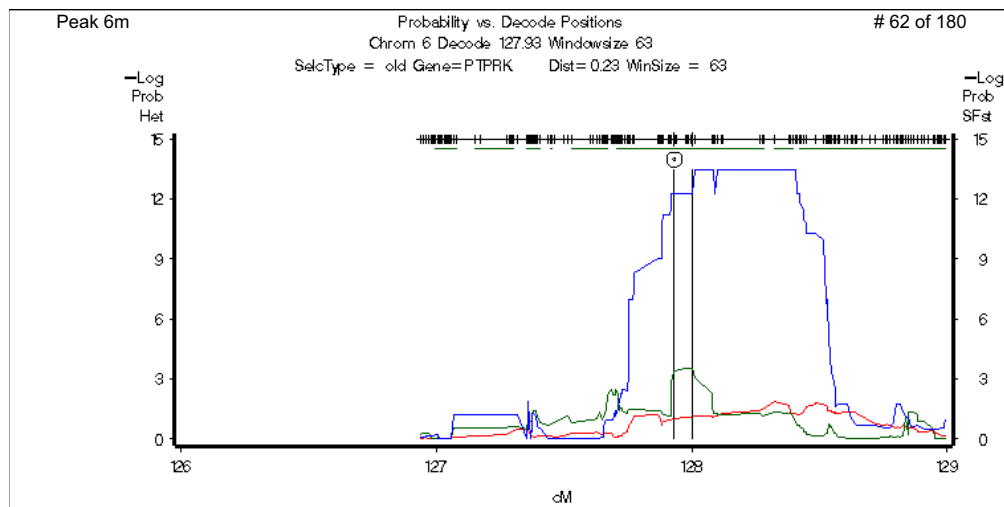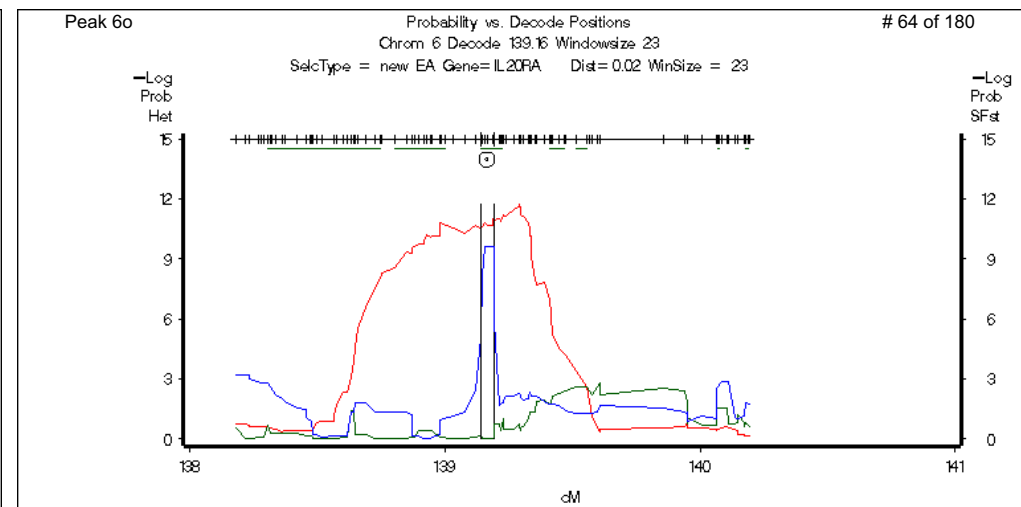

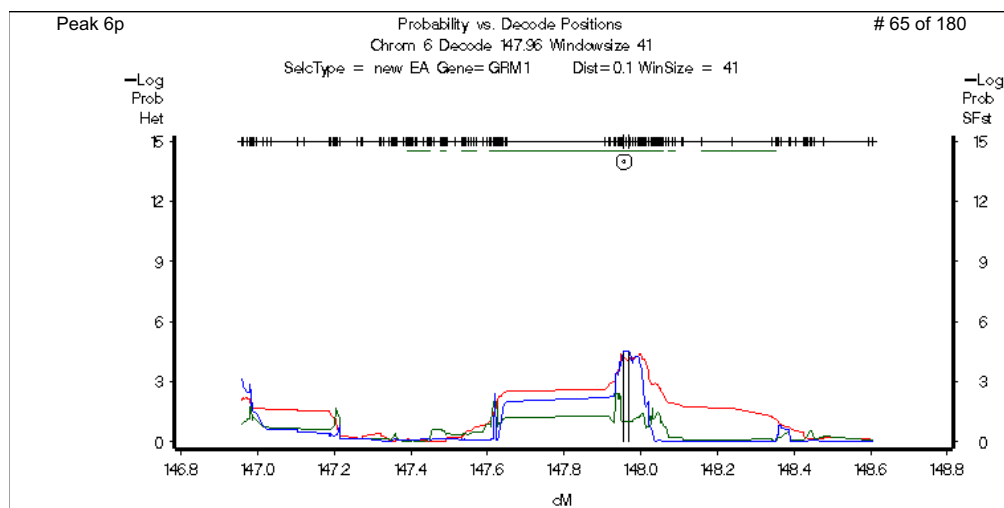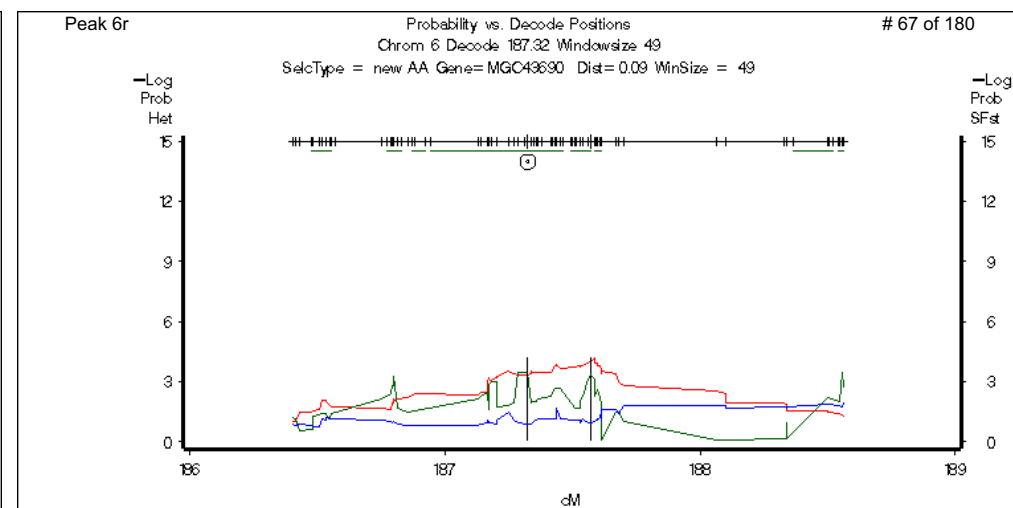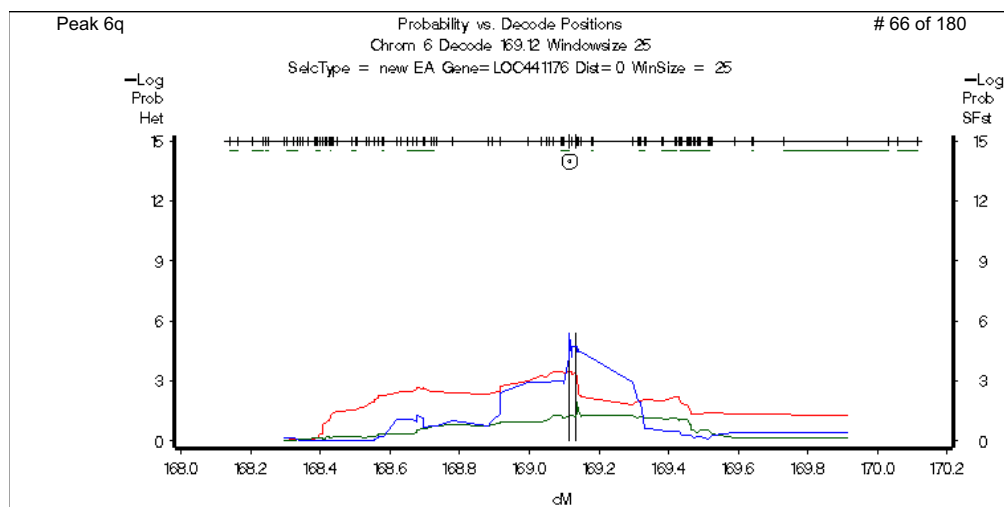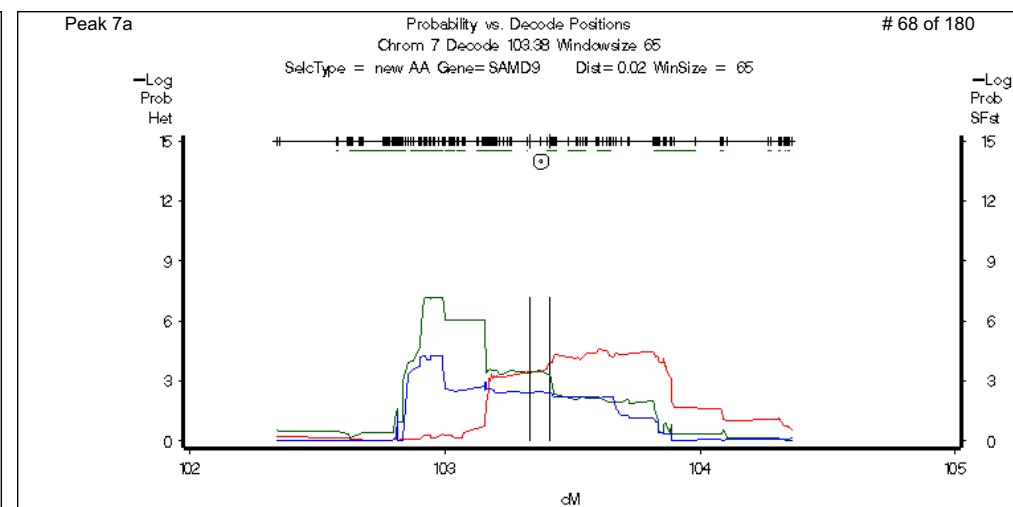

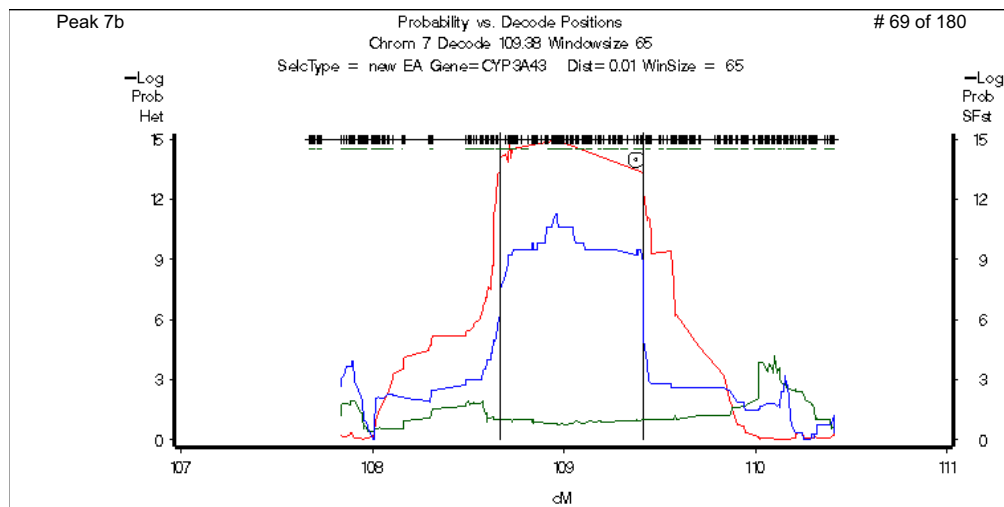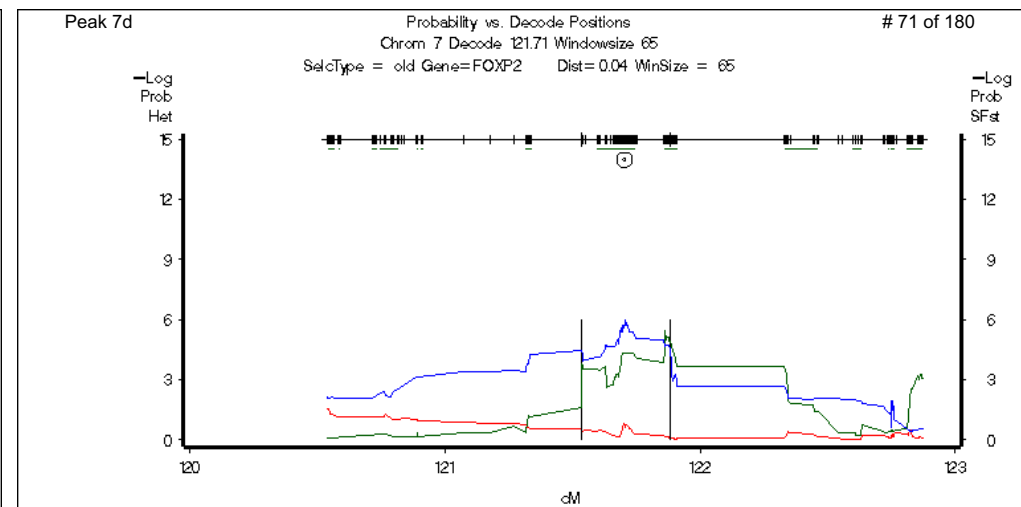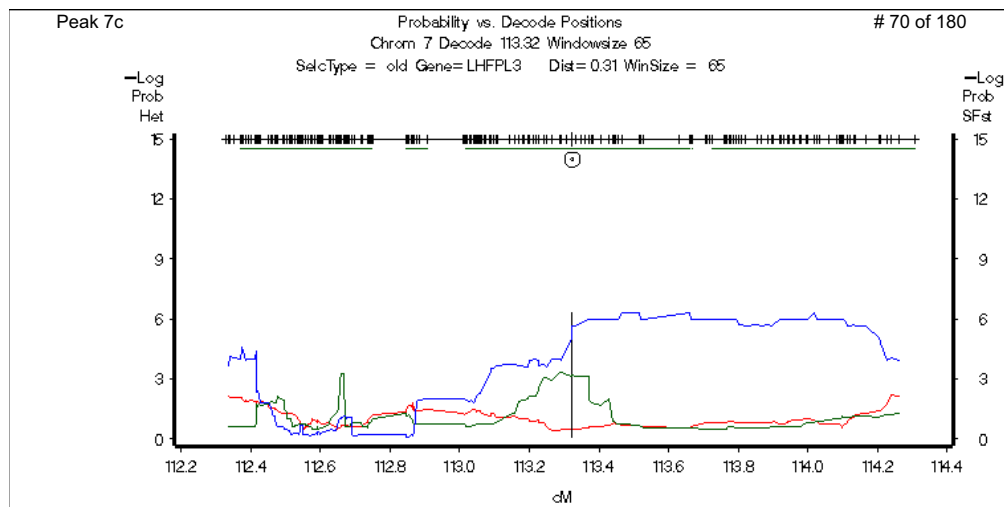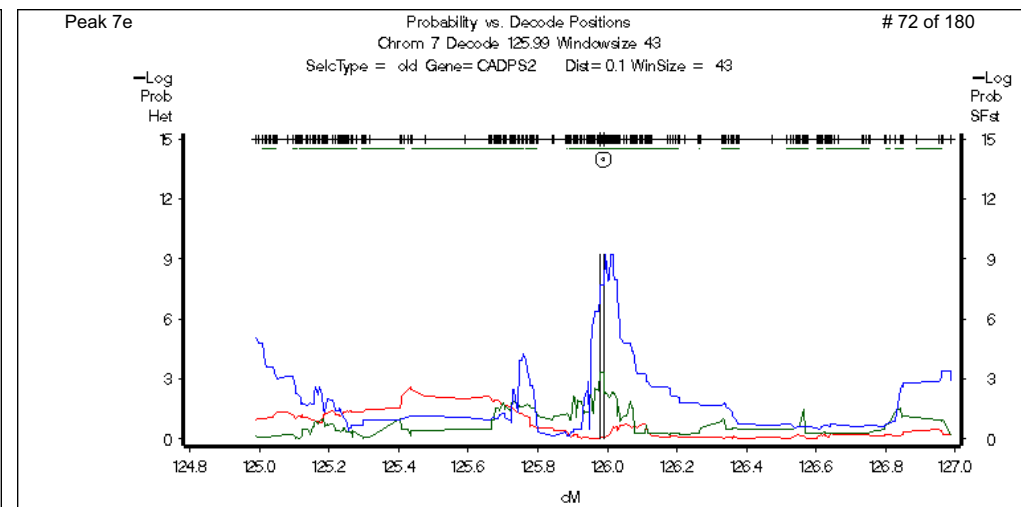

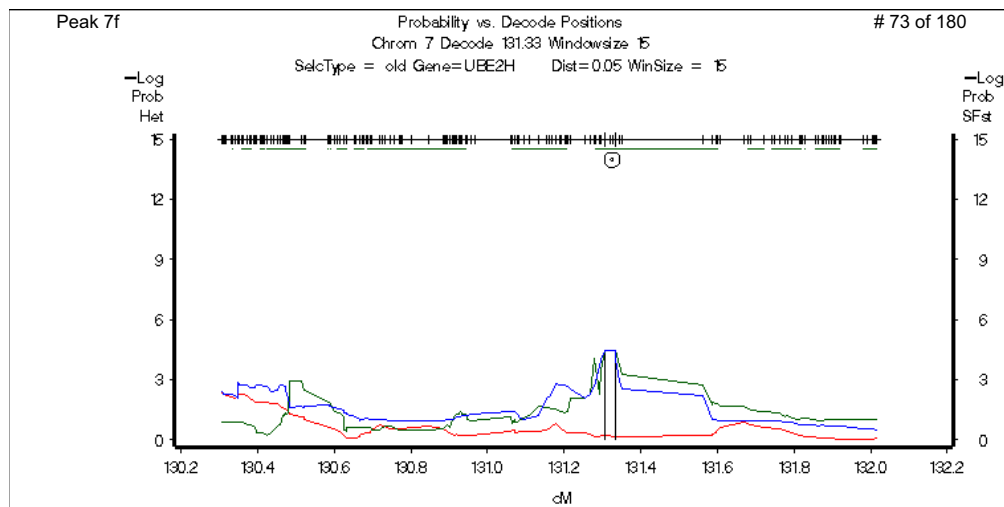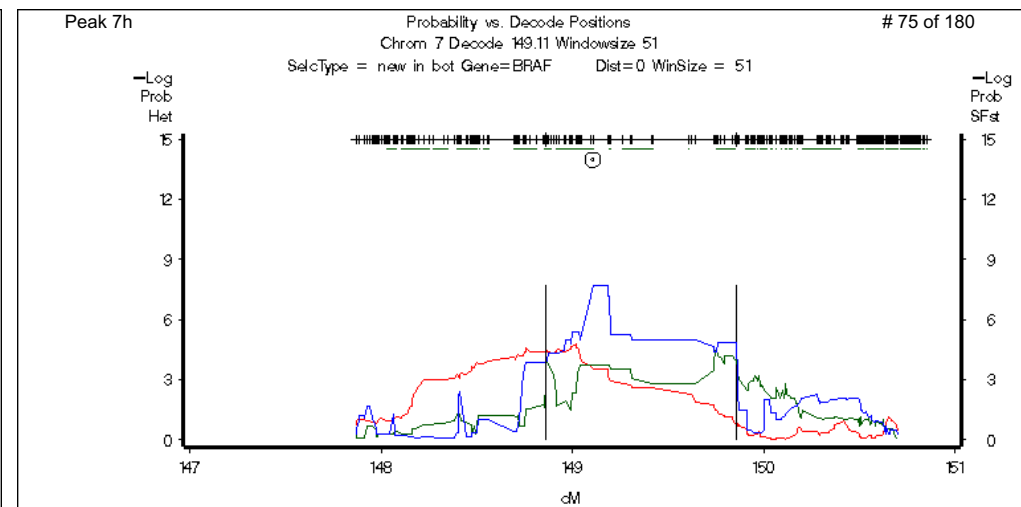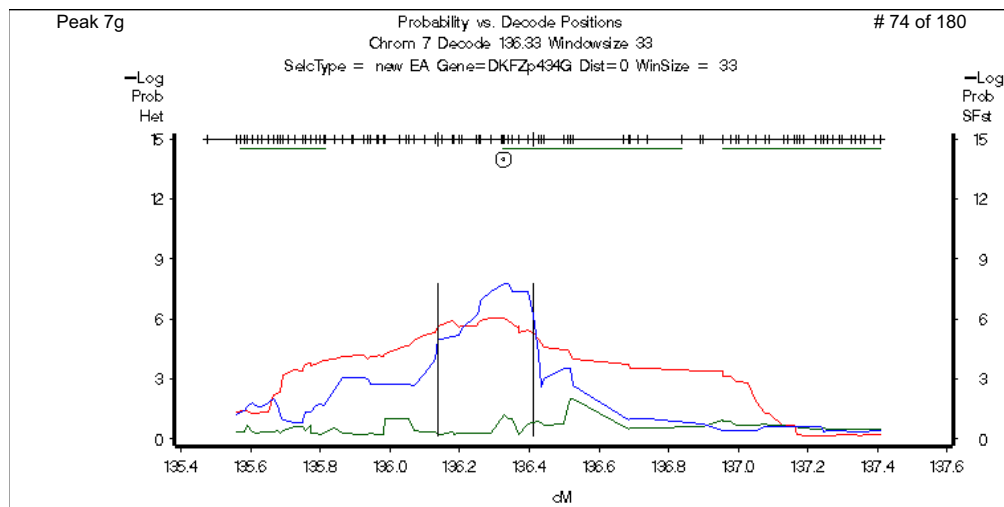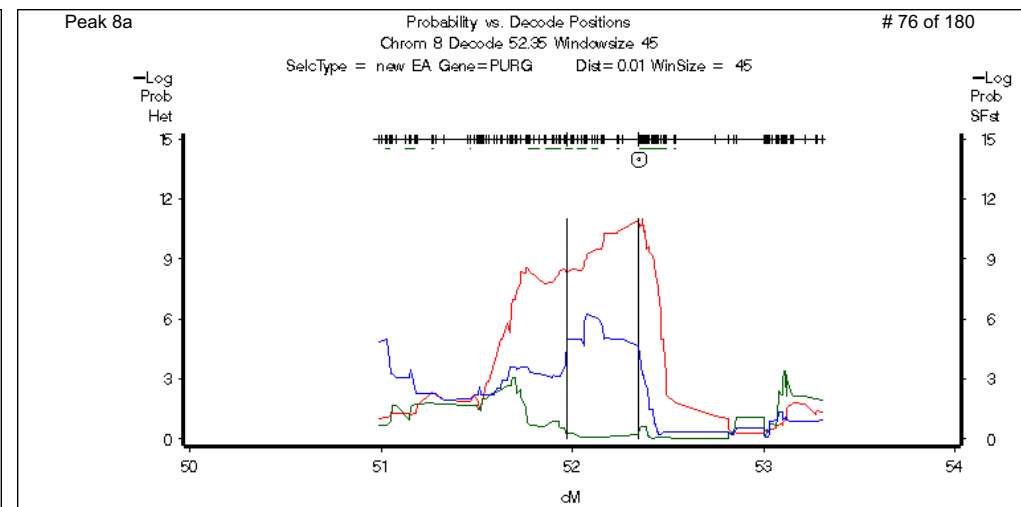

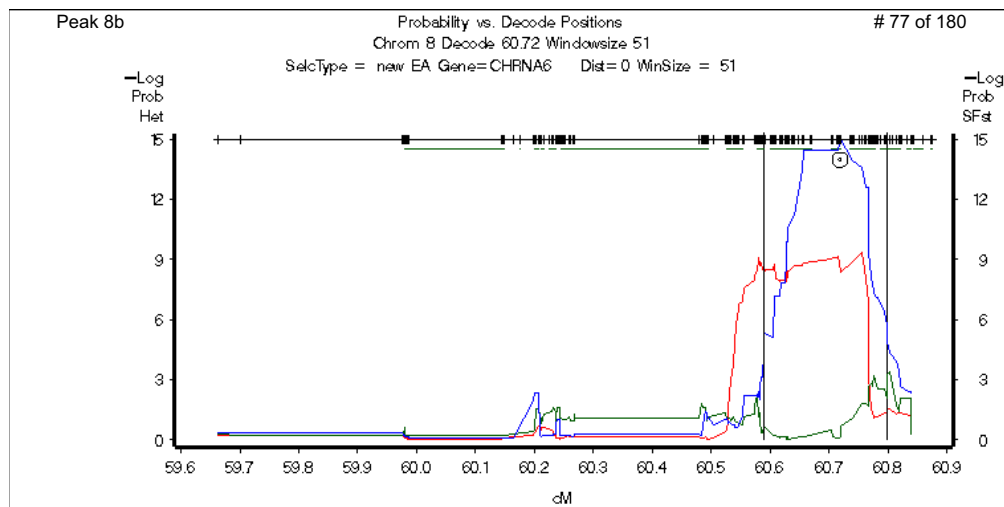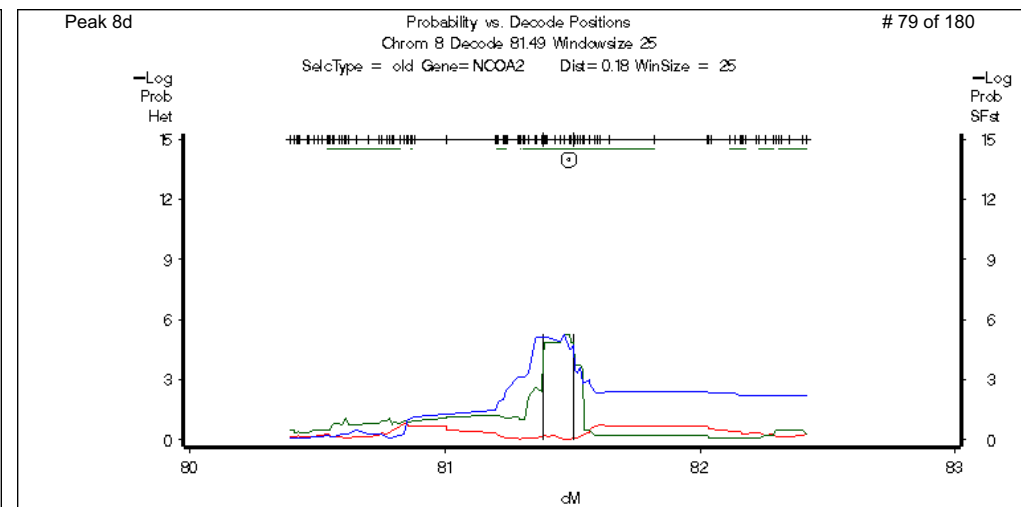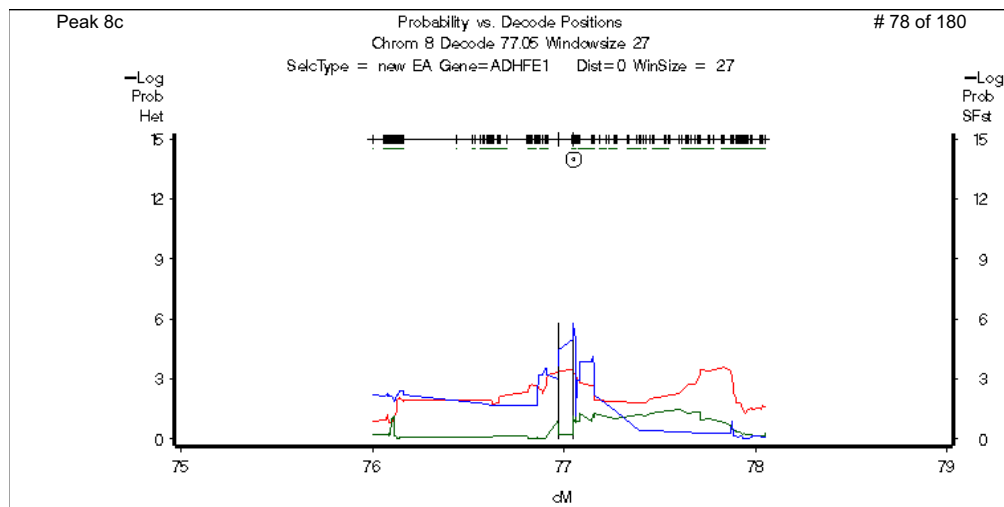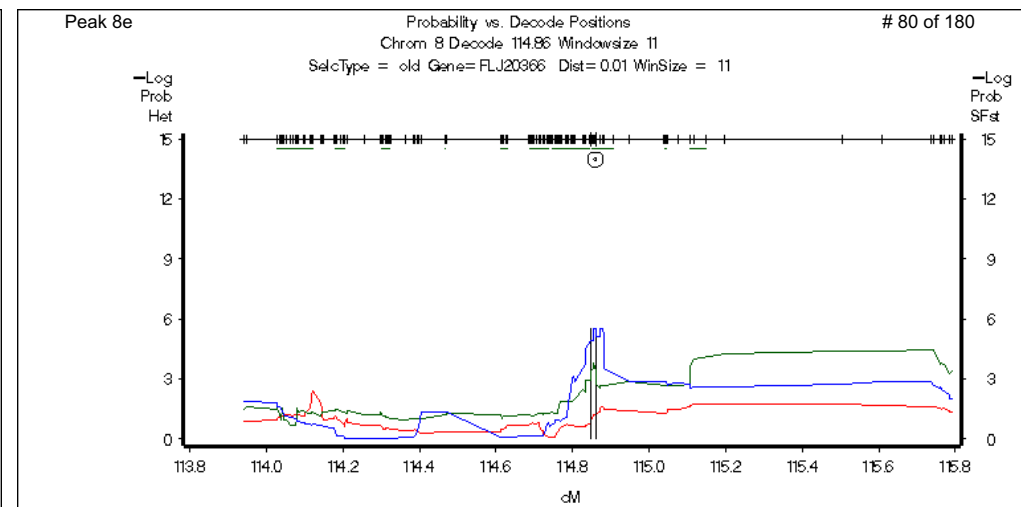

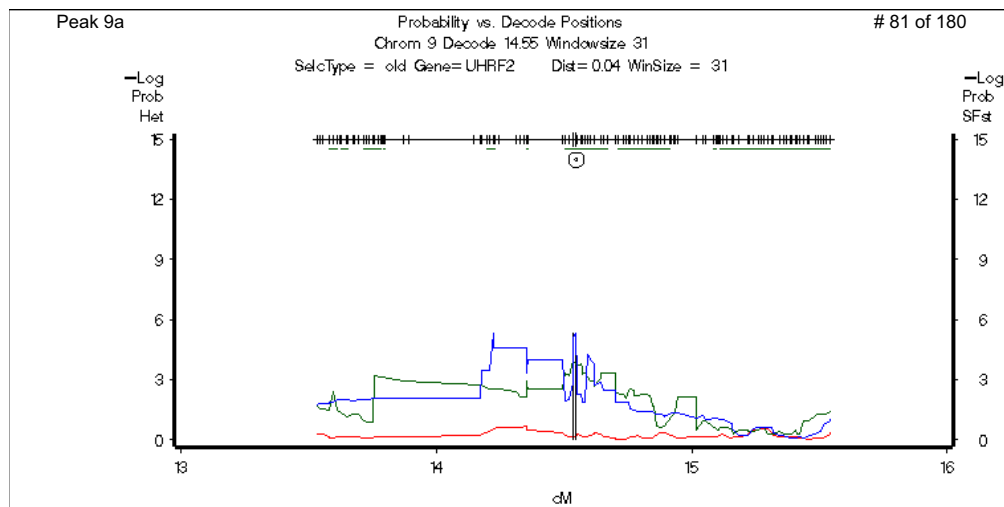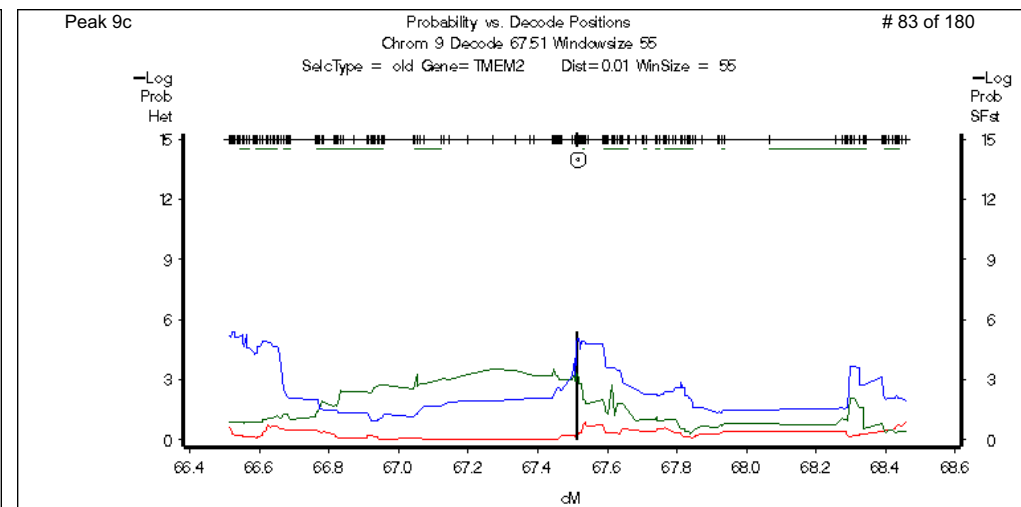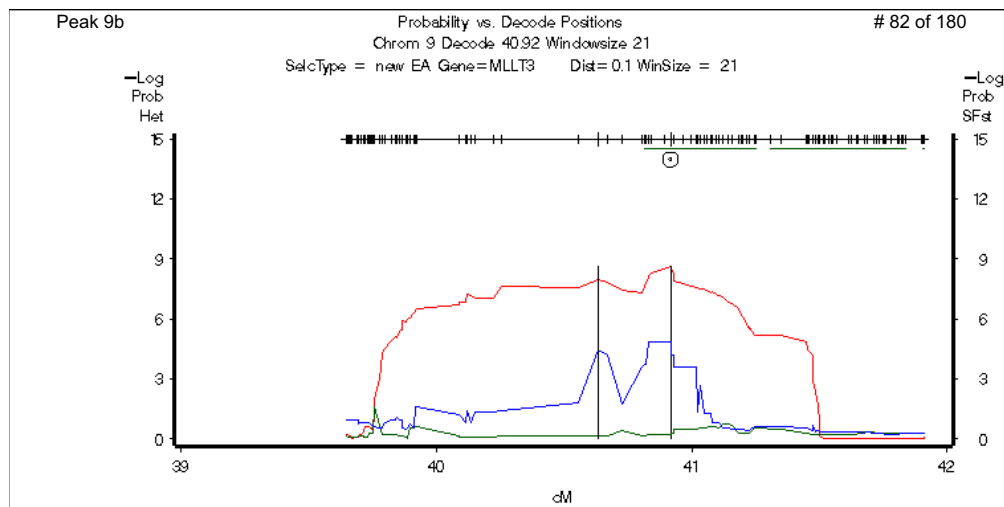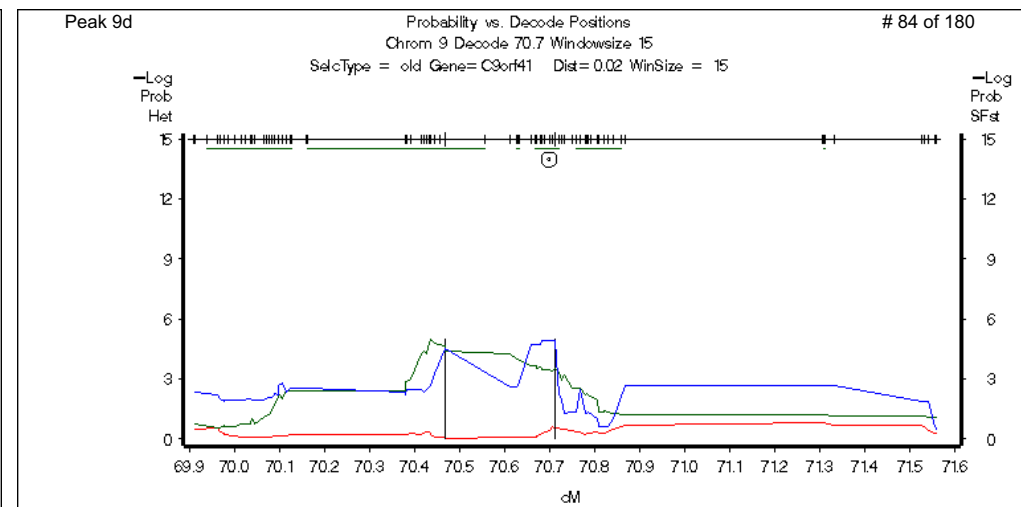

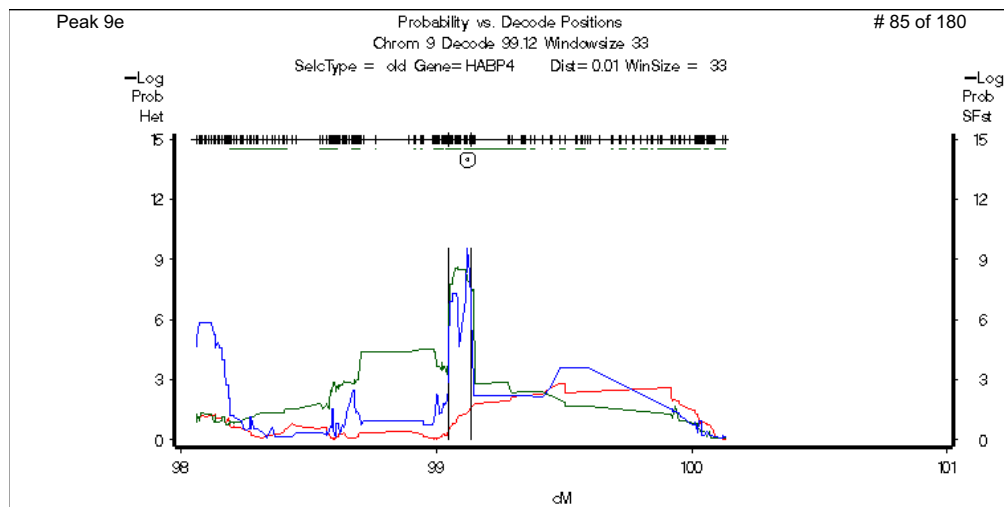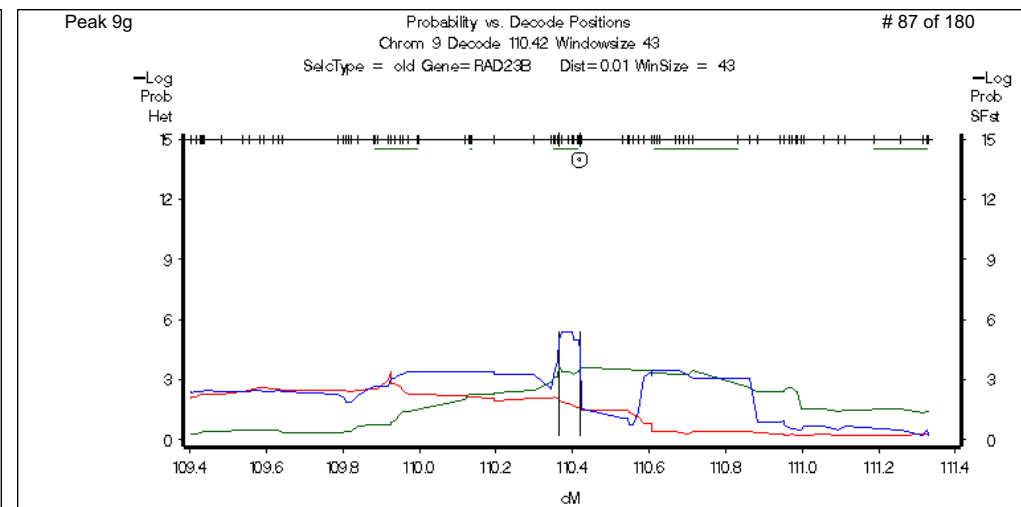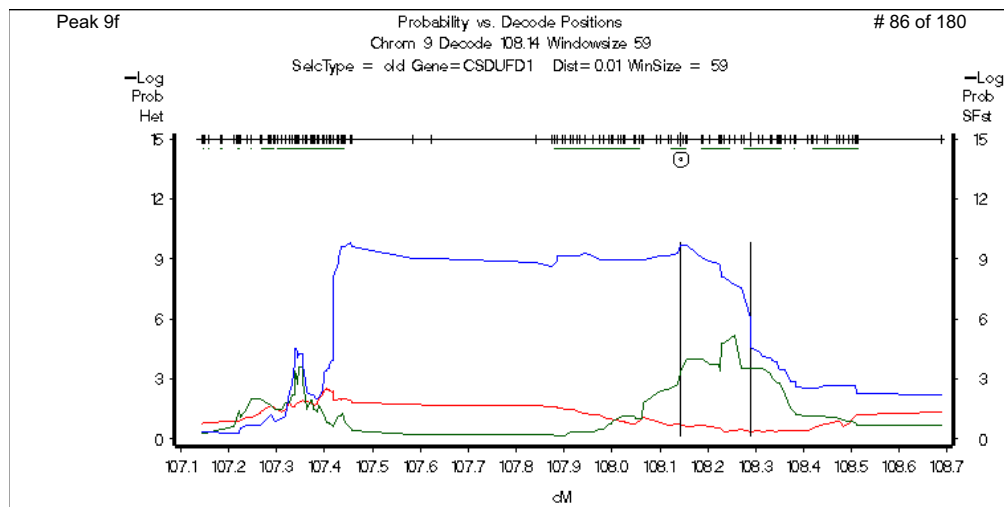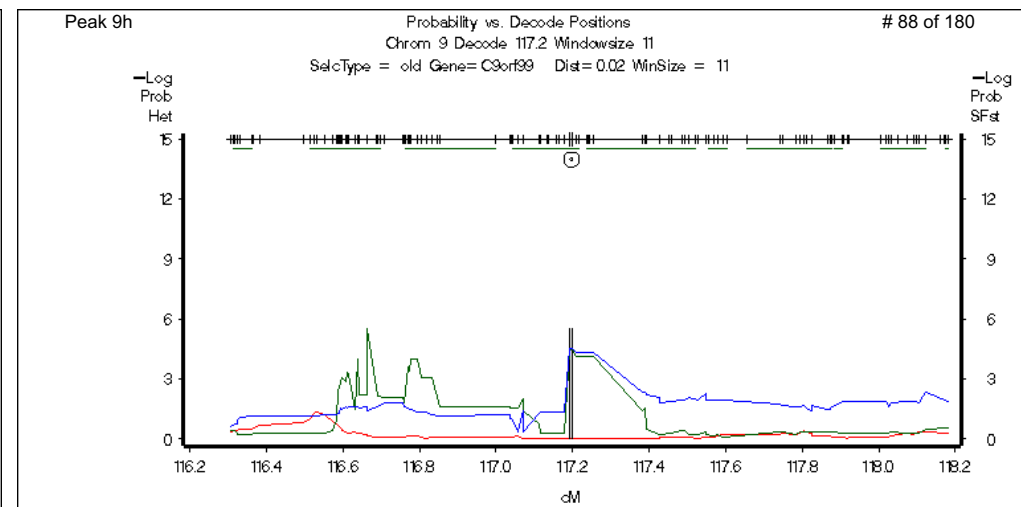

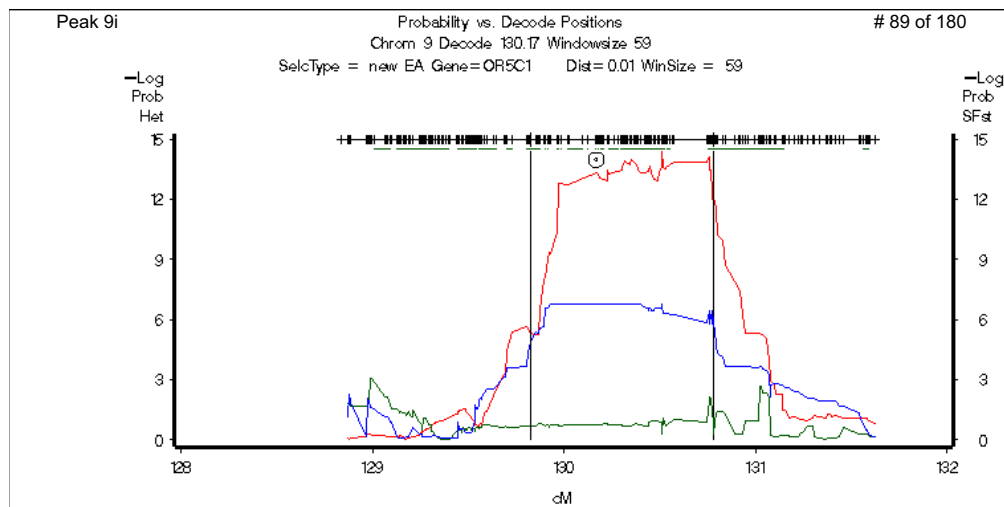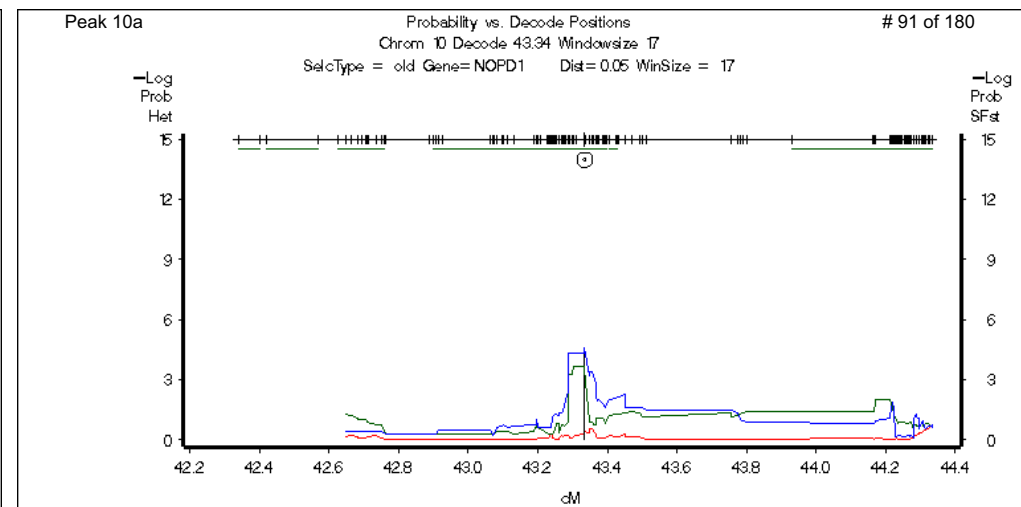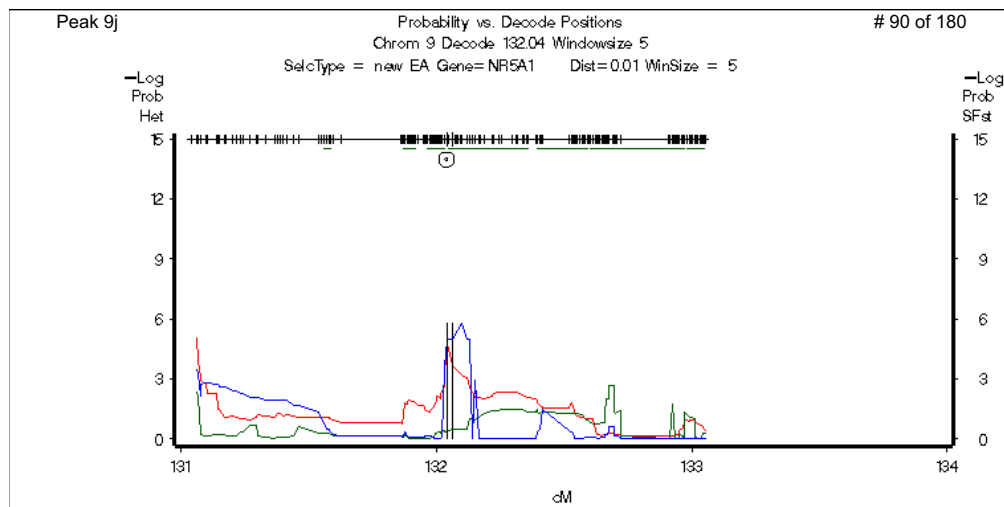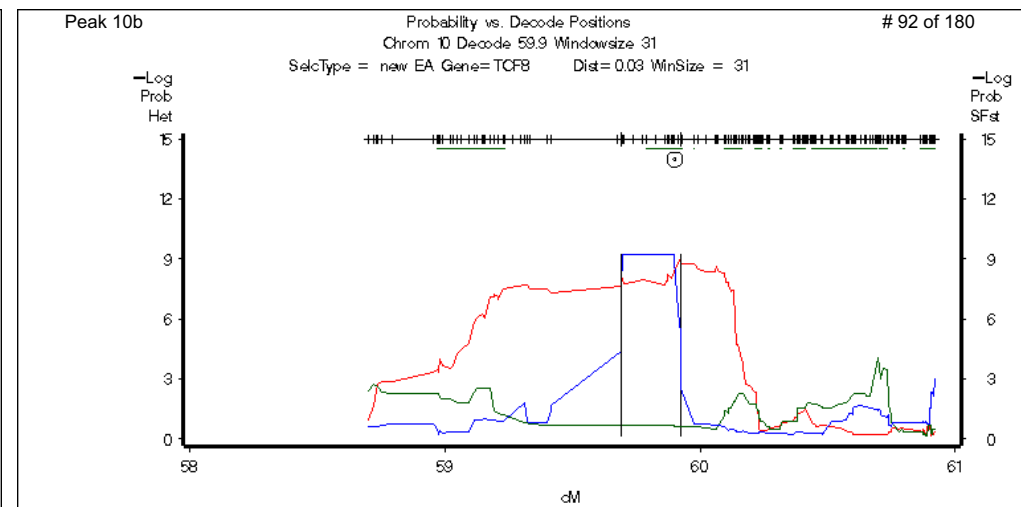

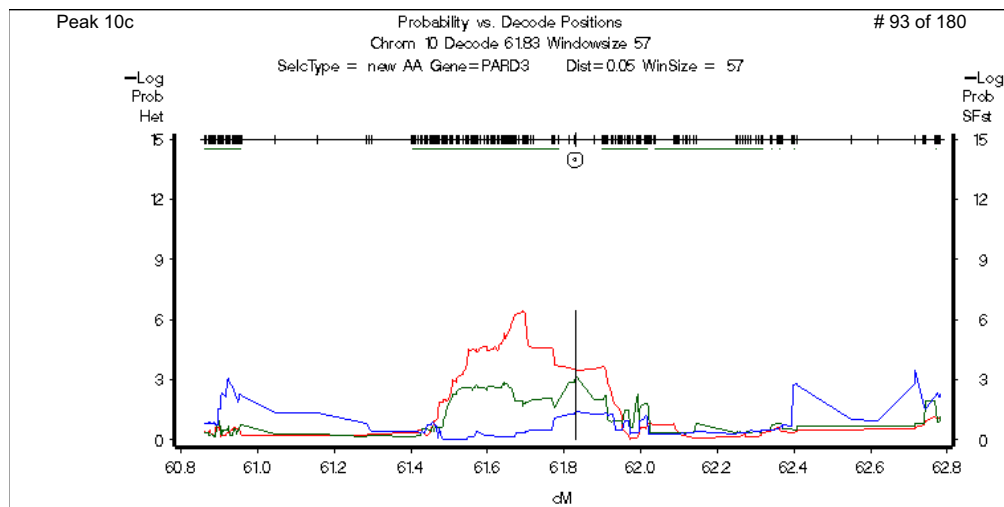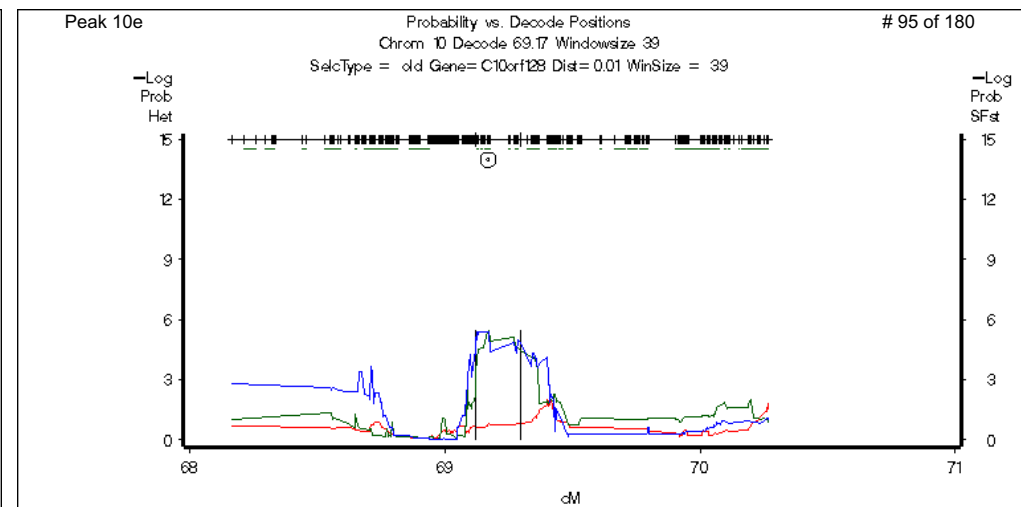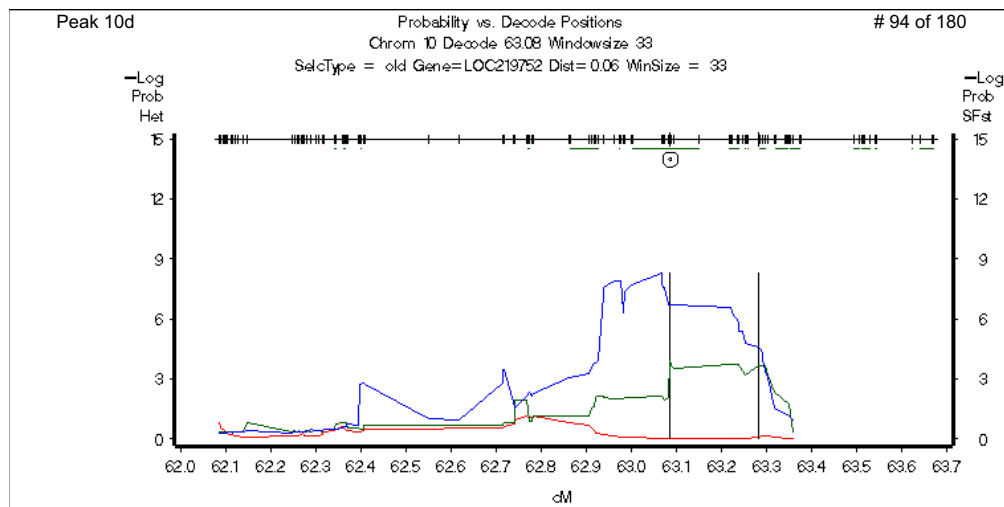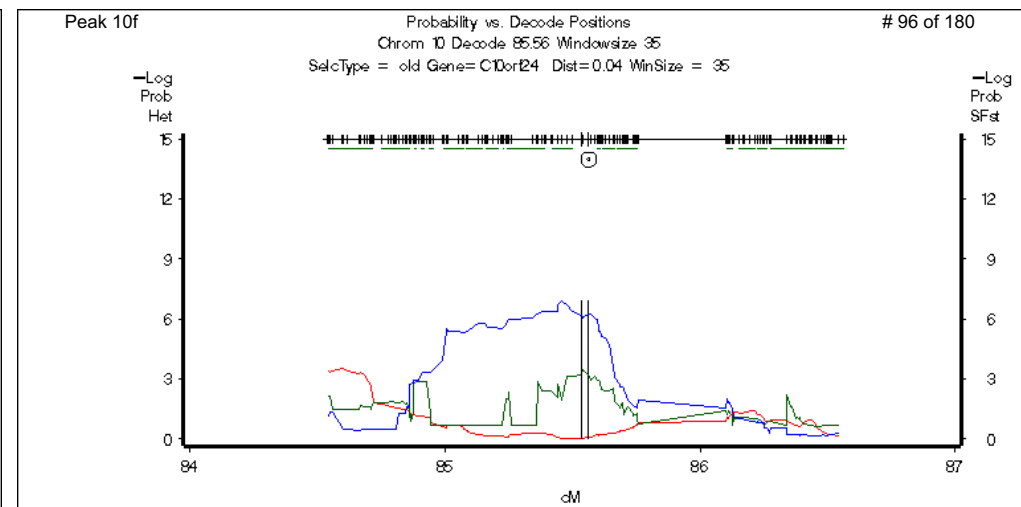

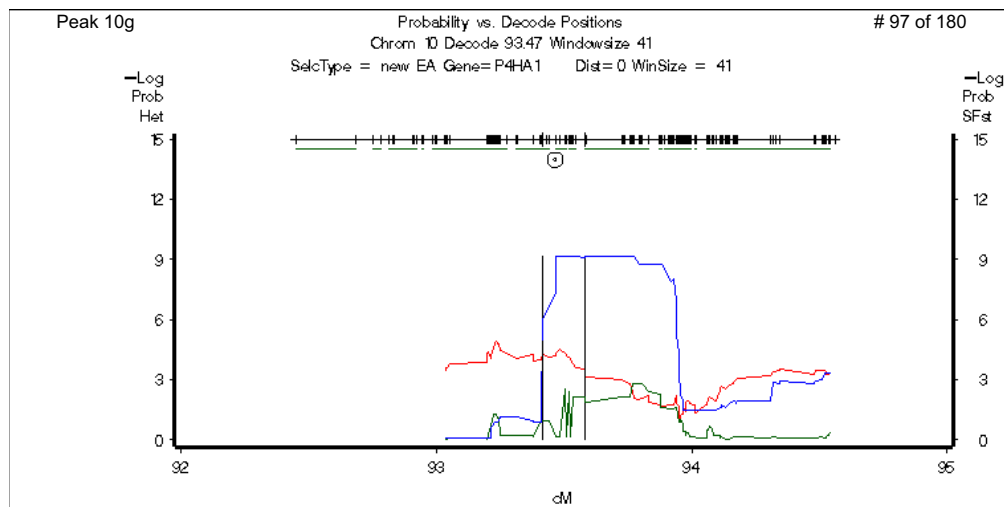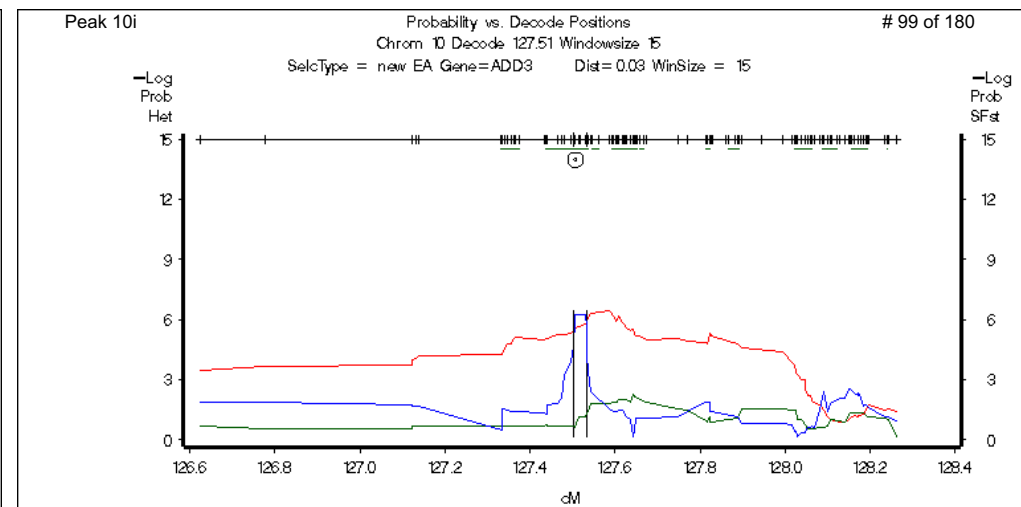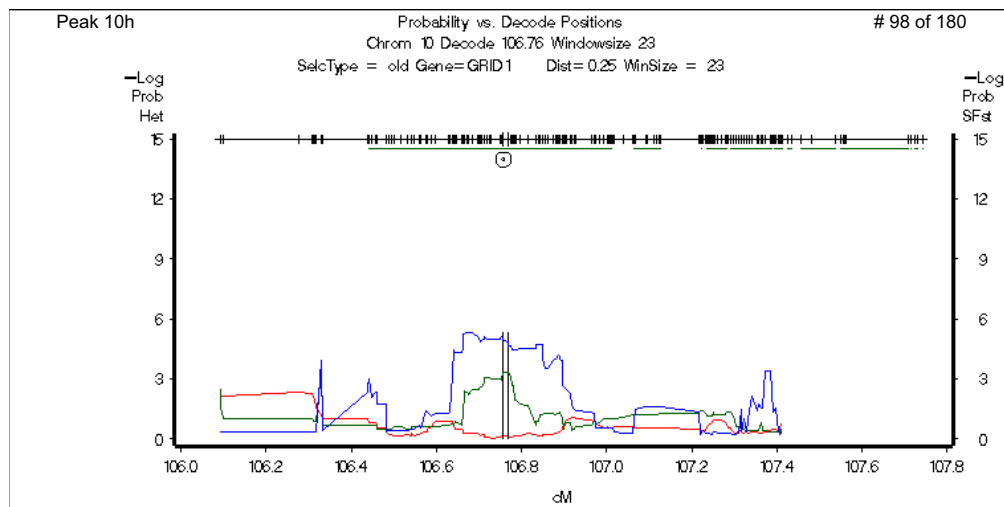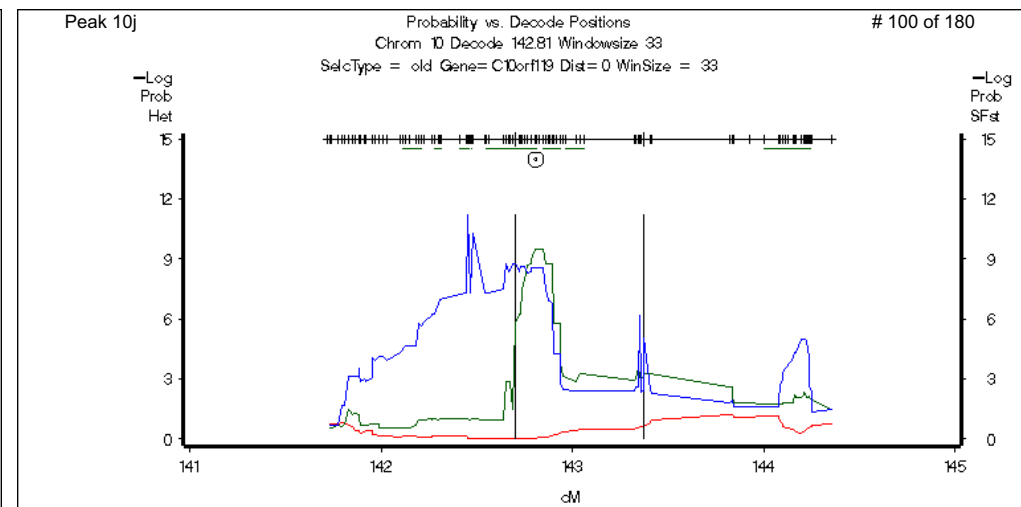

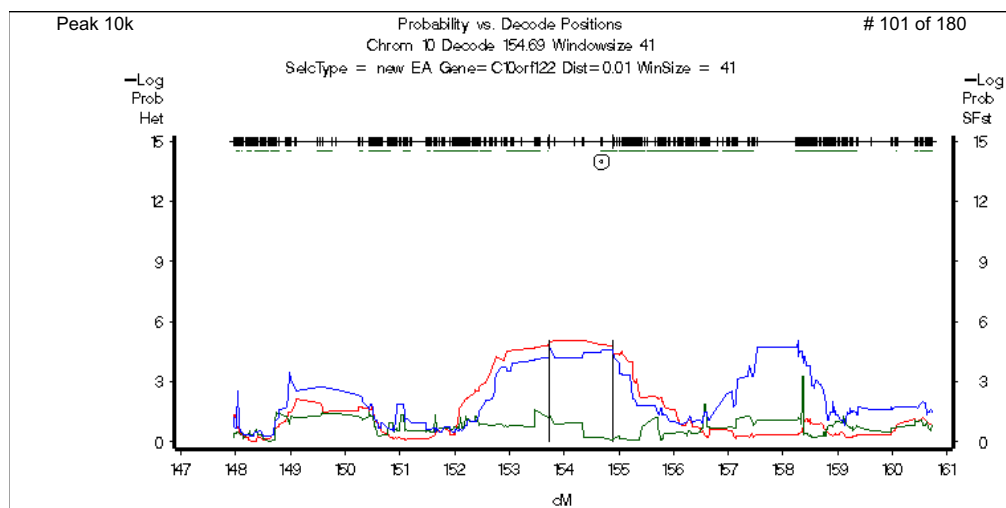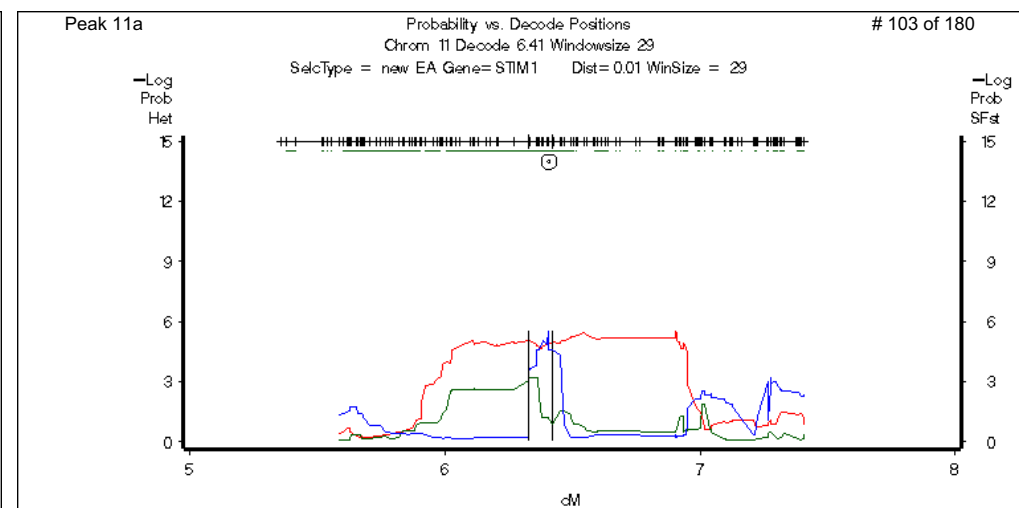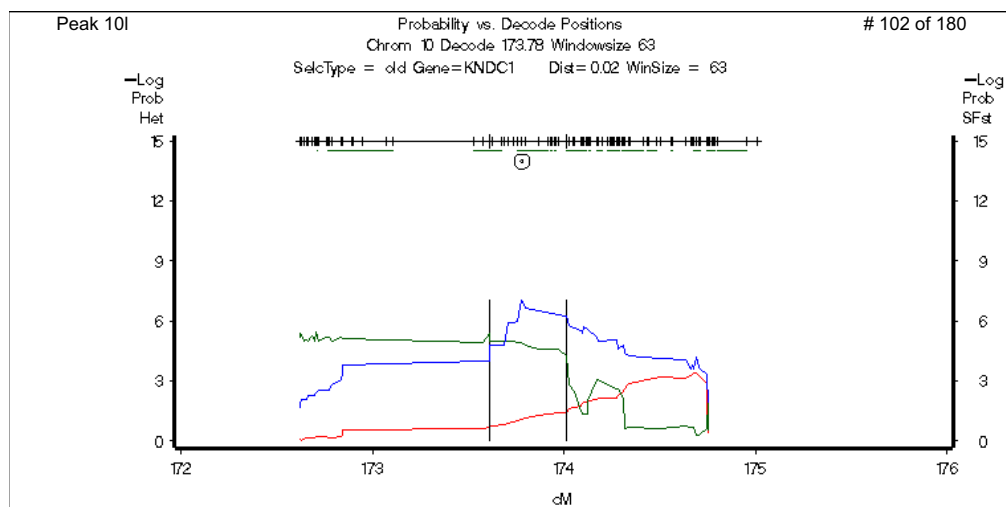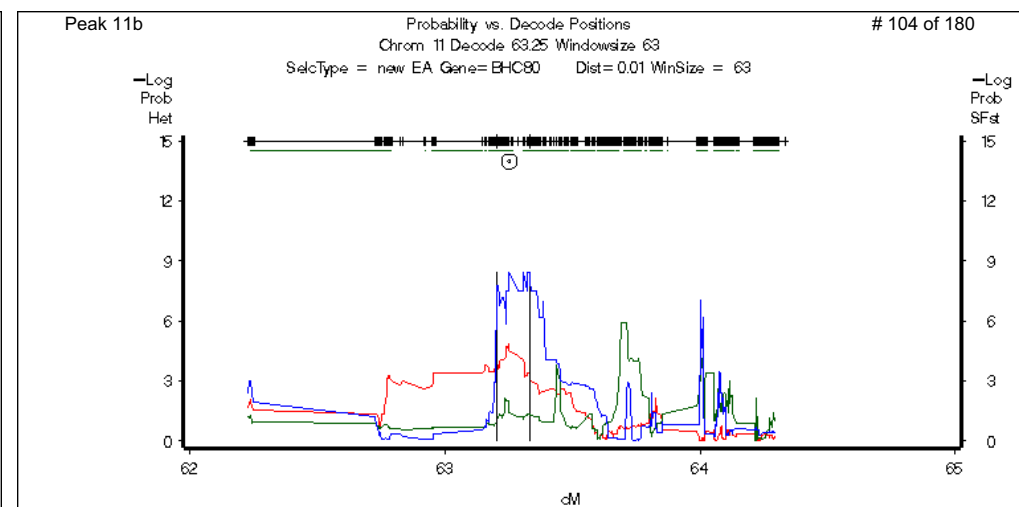

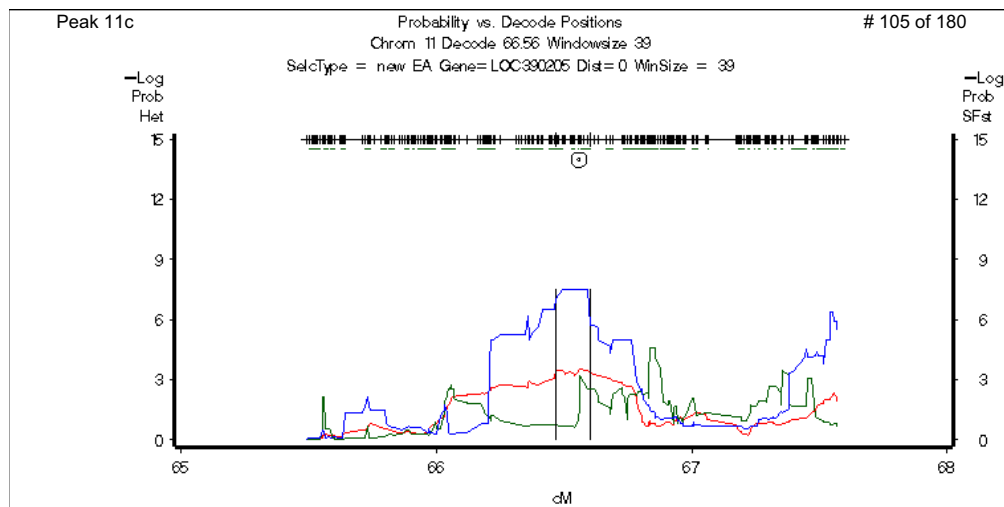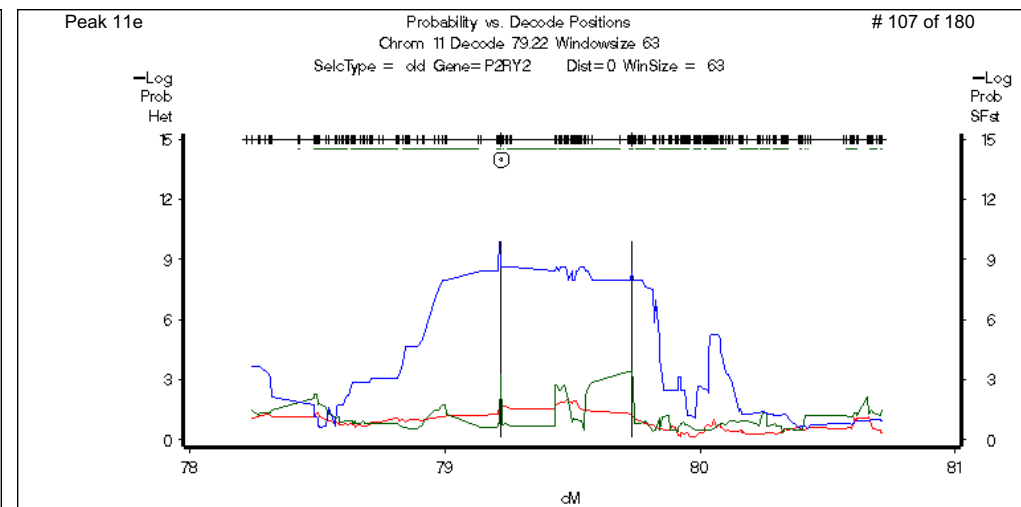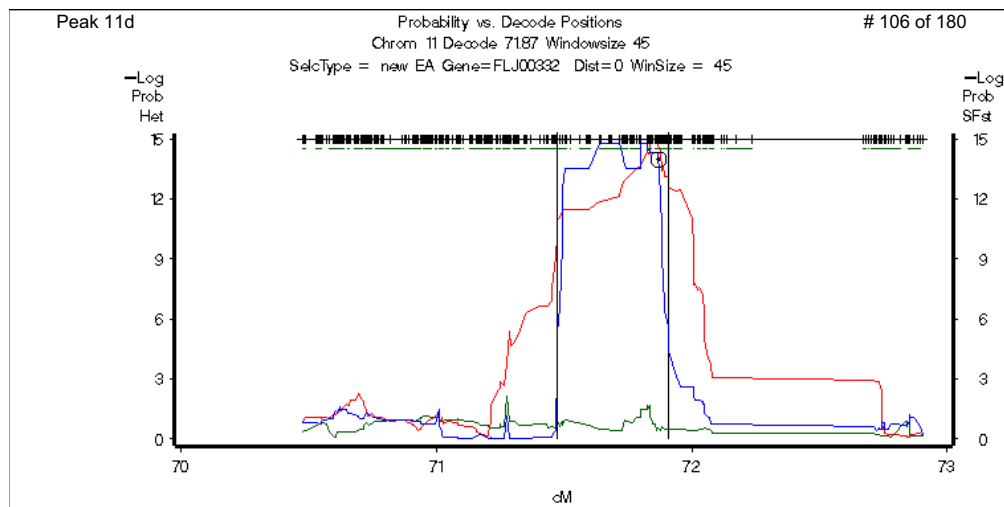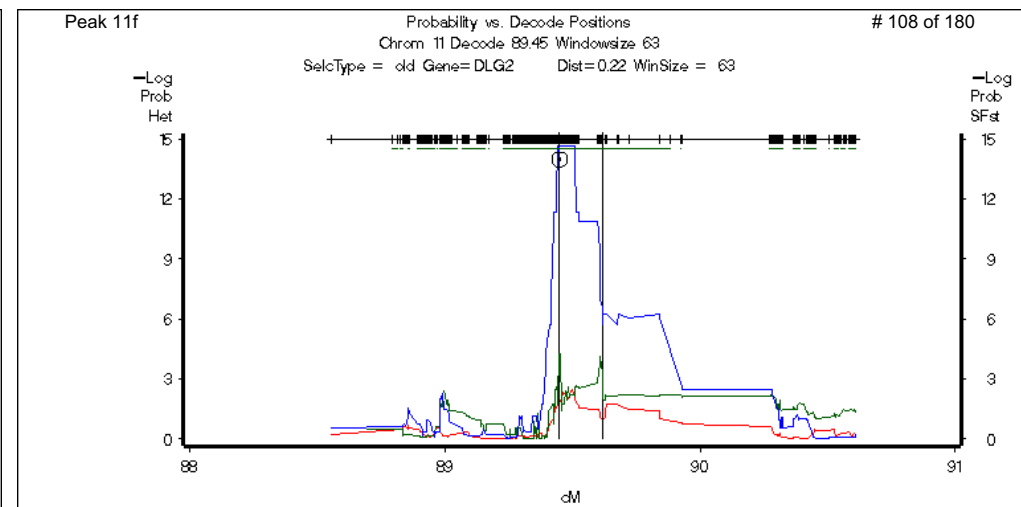

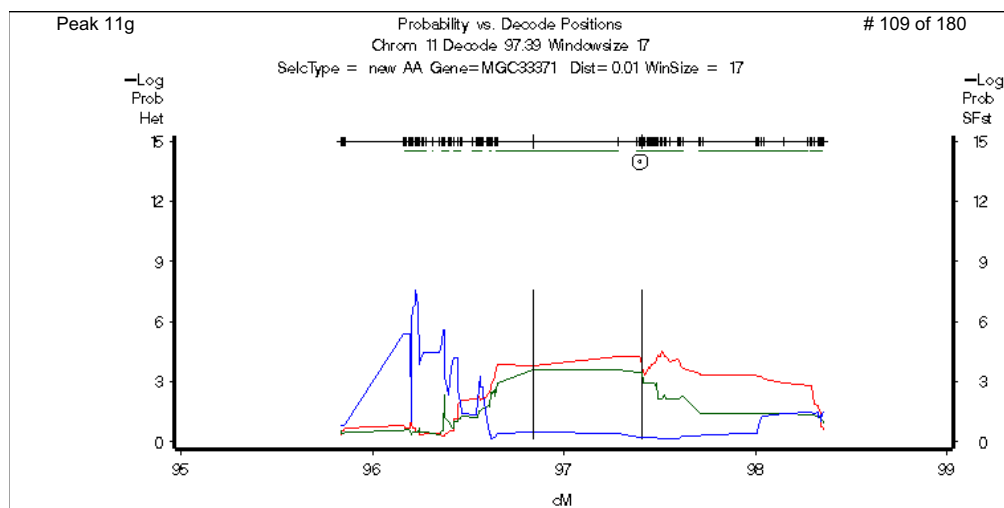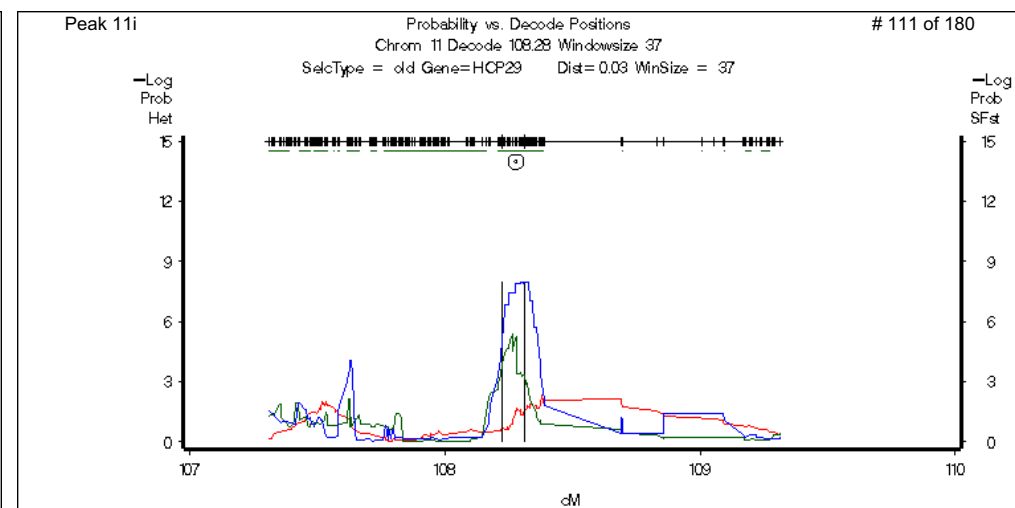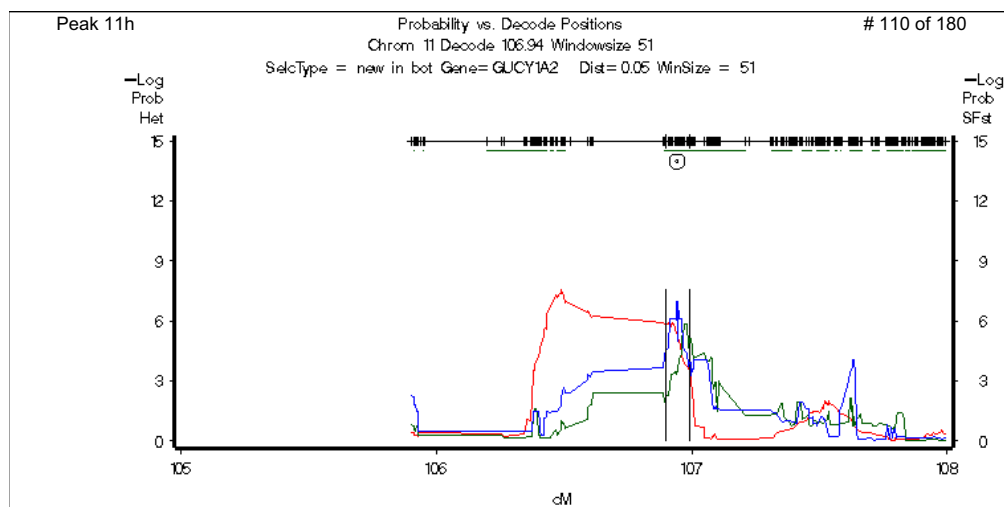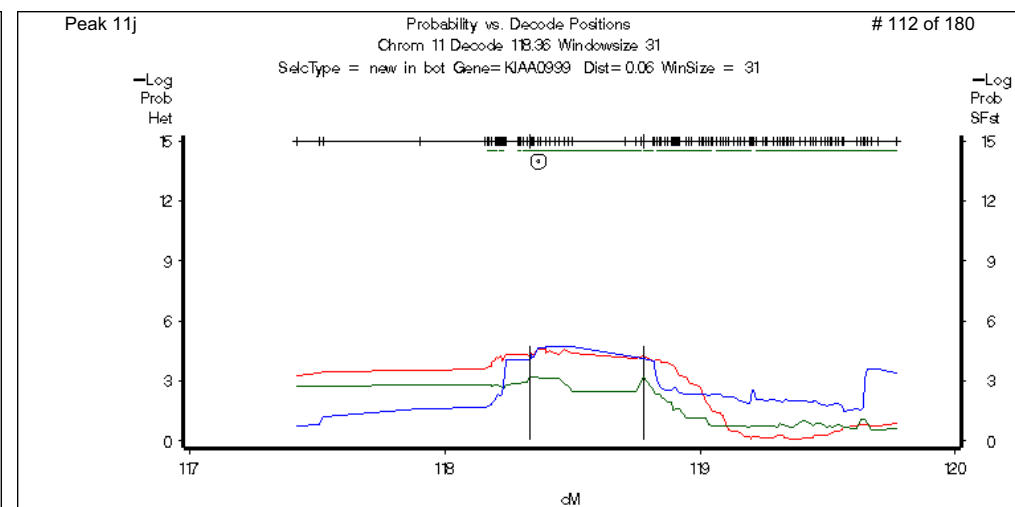

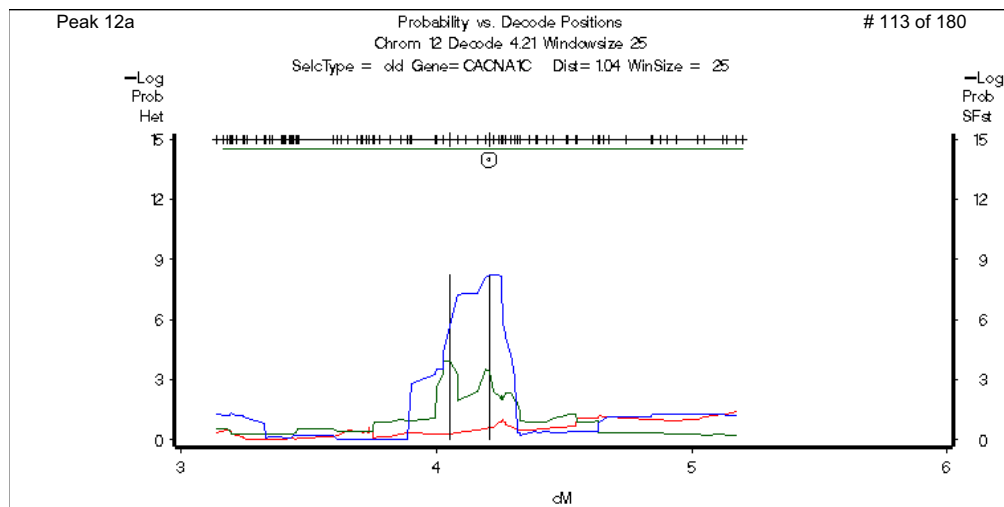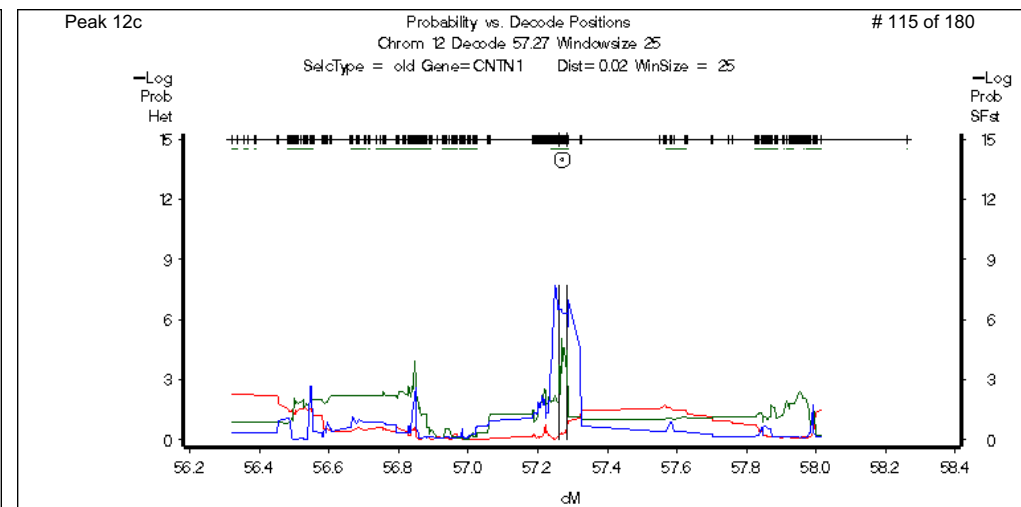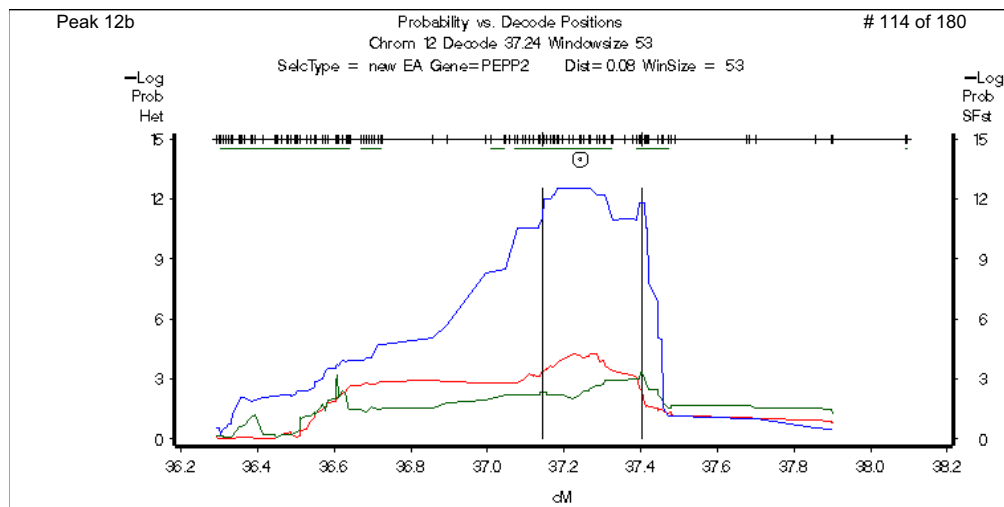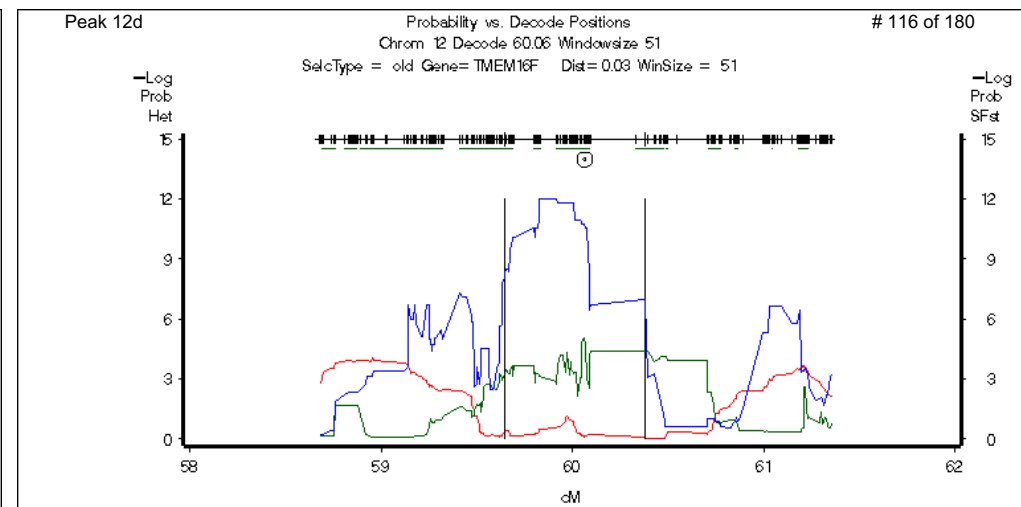

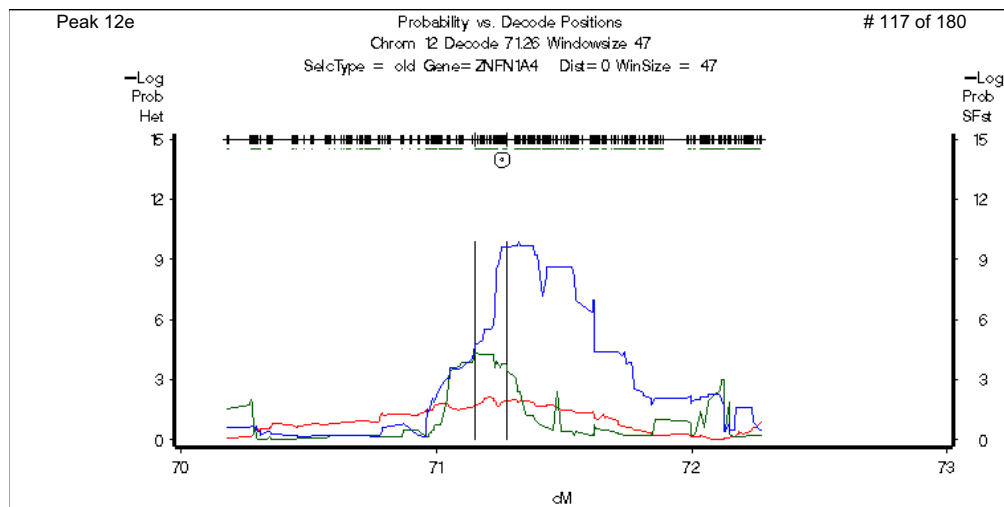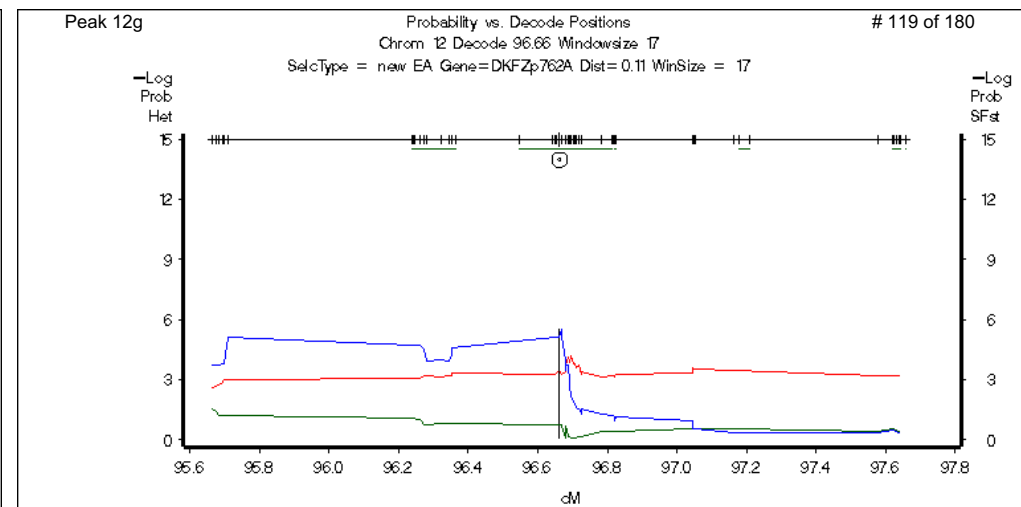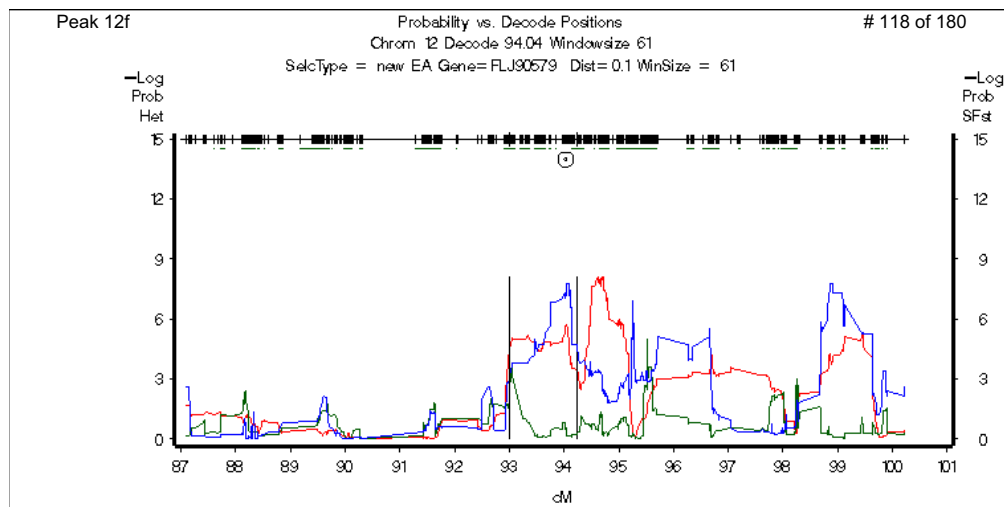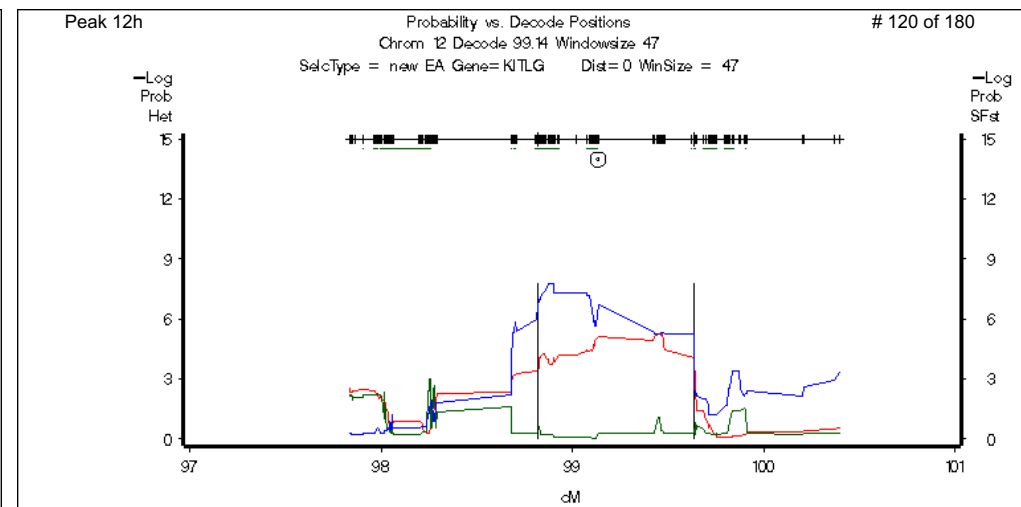

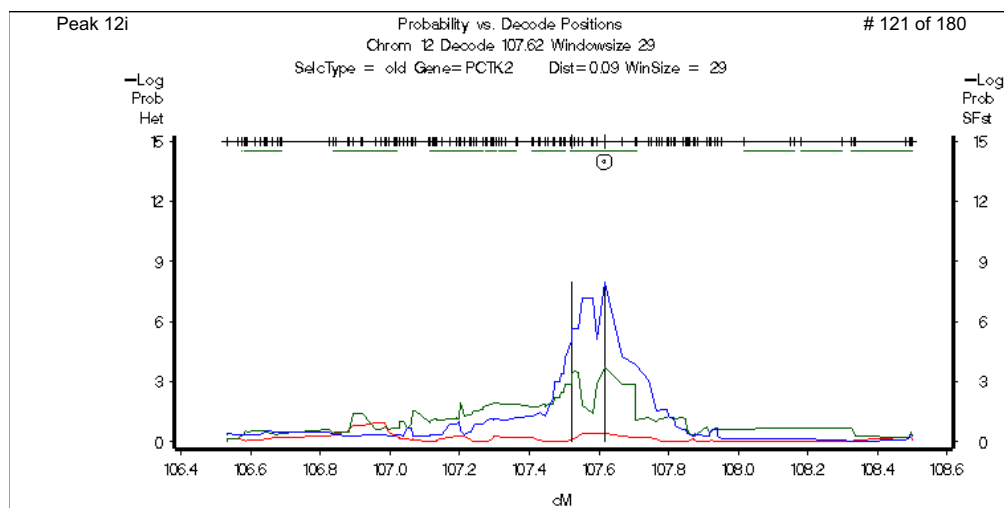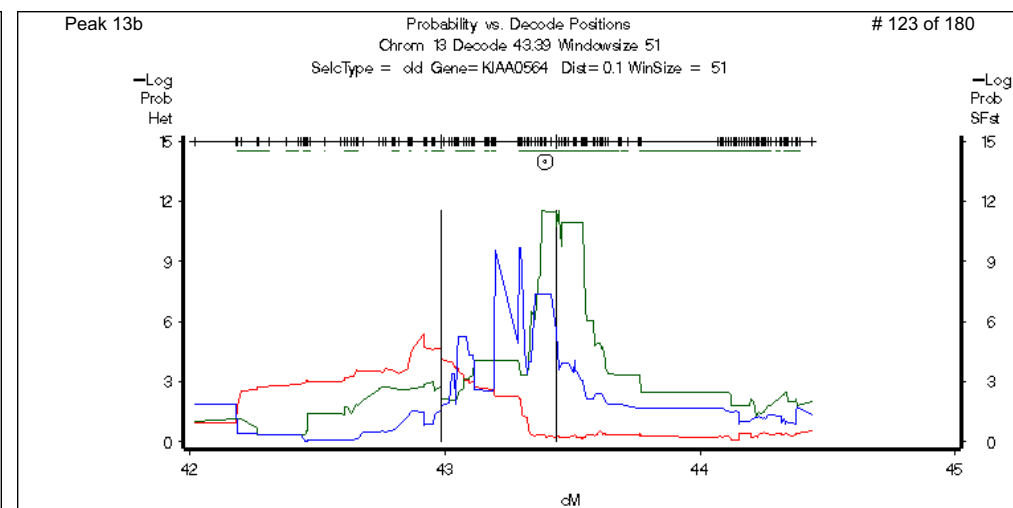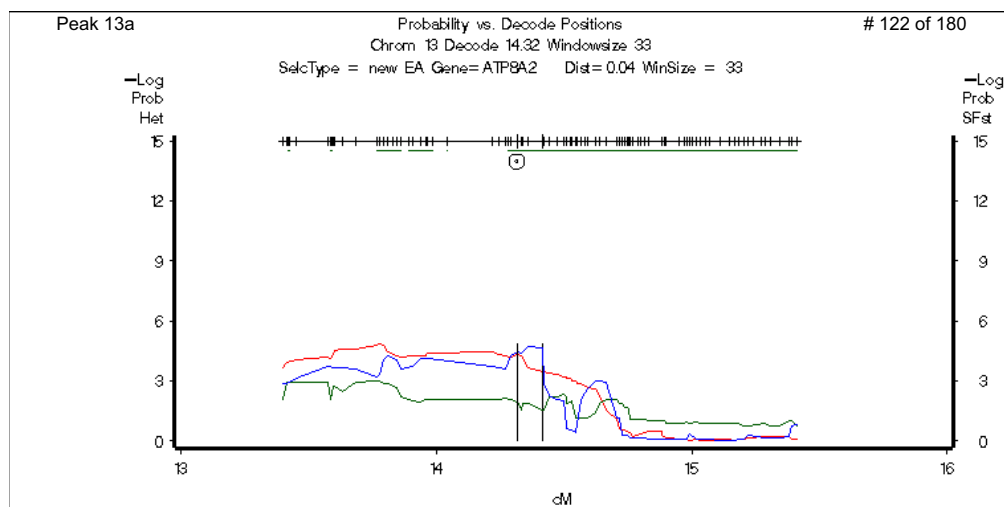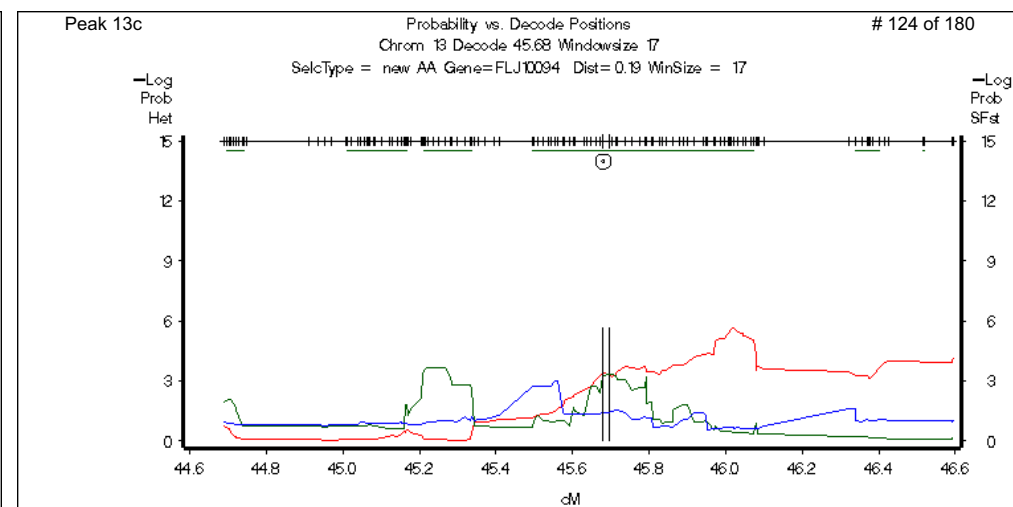

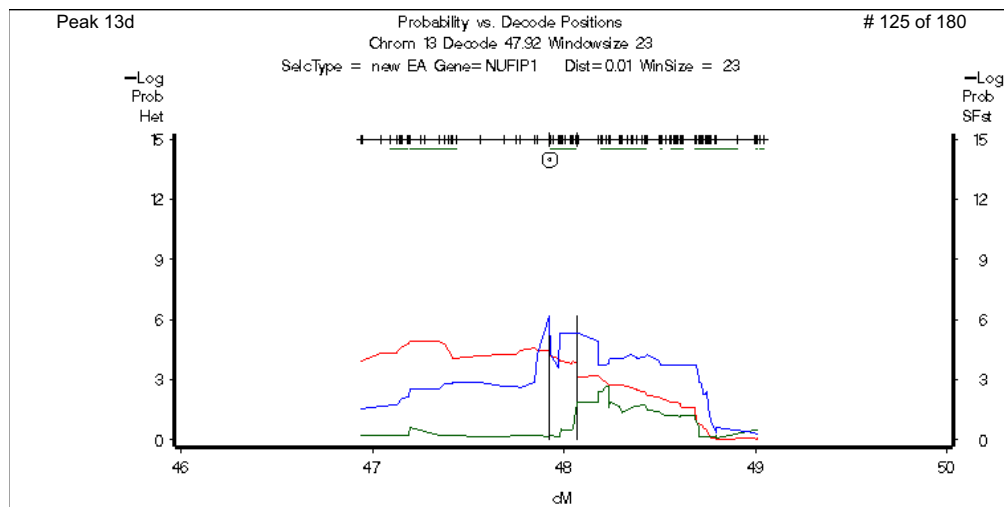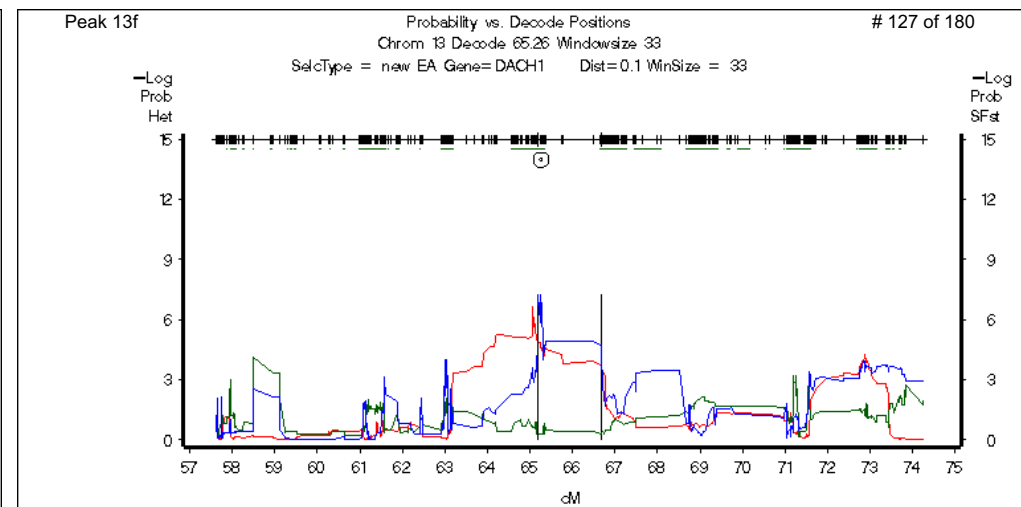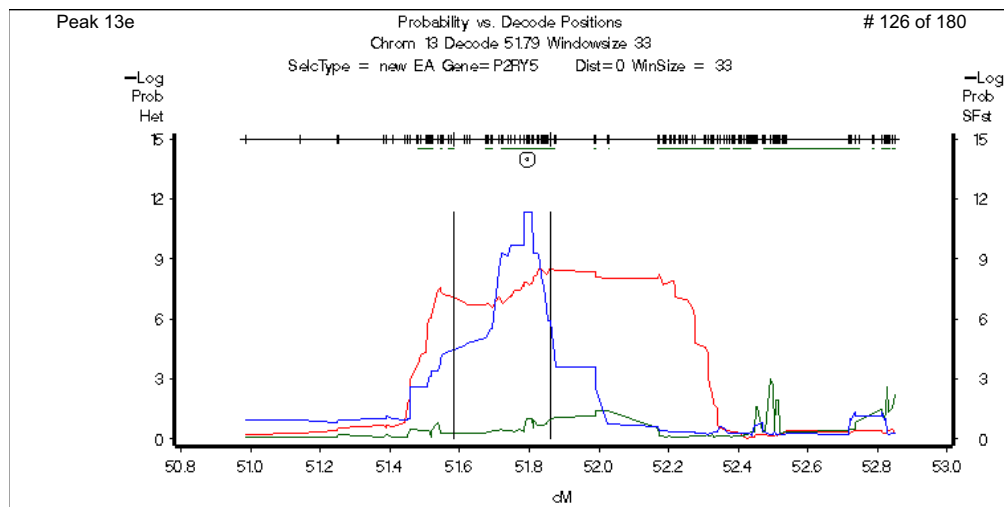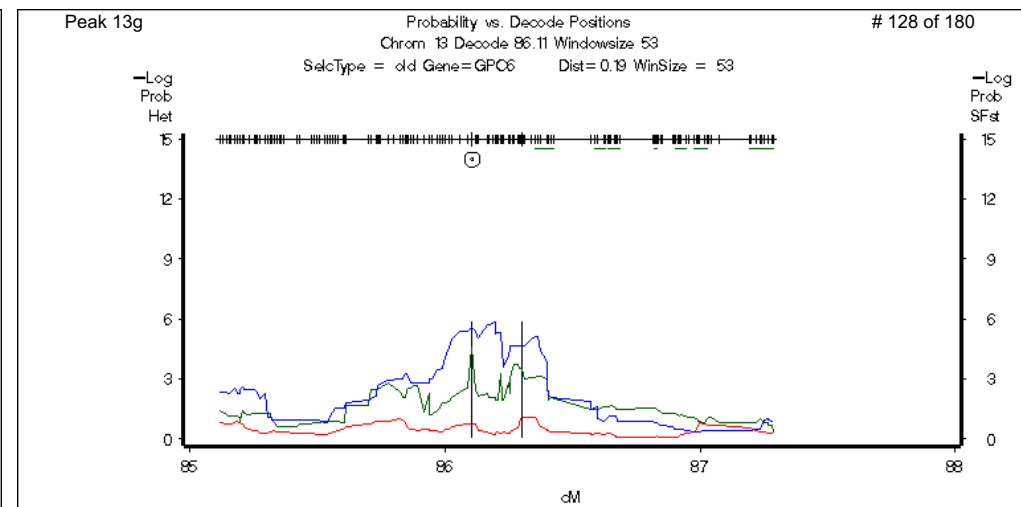

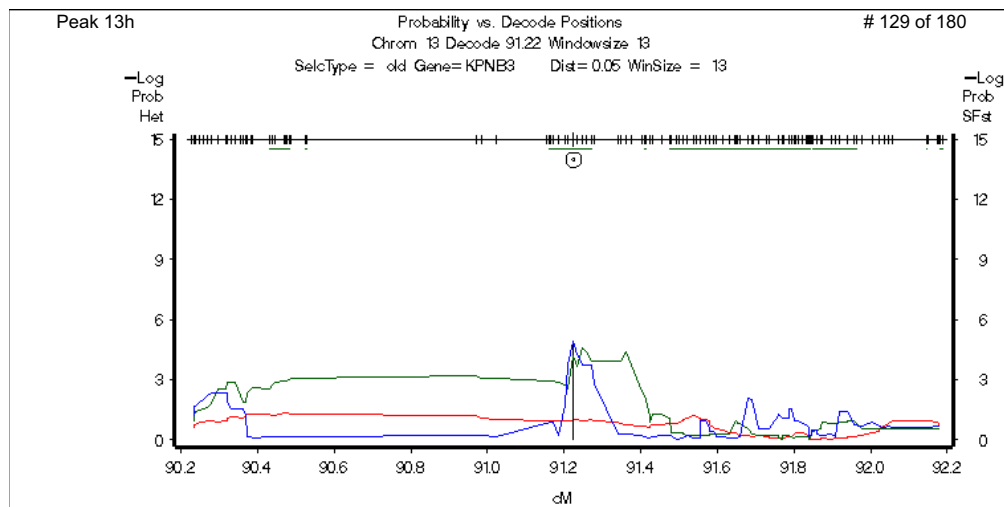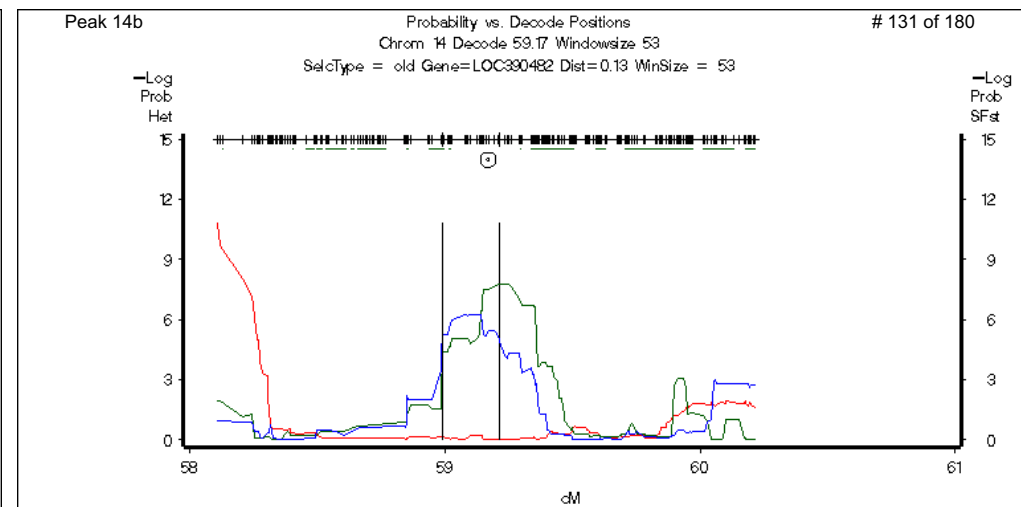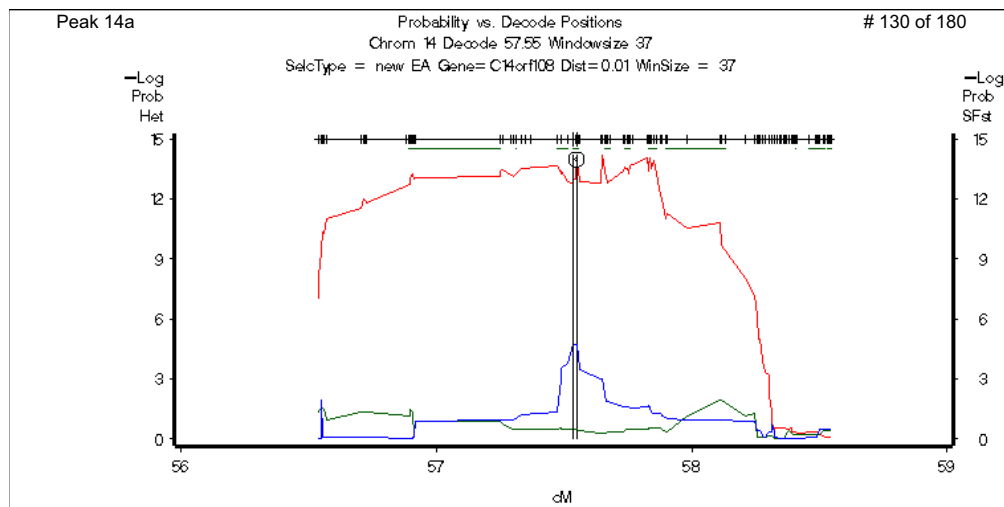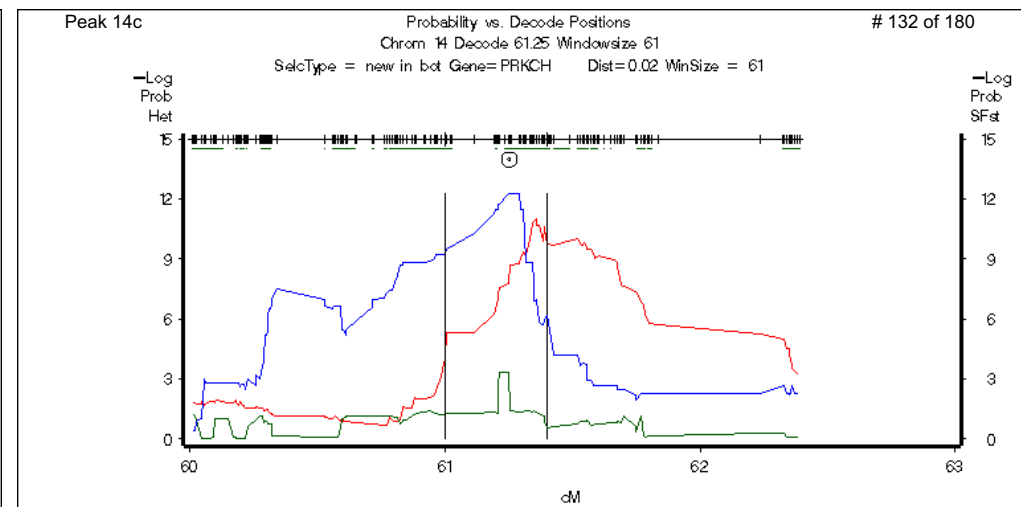

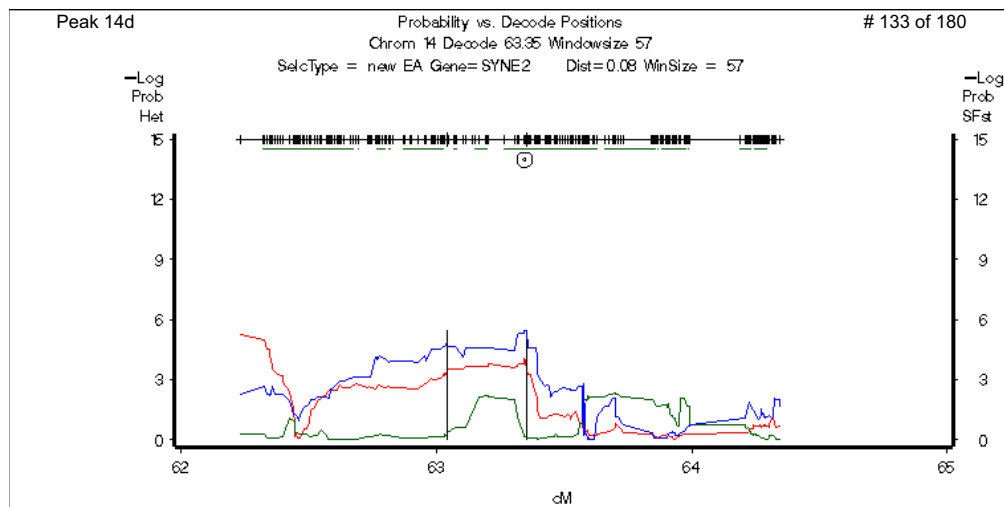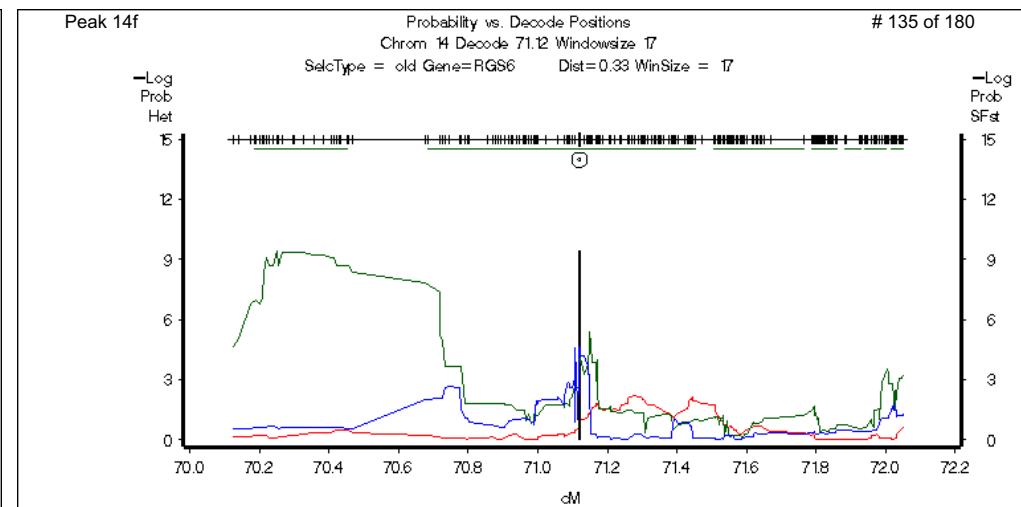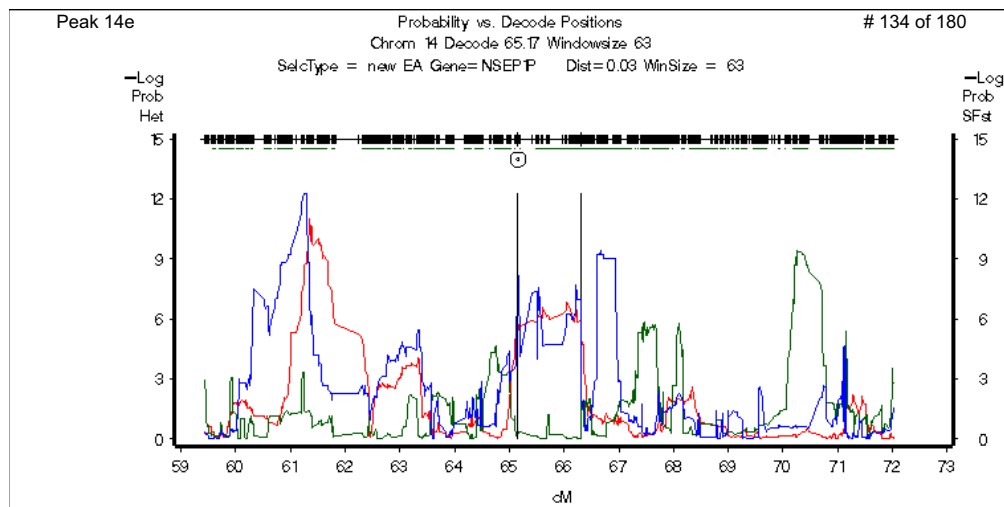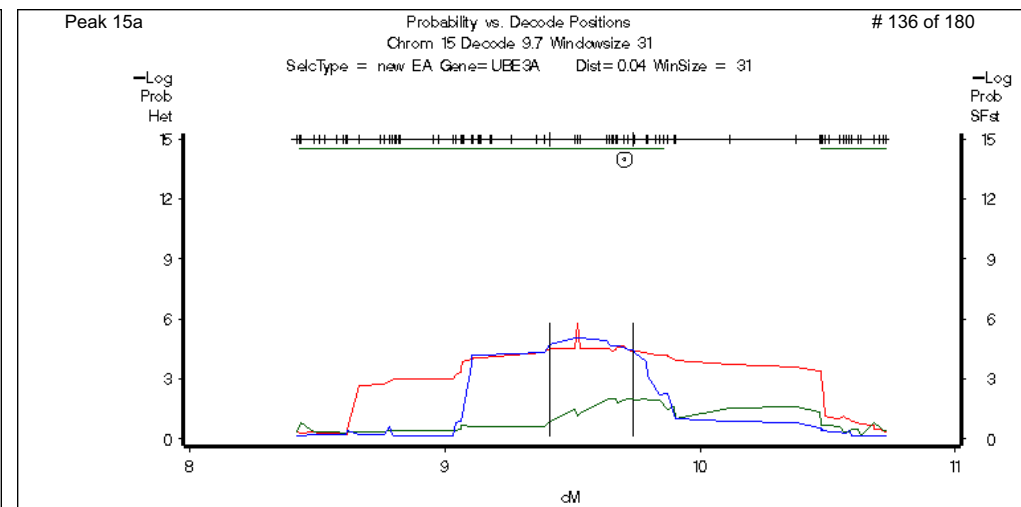

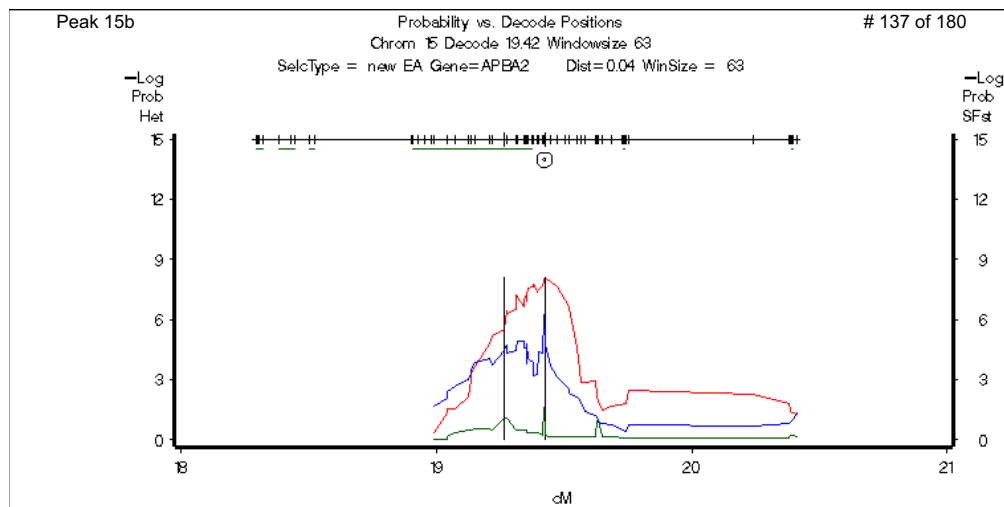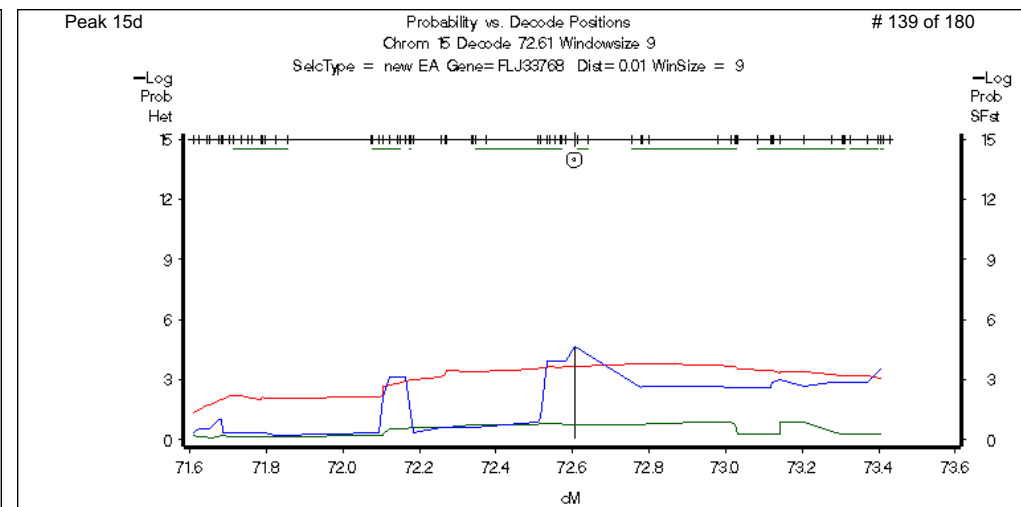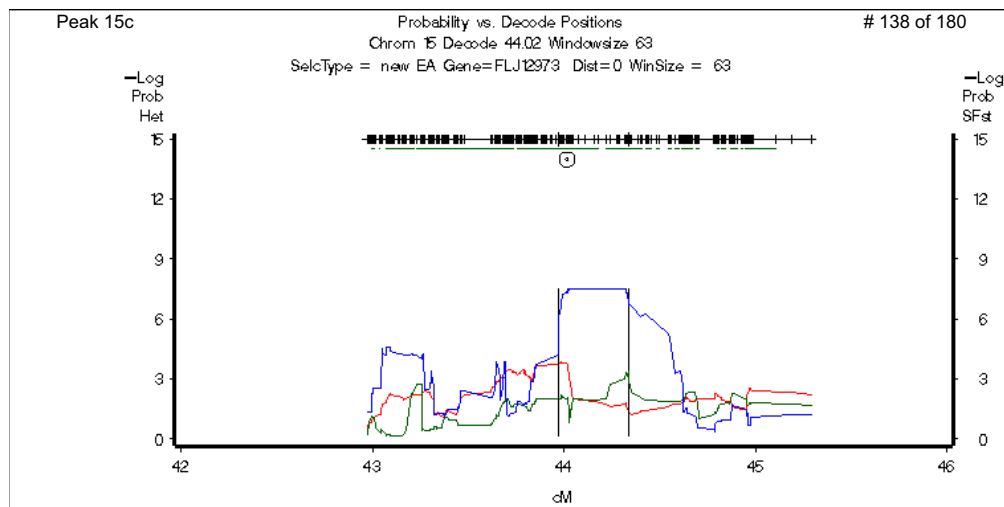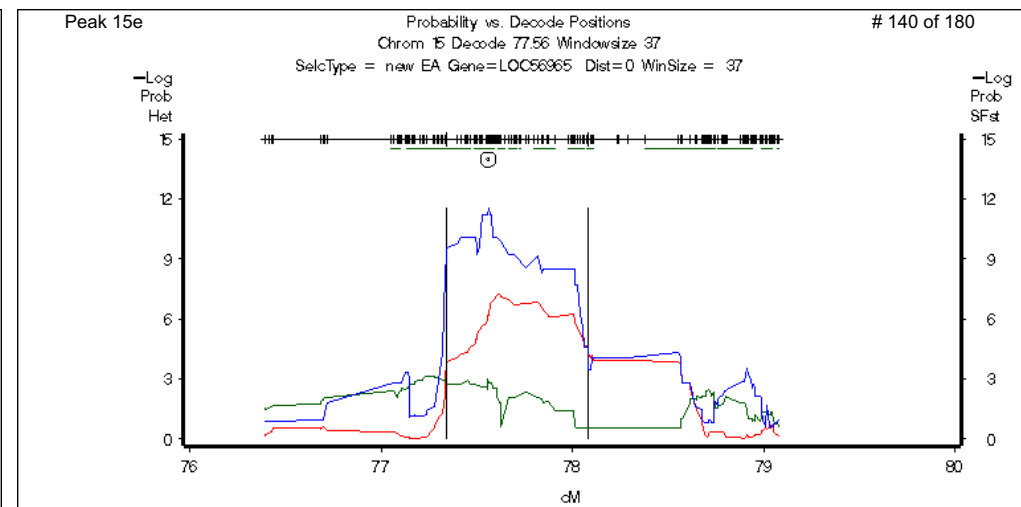

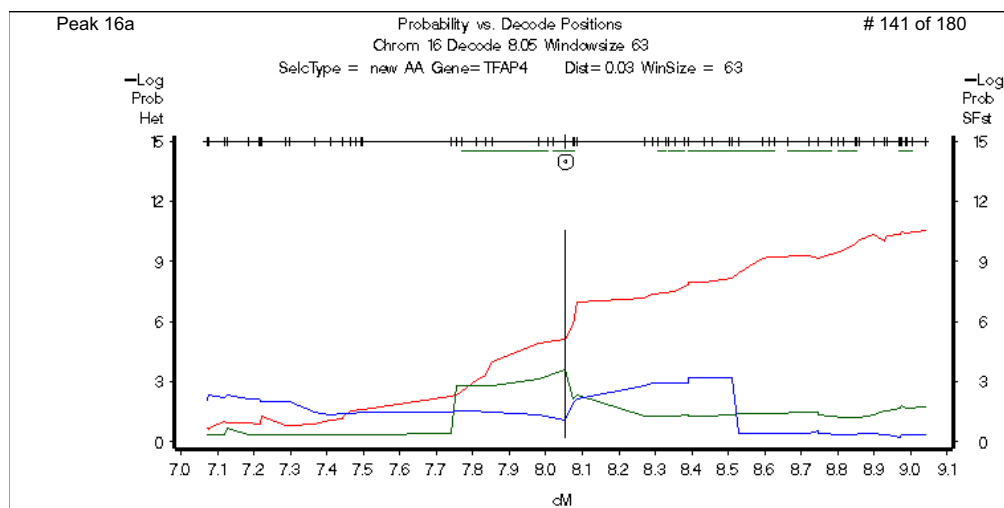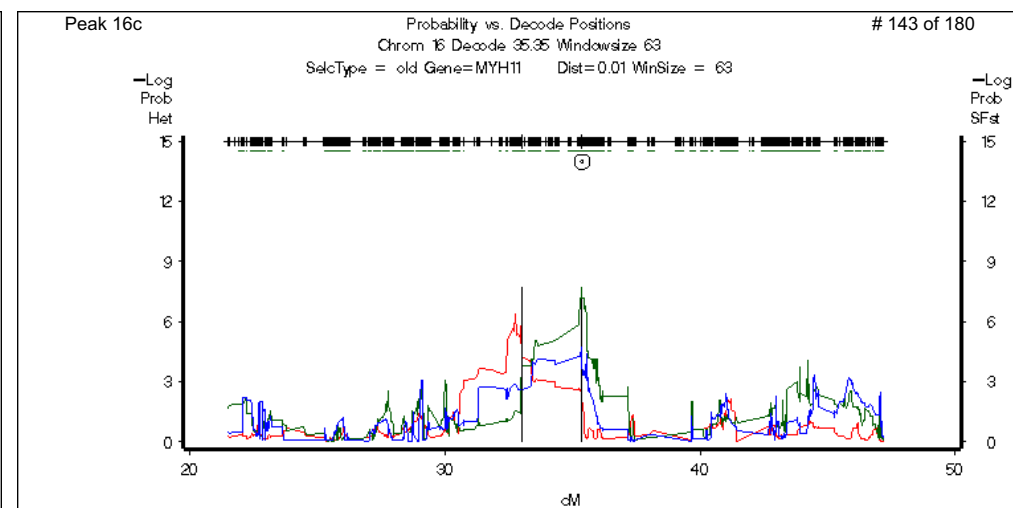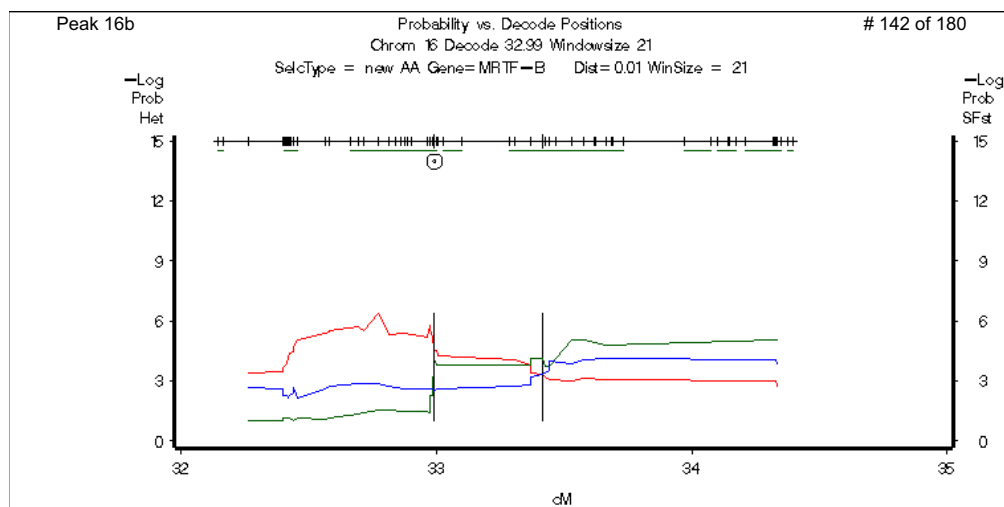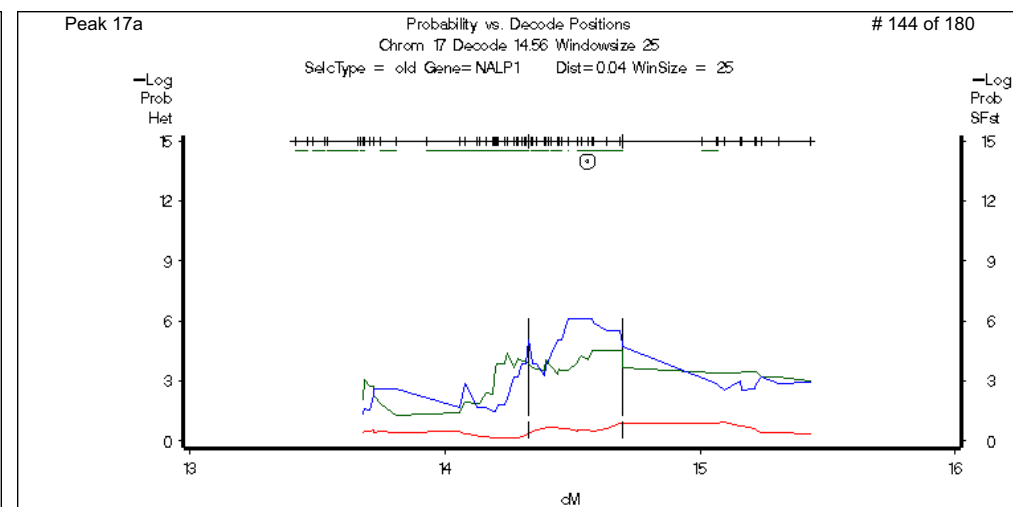

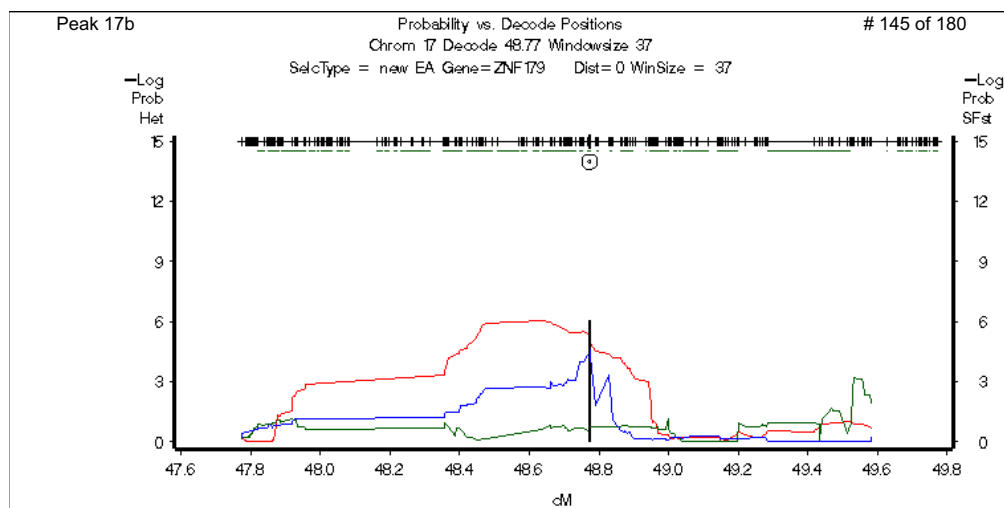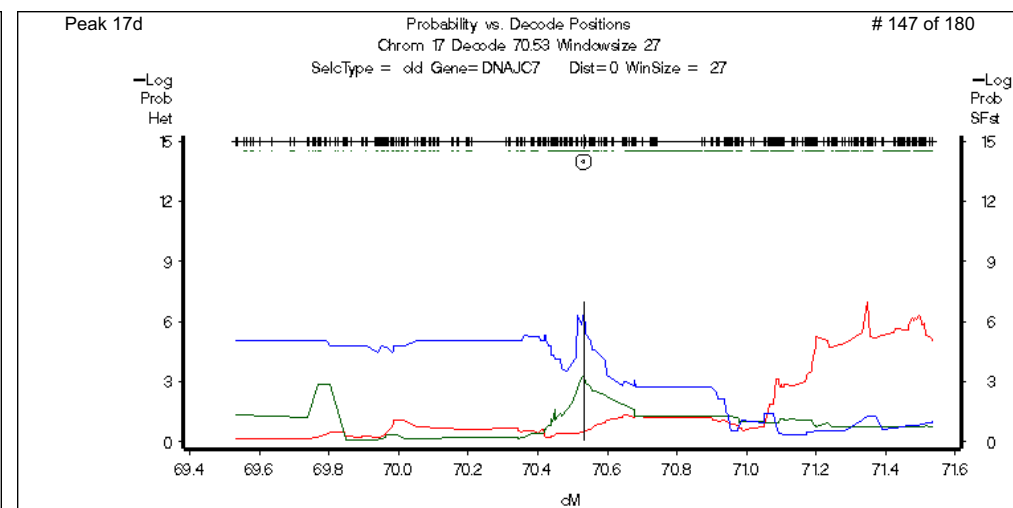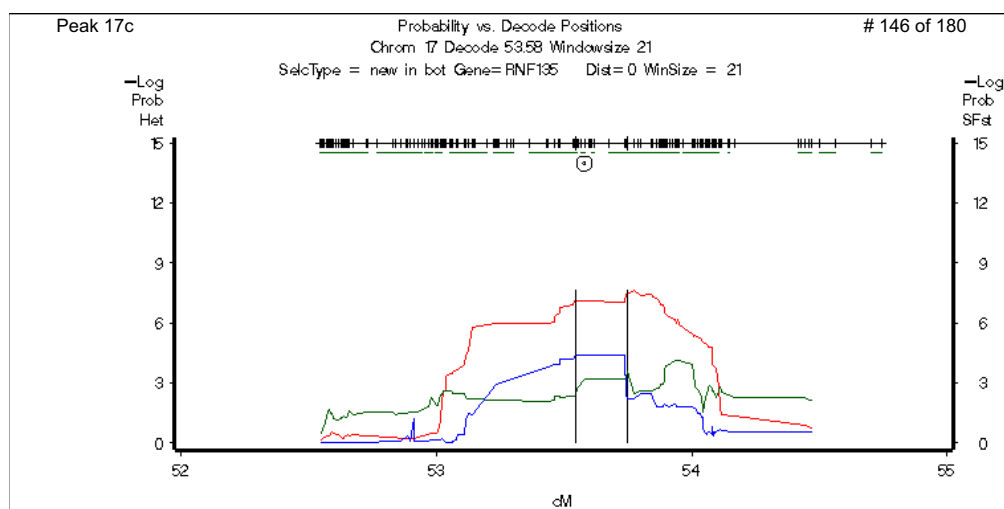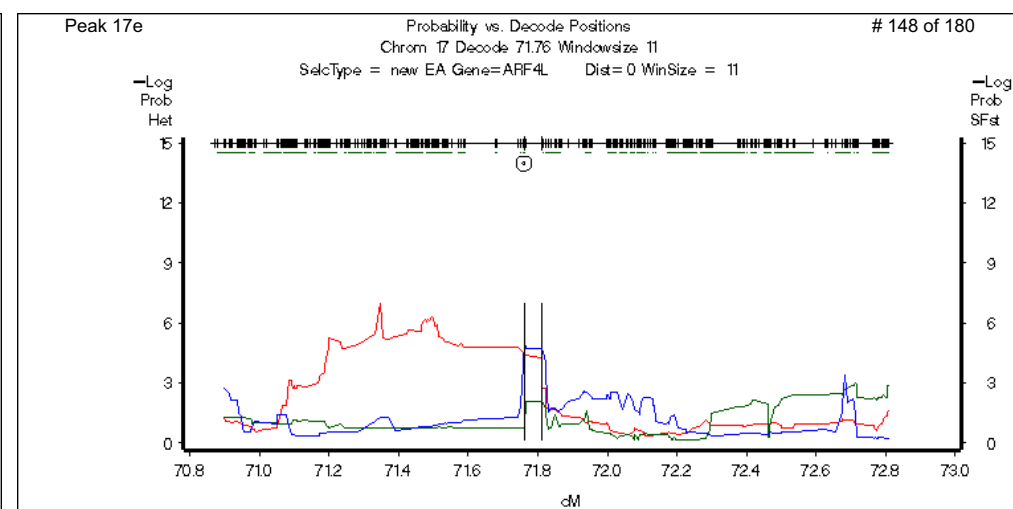

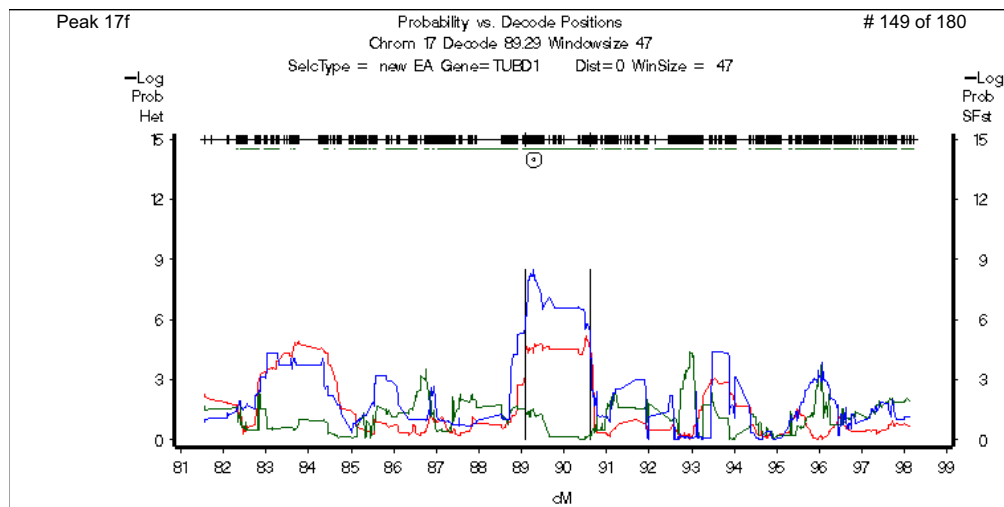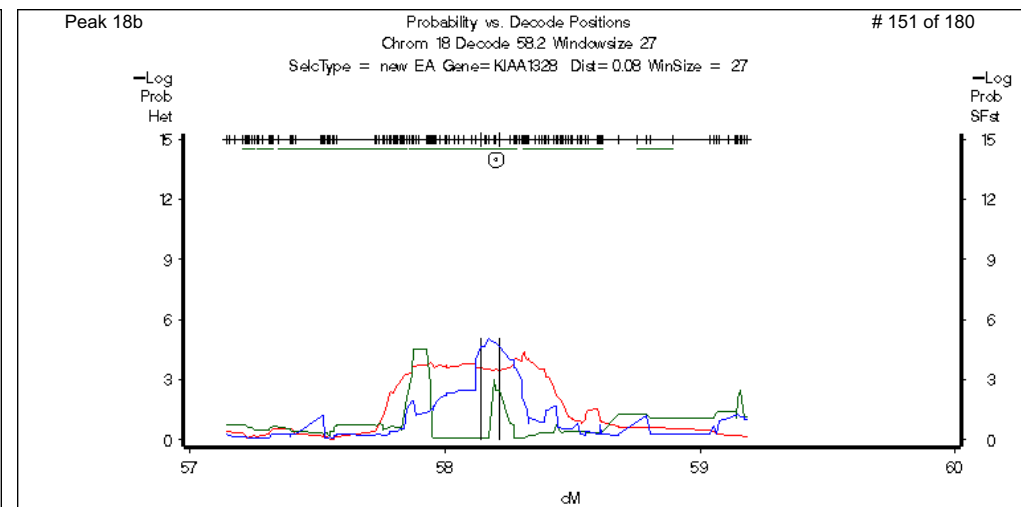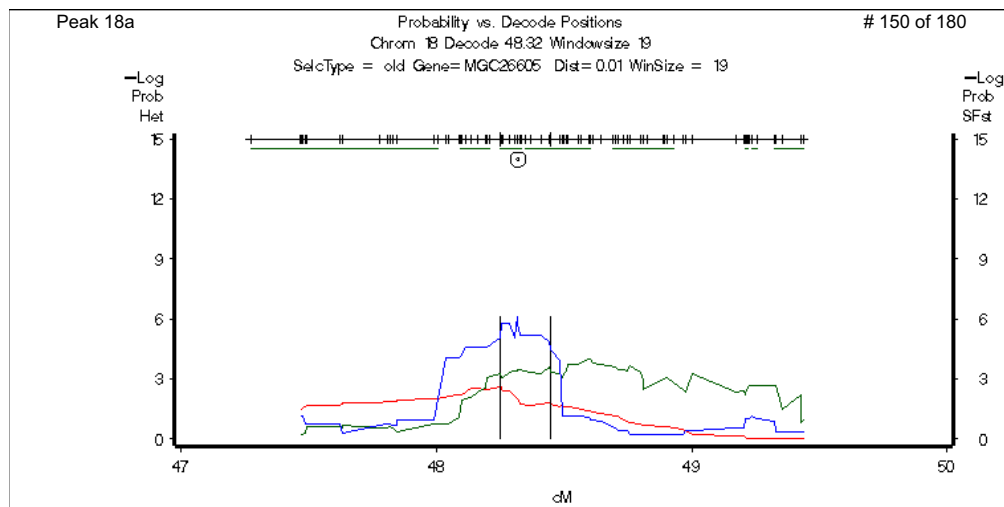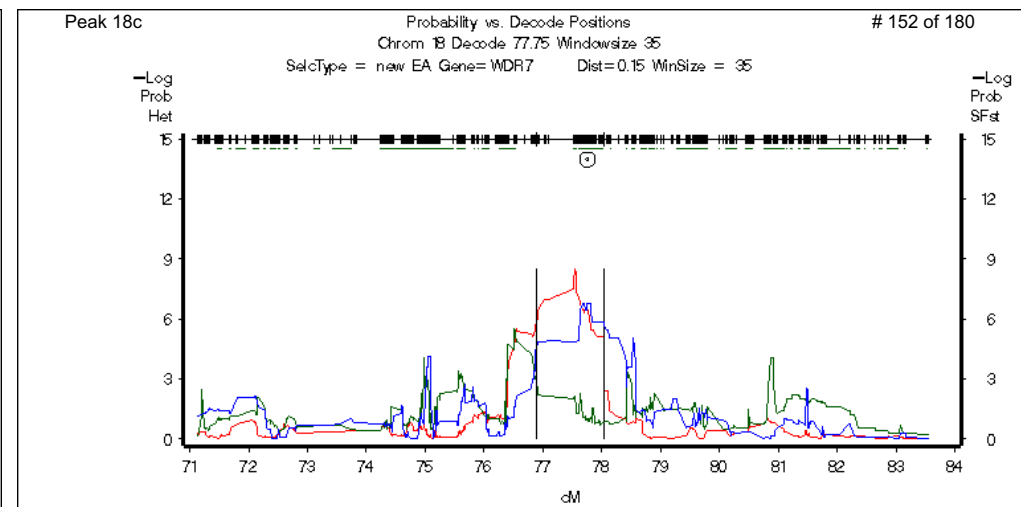

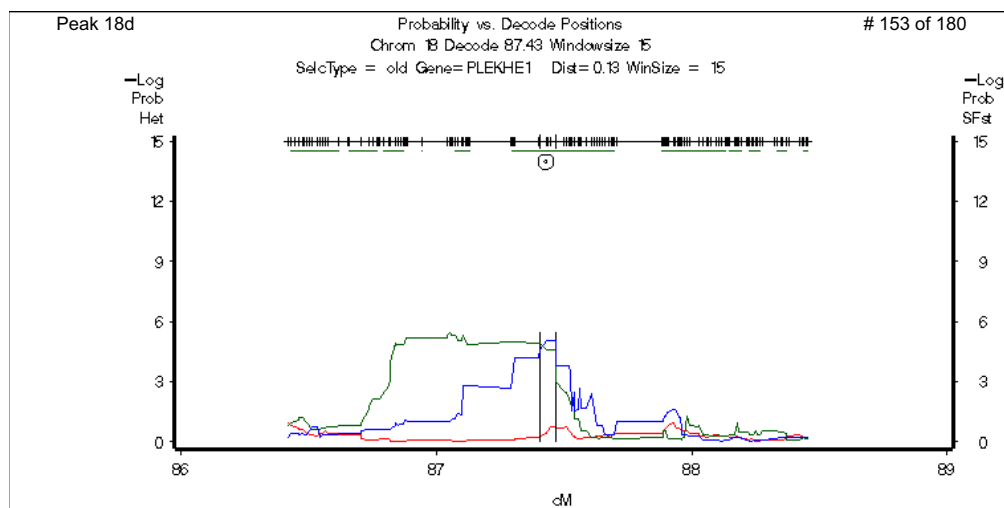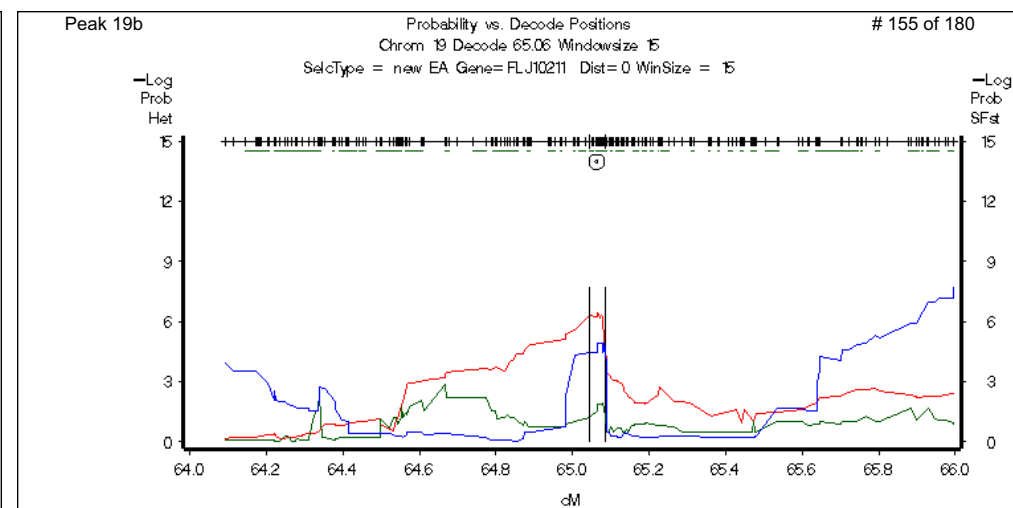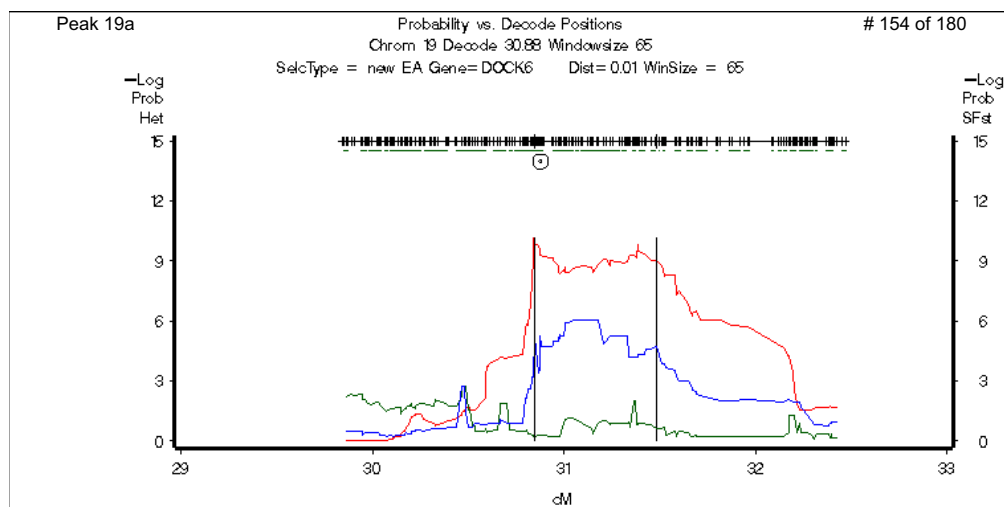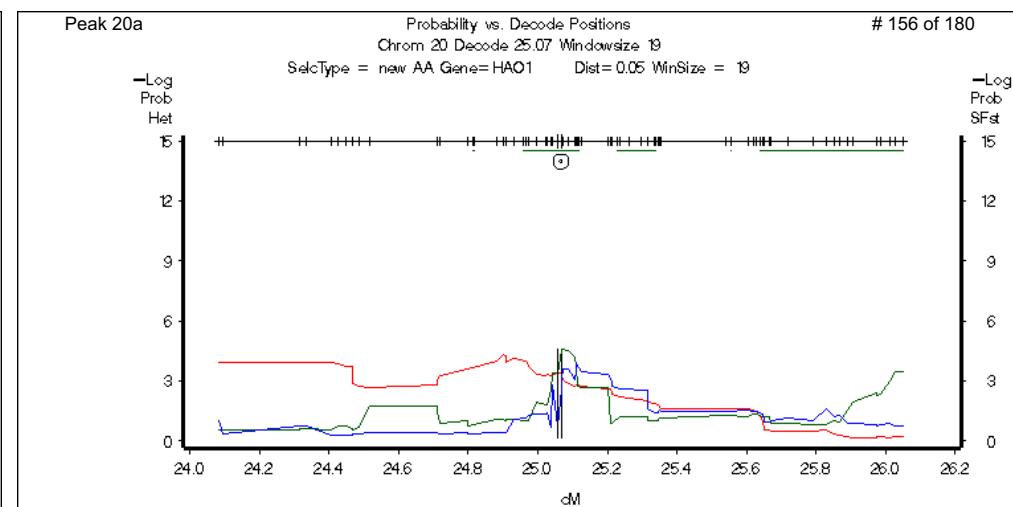

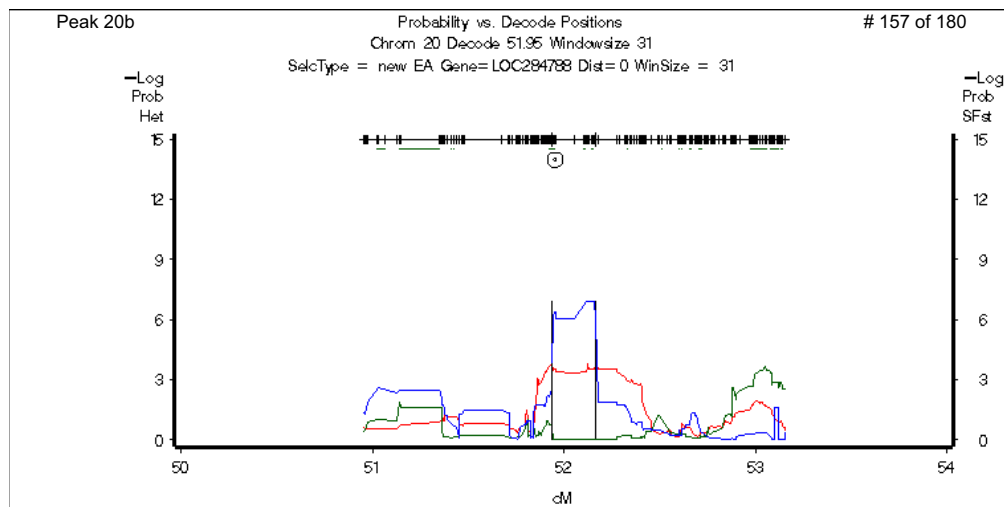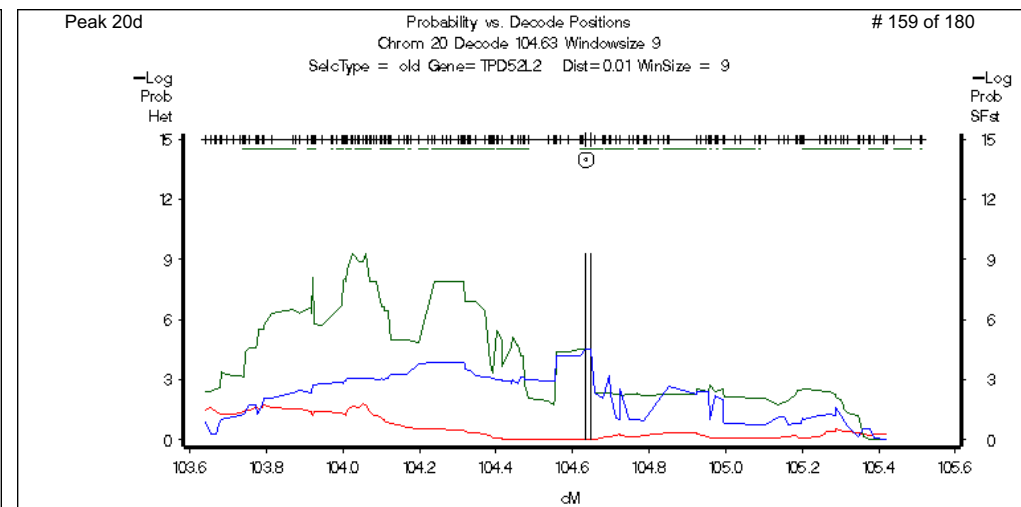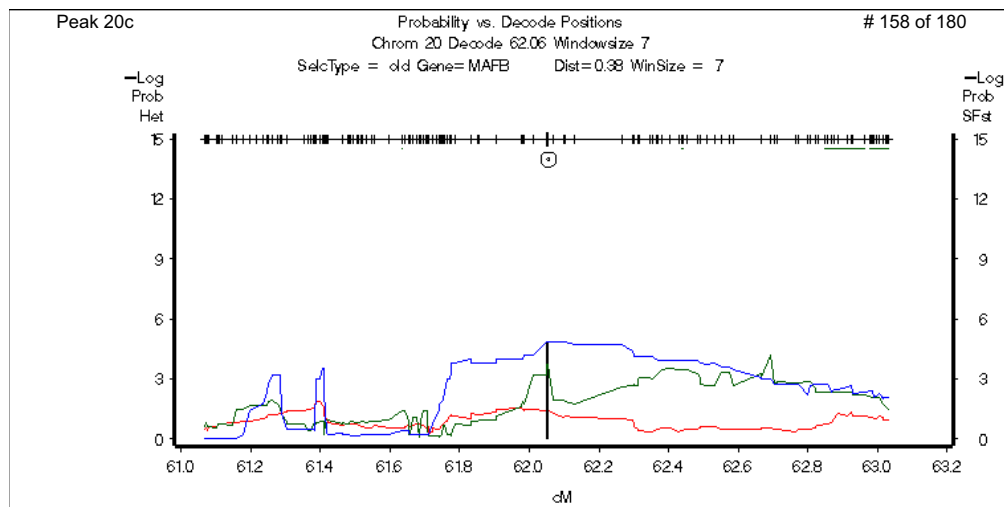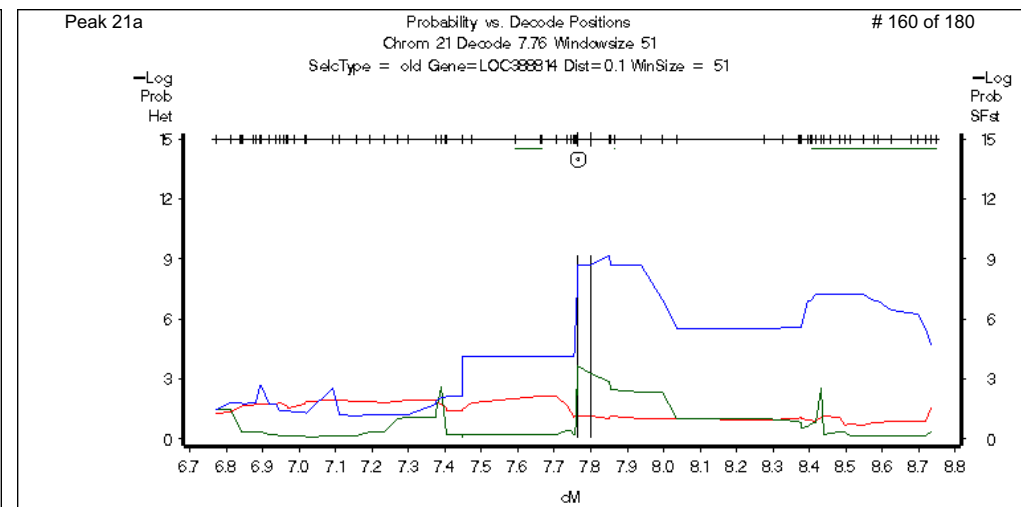

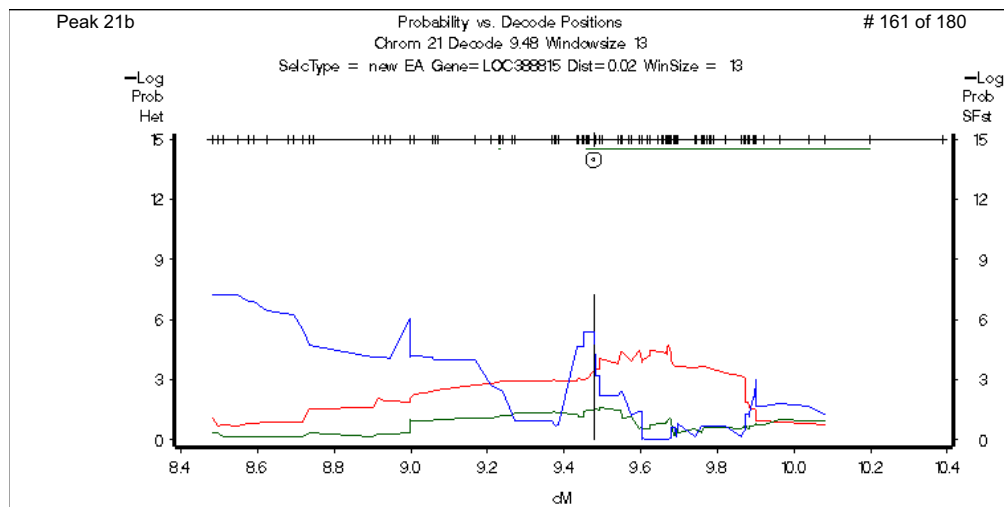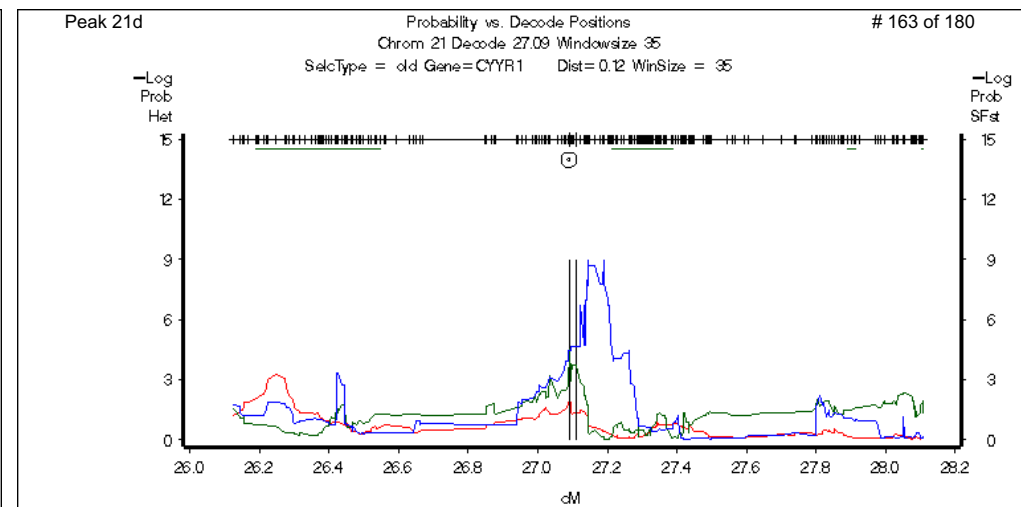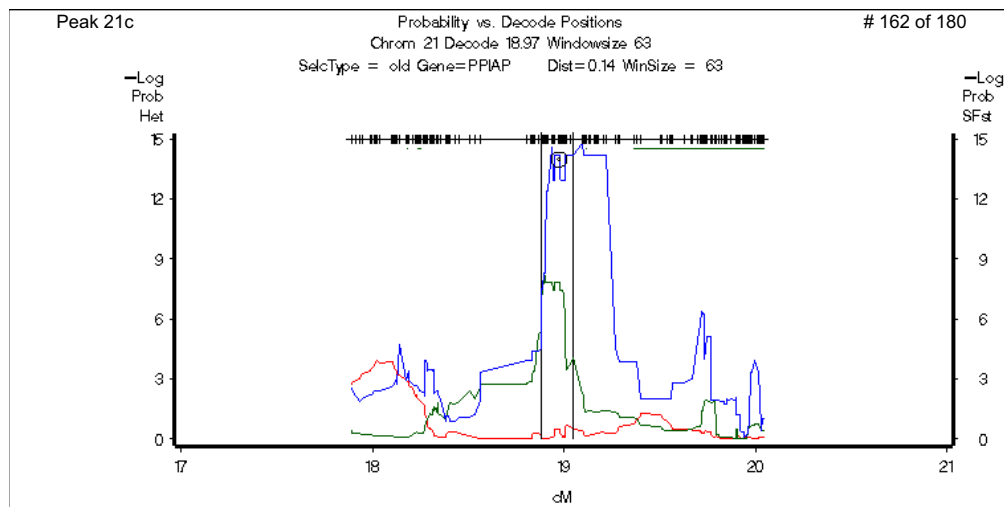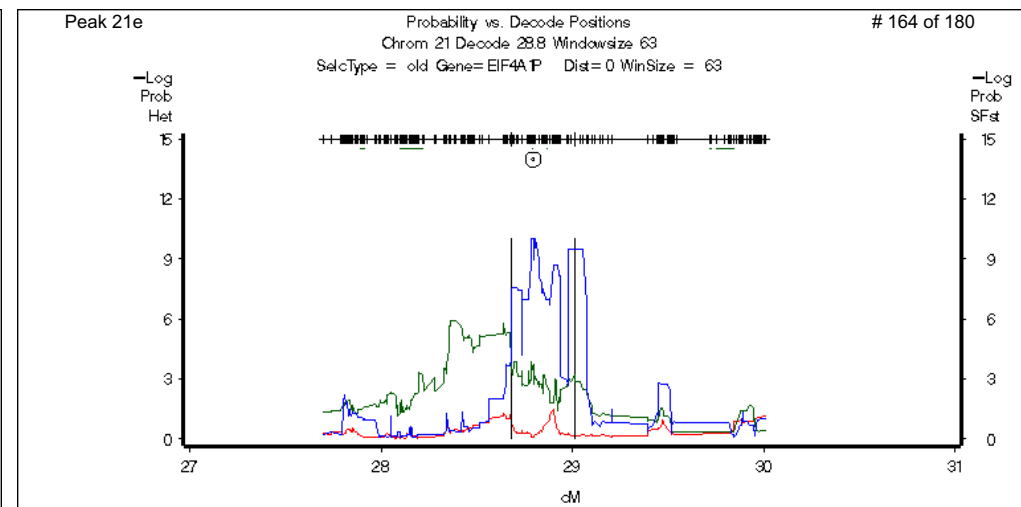

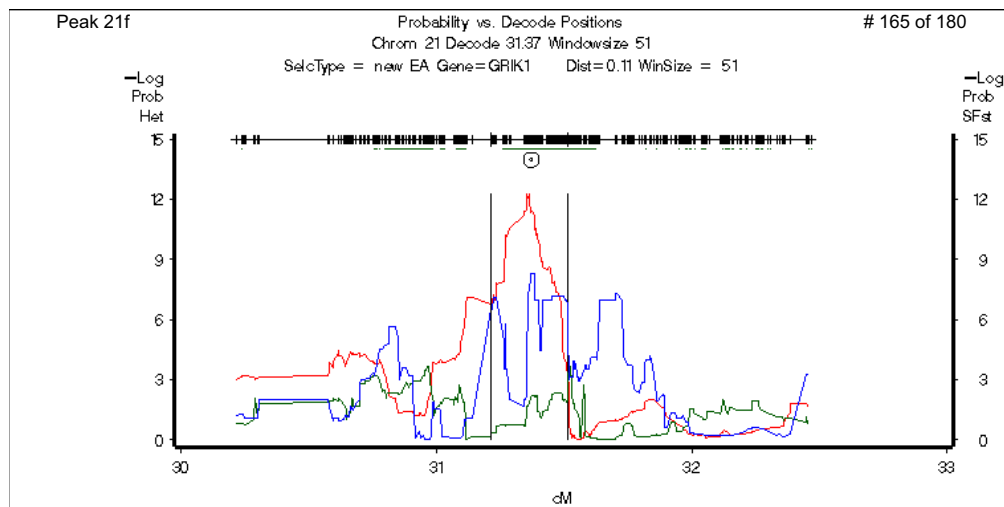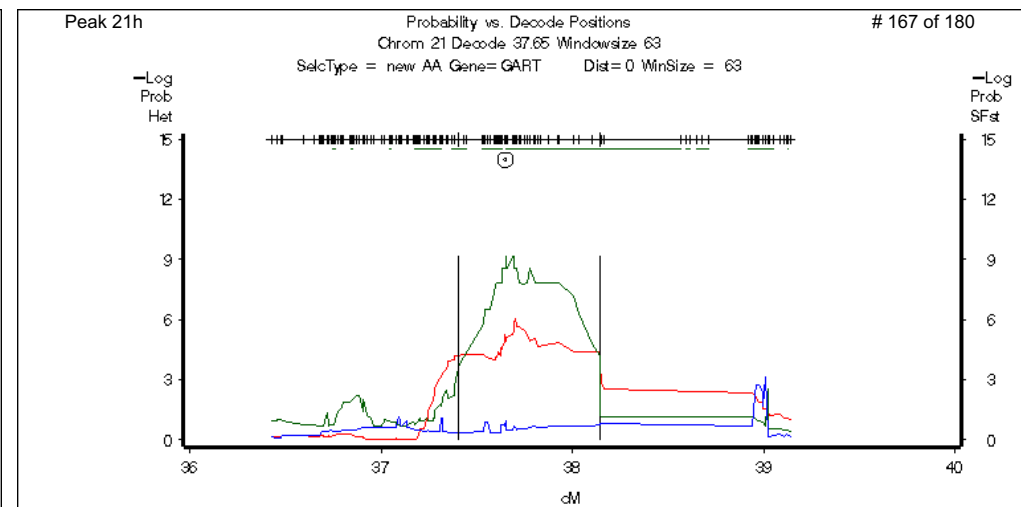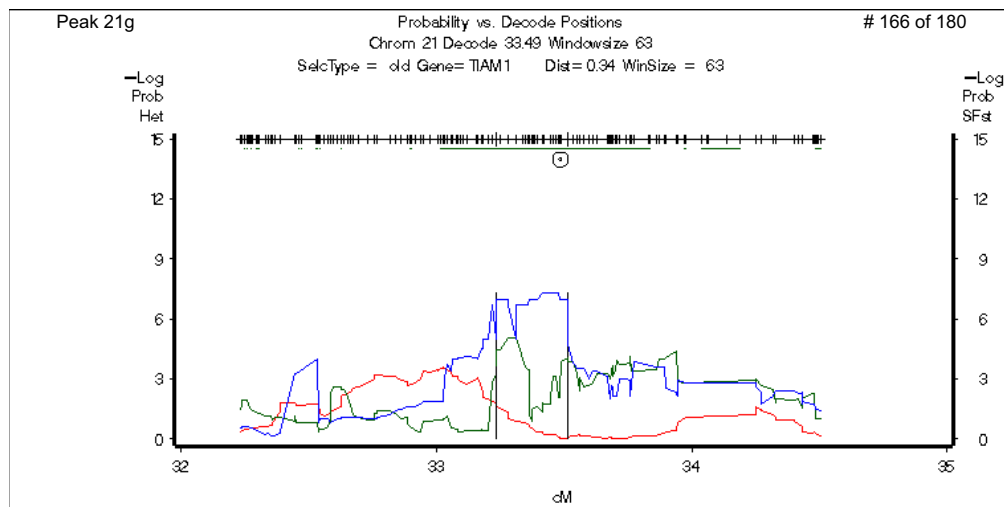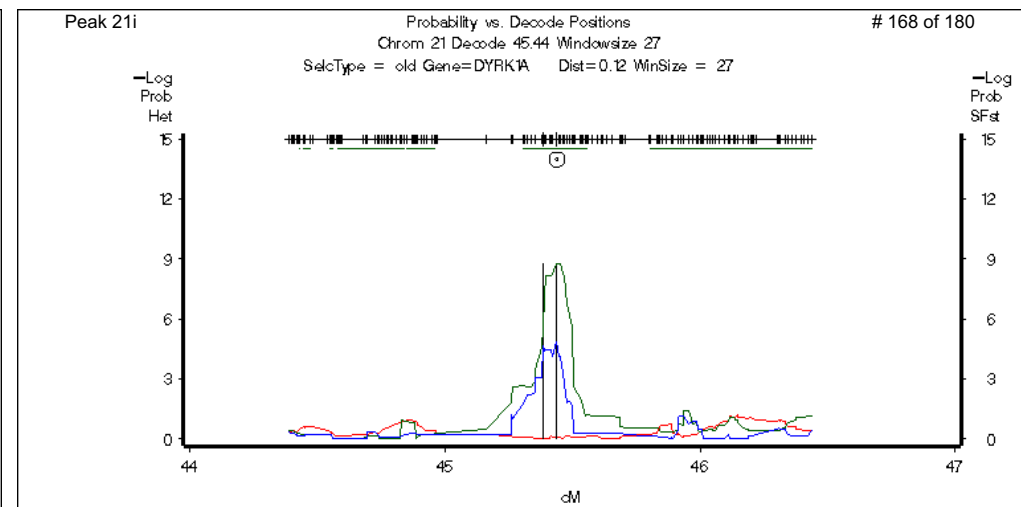

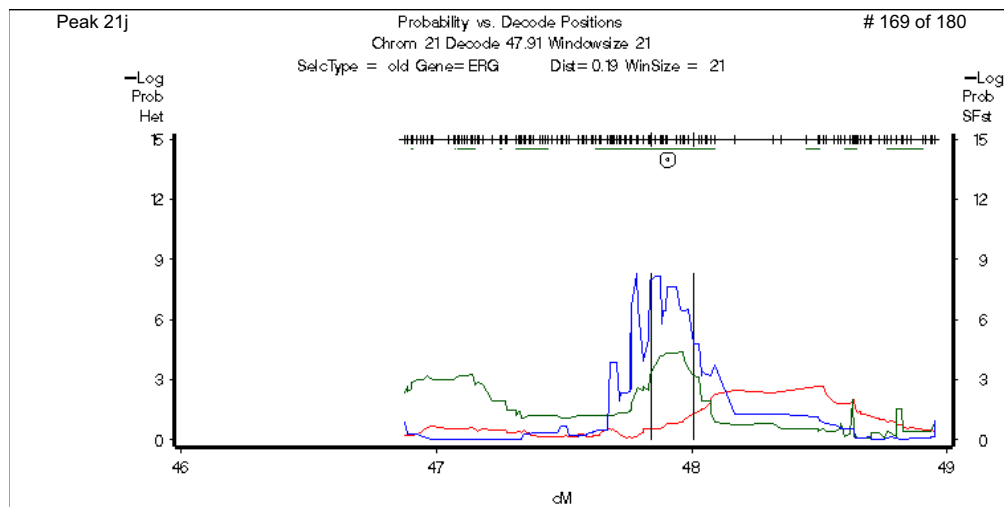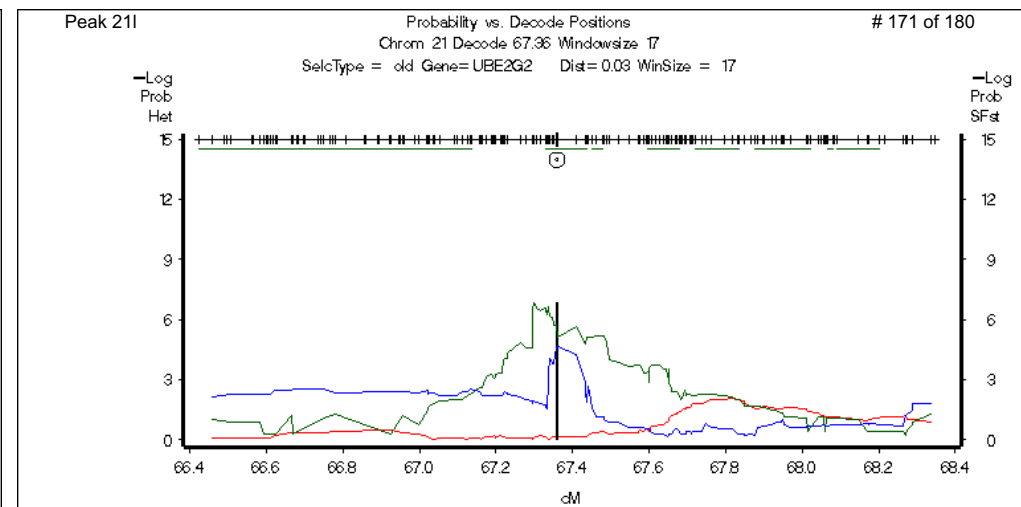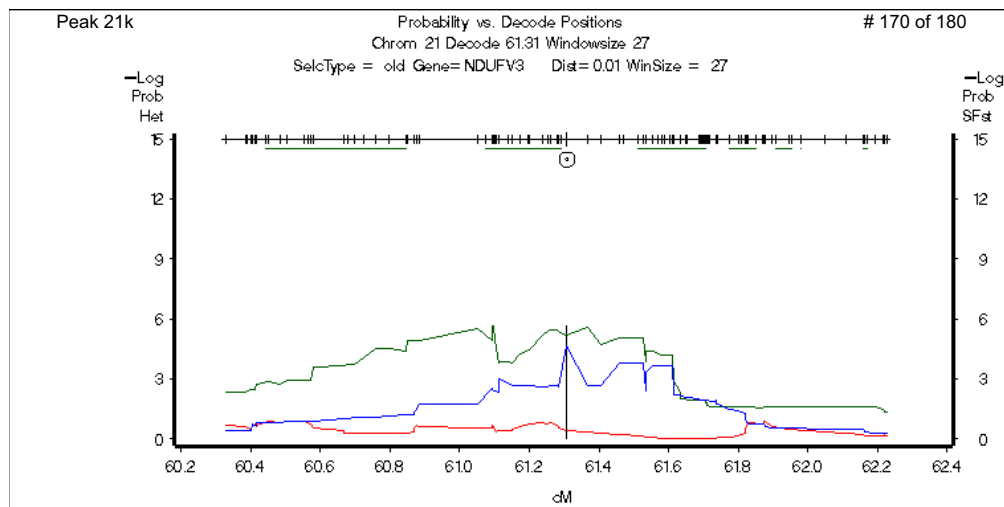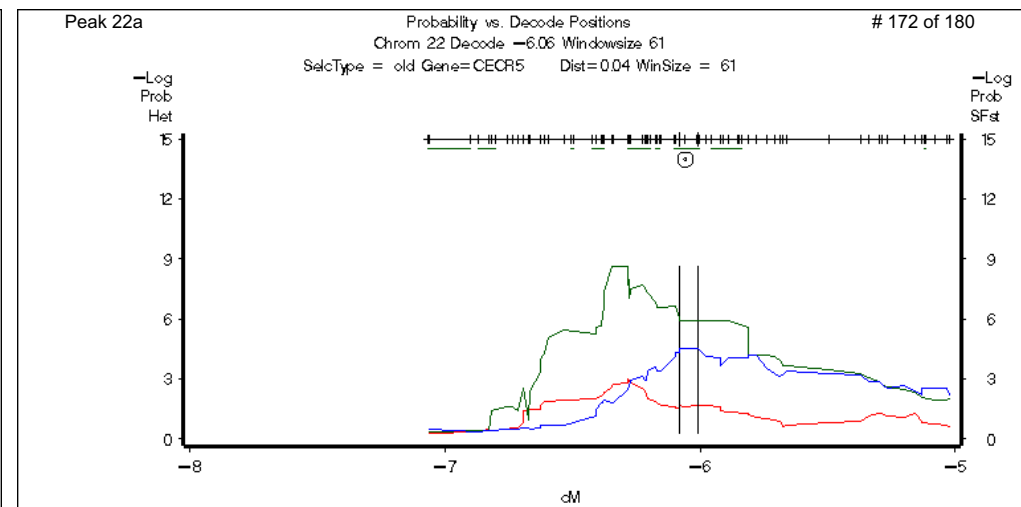

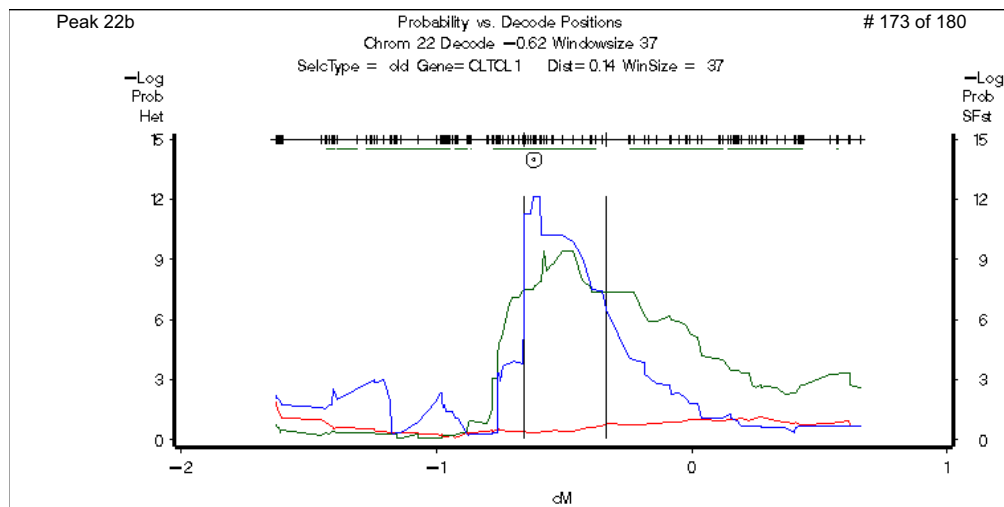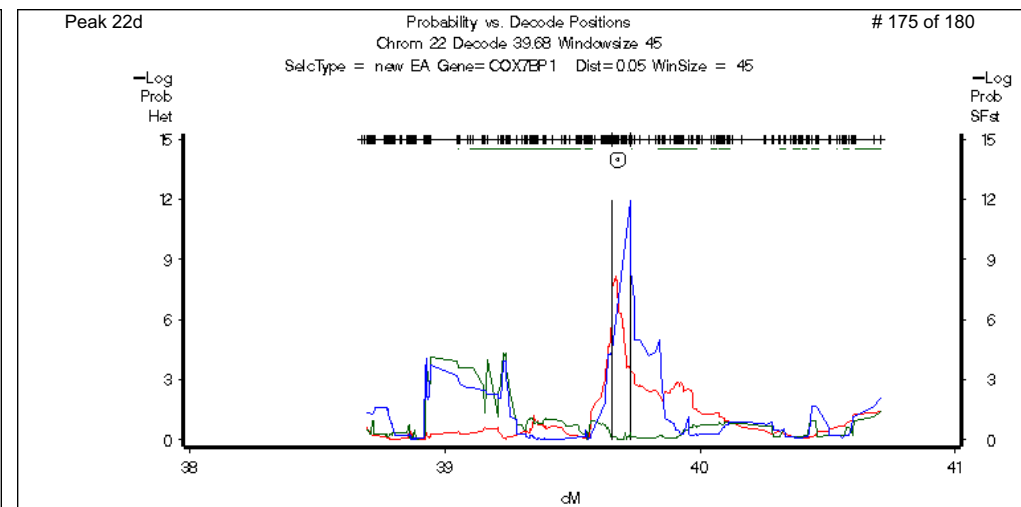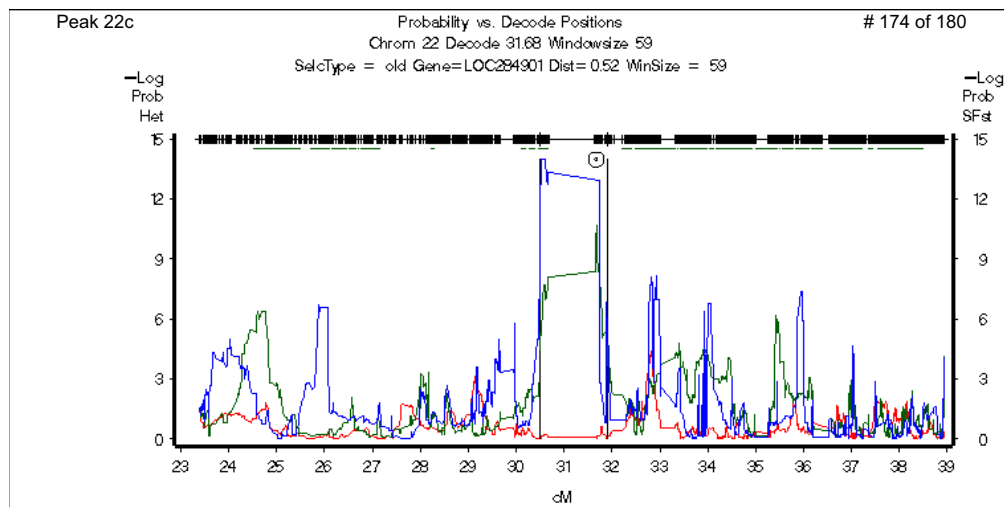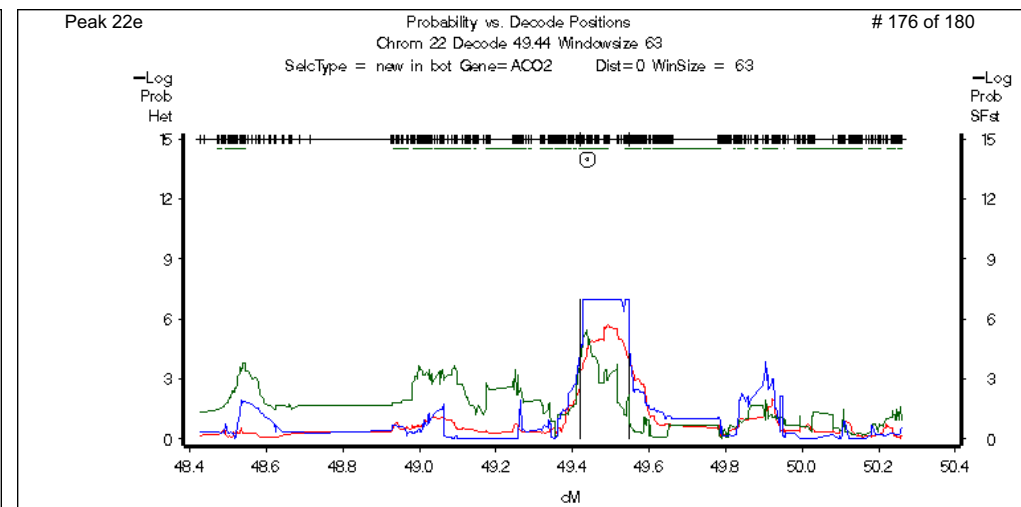

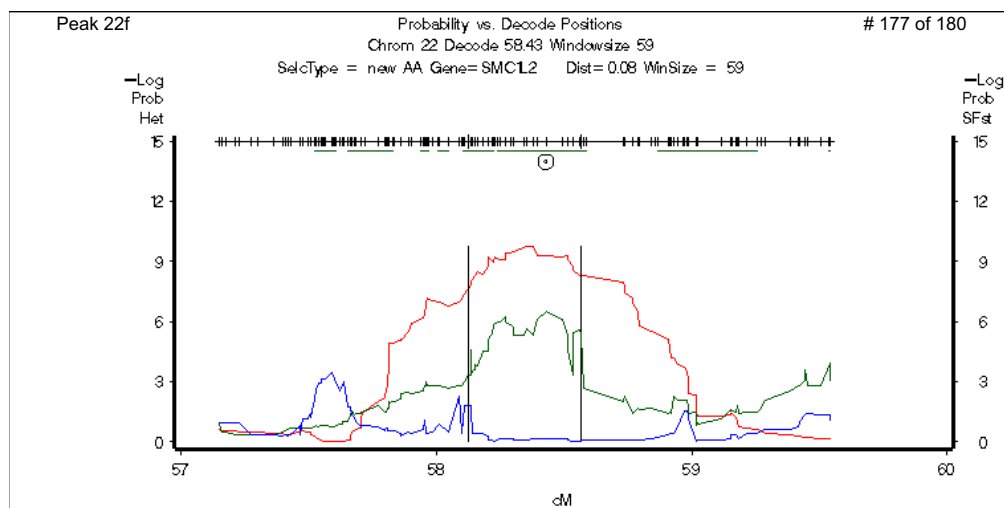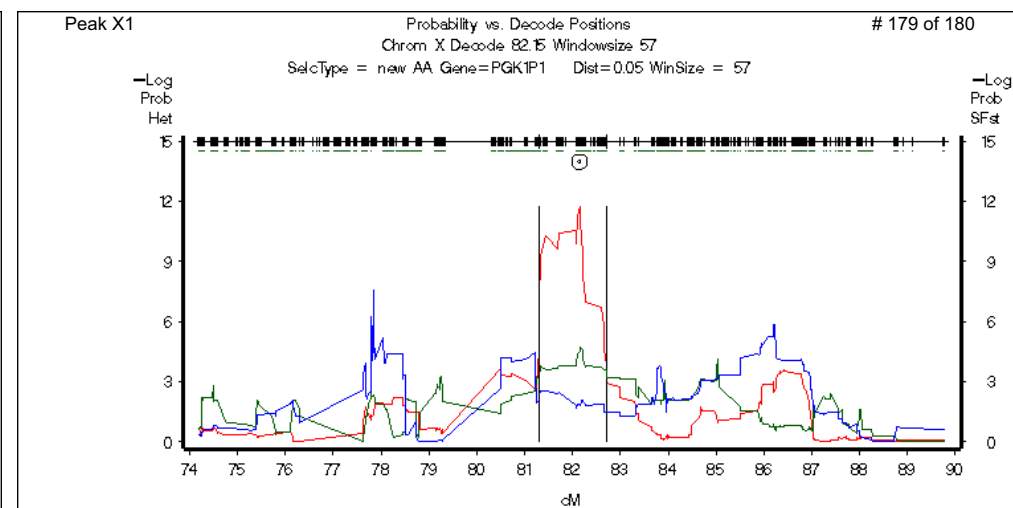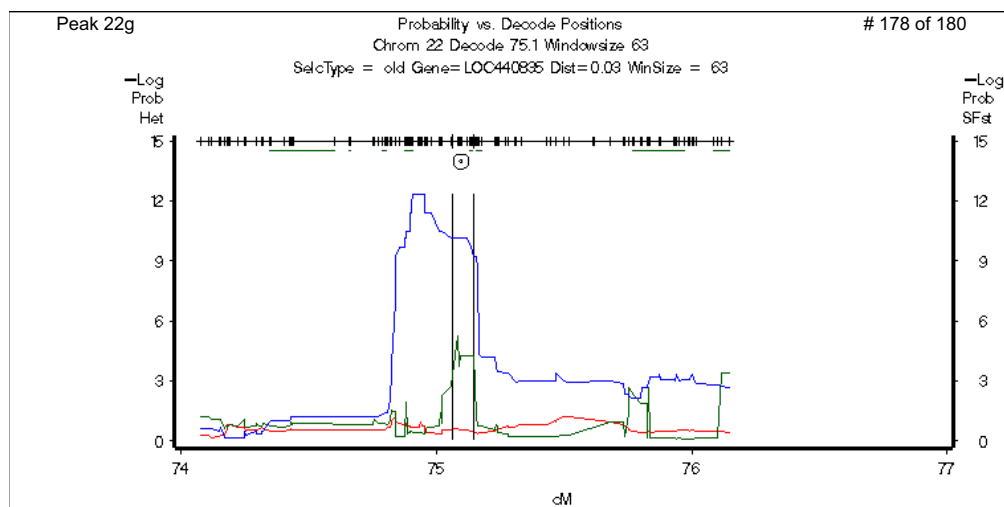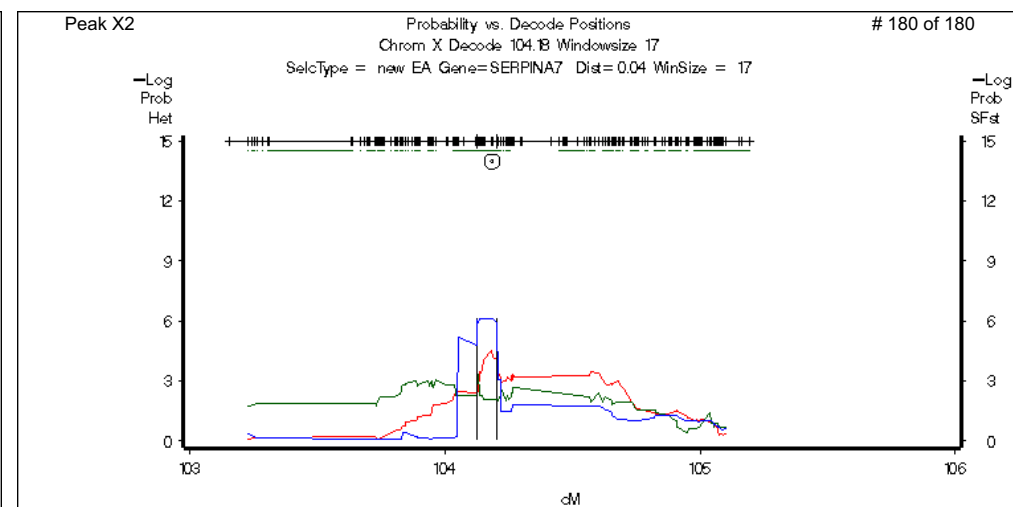

Supplement: Figure S1 — Individual graphs of 180 putative selection regions. Peak numbers correspond to Table S3 where the lowest mean rank value (λ), locations, and genes included in the putative selection regions are also shown. The vertical scale corresponds to the negative logarithm of the λ(HAA) (green line), λ(HEA) (blue line), and λ(S2FST) (red line). A putative selected site is identified where two of the peaks overlap (as in Figure 2A, bottom). The horizontal scale indicates location in cM. The locations of SNPs are represented by the black hash marks on the top of the graph. The extent of genes is represented by the horizontal blue line. Chromosome numbers (Chrom), location in cM, the most significant window size (out of 30 possible, windowsize), type of selection (SelcType), name of the closest gene (Gene), and the distance to it from the central location in cM (Dist) are all listed in the heading above each graph. The circle in the middle indicates the central location of the putative region. The range of the selected region is indicated by the two black vertical lines. The names of the genes included in the selected regions are listed from left to right in Table S3. (1.29 MB PDF) [file pone.0001712.s007.pdf]
