# Supplementary material for: Identifying Selected Regions from Heterozygosity and Divergence Using a Light-Coverage Genomic Dataset from Two Human Populations
Source: PLoS One. 2008 Mar 5;3(3):e1712. doi: 10.1371/journal.pone.0001712 (PMC2248624; doi:10.1371/journal.pone.0001712)
Supplement: Notes S2 — Figure S1 Notes (0.02 MB DOC) [file pone.0001712.s008.doc]

**Notes to Figure S1**

For each λ distribution, the upper 95% quantiles were: λ(Ĥ_EA_) = 3.8x10^-5^, λ(Ĥ_AA_) = 6.4x10^-4^, and λ(S^2^F_ST_) = 4.6x10^-4^. These threshold levels are indicated by the horizontal lines of the respective color at the beginning of each graph. Overlapping locations with λ values lower than these were classified as candidate regions for positive selection (Figure 2A). Suspected sites were inspected to pinpoint locations of genes, and to estimate sizes of gene neighborhoods that show selection signatures. Overall, 18 regions in African Americans and 77 in European Americans and 8 regions in both populations had strong evidence of recent selection, while ancestral selection was seen at 77 genomic regions as illustrated in Figure 1D (bottom) and shown in Figures 4A, 5 and 6.
