# Supplementary material for: Identifying Selected Regions from Heterozygosity and Divergence Using a Light-Coverage Genomic Dataset from Two Human Populations
Source: PLoS One. 2008 Mar 5;3(3):e1712. doi: 10.1371/journal.pone.0001712 (PMC2248624; doi:10.1371/journal.pone.0001712)
Supplement: Supplemental References S1 — (0.03 MB DOC) [file pone.0001712.s009.doc]

**Supplemental References**

1. Rockman MV, Hahn MW, Soranzo N, Goldstein DB, Wray GA (2003) Positive selection on a human-specific transcription factor binding site regulating IL4 expression. Curr Biol 13: 2118-2123.

2. Oota H, Pakstis AJ, Bonne-Tamir B, Goldman D, Grigorenko E, et al. (2004) The evolution and population genetics of the ALDH2 locus: random genetic drift, selection, and low levels of recombination. Ann Hum Genet 68: 93-109.

3. Sonnenburg JL, Altheide TK, Varki A (2004) A uniquely human consequence of domain-specific functional adaptation in a sialic acid-binding receptor. Glycobiology 14: 339-346.

4. Enard W, Przeworski M, Fisher SE, Lai CS, Wiebe V, et al. (2002) Molecular evolution of FOXP2, a gene involved in speech and language. Nature 418: 869-872.

5. Carrington M, Kissner T, Gerrard B, Ivanov S, O'Brien SJ, et al. (1997) Novel alleles of the chemokine-receptor gene CCR5. Am J Hum Genet 61: 1261-1267.

6. Angata T, Varki NM, Varki A (2001) A second uniquely human mutation affecting sialic acid biology. J Biol Chem 276: 40282-40287.

7. Holbrook JD, Birdsey GM, Yang Z, Bruford MW, Danpure CJ (2000) Molecular adaptation of alanine:glyoxylate aminotransferase targeting in primates. Mol Biol Evol 17: 387-400.

8. Tishkoff SA, Varkonyi R, Cahinhinan N, Abbes S, Argyropoulos G, et al. (2001) Haplotype diversity and linkage disequilibrium at human G6PD: recent origin of alleles that confer malarial resistance. Science 293: 455-462.

9. Bersaglieri T, Sabeti PC, Patterson N, Vanderploeg T, Schaffner SF, et al. (2004) Genetic signatures of strong recent positive selection at the lactase gene. Am J Hum Genet 74: 1111-1120.

10. Kouprina N, Pavlicek A, Mochida GH, Solomon G, Gersch W, et al. (2004) Accelerated evolution of the ASPM gene controlling brain size begins prior to human brain expansion. PLoS Biol 2: E126.

11. Osada N, Kusuda J, Hirata M, Tanuma R, Hida M, et al. (2002) Search for genes positively selected during primate evolution by 5'-end-sequence screening of cynomolgus monkey cDNAs. Genomics 79: 657-662.

12. Huttley GA, Easteal S, Southey MC, Tesoriero A, Giles GG, et al. (2000) Adaptive evolution of the tumour suppressor BRCA1 in humans and chimpanzees. Australian Breast Cancer Family Study. Nat Genet 25: 410-413.

13. Messier W, Stewart CB (1997) Episodic adaptive evolution of primate lysozymes. Nature 385: 151-154.

14. Hamblin MT, Di Rienzo A (2000) Detection of the signature of natural selection in humans: evidence from the Duffy blood group locus. Am J Hum Genet 66: 1669-1679.

15. Hamblin MT, Thompson EE, Di Rienzo A (2002) Complex signatures of natural selection at the Duffy blood group locus. Am J Hum Genet 70: 369-383. Epub 2001 Dec 2020.

16. Sabeti PC, Reich DE, Higgins JM, Levine HZ, Richter DJ, et al. (2002) Detecting recent positive selection in the human genome from haplotype structure. Nature 419: 832-837.

17. Wu W, Goodman M, Lomax MI, Grossman LI (1997) Molecular evolution of cytochrome c oxidase subunit IV: evidence for positive selection in simian primates. J Mol Evol 44: 477-491.

18. Bamshad MJ, Mummidi S, Gonzalez E, Ahuja SS, Dunn DM, et al. (2002) A strong signature of balancing selection in the 5' cis-regulatory region of CCR5. Proc Natl Acad Sci U S A 99: 10539-10544.

19. Sabeti PC, Walsh E, Schaffner SF, Varilly P, Fry B, et al. (2005) The Case for Selection at CCR5-Delta32. PLoS Biol 3: e378.

20. Huttley GA, Smith MW, Carrington M, O'Brien SJ (1999) A scan for linkage disequilibrium across the human genome. Genetics 152: 1711-1722.

21. Akey JM, Zhang G, Zhang K, Jin L, Shriver MD (2002) Interrogating a high-density SNP map for signatures of natural selection. Genome Res 12: 1805-1814.

22. Carlson CS, Thomas DJ, Eberle MA, Swanson JE, Livingston RJ, et al. (2005) Genomic regions exhibiting positive selection identified from dense genotype data. Genome Res 15: 1553-1565.

23. Nielsen R, Williamson S, Kim Y, Hubisz MJ, Clark AG, et al. (2005) Genomic scans for selective sweeps using SNP data. Genome Res 15: 1566-1575.

24. Altshuler D, Brooks LD, Chakravarti A, Collins FS, Daly MJ, et al. (2005) A haplotype map of the human genome. Nature 437: 1299-1320.

25. Voight BF, Kudaravalli S, Wen X, Pritchard JK (2006) A map of recent positive selection in the human genome. PLoS Biol 4: e72.

26. Wang ET, Kodama G, Baldi P, Moyzis RK (2006) Global landscape of recent inferred Darwinian selection for Homo sapiens. Proc Natl Acad Sci U S A 103: 135-140.

27. Frazer KA, Ballinger DG, Cox DR, Hinds DA, Stuve LL, et al. (2007) A second generation human haplotype map of over 3.1 million SNPs. Nature 449: 851-861.

28. Sabeti PC, Varilly P, Fry B, Lohmueller J, Hostetter E, et al. (2007) Genome-wide detection and characterization of positive selection in human populations. Nature 449: 913-918.
